# New [1,2,4]triazolo[4,3-*c*]quinazolines as intercalative Topo II inhibitors: Design, synthesis, biological evaluation, and *in silico* studies

**DOI:** 10.1371/journal.pone.0274081

**Published:** 2023-01-30

**Authors:** Ahmed A. Gaber, Mohamed Sobhy, Abdallah Turky, Wagdy M. Eldehna, Samiha A. El-Sebaey, Souad A. El-Metwally, Abeer M. El-Naggar, Ibrahim M. Ibrahim, Eslam B. Elkaeed, Ahmed M. Metwaly, Ibrahim H. Eissa

**Affiliations:** 1 Department of Pharmaceutical Organic Chemistry, Faculty of Pharmacy (Boys), Al-Azhar University, Cairo, Egypt; 2 Department of Pharmaceutical Chemistry, Faculty of Pharmacy, Kafrelsheikh University, Kafrelsheikh, Egypt; 3 Department of Pharmaceutical Organic Chemistry, Faculty of Pharmacy (Girls), Al-Azhar University, Cairo, Egypt; 4 Department of Basic Science, Higher Technological institute, 10th of Ramadan City, Egypt; 5 Department of Chemistry, Faculty of Science, Ain Shams University, Abassia, Cairo, Egypt; 6 Biophysics Department, Faculty of Science, Cairo University, Giza, Egypt; 7 Department of Pharmaceutical Sciences, College of Pharmacy, AlMaarefa University, Ad Diriyah, Riyadh, Saudi Arabia; 8 Pharmacognosy and Medicinal Plants Department, Faculty of Pharmacy (Boys), Al-Azhar University, Cairo, Egypt; 9 Biopharmaceutical Products Research Department, Genetic Engineering and Biotechnology Research Institute, City of Scientific Research and Technological Applications (SRTA-City), Alexandria, Egypt; 10 Pharmaceutical Medicinal Chemistry & Drug Design Department, Faculty of Pharmacy (Boys), Al-Azhar University, Cairo, Egypt; Albert Einstein Cancer Center: Albert Einstein Medical Center, UNITED STATES

## Abstract

Fifteen quinazoline derivatives were designed and synthesized as DNA intercalators. The cytotoxicity of the designed members was assessed against HCT-116 and HepG2 cancer cell lines. In addition, the topoisomerase II (Topo II) inhibitory effect was assessed. Compound **16** was the most cytotoxic and Topo II inhibitor with low cytotoxicity against Vero cells. Compounds **16**, **17**, and **18** showed significant DNA binding affinities. Compound **16** showed Topo II catalytic inhibitory effect at a concentration of 10 μM. Further mechanistic investigations revealed the capability of compound **16** to induce apoptosis in HCT-116 cells and arrest the growth at the S and G2/M phases. Also, compound **16** showed a significant increase in the level of BAX (2.18-fold) and a marked decrease in the level of Bcl-2 (1.9-fold) compared to the control cells. *In silico* studies revealed the ability of the synthesized members to bind to the DNA-Topo II complex.

## 1. Introduction

The discovery of new anticancer drugs that target DNA and topoisomerase II (Topo II) continues to pique medicinal chemists’ attention [1]. DNA intercalators are a significant class of DNA damaging agents [2]. The process of DNA intercalation causes substantial changes in DNA structure, such as elongation and stiffness, as well as changes in the helix twist angle [3]. Because many medications that block Topo II can also intercalate DNA, this class of bioactive anticancer treatments is referred to as intercalative Topo II inhibitors. Many studies have verified the apoptotic impact of intercalative Topo II inhibitors [4].

**Fig 1:**
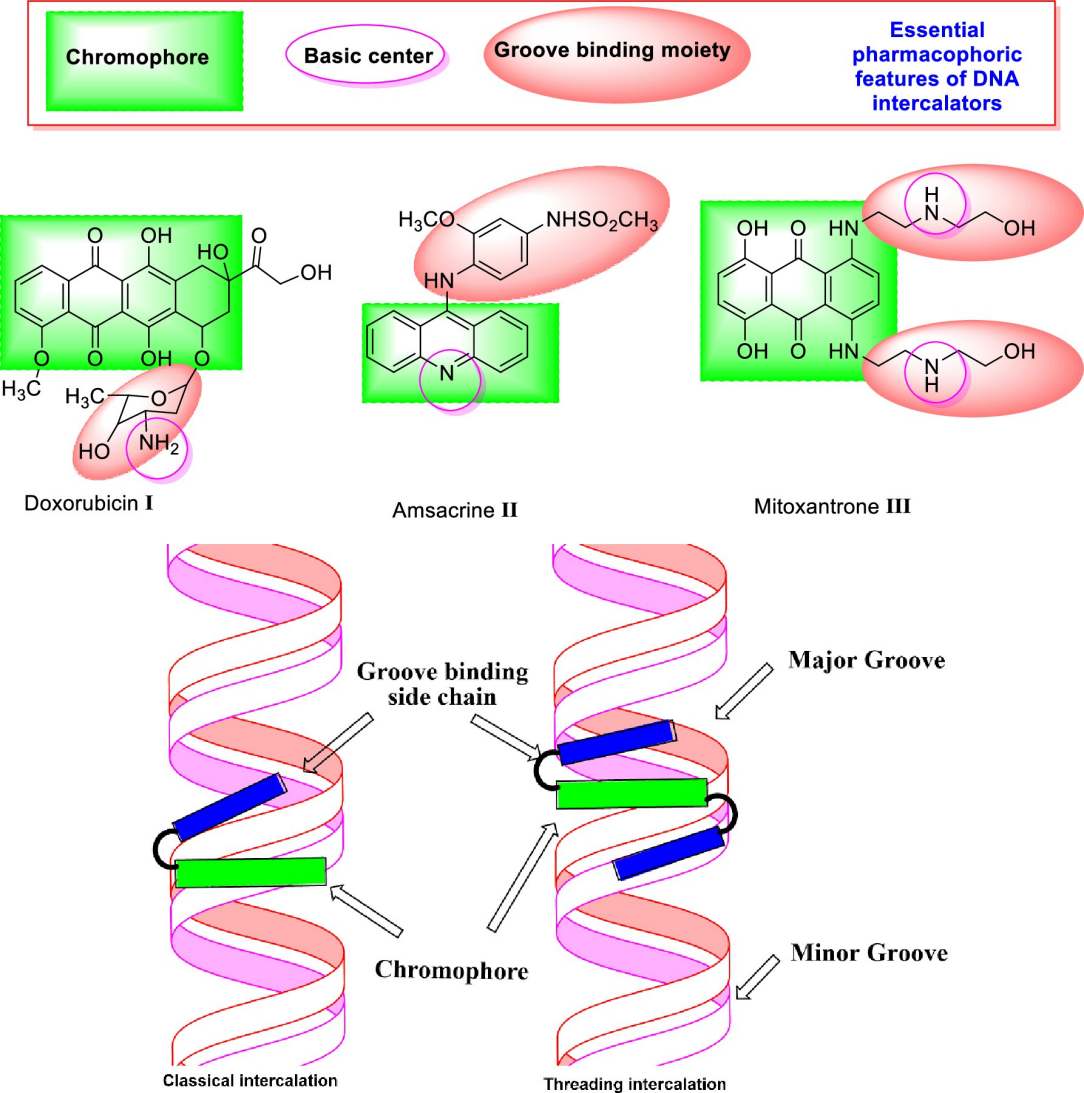
Some reported DNA intercalators and their basic pharmacophoric features. Schematic representation of classical and threading DNA intercalation (based on Ref. [17]).

Typical DNA intercalators are compounds that have the ability to be inserted between nucleotides of DNA to form a DNA-intercalator complex which can be stabilized by different hydrophobic stacking [5]. For high affinity, these compounds should have three pharmacophoric features which have an essential role in binding. i) The first feature is a planar polyaromatic system (chromophore) [6]. The second one is a cationic center to form ionic interaction with the phosphate moiety of the DNA. The third one is the groove binding side chain, which increases the binding affinity of the intercalator through the occupation of the DNA minor groove [7–9] (**Fig 1**).

There are two types of DNA intercalators. I) Classical intercalators, such as doxorubicin **I** and amsacrine **II**, that their entire aromatic system is inserted between the base pairs in the DNA helix [10]. These molecules have one groove binding side chain. II) Threading intercalators, such as mitoxantrone **III**, have a large, auxiliary, not intercalating groove binding side chain on both sides of the molecule. Classically, the flat aromatic system intercalates between base pairs, while bulky substituents are located in a minor and major DNA groove. One of these substituents must "go through" between base pairs causing a larger deformation of the helix structure and partial breakage of the double-stranded DNA (dsDNA) structure [11]. This process has very slow kinetics, and compounds of this type show higher affinity and lower rates of association and dissociation than other intercalators [12].

Topoisomerase II is a crucial cellular enzyme that modifies DNA topology. This enzyme is involved in several replicative processes such as chromosome replication, recombination, transcription, and segregation. It works by breaking the dsDNA and then resealing the breaks that have been produced [13]. Doxorubicin **I** [14], mitoxantrone **II** [15], and amsacrine **III** [16] are well-known examples of intercalative Topo II inhibitors (**Fig 1**).

For many years, our team synthesized several intercalative Topo II inhibitors which showed promising anticancer activities as compound **IV** which was evaluated as a classical DNA intercalator and Topo II inhibitor [18]. In addition, a quinoxaline-sulfonamide hybrid (compound **V**) showed good cytotoxic and Topo II inhibitory activities [17]. Recently, a new generation DNA intercalators and Topo II inhibitors were discovered by our team such as compounds **VI** [19], **VIIa**, **b** [20], **VIII** [21], and **IX** [22].

**Fig 2:**
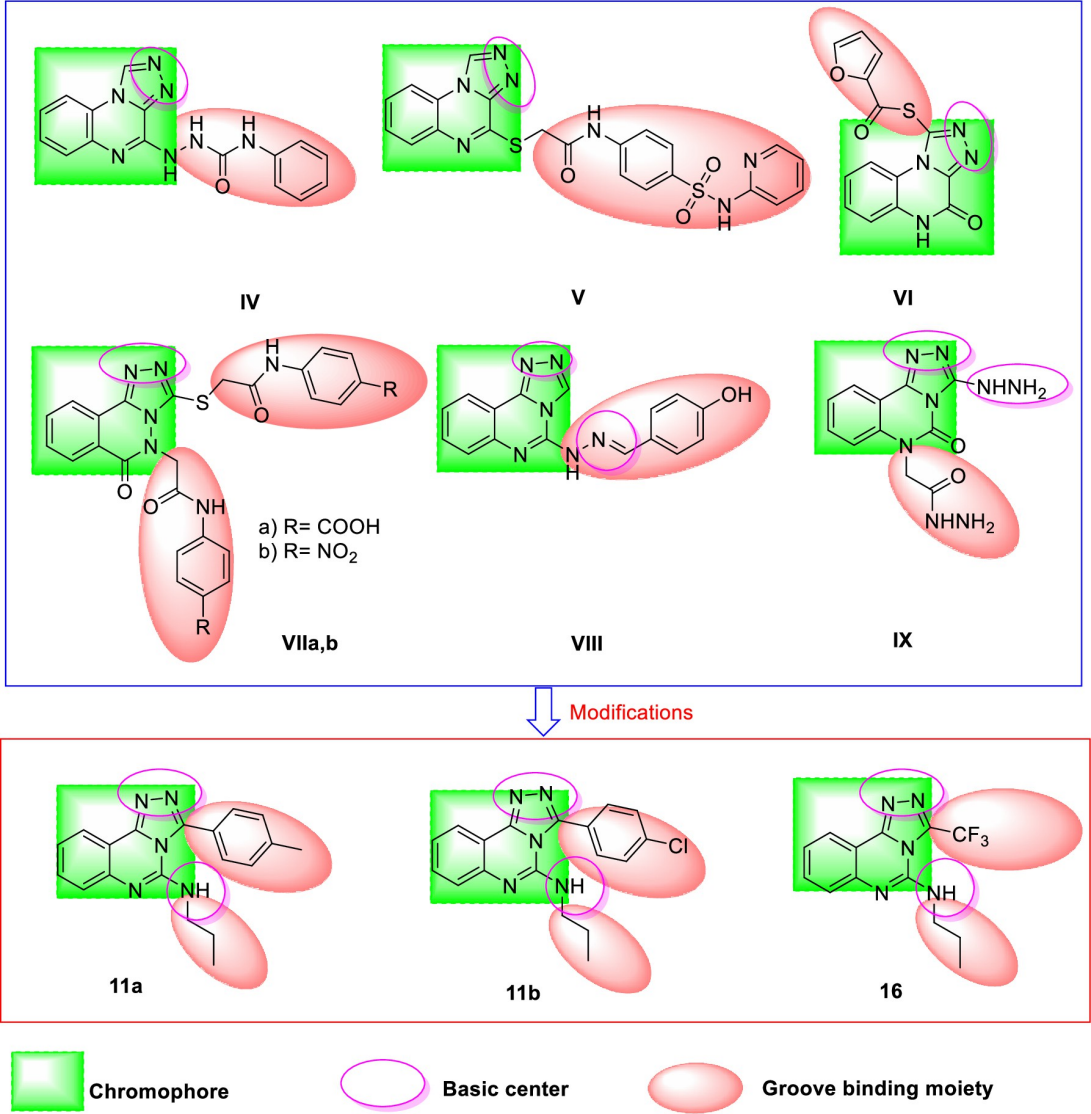
The rationale of molecular design of new DNA-intercalators.

In the current work, compounds (**III–IX**) were selected to be lead structures in the synthesis of new derivatives. The rationale of our molecular design depended on the lead modification of such compounds to get new scaffolds of threading DNA-intercalators. The [1,2,4]triazolo[4,3-*c*]quinazoline moiety was used as a planar aromatic system. Different aliphatic amines were used as the first groove binding side chain. The second groove binding side chain was 4-methylphenyl, 4-chlorophenyl, or 1,1,1-trifluoromethane moieties (**Fig 2**). The choice of the different derivatives was based on some chemical and biological considerations. The aryl groups might increase the planarity of the molecule for DNA intercalation and increase aromatic-aromatic stacking interaction with the binding site of the receptor. The chloro atom may exert halogen-halogen contacts or halogen bonding interaction with the receptor. A nonpolar methyl group may enhance hydrophobic interactions with the receptor. The trifluoromethyl group was reported to have positive effect on the activity of bioactive molecules. It can enhance several pharmacokinetic and physicochemical properties such as improved metabolic stability and enhanced membrane permeation. Increased binding affinity of fluorinated drug candidates to target protein has also been documented in a number of cases [23]. In this work. trifluoromethyl moiety was added to increase the lipophilicity of the synthesized compounds to facilitate the diffusion of these compounds across the cytoplasmic and nuclear membranes and interact with the DNA.

## 2. Result and discussion

### 2.1. Chemistry

**Scheme 1:**
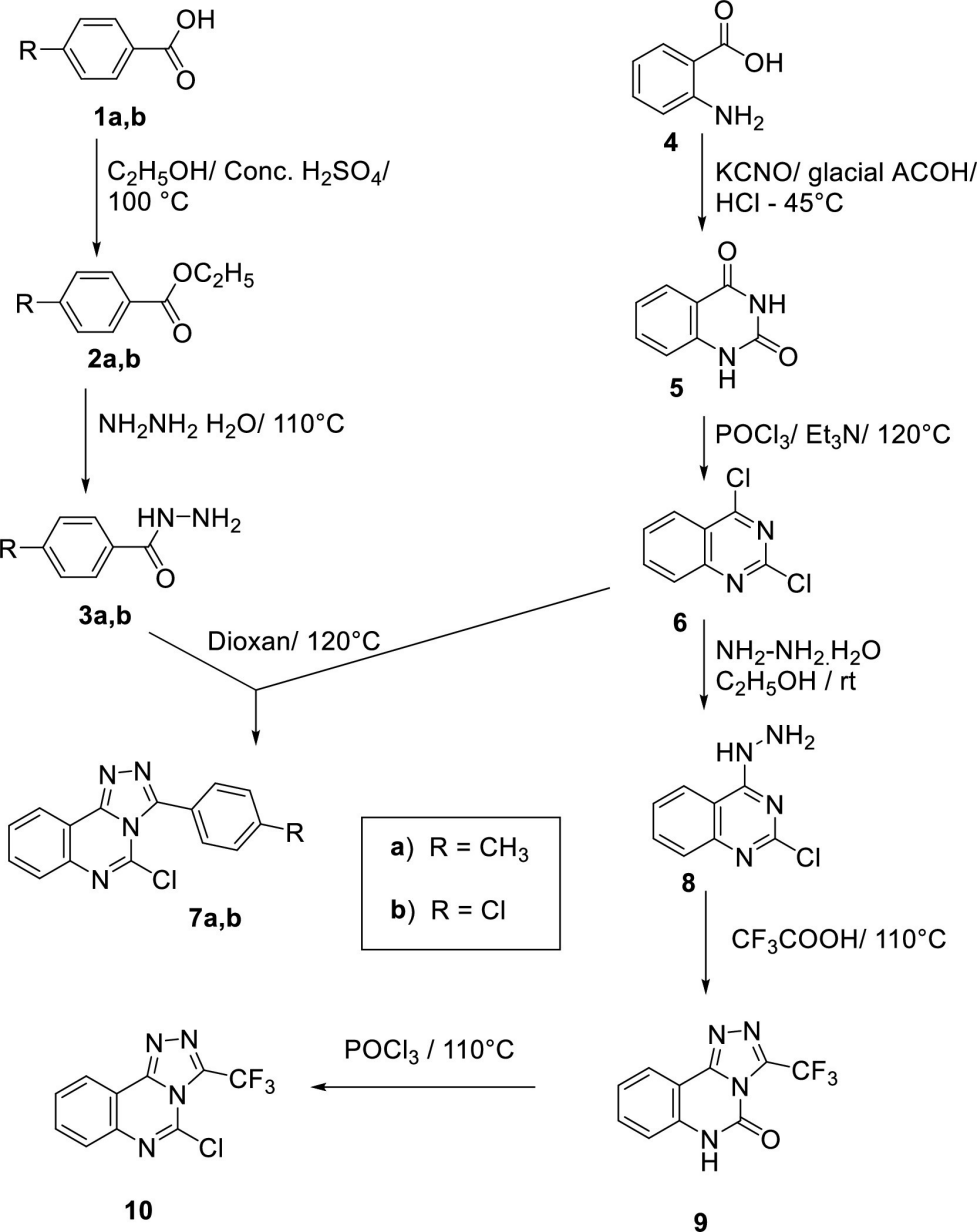
General procedures for the synthesis of the key compounds 7a, b, 9, and 10.

**Scheme 2:**
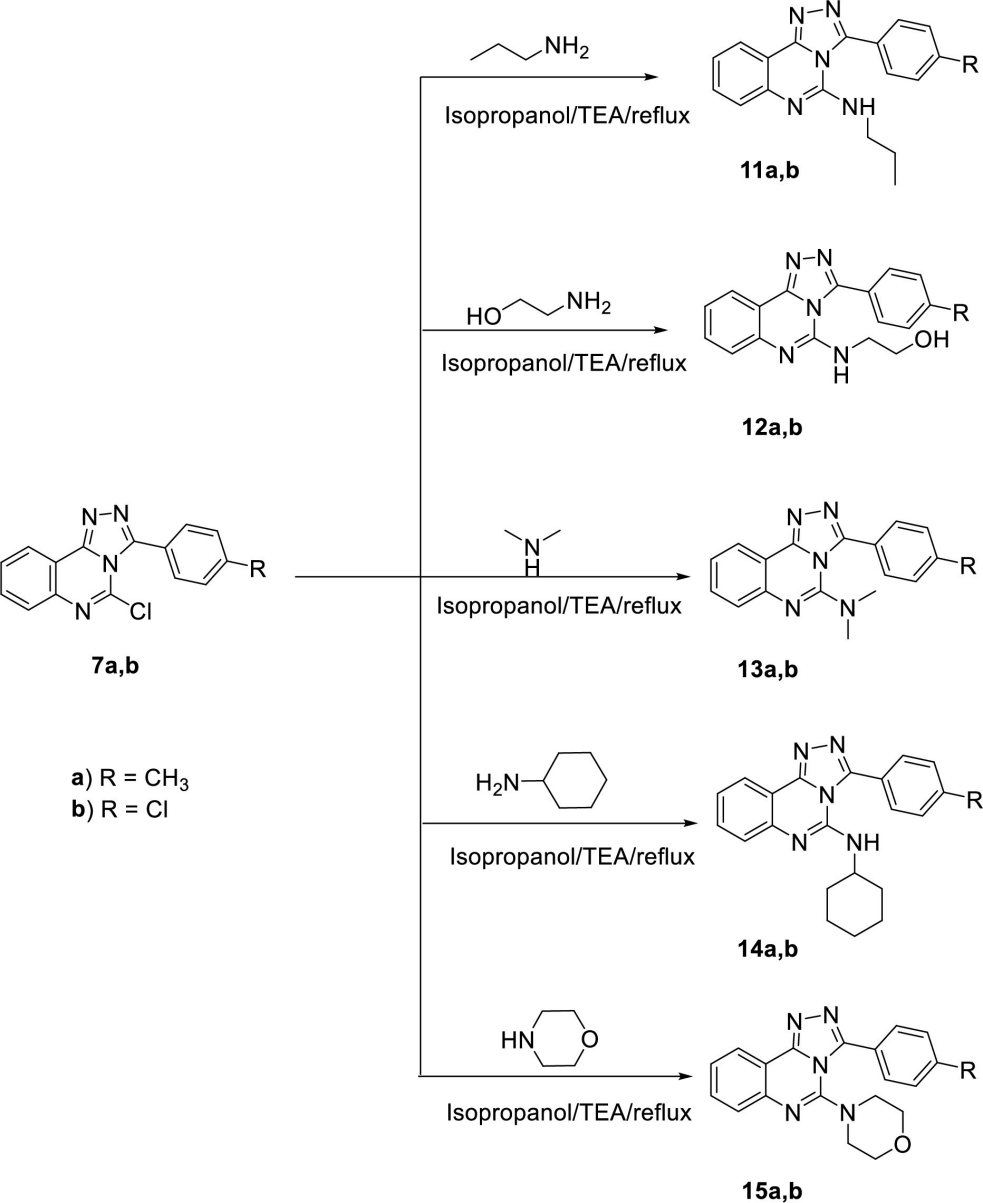
General procedure for the synthesis of the target compounds 11a,b,15a,b.

**Scheme 3:**
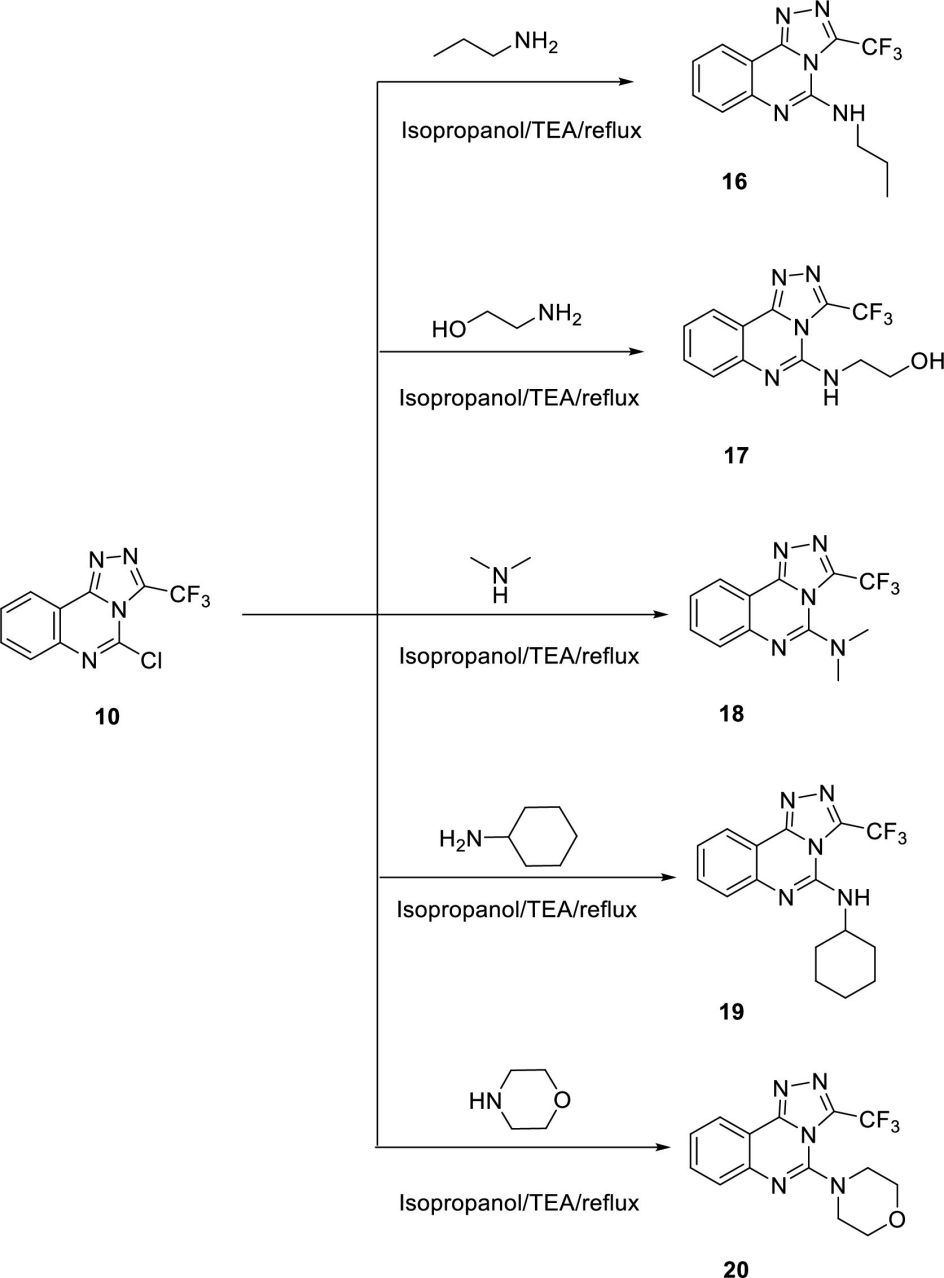
General procedure for the synthesis of the target compounds 16–20.

To synthesize the designed compounds, **Schemes 1–3** were adopted. Esters derivatives **2a** and **2b** were obtained by the Fischer esterification reaction of the commercially available 4-methylbenzoic acid **1a** and 4-chlorobenzoic acid **1b** with absolute ethanol in the presence of the dehydrating conc. Sulfuric acid as a catalyst [24, 25]. Condensation of the above ester derivatives with hydrazine hydrate in refluxing ethanol, following the reported procedures, furnished the target hydrazide derivatives **3a,b** [24, 25]. In a parallel pathway, anthranilic acid **4** was heated in an aqueous solution of potassium cyanate with a catalytic amount of glacial acetic acid to produce quinazoline-2,4(1*H*,3*H*)-dione **5** [24, 25]. Chlorination of **5** using phosphorus oxychloride and triethyl amine (TEA) afforded 2,4-dichloroquinazoline **6** [24, 25]. Cyclocondensation of 2,4-dichloroquinazoline **6** with benzohydrazide derivatives **3a** and **3b** in dioxane afforded the corresponding key compounds **7a** and **7b** [24, 25]. The addition of hydrazine hydrate dropwise to the 2,4-dichloroquinazoline **6** at 0–5°C afforded 2-chloro-4-hydrazinylquinazoline **8** [24, 25] which was reacted with trifluoroacetic acid to afford 3-(trifluoromethyl)-[1,2,4]triazolo[4,3-c]quinazolin-5-one **9.** Chlorination of **9** using phosphorus oxychloride at 110°C afforded the desired key compound **10** [24, 25] (**Scheme 1**).

The final target compounds (**11a**,**b, 15a**,**b**, and **16**–**20**) were obtained by refluxing the key compounds **7a,b**, and **10** in isopropyl alcohol with appropriate amines namely, propylamine, 2-ethanolamine, dimethylamine, cyclohexylamine, and morpholine (**Schemes 2** and **3**)

^1^H NMR analysis of compound **12a**, as a representative example, exhibited the appearance of a characteristic singlet signal for CH_3_ at *δ* 2.41 ppm. Also, it showed two signals for the CH_2_ groups at *δ* 3.85 and 3.79 ppm. Besides, the NH group was detected at *δ* 4.85 ppm. ^13^C NMR spectrum of compound **16** exhibited characteristic peaks at the aliphatic region (*δ* 43.62, 21.33, and 12.02ppm) for the propyl moiety.

### 2.2. Biological evaluation

#### 2.2.1. *In vitro* anti-proliferative activity

*In vitro* antiproliferative activities of the target molecules were assessed against two human cancer cell lines namely, hepatocellular carcinoma (HepG2) and colorectal carcinoma (HCT-116) using MTT assay [26–28]. Doxorubicin, a typical Topo II inhibitor, was utilized as a positive control. The growth inhibitory concentration (IC_50_) values were summarized in **Table 1**.

**Table 1:** The antiproliferative activities of the tested compounds toward HepG2, and HCT‐116 cell lines, Topo II activity, and DNA‐intercalating affinity.

Comp.	*In vitro* cytotoxicity IC_50_ (μM) ^a^		Topo II (IC_50,_ μM)^b^	DNA/Methyl green (IC_50,_ μM)^c^
	HepG2	HCT-116		
**11a**	15.47 ± 1.0	8.42 ± 0.3	NT	NT
**11b**	29.95 ± 1.3	24.99 ± 0.9	NT	NT
**12a**	18.75 ± 1.2	11.94 ± 0.3	NT	NT
**12b**	17.81 ± 0.9	7.03 ± 0.2	NT	NT
**13a**	10.65 ± 0.6	11.62 ± 0.5	36.07 ± 1.9	52.87 ± 1.6
**13b**	33.1 ± 1.7	16.86 ± 0.9	NT	NT
**14a**	13.02 ± 0.5	7.66 ± 0.2	30.37 ± 1.8	22.54 ± 0.9
**14b**	29.53 ± 1.2	17.44 ± 0.4	NT	NT
**15a**	20.37 ± 0.9	19.97 ± 0.4	NT	NT
**15b**	23.44 ± 0.9	21.01 ± 0.3	NT	NT
**16**	6.29 ± 0.3	2.44 ± 0.1	15.16 ± 0.9	10.25 ± 0.4
**17**	5.65 ± 0.1	2.78 ± 0.1	17.66 ± 0.8	11.09 ± 0.7
**18**	6.82 ± 0.2	4.17 ± 0.1	18.28 ± 0.8	12.54 ± 0.6
**19**	8.91 ± 0.2	9.43 ± 0.3	18.66 ± 0.9	23.58 ± 1.3
**20**	8.34 ± 0.3	4.61 ± 0.2	20.65 ± 0.9	34.51 ± 1.2
**Doxorubicin**	3.80 ± 0.1	1.87 ± 0.1	8.23 ± 0.4	5.64 ± 0.1
**VIIa**	3.91 ± 0.15	2.62 ± 0.10	7.45 ± 0.44	48.30 ± 2.30

^a^ IC_50_ values of the *in-vitro* anti-proliferative activities of the tested compounds against HepG2 and HCT-116 cell lines from three independent experiments.

^b^ 50% inhibition of Topoisomerase II (Topo II) activity

^c^ 50% inhibition concentration values of DNA/methyl green assay.

NT: Compounds not tested.

The tested molecules showed different degrees of biological activities compared the reference drug. Doxorubicin exhibited IC_50_ values of 3.80 and 1.87 μM against HepG2, and HCT-116, respectively. The activity of compounds **11–15** was generally weaker against HepG2 compared to HCT-116. Compounds **11a, 12b**, and **14a** showed moderate activity against the HCT-116 cell line.

Compounds **16**, **17**, **18**, **19**, and **20** showed significant cytotoxic activities against the two cell lines with IC_50_ values ranging from 2.44 to 9.43 μM. These results indicate that the substitution of [1,2,4]triazolo[4,3-*c*]quinazoline with trifluoromethyl moiety is beneficial for cytotoxic activity. This may be attributed to the ability of the trifluoromethyl moiety to form extra hydrogen and hydrophobic bonds and with the target receptor and consequently increase the binding affinity and activity. In addition, the trifluoromethyl moiety increases the lipophilic characters of the synthesized compounds which may facilitate the diffusion of these compounds across the cytoplasmic and nuclear membranes and interact with the DNA.

Compound **16** was the most active member showing IC_50_ values of 6.29 and 2.44 μM against HepG2 and HCT-116, respectively. The five most active derivatives were arranged in cytotoxic activity in the descending order of **16 >17 >18 > 20 >19**. This indicates that the substitution of [1,2,4]triazolo[4,3-*c*]quinazoline with different amines affects the biological activity in the descending order of propylamine > ethanolamine > dimethylamine > morpholine > cyclohexylamine. This indicates that the increase bulky structure of amines produced negative effect in the cytotoxic activity. This may be attributed to the high a chance of the open chain aliphatic groups to be oriented into the minor groove of the DNA and hence increase the binding affinity and cytotoxic activity.

Additionally, compounds **11a**, **12b**, and **14a** showed significant activities against only HCT-116 cell lines with IC_50_ values 8.42, 7.03, and 7.66 μM, respectively. Compounds **12a** and **13a** showed moderate cytotoxic activities against the two cell lines, while compounds **11a**, **12b**, and **14a** showed moderate activities against only HepG2 cell lines. Also, compounds **13b**, **14b**, and **15a** showed moderate activities against only HCT-116. Finally, compounds **11b** and **15b** showed weak activities against the two test cell lines.

#### 2.2.2. Structure-Activity Relationship (SAR)

Observing the cytotoxic activities of compounds **11a, 12a, 13a, 14a,** and **15a** incorporating toluene as groove binding side chain with the activity of the corresponding derivatives with chlorobenzene (**11b, 12b, 13b, 14b,** and **15b**) and that incorporating 1,1,1-trifluoromethane moiety (**16, 17, 18, 19,** and **20**), it was found that 1,1,1-trifluoromethane moiety is more advantageous than other moieties with higher priority for chlorobenzene moiety over the toluene one.

In addition, it was found that the different amines affect the biological activity in the descending order of propylamine > ethanolamine > dimethylamine > morpholine > cyclohexylamine when we compared the cytotoxic activity of compounds **16, 17, 18, 19,** and **20.**

#### 2.2.3. Topoisomerase II inhibitory activity

Compounds **13a**, **14a**, **16**, **17**, **18**, **19**, and **20** which showed promising cytotoxicity were further tested for their inhibitory effect against Topo II. Doxorubicin as a potent Topo II inhibitor was utilized as a reference drug. The results were summarized in **Table 1.**

The tested compounds inhibited Topo II activity to different degrees compared to doxorubicin (8.23 μM). Compounds **16**, **17**, **18**, **19**, and **20** were the most active Topo II inhibitors with IC_50_ values of 15.16, 17.66, 18.28, 18.66, and 20.65 μM, respectively. On the other hand, compounds **13a** and **14a** showed weak Topo II inhibitory activities with IC_50_ values of 36.07 and 30.37 μM, respectively. These results were consistent with the results of the *in vitro* anti-proliferative activities.

#### 2.2.4. DNA intercalation assay (DNA/methyl green assay)

The most promising members (**13a**, **14a**, **16**, **17**, **18**, **19**, and **20)** were further investigated for their binding affinity against DNA. DNA/methyl green assay [29] was applied utilizing doxorubicin as a positive control. The results of DNA-binding affinities were reported in **Table 1** as IC_50_ values calculated from the concentration-inhibition response curve.

The tested compounds exhibited strong, moderate, and weak DNA-binding affinities with IC_50_ values ranging from 10.25 to 52.87 μM, compared to doxorubicin (IC_50_ = 5.64 μM). Compounds **16**, **17**, and **18** exhibited significant DNA binding affinities with IC_50_ values of 10.25, 11.09, and 12.54 μM, respectively. Compounds **14a**, **19**, and **20** showed moderate IC_50_ values of 22.54, 23.58, and 34.51μM, respectively. On the other hand, compound **13a** showed a weak DNA binding affinity with an IC_50_ value of 52.87 μM.

Comparing the activity of the most active compound **16** with that of the most active compound ([1,2,4]triazolo[3,4-*a*]phthalazin-6(5*H*)-one derivative **VIIa**) in our previous work [20] revealed that compound **16** has higher cytotoxic activity against HCT-116 (2.44 μM) than **VIIa** (2.62 μM). On the other hand, the [1,2,4]triazolo[3,4-*a*]phthalazin-6(5*H*)-one derivative **VIIa** showed higher cytotoxic activity (3.91 μM) against HepG-2 than compound **16** (6.29 μM). Regarding the Topo II inhibitory activity, the [1,2,4]triazolo[3,4-*a*]phthalazin-6(5*H*)-one derivative **VIIa** showed higher inhibitory activity (7.45 μM) than compound **16** (15.16 μM). For DNA intercalation, compound **16** showed higher intercalation activity (10.25 μM) than **VIIa** (48.30 μM). These findings revealed that the current [1,2,4]triazolo[4,3-*c*]quinazoline derivatives have high binding affinity against DNA with less activity against Topo II when compared with the ([1,2,4]triazolo[3,4-*a*]phthalazin-6(5*H*)-one derivatives. In addition, the HCT-116 cell line are more sensitive towards [1,2,4]triazolo[4,3-*c*]quinazoline derivatives than ([1,2,4]triazolo[3,4-*a*]phthalazin-6(5*H*)-one derivatives. The sensitivity is inversed regarding to HepG-2.

#### 2.2.5. Topo II-mediated DNA cleavage assay

The Topo II inhibitors may act through two pathways. Firstly, it can stabilize the Topo II-DNA covalent complexes leading to the formation of linear DNA (Topo II poisons). Secondly, it can block the catalytic site of Topo II (Topo II catalytic inhibitors) [30–32]. To detect the proposed mode of action, the Topo II-mediated DNA cleavage assay was performed for the most active member **16** against Topo II α and supercoiled pBR322 DNA.

**Fig 3:**
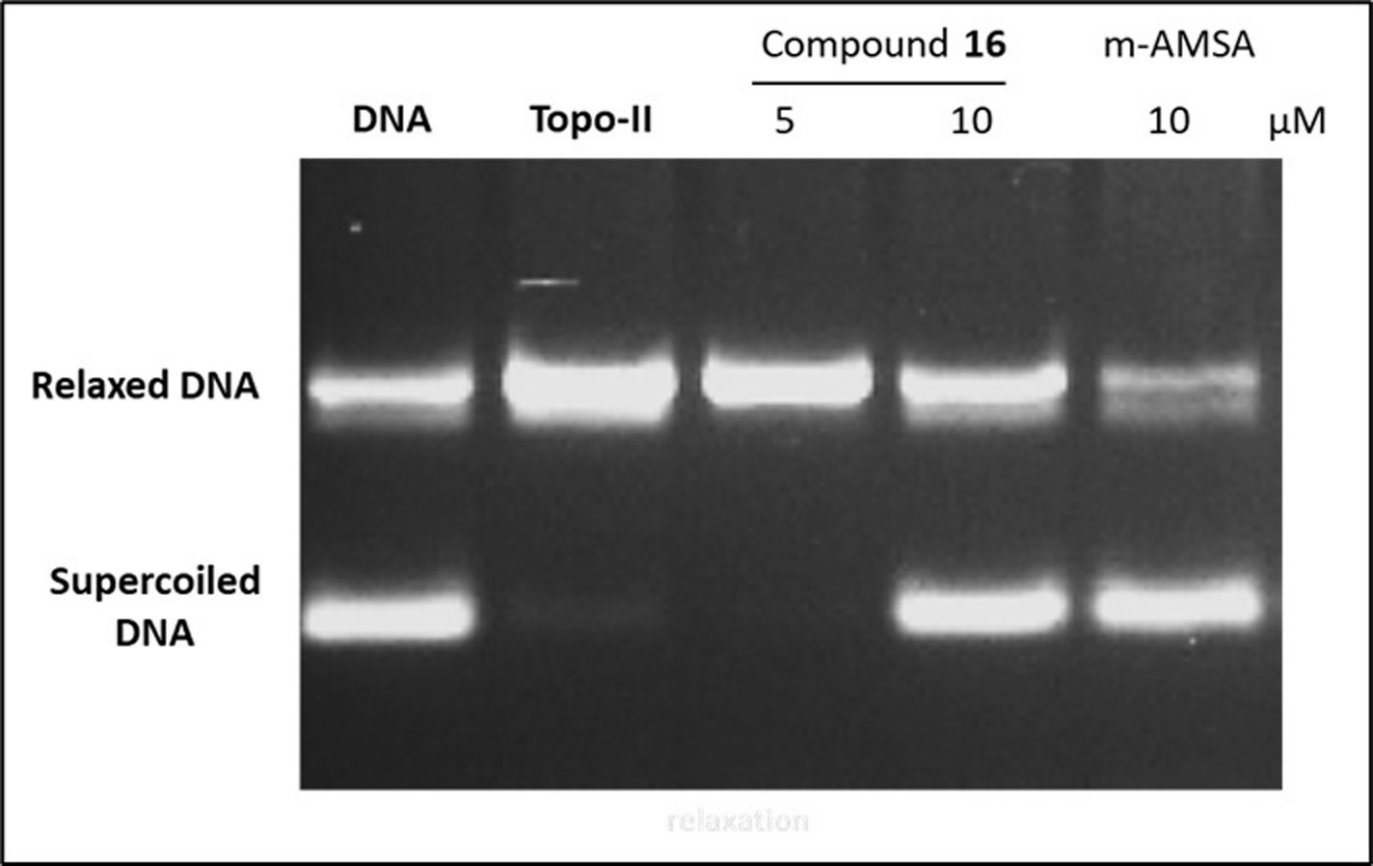
Effect of compound 16 and m-AMSA on relaxation of supercoiled pBS-SK (+) DNA by human recombinant Topo II. Supercoiled DNA was incubated with human recombinant Topo II in the absence (Topo-II) or presence of compound **16** at 5 and 10 μM concentrations.

As shown in **Fig 3**, compound **16** induced relaxation for the DNA at a concentration of 5 μM. In contrast, it blocked the formation of relaxed DNA at 10 μM. In all concentrations, the linear DNA was not formed. This revealed that compound **16** acts as a Topo II catalytic inhibitor at a high concentration (10 μM) and may serve as Topo II poison at low concentration (5 μM). These results are consistence with the previously reported findings by Atwal *et al* [33]. It was reported that anthracyclines (doxorubicin and epirubicin) and another intercalating Topo II poison (mitoxantrone) can stabilize Topo II-DNA covalent complexes at higher concentrations. On the other hand, they suppress the formation of Topo II-DNA covalent complexes, thus behaving as Topo II poisons at low concentration and inhibitors at high concentration [33].

#### 2.2.6. Cell cycle analysis

**Fig 4:**
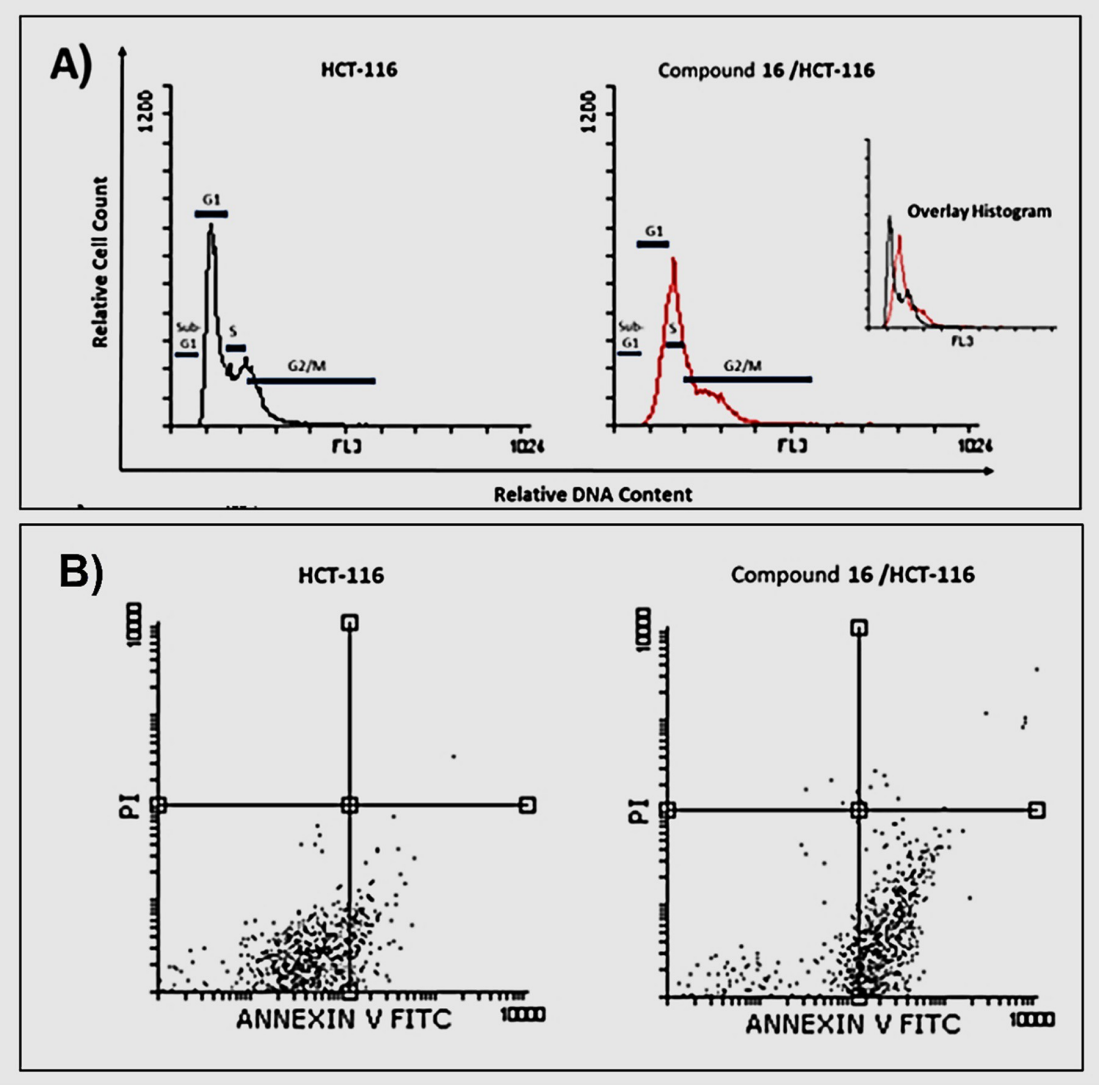
**A)** Flow cytometric analysis of cell cycle phases post the compound **16** treatment. **B)** Flow cytometric analysis of apoptosis in HCT-116 cells exposed to compound **16**.

The balance between cell proliferation and death must be maintained to maintain tissue homeostasis [34–37]. To get an additional relationship between the cell cycle and apoptosis, the effect of compound **16,** the most active candidate, on cell cycle distribution and apoptosis induction in HCT-116 cells was studied to gain a better understanding of how it inhibits cancer cell proliferation. Therefore, the HCT-116 cell line was treated with compound **16** at a concentration of 2.44 μM (the IC_50_ value of compound **16** against the HCT-116 cell) for 48 h then, the cells were harvested, stained with propidium iodide, and analyzed for cell distribution during the various phases of the cell cycle using Flowing software. The flow cytometry data revealed that the percentage of HCT-116 cells decreased at the G1 phase and increased at the sub-G1, S, and G2/M phases. In detail, for the G1 phase, it decreased from 53.75% to 19.05%. On the other hand. In the S phase, it increased from 20.08% to 42.76%. For the G2/M phase, it increased from 25.10% to 36.89%. These results indicate that compound **16** could arrest the cell growth at S and G2/M phases (**Table 2** and **Fig 4A)**. These findings were corroborated by published studies showing that Topo II inhibitors can stop cell development in the G2/M [38]. Since catalytic topo II inhibitors prevent DNA double strand breaks, they also cause cell cycle delay at the G2/M phases by interfering chromosome condensation and segregation during mitosis [39]. In addition, during S phase, topo II is required to resolve topologically linked supercoiled DNA strands while DNA replication forks proceed. Inhibition of enzyme function at this time by a catalytic inhibitor of topoisomerase II has been reported to delay cell cycle progression during the S phase and cause cells to arrest at the G2 phase, delaying entry into mitosis [40].

**Table 2:** Effect of compound 16 on cell cycle progression in HCT-116 cells after 48h treatment.

Sample	Cell cycle distribution (%) ^a^			
	%Sub-G1	%G1	%S	% G2/M
**HCT-116**	1.07 ± 0.04	53.75 ± 2.75	20.08 ± 1.12	25.10 ± 2.02
**Compound 16 / HCT-116**	1.30 ± 0.18	19.05 ± 1.38***	42.76 ± 2.48***	36.89 ± 1.81*

^**a**^ Values are given as mean ± SEM of three independent experiments.

*p < 0.05,

***p < 0.001.

#### 2.2.7. Apoptosis analysis

The Annexin V and PI double staining experiment was used to further investigate the apoptotic effect of compound **16** in HCT-116 cells. In this process, HCT-116 cells were treated with compound **16** at a concentration of 2.44 μM and incubated for 48 h. The apoptotic effect of compound **16** on HCT-116 cells was about nine times greater than that of untreated HCT-116 cells. In comparison to control cells (5.62%), compound **16** caused 48.82% programmed cell death (early apoptosis = 46.77% & late apoptosis = 2.05%) (**Table 3 and Fig 4B**).

**Table 3:** Effect of compound 16 on stages of the cell death process in HCT-116 cells after 48 h treatment.

Sample	Viable ^a^ (Left Bottom)	Apoptosis ^a^		Necrosis ^a^ (Left Top)
		Early (Right Bottom)	Late (Right Top)	
**HCT-116**	94.24 ± 0.90	5.41 ± 0.96	0.21 ± 0.07	0.14 ± 0.03
**Compound 16 / HCT-116**	50.28 ± 2.10	46.77 ± 0.83****	2.05 ± 0.92	0.90 ± 0.36

^a^ Values are given as mean ± SEM of three independent experiments.

****p < 0.0001.

#### 2.2.8. Effects on apoptotic markers (BAX and Bcl-2)

B-cell lymphoma protein 2 (Bcl-2) is a member of the B-cell lymphoma protein family that plays a key role in tumor formation and suppression of the intrinsic apoptotic pathway [41]. The anti-apoptotic Bcl-2 protein suppresses apoptosis, whereas BAX promotes it (proapoptotic). As a result, the balance between these two contradictory proteins controls cell fate [42].

**Fig 5:**
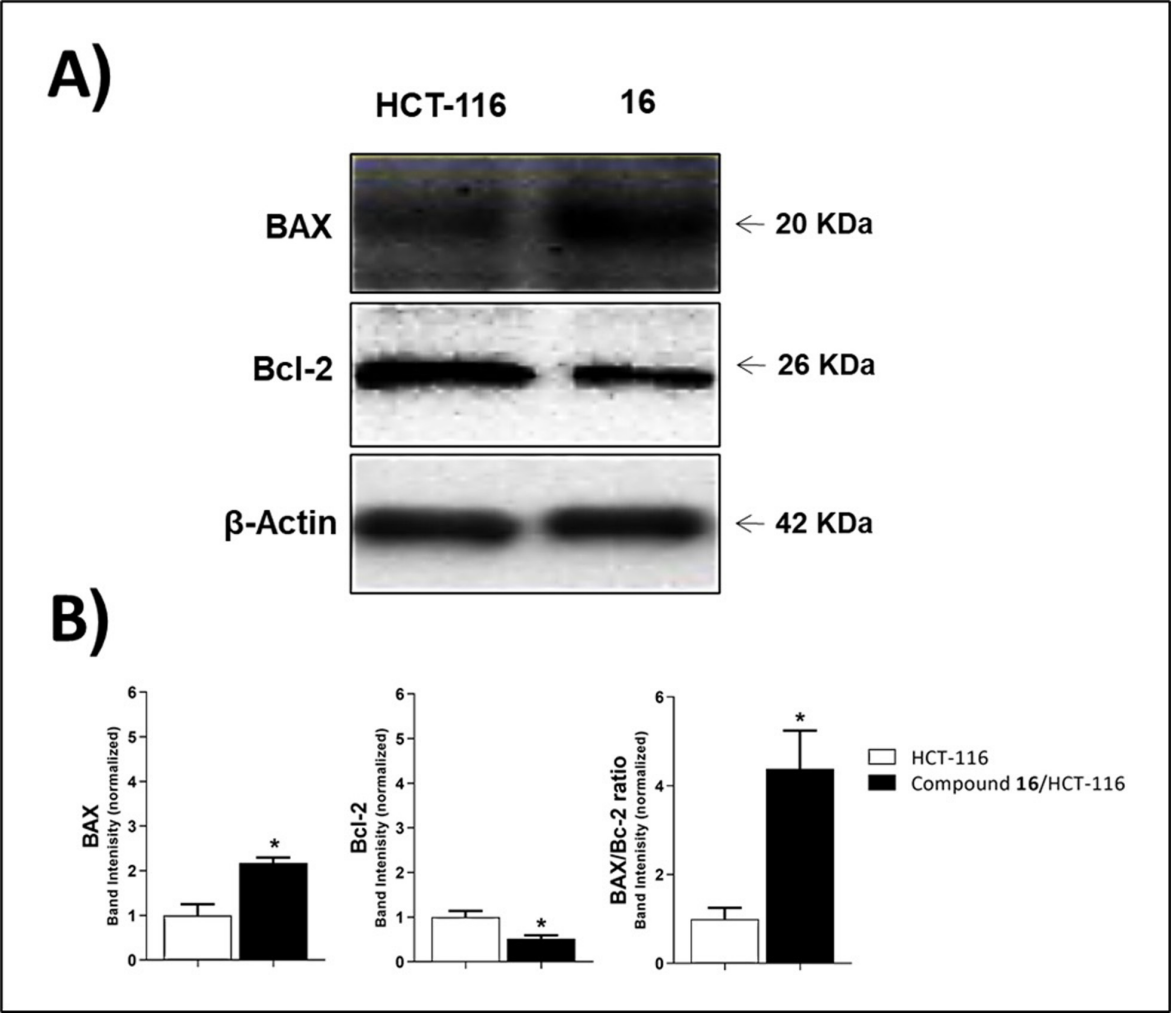
The immunoblotting of BAX and Bcl-2 (Normalized to β-actin).

In this investigation, HCT-116 cells were treated with 2.44 μM (IC_50_ value of compound **16**) for 48 hours to see its effect on Bcl-2 and BAX levels. In comparison to the control, compound **16** elevated the level of the proapoptotic protein (BAX) by 2.18 times. Furthermore, compound **16** significantly reduced the anti-apoptotic protein Bcl-2 levels by 1.9-fold when compared to the control. Furthermore, compound **16** enhanced the BAX/Bcl-2 ratio by 4.38-fold in comparison to control cells. These findings demonstrated that the BAX/Bcl-2 ratio may promote the apoptotic process (**Table 4** and **Fig 5**).

**Table 4:** Effect of compound 16 on the levels of BAX, and Bcl-2 proteins expression in HCT-116 cells treated for48 h.

Sample	Protein expression (normalized to β-actin) ^a^		
	BAX	Bcl-2	BAX/Bcl-2 ratio
**HCT-116**	1.00 ± 0.25	1.00 ± 0.14	1.00 ± 0.25
**Compound 16 / HCT-116**	2.18 ± 0.12*	0.52 ± 0.08*	4.38 ± 0.86*

^**a**^ Values are given as mean ± SEM of three independent experiments.

*p < 0.05

#### 2.2.9. *In vitro* cytotoxicity against normal cell line

The most active candidate **16** was tested against normal cell lines (Vero cells) using. Although Vero cell are not human cell, it was reported that Vero cells can be used as normal cells to validate the toxicity of the synthesized compounds [43]. In this work, compound **16** showed an IC_50_ value of 63.36 μM against the tested normal cells. These results indicated that the tested compound possesses high selectivity against tumor cells. comparing its activity against normal Vero cells, its activity was 10-fold and 26-fold against HepG2 and HCT-116 cells, respectively. The prototype VEGFR-2 was reported to have IC_50_ value of 24.34 μM against Vero cells [44]. This indicates that the toxicity of compound 16 is less than that of sorafenib by about 2.5-fold.

### 2.3. *In silico* studies

#### 2.3.1. Docking studies

Docking studies were carried out for the target compounds against the DNA-topoisomerase II complex (PDB ID: 4G0U). The co-crystallized ligand (amsacrine) was used as a reference molecule. The reported key binding site of the DNA-topoisomerase II complex involves Asp479, Arg503, Gln778, Met782, Cyt8, Ade12, Thy9, Gua13, and, Cyt11 [45].

**Fig 6:**
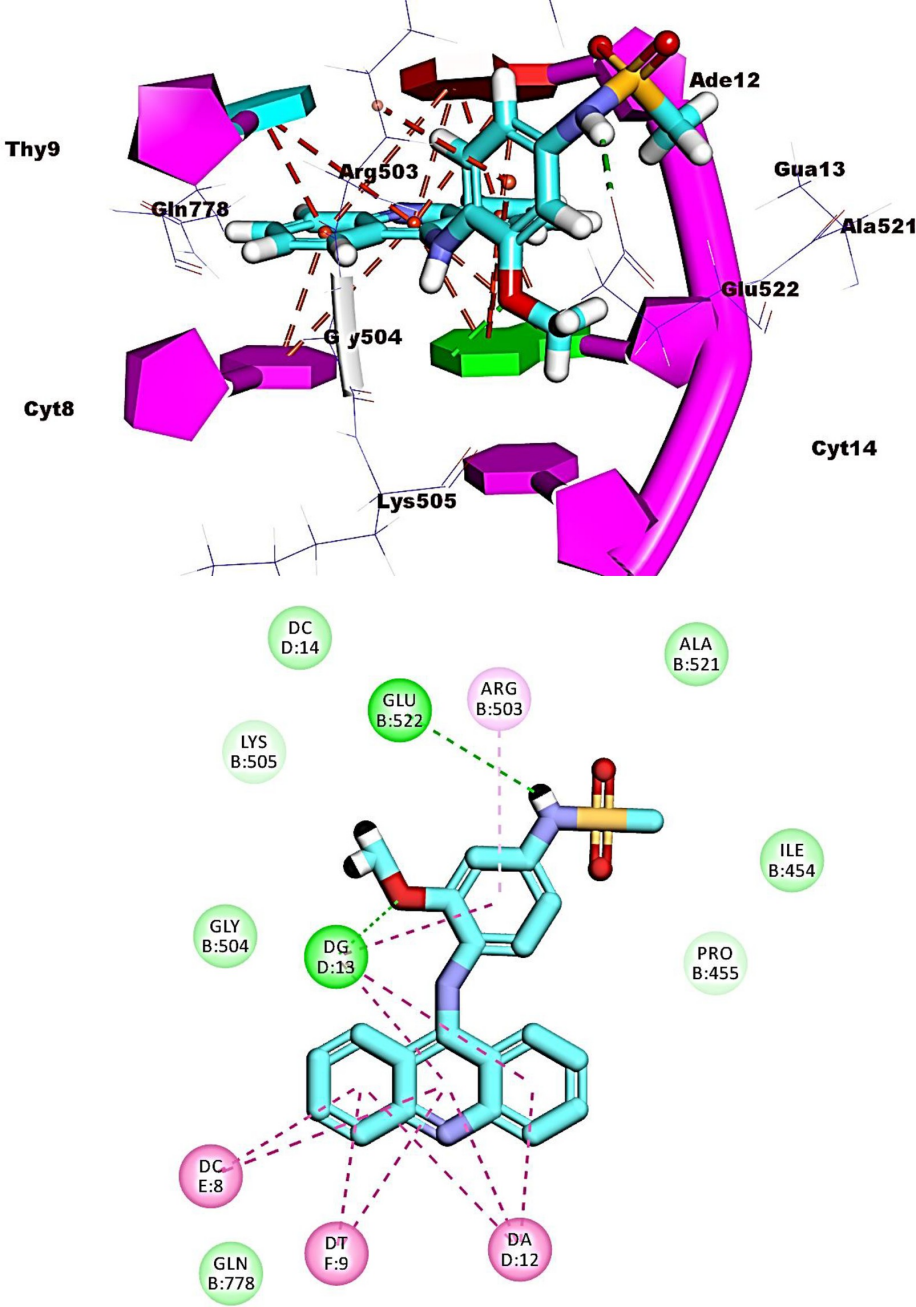
Binding of amsacrine with DNA-Topo II complex.

For amsacrine, its planar aromatic (acridine) moiety was inserted between the nucleotide of the DNA forming nine pi-pi interactions with Thy9, Cyt8, Ade12, and Gua13. The groove binding side chain (*N*-(4-amino-3-methoxyphenyl)methanesulfonamide) was oriented into the minor groove of DNA forming two hydrogen bonds with Glu522 and Gua13. Also, it formed two hydrophobic interactions with Arg503 and Gua13 (**Fig 6**).

The results of the docking study revealed that the docked compounds have a similar binding mode to the reference ligand amsacrine. The binding energies of the docked compounds were summarized in **Table 5**.

**Table 5:** The docking binding free energies (ΔG) of the synthesized compounds with DNA-topoisomerase II complex.

Comp.	ΔG (kcal/mol)	Comp.	ΔG (kcal/mol)
**11a**	- 48.1944	**15a**	-47.8213
**11b**	-48.222	**15b**	-49.3717
**12a**	-46.6562	**16**	-34.8376
**12b**	-51.4022	**17**	-42.6689
**13a**	-43.1622	**18**	-35.8691
**13b**	-43.589	**19**	-41.0473
**14a**	-44.7025	**20**	-36.8204
**14b**	-45.2039	**Amsacrine**	-56.3034

All the docked molecules were inserted between the adjacent nucleotides at the active site. These compounds were stabilized in the active site through different interactions. The planar system was involved in hydrophobic stacking with the base pairs of different nucleotides (Thy9, Cyt8, Gua13, and Ade12). The hydrophilic moieties formed several hydrogen bonds with aforementioned nucleotides. Other stabilizing interactions was achieved by the aid of the side chains (groove binding side chains). These moieties were oriented into the minor groove of the DNA forming hydrogen and hydrophobic bonds. The binding modes of the docked molecules were consistence with the reported findings [18–20].

**Fig 7:**
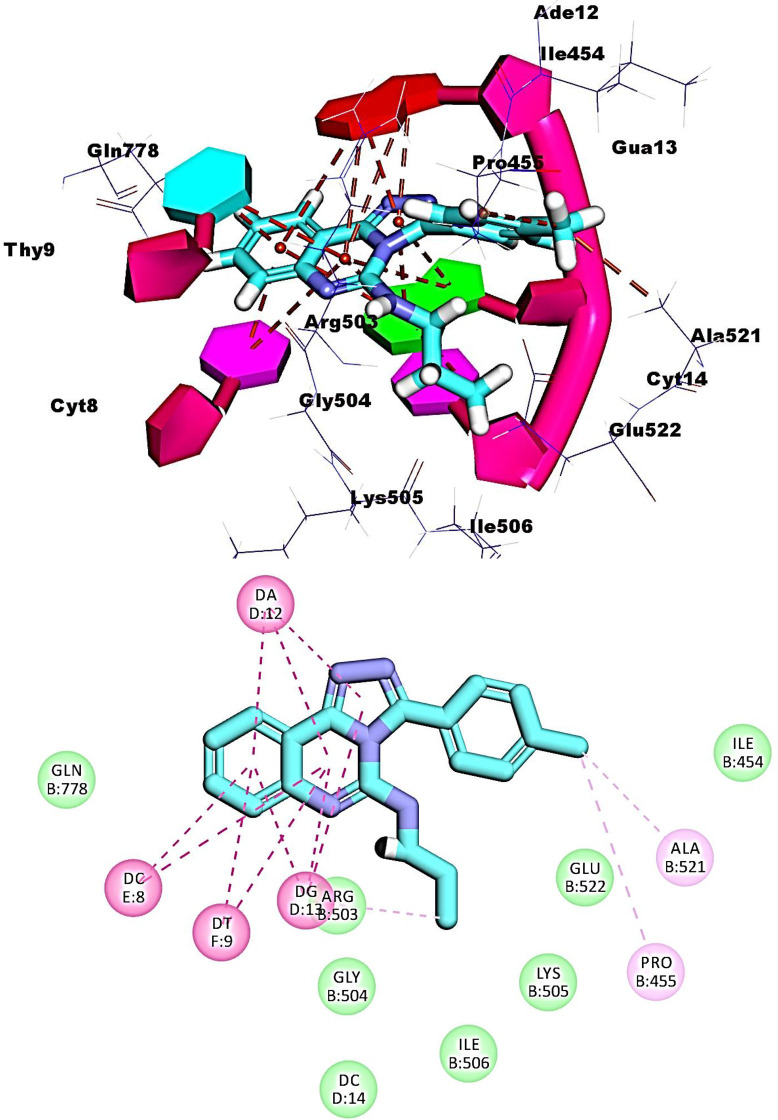
Binding of compound 11a with DNA-Topo II complex.

Regarding compound **11a,** The planar aromatic system ([1,2,4]triazolo[4,3-c]quinazoline) was inserted between the adjacent nucleotides of DNA forming ten hydrophobic stacking with, Thy9, Cyt8, Gua13, and Ade12. The terminal toluene moiety bound the minor groove of DNA forming two hydrophobic interactions with Ala521and Pro455. Moreover, the propane moiety was oriented into the minor groove with the formation of one hydrophobic interaction with Gua13 (**Fig 7**).

**Fig 8:**
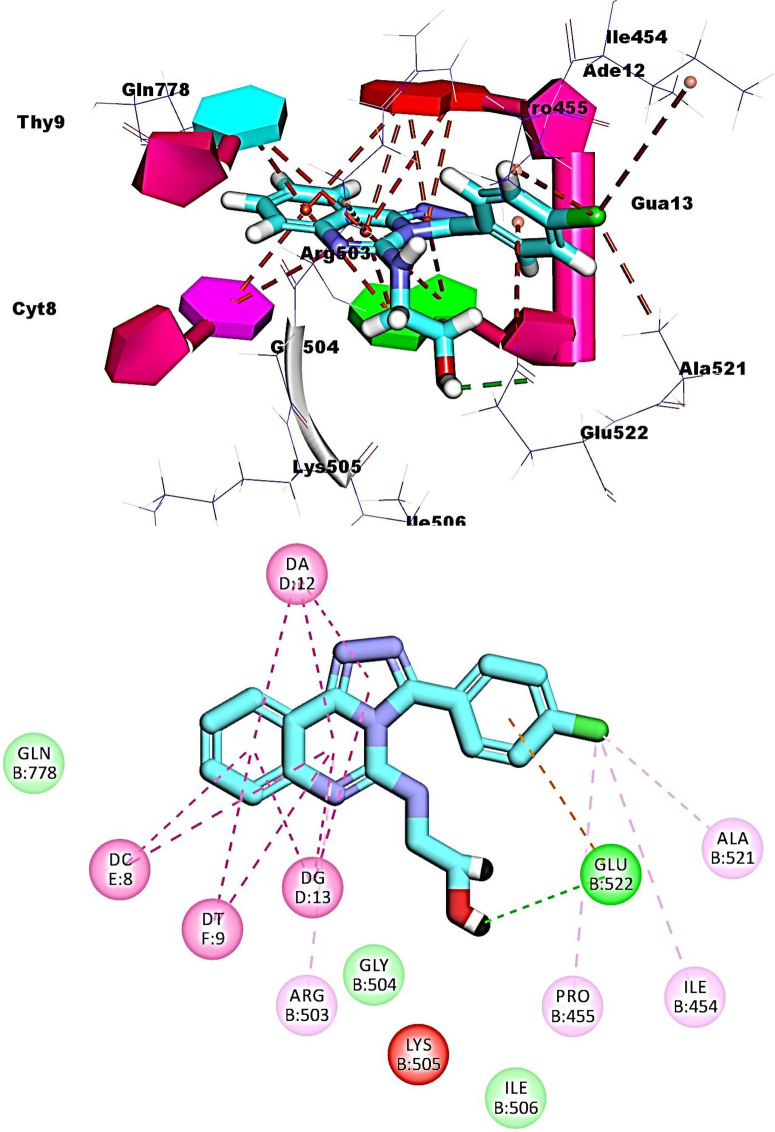
Binding of compound 12b with DNA-Topo II complex.

The ([1,2,4]triazolo[4,3-c]quinazoline moiety) of compound **12b** formed ten pi-pi interactions with Ade12, Gua13, Cyt8 and Thy9. Both chlorobenzene and ethanolamine moieties were oriented into the minor groove of DNA forming four hydrophobic interactions with Ala521, Ile454, Pro455, and Glu522. The OH group formed one hydrogen bond with Glu522 (**Fig 8**).

**Fig 9:**
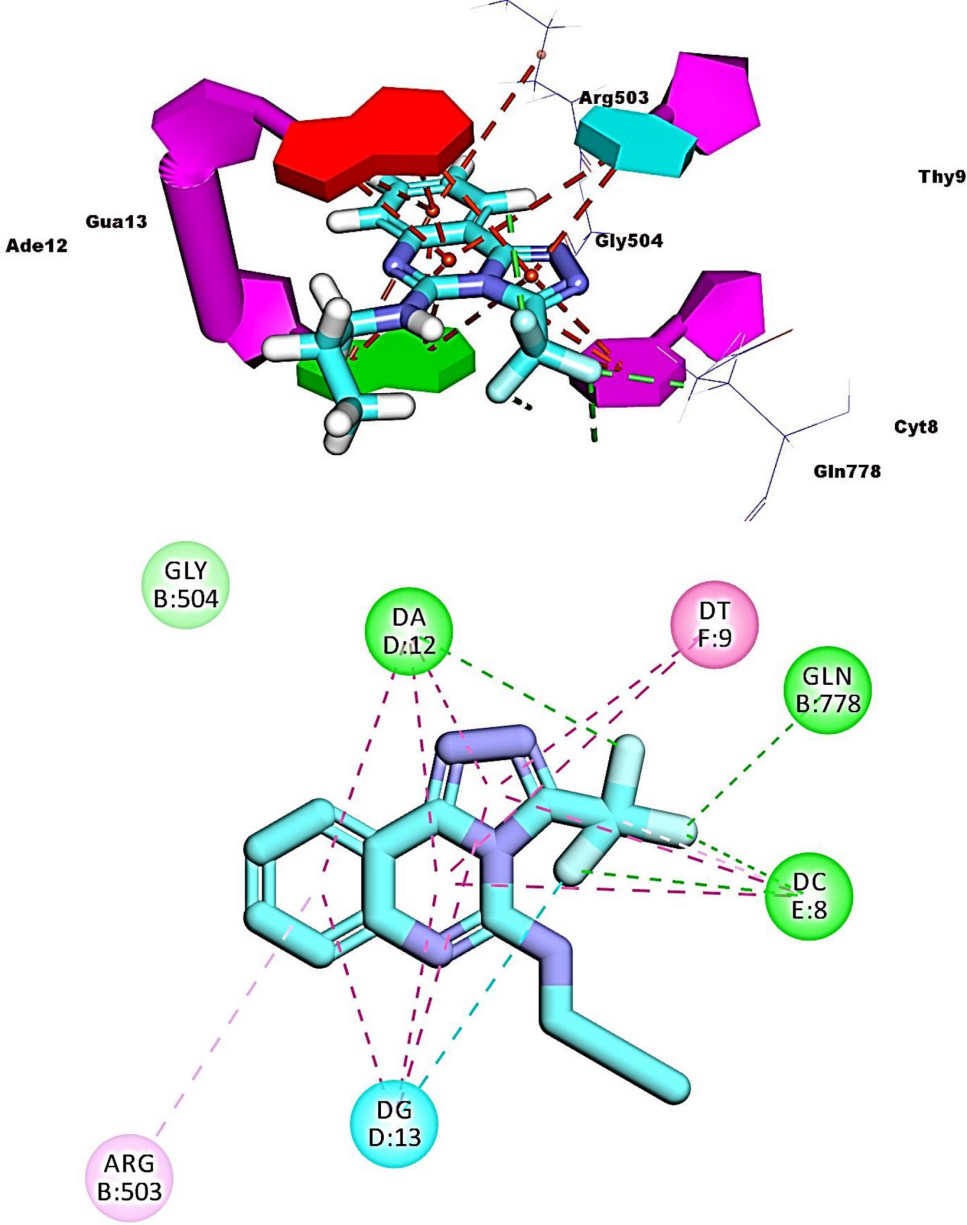
Binding of compound 16 with DNA-Topo II complex.

**Fig 10:**
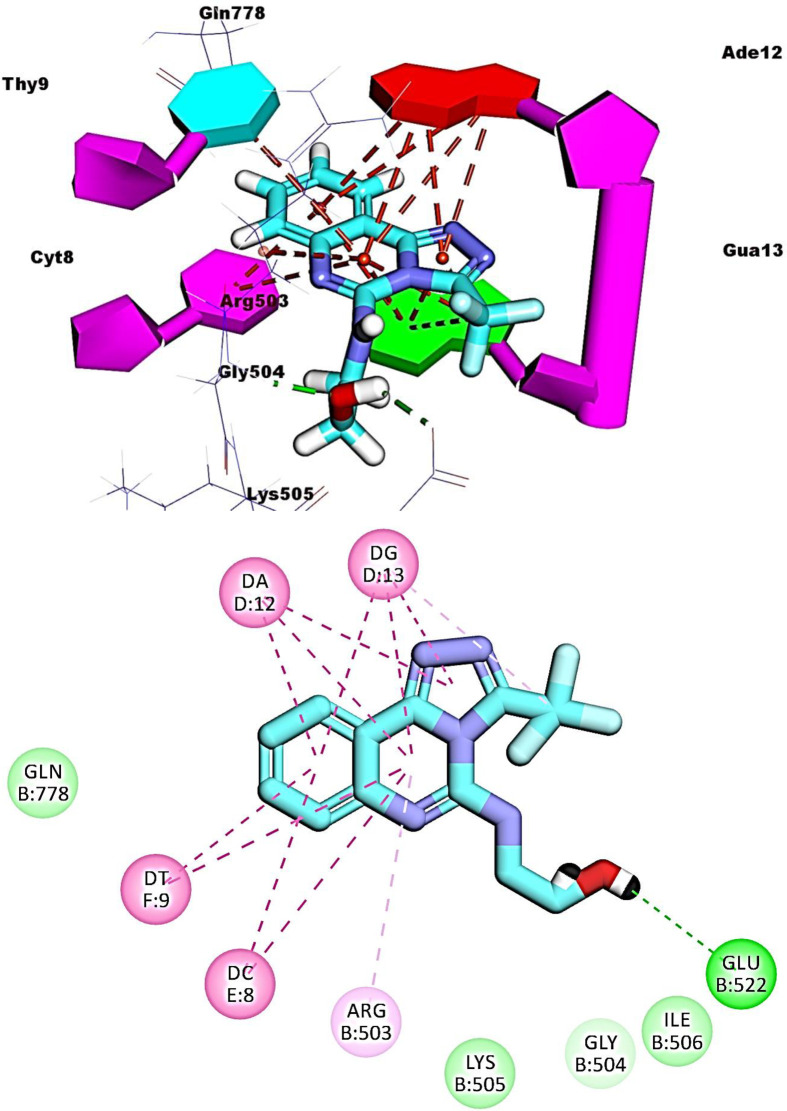
Binding of compound 17 with DNA-Topo II complex.

**Fig 11:**
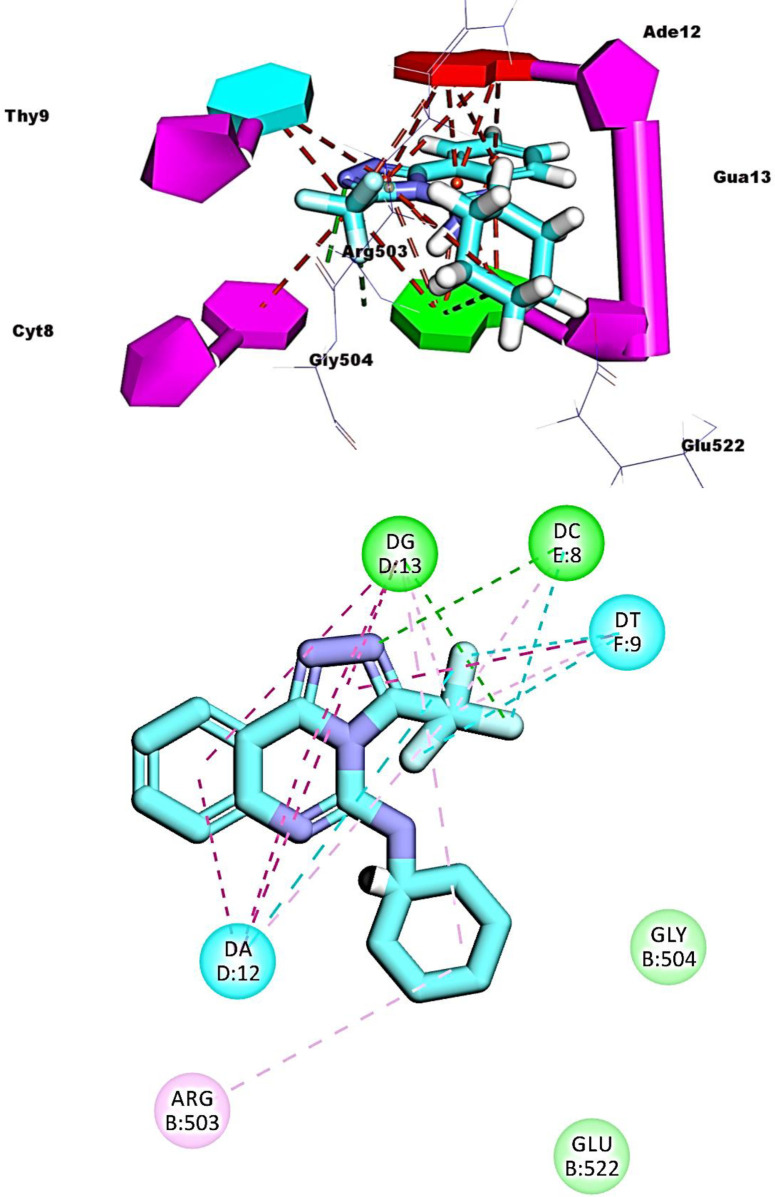
Binding of compound 19 with DNA-Topo II complex.

Compounds **16**, **17**, and **19** exhibited similar fitting patterns against the active site. The planar aromatic systems ([1,2,4]triazolo[4,3-c]quinazoline) formed ten, eleven, and seven pi-pi interactions, respectively. The side chains of these compounds were oriented towards the minor groove of DNA. The trifluoromethyl moiety of compound **16** formed four hydrogen bonds with Ade12, Cyt8, and Gln778. The ethanolamine moiety of compound **17** formed one hydrogen bond with Glu522. The trifluoromethyl moiety of compound **19** formed two hydrogen bonds with Cyt8 and Gua13 (**Figs 9–11**).

#### 2.3.2. *In silico* toxicity studies

The toxicity profile of the tested compounds was investigated against seven constructed models using Discovery studio software. Amsacrine was utilized as a reference drug [46–54].

Most compounds showed *in silico* low adverse effects and toxicity against the tested models. In general, all compounds were predicted to be non-carcinogenic against the FDA rodent carcinogenicity mouse model.

For carcinogenic potency TD_50_ rat model, compounds **11a**, **b**, **12a**, **b**, **16**, **17**, **18**, and **20** showed TD_50_ values ranging from 4.274 to 43.706 g/kg body weight, which are higher than amsacrine (3.569 g/kg body weight). Regarding the rat maximum tolerated dose model, all candidates except **13a**, **15a**, **18**, and **20** demonstrated maximum tolerated dose with values ranging from 0.070 to 0.241 g/kg body weight which are higher than that of amsacrine **(**0.068 g/kg body weight). For the rat oral LD_50_ model, all compounds revealed oral LD_50_ values lower than that of amsacrine (3.005 g/kg body weight). Additionally, all compounds were anticipated to be non-toxic against the developmental toxicity potential model. For rat chronic LOAEL model, compounds **11a**, **12a**, **13a**, **14a**, and **15a** displayed LOAEL values ranging from 0.029 to 0.044 g/kg body weight. These values are higher than amsacrine (0.025 g/kg body weight). Finally, all the tested compounds were predicted to be non-irritant against the skin irritancy model **(Electronic Supplementary Material S4.1 in S1 Appendix)**.

#### 2.3.3. Molecular dynamic simulation

**Fig 12:**
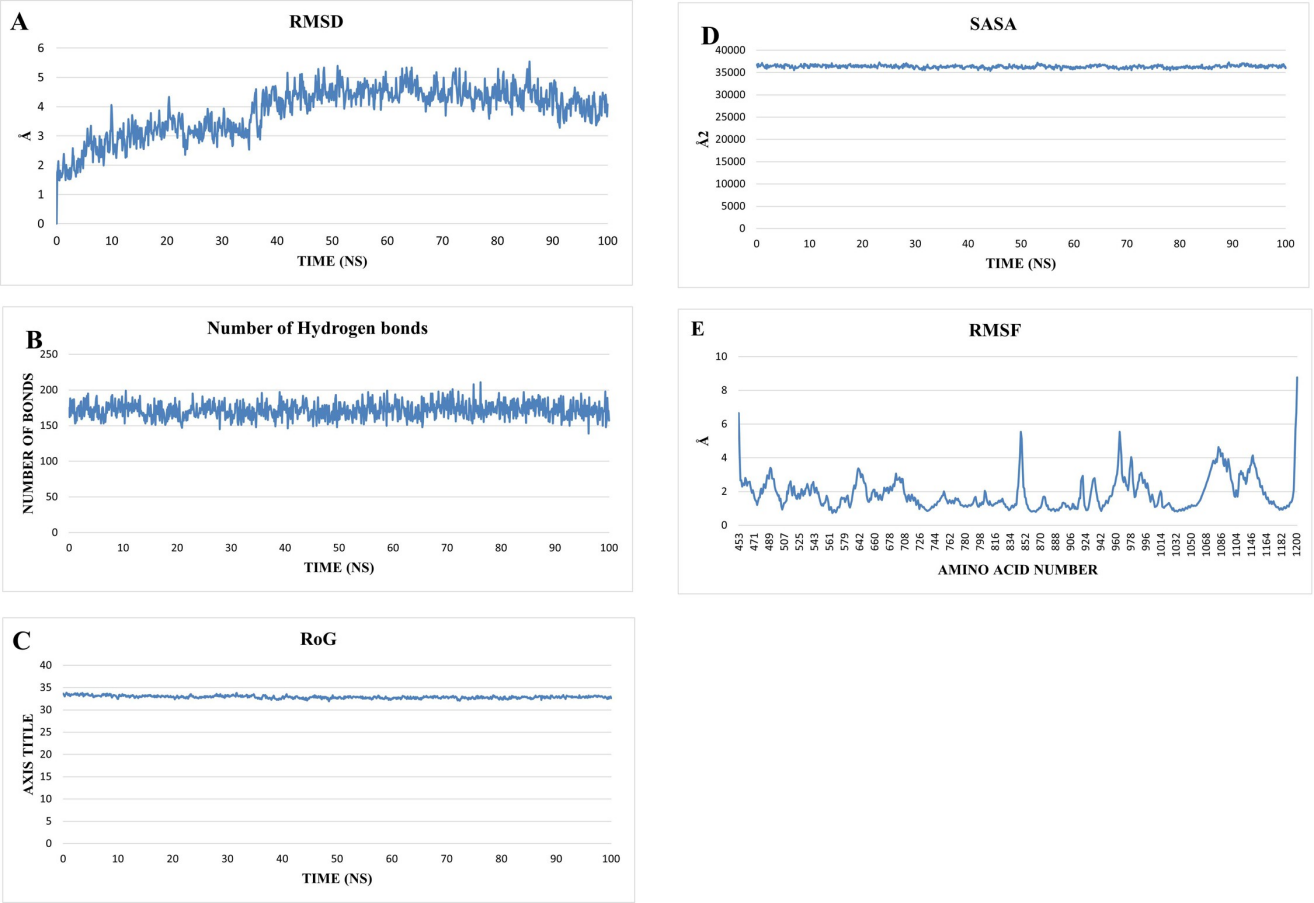
The different MD analyses results done on the protein including **A**) RMSD, **B**) H-bonds, **C**) RoG, **D**) SASA, and **E**) RMSF.

To analyze the trajectory obtained from the MD simulation, Root Mean Square Deviation (RMSD), Root Mean Square Fluctuation (RMSF), Solvent Accessible Surface Area (SASA), Radius of Gyration (RoG), and the change in the number of H-bonds were calculated using TCL scripts in VMD (**Fig 12**). **Fig 12A** showed the RMSD of the protein backbone along the trajectory. The protein showed a short stable fluctuation from 5 ns to 35 ns around 3.08 Å before rising to an average of 4.36 Å in the last 65 ns. This indicates that the system has stabilized. **Fig 12B–12D** give information about the folding and unfolding of the protein. The number of internal H-bonds, RoG, and SASA, show a stable trend during the whole simulation with an average of approximately 172 bonds, 32.8 Å, and 36335 Å^2^, respectively. **Fig 12E** showed the C_α_ RMSF values which give an indication on the average fluctuation of each amino acid during the simulation. Some regions of the protein showed high fluctuations such as both the C and N terminals and loops within the protein as they are not included in a secondary structure.

**Fig 13:**
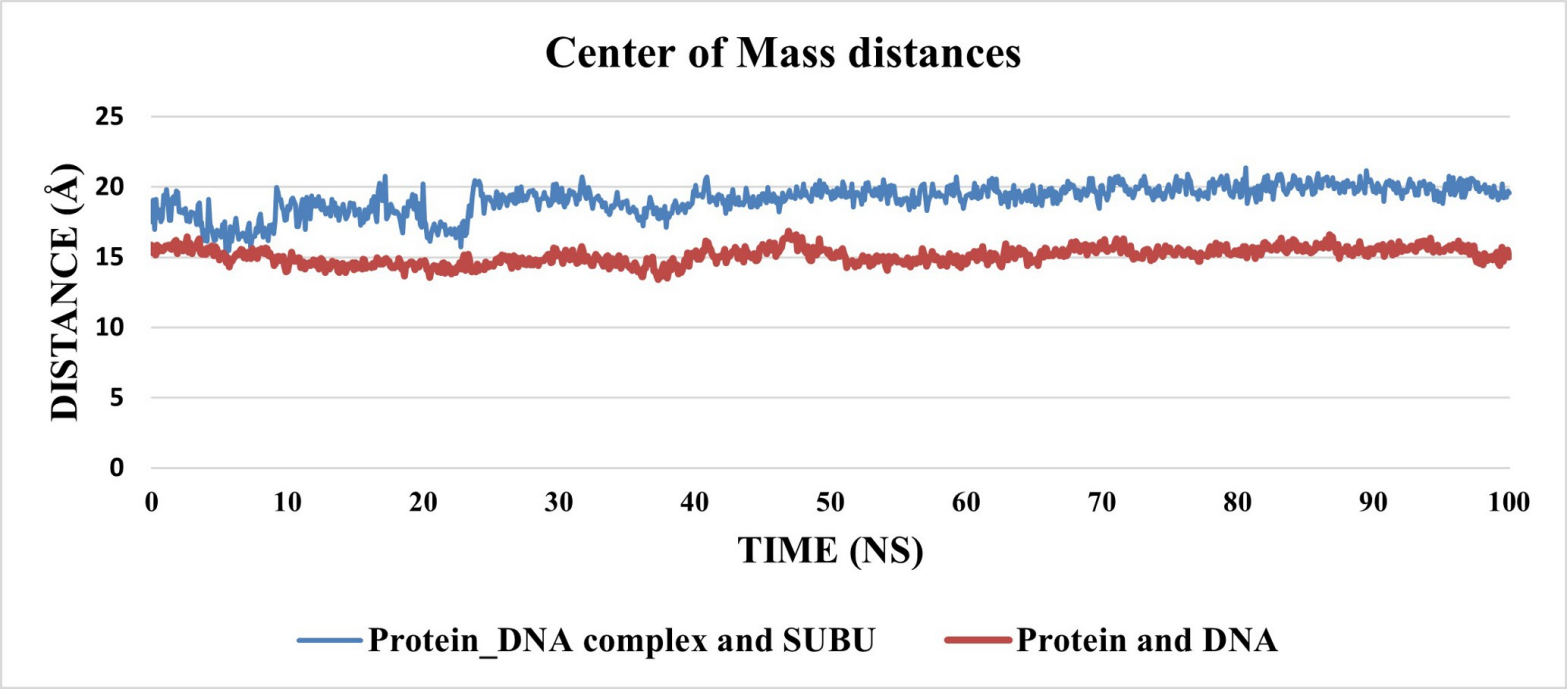
The center of mass distances between the protein-DNA complex and the ligand (Blue line), and the protein and DNA (red line).

**Fig 14:**
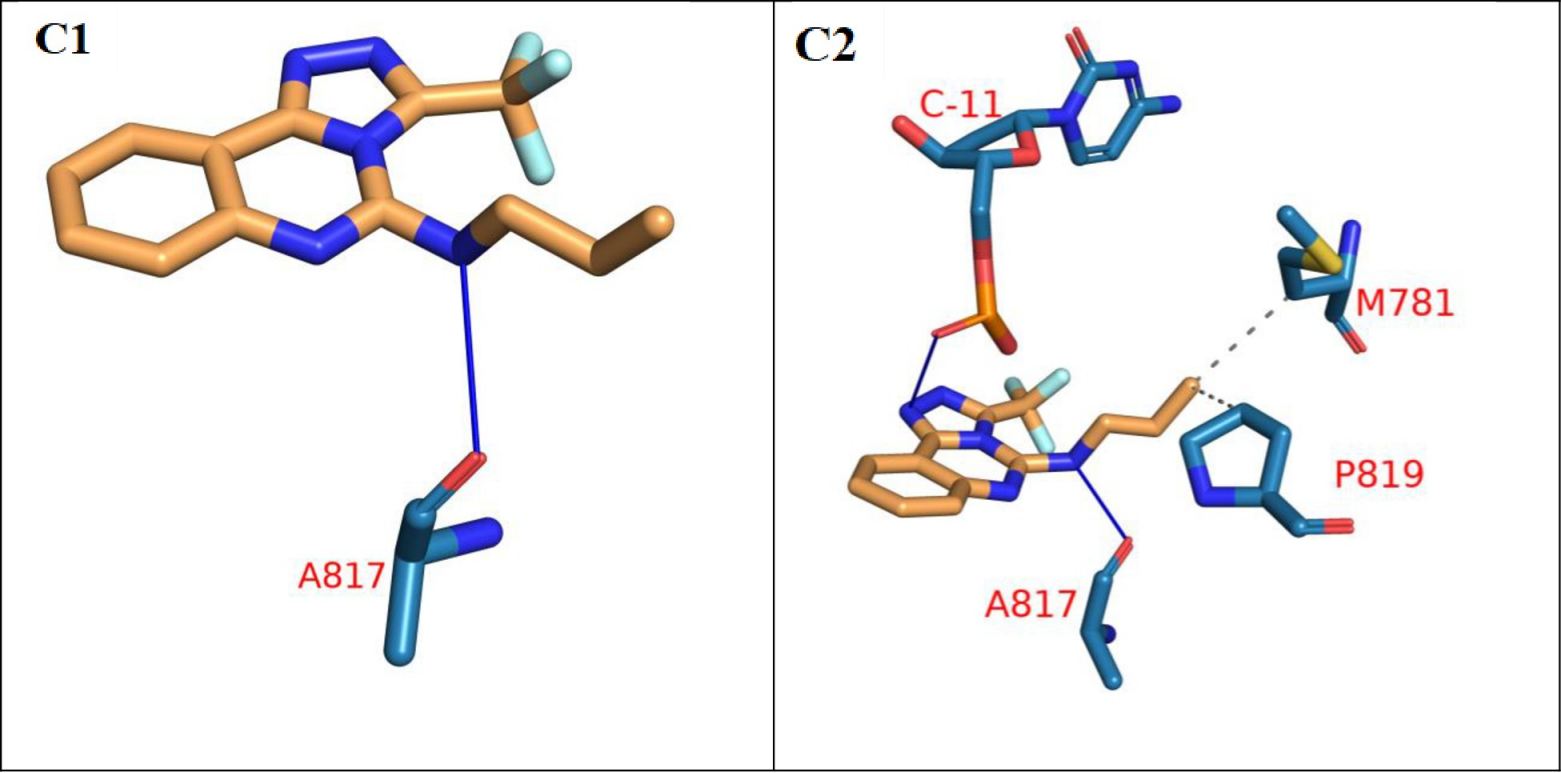
3D interactions between the ligand and the protein_DNA complex in the first (C1) and second (C2) clusters. Ligand is in orange sticks representation. Amino acid and nitrogenous bases are in blue sticks representations. H-bond: Solid blue line, Hydrophobic interactions: dashed grey lines. Names of amino acids and nitrogenous bases are in red letters.

Since this system includes DNA molecule and ligand, the center of mass (COM) distance between the ligand and the protein and the distance between the DNA and the protein were measured to see whether either of them moves away from the protein (**Fig 13**). The distance between the COM of the protein and DNA (red line) showed a stable trend along the trajectory with an average of 15.1 Å. On the other hand, the COM distance between the ligand and the protein showed stability starting from 25 ns with an average of 19.5 Å. The trajectory was clustered using TTClust library and to obtain a representative frame for each cluster. Two clusters were obtained from the trajectory and were used with PLIP webserver to get the interacting amino acids and the types of interactions (**Table 6**). Only two types of interactions are present: H-bond (three interactions) and hydrophobic interactions (two interactions). **Fig 14** showed the 3D interactions between the ligand and the protein_DNA complex in the two cluster representatives.

**Table 6:** The number of clusters, number of interactions, and their types between the ligand and protein_DNA complex.

Cluster number	Number of hydrophobic interactions	Amino acids in receptor	Number of hydrogen bonds	Amino acids in receptor
**C1**	0	None	1	A817
**C2**	2	M781—P819	2	**C-11**—A817

The second cluster shows an interaction between the Cytosine nitrogenous base (underlined and bold) and the ligand.

**Fig 15:**
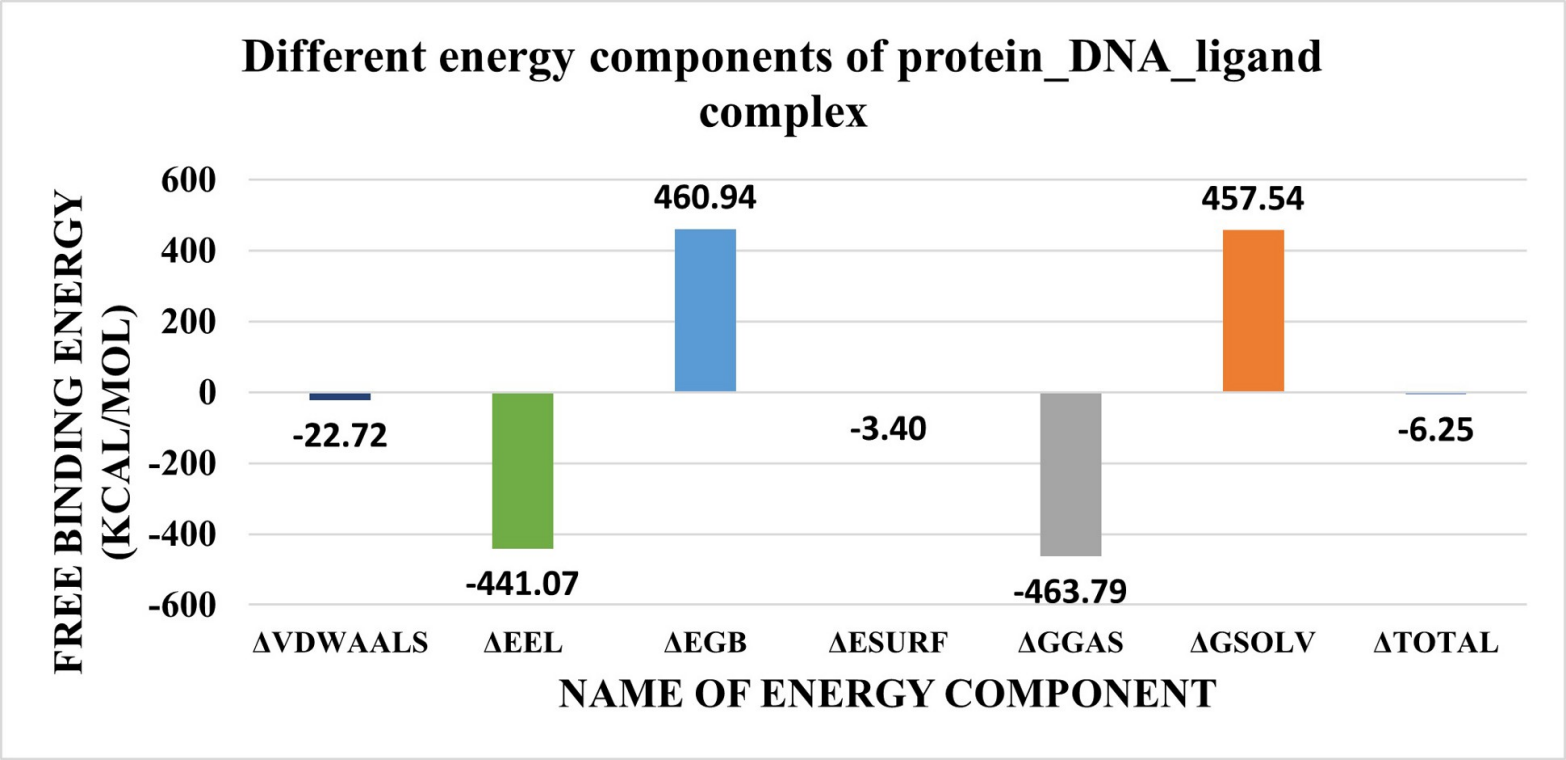
The contributions from the different energy components of MM-GBSA.

Furthermore, Molecular Mechanics Generalized Born Surface Area (MM-GBSA) was utilized to calculate the binding free energy between the ligand and the protein_DNA complex. **Fig 15** showed the contribution of different components. The most components contributing to the binding of the ligand to the protein_DNA complex are the electrostatics (-441.07 Kcal/mol) and the solvation energy (+460.94 Kcal/mol) with a total binding energy of -6.25 Kcal/mol.

**Fig 16:**
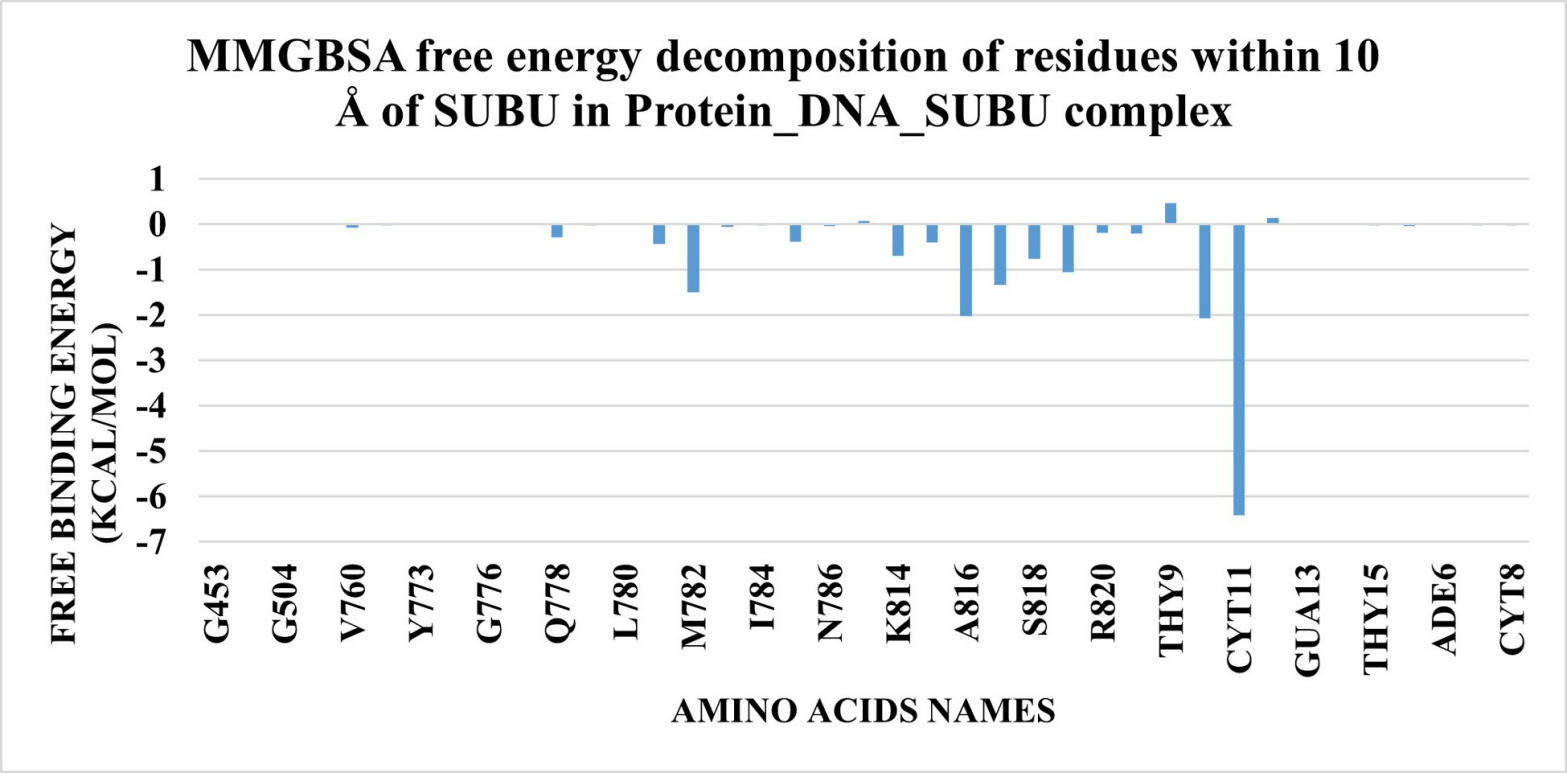
The contributions of amino acids around 10 Å of the ligand to the binding free energy.

To know which amino acid contributed strongly to the interaction, decomposition was performed. **Fig 16** showed the contribution of amino acids within 10 Å around the ligand. Two nitrogenous bases (Cytosine 11 and Guanine 10) show the strongest two interactions with the ligand -6.40 Kcal/mol and -2.07 Kcal/mol, respectively. Other interactions were formed between the amino acids of the protein with amino acids A816, M782, A817, and P819 showing the highest binding affinity of -2.01 Kcal/mol, -1.5 Kcal/mol, -1.33 Kcal/mol, and -1.05 Kcal/mol, respectively.

## 3. Conclusion

To conclude, fifteen new [1,2,4]triazolo[4,3-*c*]quinazoline derivatives were designed, synthesized, and biologically examined as antiproliferative Topo II inhibitors. The antiproliferative assay of the synthesized members against HepG2 and HCT-116 cancer cell lines revealed that compounds **13a**, **14a**, **16**, **17**, **18**, **19,** and **20** showed promising cytotoxicity results with IC_50_ values ranging from 2.44 to 13.02 μM. Further biological examinations were carried out for the most active members including Topo II inhibition and DNA-binding assay. Compounds **16**, **17**, and **18** were the most active Topo II inhibitors with IC_50_ values of 15.16, 17.66, and 18.28 μM, respectively. Also, the same three compounds **16**, **17**, and **18** exhibited significant DNA binding affinity with IC_50_ values of 10.25, 11.09, and 12.54 μM, respectively. The structure-activity relationship revealed that substitution of [1,2,4]triazolo[4,3-*c*]quinazoline with trifluoromethyl moiety is beneficial for cytotoxic activity. Besides, compound **16** arrested the cell cycle of HCT-116 cells at S and G2/M phases. The apoptotic effect of compound **16** (48.82%) was nine times larger than that in control cells (5.62%). Furthermore, compared to control HCT-116 cells, compound **16** boosted the level of BAX by 2.18-fold, downregulated the level of Bcl-2 by 1.9-fold and boosted the BAX/Bcl-2 ratio by 4.38-fold. The results of docking studies revealed that the docked compounds have a similar binding mode to that of doxorubicin with binding energies ranging from—29.41 to—46.03 kcal/mol. Finally, the synthesized candidates had good toxicity profiles and may serve as lead compounds for the discovery of new anticancer agents.

## 4. Experimental

### 4.1. Chemistry

#### 4.1.1. General

Reagents, solvents, and apparatus used in chemical synthesis were shown in **Electronic Supplementary Material S1 in S1 Appendix**. Compounds **2**, **3**, **5**, **6**, **8**, **9**, and **10** were prepared according to the reported procedure [24, 25]. Compound **15b** was previously synthesized by El-Kerdawy *et al*. in a different manner [55].

#### 4.1.2. General procedure for the synthesis of compounds 11_a,b_ -20

Compounds **7a**, **b**, and **10** (1.8 mmol) were dissolved in isopropyl alcohol (20 mL) and then added to appropriate amine (3.6 mmol). The mixture was refluxed at 120°C for 6–10 h. After the end of the reaction (monitored by TLC), the mixture was allowed to cool. Water (200 mL) was added to the reaction mixture and the formed precipitate was collected by filtration and dried. The solid mass was crystallized from a mixture of dichloromethane and methanol 1:1 to afford the corresponding compounds **11a**, **b**-**20.**

*4*.*1*.*2*.*1*. *N-Propyl-3-(p-tolyl)-[1*,*2*,*4]triazolo[4*,*3-c]quinazolin-5-amine 11a*. White powder (yield 77%); mp: 251–253 °C; FT-IR (vmax, cm^-1^): 3258 (NH), 3063 (CH aromatic), 2957 (CH aliphatic), 1548 (C = N); ^1^H NMR (400 MHz, DMSO-d6) δ 8.44 (d, *J* = 7.9 Hz, 1H), 8.35 (dd, *J* = 8.1, 3.9 Hz, 3H), 7.99 (t, *J* = 7.6 Hz, 1H), 7.89–7.77 (m, 2H), 7.35 (d, *J* = 7.9 Hz, 2H), 3.49–3.38 (m, 2H), 2.44 (s, 3H), 1.78 (t, *J* = 7.4 Hz, 2H), 1.02 (t, *J* = 7.4 Hz, 3H); ^13^C NMR (DMSO-d6) δ ppm: 153.8, 149.2, 144.6, 137.4, 135.2, 133.7, 131.5, 129.1, 126.9, 125.5, 122.7, 120.1, 114.6, 40.9, 38.6, 20.4, 11.2. Anal. Calc. for: (C_19_H_19_N_5_) (M.W. = 317).

*4*.*1*.*2*.*2*. *3-(4-Chlorophenyl)-N-propyl-[1*,*2*,*4]triazolo[4*,*3-c]quinazolin-5-amine 11b*. White powder (yield 79%); mp: 267–269 °C; FT-IR (vmax, cm^-1^): 3259 (NH), 3067 (CH aromatic), 2960 (CH aliphatic), 1551 (C = N); ^1^H NMR (400 MHz, DMSO-d6) δ 8.47 (dd, *J* = 11.4, 8.3 Hz, 3H), 8.37 (d, *J* = 8.2 Hz, 1H), 7.95 (t, *J* = 7.6 Hz, 1H), 7.91–7.83 (m, 2H), 7.63 (d, *J* = 8.6 Hz, 2H), 3.42 (q, *J* = 6.7 Hz, 2H), 1.75 (h, *J* = 7.3 Hz, 2H), 1.05 (t, *J* = 7.4 Hz, 3H);^13^C NMR (DMSO-d6) δ ppm: 153.1, 148.1, 144.8, 136.5, 135.4, 132.9, 131.32, 130.7, 128.4, 126.8, 125.6, 122.2, 120.6, 44.9, 39.6, 20.4, 13.2. Anal. Calc. for: (C_18_H_16_ClN_5_) (M.W. = 337).

*4*.*1*.*2*.*3*. *2-((3-(p-Tolyl)-[1*,*2*,*4]triazolo[4*,*3-c]quinazolin-5-yl)amino)ethan-1-ol 12a*. White solid (yield 68%); mp: 251–253 °C; FT-IR (vmax, cm^-1^): 3335 (OH), 3190 (NH), 3095 (CH aromatic), 2925 (CH aliphatic), 1557 (C = N); ^1^H NMR (400 MHz, DMSO-d6) δ 8.55–8.40 (m, 2H), 8.38 (d, *J* = 7.9 Hz, 2H), 7.98 (t, *J* = 7.5 Hz, 1H), 7.86 (t, *J* = 7.7 Hz, 1H), 7.77 (s, 1H), 7.42 (d, *J* = 8.1 Hz, 2H), 4.81 (s, 1H), 3.79 (t, *J* = 5.8 Hz, 2H), 3.58 (t, *J* = 5.8 Hz, 2H), 2.44 (s, 3H); ^13^C NMR (DMSO-d6) δ ppm: 154, 149.3, 144.6, 141.4, 135.3, 132.7, 131.6, 129.2, 126.8, 126.6, 125.7, 125.1, 120.6, 60.9, 46.9, 21.4. Anal. Calc. for: (C_18_H_17_N_5_O) (M.W. = 319).

*4*.*1*.*2*.*4*. *2-((3-(4-Chlorophenyl)-[1*,*2*,*4]triazolo[4*,*3-c]quinazolin-5-yl)amino)ethan-1-ol 12b*. White solid. Yield: 67%; m.p. 270–272°C; FT-IR (vmax, cm^-1^): 3327(OH), 3194 (NH), 3097 (CH aromatic), 2921 (CH aliphatic), 1594 (C = N); ^1^H NMR (400 MHz, DMSO-d6) δ 8.53 (dd, *J* = 8.1, 4.3 Hz, 3H), 8.45 (d, *J* = 8.1 Hz, 1H), 7.98 (t, *J* = 7.6 Hz, 1H), 7.85 (t, *J* = 7.9 Hz, 2H), 7.69 (d, *J* = 8.4 Hz, 2H), 4.90 (s, 1H), 3.75 (t, *J* = 6.0 Hz, 2H), 3.55 (q, *J* = 5.7 Hz, 2H); ^13^C NMR (DMSO-d6) δ ppm: 151.2, 145.3, 141.9, 133.4, 132.4, 130, 128.2, 127.8, 125.4, 123.6, 122.5, 122.2, 117.6, 57.8, 43.9. Anal. Calc. for: (C_17_H_14_ClN_5_O) (M.W. = 339).

*4*.*1*.*2*.*5*. *N*,*N-Dimethyl-3-(p-tolyl)-[1*,*2*,*4]triazolo[4*,*3-c]quinazolin-5-amine 13a*. White solid. Yield: 72%; m.p. 247–249°C; FT-IR (vmax, cm^-1^): 3055(CH aromatic), 2921 (CH aliphatic), 1509 (C = N); ^1^H NMR (400 MHz, DMSO-d6) δ 8.55–8.48 (m, 1H), 8.35–8.27 (m, 2H), 8.19 (d, *J* = 8.2 Hz, 1H), 8.02–7.95 (m, 1H), 7.90–7.83 (m, 1H), 7.43–7.36 (m, 2H), 3.09 (s, 6H), 2.39 (s, 3H); ^13^C NMR (DMSO-d6) δ ppm: 159.2, 148.7, 144.1, 140.8, 134.5, 131.7, 130.7, 128.6, 127.9, 125.6, 124.8, 122.4, 120.9, 43.9, 22.4. Anal. Calc. for: (C_18_H_17_N_5_) (M.W. = 303).

*4*.*1*.*2*.*6*. *3-(4-Chlorophenyl)-N*,*N-dimethyl-[1*,*2*,*4]triazolo[4*,*3-c]quinazolin-5-amine 13b*. White solid. Yield: 69%; m.p. 275–277°C; FT-IR (vmax, cm^-1^): 3096(CH aromatic), 2957 (CH aliphatic), 1505 (C = N); ^1^H NMR (400 MHz, DMSO-d6) δ 8.49 (dd, *J* = 8.0, 5.1 Hz, 1H), 8.46–8.38 (m, 2H), 8.18 (dd, *J* = 8.3, 4.6 Hz, 1H), 7.99–7.93 (m, 1H), 7.89–7.84 (m, 1H), 7.67–7.58 (m, 2H), 3.09 (s, 6H); ^13^C NMR (101 MHz, DMSO) δ 194.03, 160.44, 148.80, 144.40 136.85, 135.70, 130.97, 129.35, 129.11, 127.77, 126.03, 124.59, 123.53, 120.02, 40.97; Anal. Calc. for: (C_17_H_14_ClN_5_) (M.W. = 323).

*4*.*1*.*2*.*7*. *N-Cyclohexyl-3-(p-tolyl)-[1*,*2*,*4]triazolo[4*,*3-c]quinazolin-5-amine 14a*. White solid. Yield: 79%; m.p. 252–254°C; FT-IR (vmax, cm^-1^): 3325 (NH), 3066 (CH aromatic), 2922 (CH aliphatic), 1510 (C = N); ^1^H NMR (400 MHz, DMSO-d6) δ 8.50 (dt, *J* = 7.9, 1.1 Hz, 2H), 8.44–8.37 (m, 2H), 7.98 (td, *J* = 7.5, 1.1 Hz, 1H), 7.88 (ddd, *J* = 8.5, 7.3, 1.3 Hz, 1H), 7.47 (d, *J* = 7.0 Hz, 1H), 7.41–7.36 (m, 2H), 3.92 (s, 1H), 2.44 (s, 3H), 2.19 (s, 2H), 1.91–1.82 (m, 2H), 1.73 (d, *J* = 12.8 Hz, 1H), 1.44 (t, *J* = 9.6 Hz, 4H), 1.23 (s, 1H); ^13^C NMR (DMSO-d6) δ ppm: 152.9, 146.1, 143.5, 138.4, 134.2, 133.6, 130.5, 129, 127.9, 124.9, 123.9, 123.03, 116.5, 49.6, 35.1, 23, 22.6, 19.4. Anal. Calc. for: (C_22_H_23_N_5_) (M.W. = 357).

*4*.*1*.*2*.*8*. *3-(4-Chlorophenyl)-N-cyclohexyl-[1*,*2*,*4]triazolo[4*,*3-c]quinazolin-5-amine 14b*. White solid. Yield: 69%; m.p. 2261–263°C; FT-IR (vmax, cm^-1^): 3451 (NH), 3060 (CH aromatic), 2929 (CH aliphatic), 1591(C = N); ^1^H NMR (400 MHz, DMSO-d6) δ 8.44 (dd, *J* = 17.3, 8.2 Hz, 4H), 7.91 (t, *J* = 7.6 Hz, 1H), 7.81 (t, *J* = 7.8 Hz, 1H), 7.58 (d, *J* = 8.3 Hz, 2H), 7.41 (d, *J* = 6.9 Hz, 1H), 3.79 (s, 1H), 2.14 (d, *J* = 9.6 Hz, 2H), 1.83 (d, *J* = 10.3 Hz, 2H), 1.70 (d, *J* = 12.9 Hz, 1H), 1.51–1.33 (m, 4H), 1.30–1.14 (m, 1H); ^13^C NMR (DMSO-d6) δ ppm: 154.1, 145.1, 140.8, 136.5, 135.4, 132.9, 130.20, 129.7, 127.4, 122.8, 121.6, 120.9, 119.6, 53.6, 35.3, 28.4, 23.5. Anal. Calc. for: (C_21_H_20_ClN_5_) (M.W. = 377).

*4*.*1*.*2*.*9*. *4-(3-(p-Tolyl)-[1*,*2*,*4]triazolo[4*,*3-c]quinazolin-5-yl)morpholine 15a*. Buff powder (yield 70%); mp: 292–294 °C; FT-IR (vmax, cm^-1^): 3077 (CH aromatic), 2971 (CH aliphatic), 1546 (C = N); ^1^H NMR (400 MHz, DMSO-d6) δ 8.57 (d, *J* = 8.0 Hz, 1H), 8.33 (dd, *J* = 8.2, 2.5 Hz, 2H), 8.23 (d, *J* = 8.5 Hz, 1H), 8.12 (t, *J* = 7.6 Hz, 1H), 7.93 (t, *J* = 7.6 Hz, 1H), 7.46 (d, *J* = 8.2 Hz, 2H), 3.97 (d, *J* = 4.4 Hz, 4H), 3.44 (t, *J* = 4.5 Hz, 4H), 2.45 (s, 3H); ^13^C NMR (DMSO-d6) δ ppm: 160.6, 151, 146.1, 137.9, 135.8, 133, 130.7, 129.4, 127.9, 126.6, 125.1, 124.4, 120.6, 68.3, 53.8, 24.4. Anal. Calc. for: (C_20_H_19_N_5_O) (M.W. = 345).

*4*.*1*.*2*.*10*. *4-(3-(4-Chlorophenyl)-[1*,*2*,*4]triazolo[4*,*3-c]quinazolin-5-yl)morpholine 15b*. White solid. Yield: 69%; mp 263–265°C; FT-IR (vmax, cm^-1^): 3056 (CH aromatic), 2966 (CH aliphatic), 1507 (C = N); ^1^H NMR (400 MHz, DMSO-d6) δ 8.51 (d, *J* = 7.9 Hz, 1H), 8.42–8.37 (m, 2H), 8.17 (d, *J* = 8.2 Hz, 1H), 8.00 (t, *J* = 7.7 Hz, 1H), 7.91–7.85 (m, 1H), 7.69–7.62 (m, 2H), 3.91 (s, 4H), 3.40 (s, 4H); ^13^C NMR (101 MHz, DMSO-d6) δ 159.91, 149.05, 145.47, 136.97, 135.00, 133.26, 131.36, 130.20, 128.27, 127.75, 125.48, 120.55, 116.72, 63.30, 53.86; Anal. Calc. for: (C_19_H_16_ClN_5_O) (M.W. = 365).

*4*.*1*.*2*.*11*. *N-Propyl-3-(trifluoromethyl)-[1*,*2*,*4]triazolo[4*,*3-c]quinazolin-5-amine 16*. White solid. Yield: 77%; m.p. 269–271°C; FT-IR (vmax, cm^-1^): 3329 (NH), 3072 (CH aromatic), 2964 (CH aliphatic), 1597 (C = N); ^1^H NMR (400 MHz, DMSO-d6) δ 8.55 (dd, *J* = 7.8, 1.5 Hz, 1H), 8.41 (d, *J* = 8.1 Hz, 1H), 8.04–7.96 (m, 2H), 7.93 (td, *J* = 7.7, 1.5 Hz, 1H), 3.42–3.36 (m, 2H), 1.77 (h, *J* = 7.3 Hz, 2H), 0.96 (t, *J* = 7.4 Hz, 3H); ^13^C NMR (101 MHz, DMSO-d6) δ 155.93, 147.15, 140.8, 139.4, 136.73, 133.05, 124.72, 123.64, 122.51, 119.26, 40.62, 24.33, 15.02; Anal. Calc. for: (C_13_H_12_F_3_N_5_) (M.W. = 295).

*4*.*1*.*2*.*12*. *2-((3-(Trifluoromethyl)-[1*,*2*,*4]triazolo[4*,*3-c]quinazolin-5-yl)amino)ethan-1-ol 17*. White solid. Yield: 73%; m.p. 262–264°C; FT-IR (vmax, cm^-1^): 3335 (NH), 3097 (CH aromatic), 2940 (CH aliphatic), 1591 (C = N); ^1^H NMR (400 MHz, DMSO-d6) δ 8.53 (dd, *J* = 7.9, 1.4 Hz, 1H), 8.47–8.42 (m, 1H), 8.06–7.99 (m, 2H), 7.99–7.93 (m, 1H), 4.80 (t, *J* = 5.6 Hz, 1H), 3.75 (q, *J* = 5.8 Hz, 2H), 3.54 (q, *J* = 5.8 Hz, 2H); ^13^C NMR (101 MHz, DMSO-d6) δ 152.10, 142.22, 137.13, 134.83, 131.10, 126.91, 124.68, 121.58, 120.62, 117.37, 61.11, 47.71; Anal. Calc. for: (C_12_H_10_F_3_N_5_O) (M.W. = 297).

*4*.*1*.*2*.*13*. *N*,*N-Dimethyl-3-(trifluoromethyl)-[1*,*2*,*4]triazolo[4*,*3-c]quinazolin-5-amine 18*. White solid. Yield: 69%; m.p. 267–269°C; FT-IR (vmax, cm^-1^): 3061 (CH aromatic), 2949 (CH aliphatic), 1527 (C = N); ^1^H NMR (400 MHz, DMSO-d6) δ 8.52 (dd, *J* = 7.8, 1.6 Hz, 1H), 8.23 (dd, *J* = 8.2, 1.4 Hz, 1H), 8.02 (tt, *J* = 7.7, 1.3 Hz, 1H), 7.93 (ddt, *J* = 8.8, 7.4, 1.4 Hz, 1H), 3.08 (s, 6H); ^13^C NMR (101 MHz, DMSO-d6) δ 161.32, 145.05, 135.05, 132.97, 129.13, 125.96, 122.63, 118.73, 40.75; Anal. Calc. for: (C_12_H_10_F_3_N_5_) (M.W. = 281).

*4*.*1*.*2*.*14*. *N-Cyclohexyl-3-(trifluoromethyl)-[1*,*2*,*4]triazolo[4*,*3-c]quinazolin-5-amine 19*. White solid. Yield: 73%; m.p. 269–271°C; FT-IR (vmax, cm^-1^): 3325 (NH), 3071 (CH aromatic), 2938 (CH aliphatic), 1550 (C = N); ^1^H NMR (400 MHz, DMSO-d6) δ 8.57–8.45 (m, 2H), 8.04–7.97 (m, 1H), 7.96–7.89 (m, 1H), 7.61 (d, *J* = 6.8 Hz, 1H), 3.85 (dt, *J* = 7.1, 3.8 Hz, 1H), 2.15–2.03 (m, 2H), 1.81 (d, *J* = 11.8 Hz, 2H), 1.67 (d, *J* = 12.6 Hz, 1H), 1.38 (dp, *J* = 24.9, 12.4, 11.9 Hz, 4H), 1.20 (d, *J* = 11.8 Hz, 1H); ^13^C NMR (101 MHz, DMSO-d6) δ 153.09, 146.14, 137.81, 134.81, 133.02, 126.08, 124.66, 123.63, 121.51, 118.31, 52.23, 33.84, 26.88, 24.42; Anal. Calc. for: (C_16_H_16_F_3_N_5_) (M.W. = 335).

*4*.*1*.*2*.*15*. *4-(3-(Trifluoromethyl)-[1*,*2*,*4]triazolo[4*,*3-c]quinazolin-5-yl)morpholine 20*. White solid. Yield: 78%; m.p. 265–267°C; FT-IR (vmax, cm^-1^): 3079 (CH aromatic), 2961 (CH aliphatic), 1555 (C = N); ^1^H NMR (400 MHz, DMSO-d6) δ 8.59 (dd, *J* = 7.9, 1.5 Hz, 1H), 8.25 (d, *J* = 8.2 Hz, 1H), 8.09 (td, *J* = 7.6, 1.1 Hz, 1H), 7.99 (ddt, *J* = 8.4, 7.3, 1.2 Hz, 1H), 3.91–3.85 (m, 4H), 3.40 (t, *J* = 4.6 Hz, 4H); ^13^C NMR (101 MHz, DMSO-d6) δ 158.00, 143.92, 138.38, 134.30, 131.27, 128.64, 125.05, 122.72, 121.53, 121.31, 67.08, 53.77; Anal. Calc. for: (C_14_H_12_F_3_N_5_O) (M.W. = 323).

### 4.2. Biological evaluation

#### 4.2.1. *In vitro* anti-proliferative activity

The anti-proliferative activity of the synthesized compounds was assessed using MTT assay protocol [26–28, 56]. A panel of human cancer cell lines namely, colorectal carcinoma (HCT-116) and hepatocellular carcinoma (HepG2) was used in this test. Doxorubicin was used as a positive control. The cell lines were got from ATCC (American Type Culture Collection) via the Holding company for biological products and vaccines (VACSERA, Cairo, Egypt). The anti-proliferative activities of the tested compounds were determined quantitatively as follows:

At first, the cells were cultured into a medium of RPMI-1640 with 10% fetal bovine serum. Then, two different antibiotics were added at 37°C in a 5% CO2 incubator: penicillin (100 units/mL) and streptomycin (100 μg/mL). Next, we seeded the cells in a 96-well plate by a density of 1.0 x 104 cells / well at 37°C for 48 h under 5% CO2. The synthesized compounds with different concentrations were applied into the cell lines and incubated for 48 h. After 48 h, 20 μl of MTT solution (5mg/mL) was added and incubated for 4 h. Then, DMSO (100 μl) was added into each well to dissolve the formed purple formazan. After that, a colorimetric assay was measured and recorded at absorbance of 570 nm using a plate reader (EXL 800, USA). The relative cell viability in percentage was calculated as (A570 of treated samples/A570 of untreated sample) X 100. Results for IC50values of the active compounds were summarized in **Table 1**.

#### 4.2.2. *In vitro* Topo II inhibitory assay

The most active anti-proliferative members (**13a**, **14a**, **16**, **17**, **18, 19,** and **20**) were analyzed for their Topo II inhibitory activities. The reported method described by Patra *et al* [57] was applied using Topo II drug screening kit (TopoGEN, Inc., Columbus). Doxorubicin was used as a positive control.

A typical enzyme reaction was structured to determine Topo II activity. The reaction mixture included Topo II (2 μl), substrate super coiled pHot1 DNA (0.25 μg), 50 μg/ml test compound (2 μl), and assay buffer (4 μl). To start the reaction, the mixture was allowed to incubate in 37°C for 30 min. To terminate the reaction, a mixture of 10% sodium dodecylsulphate (2 μl) and proteinase K (50 μg/mL) was added at 37°C for 15 min. then incubated for 15 min at 37°C.

After that, the DNA was run on 1% agarose gel in BioRad gel electrophoresis system for 1–2 h followed by staining with GelRedTM stain for 2 h and destained for 15 min with TAE buffer. The gel was imaged via BioRad’s Gel DocTMEZ system. Both supercoiled and linear strands DNA were incorporated in the gel as markers for DNA-Topo II intercalators. The results of IC_50_ values were calculated using the GraphPad Prism version 7. Each reaction was performed in duplicate, and at least three independent determinations of each IC_50_ were made.

#### 4.2.3. DNA/Methyl green assay

The most active anti-proliferative members (**13a**, **14a**, **16**, **17**, **18, 19,** and **20**) were evaluated for their DNA-binding affinities, using doxorubicin **as** a positive control according to methyl green dye method described by Burres *et al* [29]. Activated Calf Thymus DNA (Merk, Germany) was treated with methyl green (Merk, Germany), then the synthesized compounds were applied to displace the methyl green dye, producing equivalent color. The results were reported as a 50% inhibition concentration values (IC_50_) calculated by linear regression of data plotted on a semi-log scale and summarized in **Table 1**.

#### 4.2.4. Topo II-mediated DNA cleavage assay

Topo II-mediated DNA cleavage assay was carried out according the reported method by Huang et al. [58] as follows.

Topo II α (10 U), supercoiled pBR322 DNA (0.2 μg) and compound **16** at concentration of 5 and 10 μM were added in Topo II buffer (0mM Trise HCl, pH 8.0, 150 mM NaCl, 10 mM MgCl2 2mM ATP, 0.5mM dithiothreitol, and 30 μg/mL BSA) of 20 μL volume. After incubating for 6 min at 37°C and then respectively adding 2 μL of 10% SDS, 2 μL of 250mM NaEDTA, pH 8.0, 2 μL of 0.8 mg/mL Proteinase K. Following reactions were incubated for another 30 min at 45°C. Samples were mixed with 4 μL of 6×loading buffer, heated at 70°C for 2 min and subjected to electrophoresis in a 1% agarose gel in 1×TAE buffer (30 mL) with 1 μL Gel Red. Finally, DNA bands were visualized by using UV light, photographed by using Alpha Innotech digital imaging system.

#### 4.2.5. Flow cytometry analysis for cell cycle

Cell cycle analysis was performed using propidium iodide (PI) staining and flow cytometry analysis [35, 59, 60] for compound **16** using flow cytometry analysis as follows.

Flow Cytometry Kit for Cell Cycle Analysis (ab139418_Propidium Iodide Flow Cytometry Kit/BD) was used in this test. HepG2 cells were treated with compound **16** (2.44 μM) for 48 h. Then, the cells were fixed in 70% ethanol at 4°C for 12 h. After that, the cells were washed with cold PBS, incubated with 100 μl RNase A at 37°C for 30 min, and stained with 400 μl PI in the dark at room temperature for further 30 min. The stained cells were measured using Epics XL-MCL™ Flow Cytometer (Beckman Coulter), and the data were analyzed using Flowing software (version 2.5.1, Turku Centre for Biotechnology, Turku, Finland).

#### 4.2.6. Flow cytometry analysis for apoptosis

Flow cytometry cell apoptosis analysis [36, 61] was used to investigate the apoptotic effect of compound **16** as follows.

HepG2 cells were treated with compound **16** (2.44 μM) for 48 h, collected by trypsin, centrifuged, washed two successive times with PBS, suspended in 500 μl binding buffer, and double stained with 5 μl Annexin V-FITC and 5 μl PI in the dark at room temperature for 15 min. The stained cells were measured using Epics XL-MCL™ Flow Cytometer and analyzed using Flowing software.

#### 4.2.7. Western blot analysis

The effects of compound **16** on the expression of BAX, and Bcl‐2 were determined using Western blot analysis [62–64]as described in **Electronic Supplementary Material S2.7 in S1 Appendix**.

### 4.3. *In silico* studies

#### 4.3.1. Docking studies

Docking studies were carried out utilizing discovery studio 4.0. The 3D crystal structure of the target macromolecule (DNA-topoisomerase II complex) was obtained from the protein databank (PDB ID: 4G0U, resolution: 2.7 Å).

At first, the co-crystallized ligand and water molecules were deleted from the DNA-topoisomerase II complex, leaving protein and DNA. Then, Valence monitor option was applied to correct any incorrect valence. Next, the energy of the complex was minimized by applying CHARMM and MMFF94 force fields. After that, the active binding site was defined and prepared for docking. The structures of the synthesized compounds and doxorubicin were sketched using ChemBioDraw Ultra 14.0 and saved in MDL-SD file format. Next, the MDL-SD file was opened, 3D structures were protonated, and the energy minimized by applying CHARMM and MMFF94 force fields then prepared for docking.

CDOCKER protocol was used for carrying out the docking studies. A maximum of 10 conformers was considered for each molecule in the docking analysis. Finally, the most ideal pose was selected according to its binding free energy with DNA–Topo II as well as its binding mode with the target molecule [65–69].

#### 4.3.2. Toxicity studies

The toxicity parameters of the synthesized compounds were calculated using Discovery studio 4.0. Sorafenib was used as a reference drug. At first, the CHARMM force field was applied then the compounds were prepared and minimized according to the preparation of small molecule protocol. Then different parameters were calculated from toxicity prediction (extensible) protocol [70].

#### 4.3.3. Molecular dynamic simulation and binding free energy calculation using MM-GBSA

Molecular dynamic simulation MM-GBSA studies were carried out using GROMACS 2019 [71–78] as described in **Electronic Supplementary Material S5 in S1 Appendix**.

## Supporting information

S1 Appendix Electronic supplementary material. Electronic supplementary material related to this manuscript is found in a separate file.
(PDF)
