# Supplementary material for: New [1,2,4]triazolo[4,3-c]quinazolines as intercalative Topo II inhibitors: Design, synthesis, biological evaluation, and in silico studies
Source: PLoS One. 2023 Jan 30;18(1):e0274081. doi: 10.1371/journal.pone.0274081 (PMC9886266; doi:10.1371/journal.pone.0274081)
Supplement: S1 Appendix — Electronic supplementary material related to this manuscript is found in a separate file. (PDF) [file pone.0274081.s001.pdf]

New [1,2,4]triazolo[4,3-*c*]quinazolines as intercalative Topo II inhibitors: Design, synthesis, biological evaluation, and *in silico* studies

Ahmed A. Gaber[a], Mohamed Sobhy[a], Abdallah Turky[a], Wagdy M. Eldehna[b], Samiha A. El-Sebaey[c], Souad A. El-Metwally[d], Abeer M. El-Naggar[e], Ibrahim M. Ibrahim[f], Eslam B. Elkaeed[g], Ahmed M. Metwaly[h, i], Ibrahim H. Eissa[j],

[a] Department of Pharmaceutical Organic Chemistry, Faculty of Pharmacy (Boys), Al-Azhar University, Cairo 11884, Egypt.

[d] Department of Pharmaceutical Chemistry, Faculty of Pharmacy, Kafrelsheikh University, Kafrelsheikh, 33516, Egypt.

[c] Department of Pharmaceutical Organic Chemistry, Faculty of Pharmacy (Girls), Al-Azhar University, Cairo 11754, Egypt.

[d] Department of Basic Science, Higher Technological institute, 10th of Ramadan City, Egypt.

[e] Department of Chemistry, Faculty of Science, Ain Shams University, Abassia, Cairo 11566, Egypt.

[f] Biophysics Department, Faculty of Science, Cairo University

[g] Department of Pharmaceutical Sciences, College of Pharmacy, AlMaarefa University, Ad Diriyah 13713, Riyadh, Saudi Arabia.

[h] Pharmacognosy and Medicinal Plants Department, Faculty of Pharmacy (Boys), Al-Azhar University, Cairo 11884, Egypt

[i] Biopharmaceutical Products Research Department, Genetic Engineering and Biotechnology Research Institute, City of Scientific Research and Technological Applications (SRTA-City), Alexandria, Egypt

[j] Pharmaceutical Medicinal Chemistry & Drug Design Department, Faculty of Pharmacy (Boys), Al-Azhar University, Cairo 11884, Egypt.

*** Corresponding author:**

Ibrahim H. Eissa

Pharmaceutical Medicinal Chemistry & Drug Design Department, Faculty of Pharmacy (Boys), Al-Azhar University, Cairo 11884, Egypt

Email: Ibrahimeissa@azhar.edu.eg

Content

**S1**	Chemistry (Structures of the final compounds, Reagents, solvents, and apparatus)
**S2**	Biological tests
**S3**	Docking studies procedures and figures
**S4**	*In silico* toxicity studies procedure and results
**S5**	Molecular Dynamic Simulation
**S6**	Raw data of biological testing
**S7**	Spectral data

S1: Chemistry

All melting points were taken on electro-thermal (LA 9000 SERIS) digital melting point apparatus (UK) at The Pharmaceutical Analytical Unit, Faculty of Pharmacy, Al-Azhar University. IR spectra were recorded on Pye Unicam SP 1000 IR spectrophotometer (Russian Federation) at The Pharmaceutical Analytical Unit, Faculty of Pharmacy, Al-Azhar University. The ^{1}HNMR spectra and the ^{13}CNMR spectra were recorded in DMSO-d_6 on BURKER 400 MHZ spectrophotometer (Germany) at the Nuclear Magnetic Resonance Lab, Faculty of Pharmacy, Al-Mansoura University, Egypt. Chemical shifts were expressed in δ (ppm) with reference to TMS and coupling constant (J) in Hertz using DMSO-d_6 as solvent. Progress of reactions were monitored by TLC using TLC sheets coated with UV fluorescent silica gel (Kieselgel 0.25mm, 60 F254, Merck Germany) with a developing solvent system of DCM/methanol (95:5) and were visualized using UV lamp.

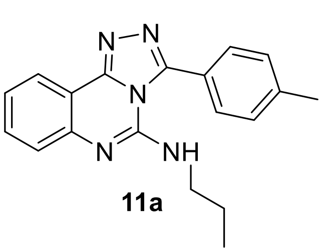

11a

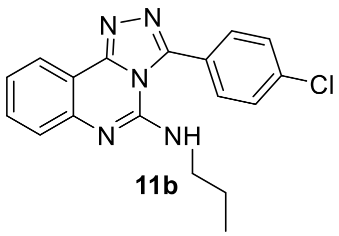

11b

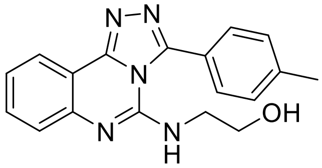

12a

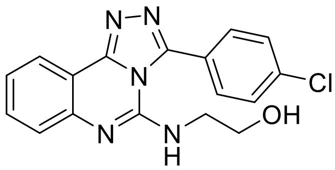

12b

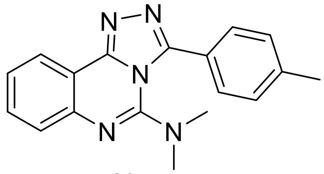

13a

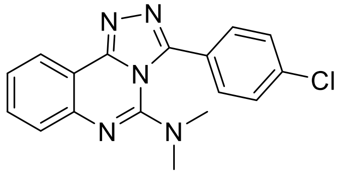

13b

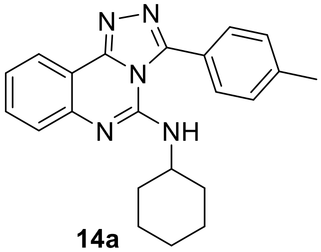

14a

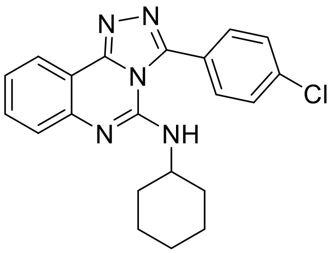

14b

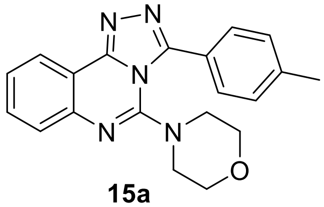

15a

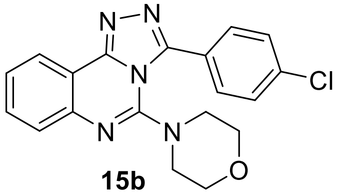

15b

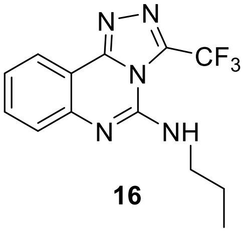

16

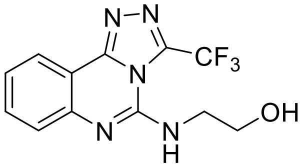

17

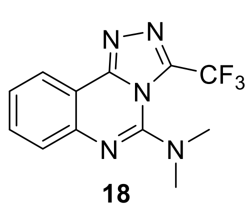

18

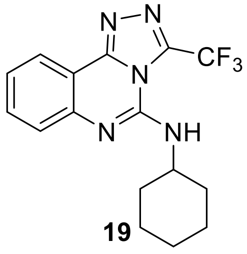

19

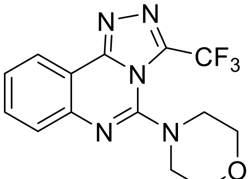

20

S2: Biological evaluation

S2.1. *In vitro* cytotoxic activity

The anti-proliferative activity of the synthesized compounds was assessed using MTT assay protocol [1-3]. A panel of human cancer cell lines namely, colorectal carcinoma (HCT-116) and hepatocellular carcinoma (HepG2) was used in this test. As we targeted the DNA and Topo II, doxorubicin as a potential intercalative Topo II inhibitor was used as a positive control. The cell lines were got from ATCC (American Type Culture Collection) via the Holding company for biological products and vaccines (VACSERA, Cairo, Egypt). The anti-proliferative activities of the tested compounds were determined quantitatively as follows:

At first, the cells were cultured into a medium of RPMI-1640 with 10% fetal bovine serum. Then, two different antibiotics were added at 37 ˚C in a 5% CO_2 incubator: penicillin (100 units/mL) and streptomycin (100 µg/mL). Next, we seeded the cells in a 96-well plate by a density of 1.0 x 10^4 cells / well at 37 ˚C for 48 h under 5% CO_2. The synthesized compounds with different concentrations were applied into the cell lines and incubated for 48 h. After 48 h, 20 µl of MTT solution (5mg/mL) was added and incubated for 4 h. Then, DMSO (100 µl) was added into each well to dissolve the formed purple formazan. After that, a colorimetric assay was measured and recorded at absorbance of 570 nm using a plate reader (EXL 800, USA). The relative cell viability in percentage was calculated as (A570 of treated samples/A570 of untreated sample) X 100. Results for IC_{50}values of the active compounds were summarized in **Table 2**.

S2.2. Measurement of topoisomerase II Activity

The most active anti-proliferative members (**13a**, **14a**, **16**, **17**, **18, 19,** and **20**) were analyzed for their Topo II inhibitory activities. The reported method described by Patra *et al* [4] was applied using Topo II drug screening kit (TopoGEN, Inc., Columbus). Doxorubicin was used as a positive control.

A typical enzyme reaction was structured to determine Topo II activity. The reaction mixture included Topo II (2 µl), substrate super coiled pHot1 DNA (0.25 µg), 50 µg/ml test compound (2 µl), and assay buffer (4 µl). To start the reaction, the mixture was allowed to incubate in 37 ºC for 30 min. To terminate the reaction, a mixture of 10% sodium dodecylsulphate (2 µl) and proteinase K (50 µg/mL) was added at 37ºC for 15 min. then incubated for 15 min at 37ºC.

After that, the DNA was run on 1% agarose gel in BioRad gel electrophoresis system for 1–2 h followed by staining with GelRedTM stain for 2 h and destained for 15 min with TAE buffer. The gel was imaged via BioRad's Gel DocTMEZ system. Both supercoiled and linear strands DNA were incorporated in the gel as markers for DNA-Topo II intercalators. The results of IC_{50} values were calculated using the GraphPad Prism version 7. Each reaction was performed in duplicate, and at least three independent determinations of each IC_{50} were made.

S2.3. DNA/Methyl Green Assay

The most active anti-proliferative members (**13a**, **14a**, **16**, **17**, **18, 19,** and **20**) were evaluated for their DNA-binding affinities, using doxorubicin **as** a positive control according to methyl green dye method described by Burres *et al* [5]. Activated Calf Thymus DNA (Merk, Germany) was treated with methyl green (Merk, Germany), then the synthesized compounds were applied to displace the methyl green dye, producing equivalent color. The results were reported as a 50% inhibition concentration values (IC_{50}) calculated by linear regression of data plotted on a semi-log scale and summarized in **Table 3**.

S2.4. Topo II-mediated DNA cleavage assay

Topo II α (10 U), supercoiled pBR322 DNA (0.2 μg) and compound **16** at concentration of 5 and 10 μM were added in Topo II buffer (0mM Trise HCl, pH 8.0, 150 mM NaCl, 10 mM $MgCl_2$ 2mM ATP, 0.5mM dithiothreitol, and 30 μg/mL BSA) of 20 μL volume. After incubating for 6 min at 37 °C and then respectively adding 2 μL of 10% SDS, 2 μL of 250mM NaEDTA, pH 8.0, 2 μL of 0.8 mg/mL Proteinase K. Following reactions were incubated for another 30 min at 45 °C. Samples were mixed with 4 μL of 6×loading buffer, heated at 70 °C for 2 min and subjected to electrophoresis in a 1% agarose gel in 1×TAE buffer (30 mL) with 1 μL Gel Red. Finally, DNA bands were visualized by using UV light, photographed by using Alpha Innotech digital imaging system.

S2.5. Flow cytometry analysis for cell cycle

To determine the role of the synthesized compounds in cell cycle distribution, cell cycle analysis was performed using propidium iodide (PI) staining and flow cytometry analysis for compound **16**. Flow Cytometry Kit for Cell Cycle Analysis (ab139418_Propidium Iodide Flow Cytometry Kit/BD) was used in this test. HepG2 cells were treated with compound **16** (2.44 μM)

for 48 h. Then, the cells were fixed in 70% ethanol at 4 °C for 12 h. After that, the cells were washed with cold PBS, incubated with 100 µl RNase A at 37 °C for 30 min, and stained with 400 µl PI in the dark at room temperature for further 30 min. The stained cells were measured using Epics XL-MCL™ Flow Cytometer (Beckman Coulter), and the data were analyzed using Flowing software (version 2.5.1, Turku Centre for Biotechnology, Turku, Finland).

S2.6. Flow cytometry analysis for apoptosis

Flow cytometry cell apoptosis analysis was used to investigate the apoptotic effect of the synthesized compounds. HepG2 cells were treated with compound **16** (2.44 µM) for 48 h, collected by trypsin, centrifuged, washed two successive times with PBS, suspended in 500 µl binding buffer, and double stained with 5 µl Annexin V-FITC and 5 µl PI in the dark at room temperature for 15 min. The stained cells were measured using Epics XL-MCL™ Flow Cytometer and analyzed using Flowing software.

S2.7. Western blot analysis

The proteins expression of BAX, and Bcl-2 were determined using Western blot analysis. In brief, HepG2 cells were treated with control, or with the synthesized compound **16**. Then cells were lysed in 250 µL precold lysis buffer (pH 7.4: Tris-Base [10 mM], NaCl [100 mM], ethylenediaminetetraacetic acid [EDTA, 25 mM], ethylene glycol bis (2-aminoethyl) tetraacetic acid [EGTA, 25 mM], 1% [v/v] NP-40, and 1% [v/v] Triton X-100) supplemented with 1:350 protease:phosphatase inhibitors cocktail (Sigma). The cells were immediately frozen at −20°C for 1.5 hours for further lysis, collected by cell scraper, sonicated 3 × 10 seconds, and centrifuged (13000 rpm, 15 minutes). Total protein concentrations in the supernatant were determined colorimetrically using the Pierce 660 nm Protein Assay method (Thermo Fisher Scientific, Rockford, IL), with BSA as the standard. Equal amounts of protein (25 µg) samples were mixed with SDS-loading buffer (pH 6.8: Tris-HCl [700 mM], dithiothreitol [DTT, 600 mM], sodium dodecyl sulfate [SDS, 12%], glycerol [60%], and bromophenol blue [0.012%]), denatured by boiling at 95°C for 10 minutes, allowed to cool on ice for 15 minutes, vortexed vigorously for 30 seconds and loaded into SDS-polyacrylamide gel and separated by an electrophoresis unit (Cleaver Scientific Ltd, UK), transferred onto polyvinylidene fluoride membranes (Bio-Rad) for 35 minutes using a Trans-Blot SD semi-dry transfer cell (Bio-Rad) at 250 mA and 22 V. Membranes were

blocked with 5% (w/v) blotting grade dry milk (Bio-Rad) in Tris-buffered saline/Tween-20 (TBS-T) (pH 7.5: Tris-base [20 mM], NaCl [150 mM], and 0.05% [v/v] Tween- 20) while shaking for 1.5 hour at RT, and then incubated with the corresponding primary antibody against β-actin (1:2000, #A5060; Sigma), BAX (Biovision, USA) and Bcl-2(Bioimaging, system, syngene, UK) for 9-10 hours at 4°C in a humidified chamber. The blots were washed with TBS-T three times for 15 minutes and incubated with matched horseradish peroxidase (HRP)-linked secondary antibodies (Dako, Denmark) for another 1 hour at RT, followed by washing 3 × 15 min with TBS-T. After membranes were incubated at RT with 1:1 reagent mixture of chemiluminescence Western Lightning ECL (Perkin Elmer, Waltham, MA) for 1 minute, the bands were visualized in Chemi-Doc imager (Bio-Rad). Means of the detected blot intensities were then quantified, analyzed by the combined Bio-Rad Image Lab software and their corresponding background subtracted, with normalization to the corresponding bands density of β-actin as the sampling loading control. Data were collected from three separate experiments.

S2.7. Statistical analyses

All data obtained from the biological evaluation studies are presented as mean ± SEM values and analyzed with unpaired student's *t*-test using GraphPad Prism version 7 (GraphPad Software, San Diego, CA), with $p < 0.05$ was considered statistically significant.

S2.8. Western blots

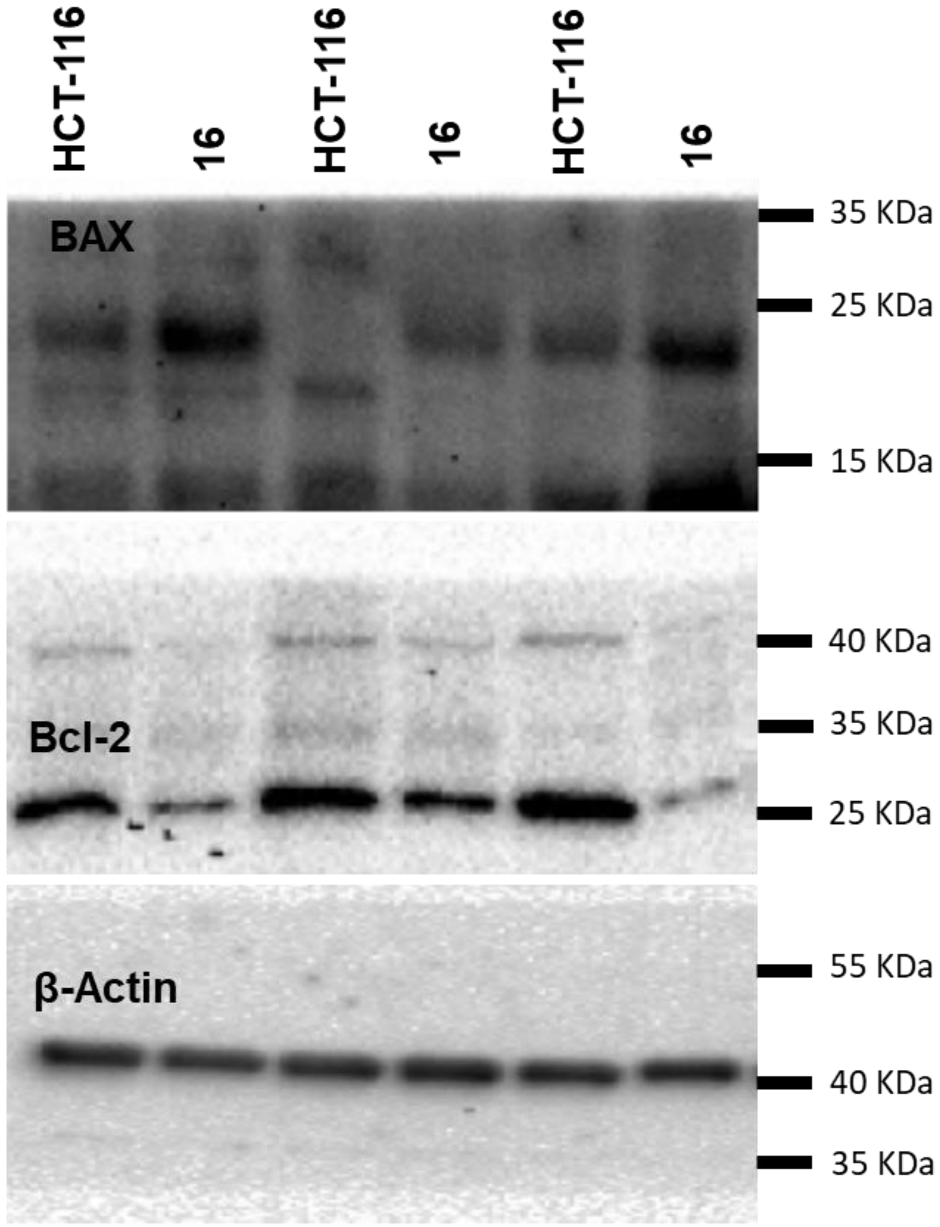

S3. Docking studies

Docking studies were carried out utilizing discovery studio 4.0. The 3D crystal structure of the target macromolecule (DNA-topoisomerase II complex) was obtained from the protein databank (PDB ID: 4G0U, resolution: 2.7 Å).

At first, the co-crystallized ligand and water molecules were deleted from the DNA-topoisomerase II complex, leaving protein and DNA. Then, Valence monitor option was applied to correct any incorrect valence. Next, the energy of the complex was minimized by applying CHARMM and MMFF94 force fields. After that, the active binding site was defined and prepared for docking. The structures of the synthesized compounds and doxorubicin were sketched using ChemBioDraw Ultra 14.0 and saved in MDL-SD file format. Next, the MDL-SD file was opened, 3D structures were protonated, and the energy minimized by applying CHARMM and MMFF94 force fields then prepared for docking.

CDOCKER protocol was used for carrying out the docking studies. A maximum of 10 conformers was considered for each molecule in the docking analysis. Finally, the most ideal pose was selected according to its binding free energy with DNA–Topo II as well as its binding mode with the target molecule.

S3.1. Binding of compound **11a** with DNA-Topo II complex.

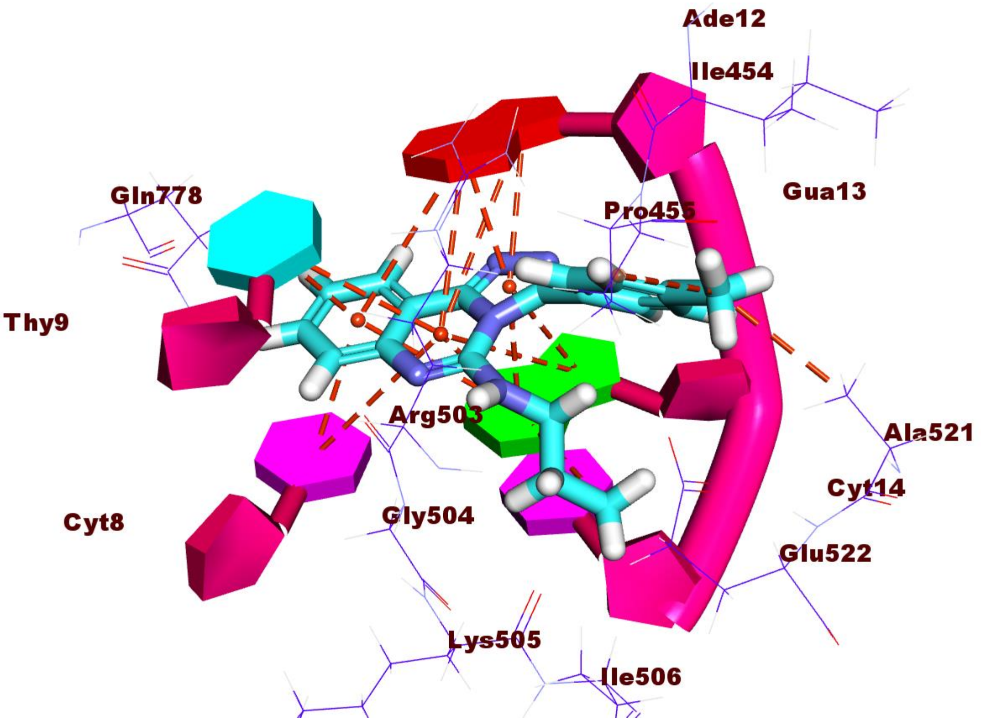

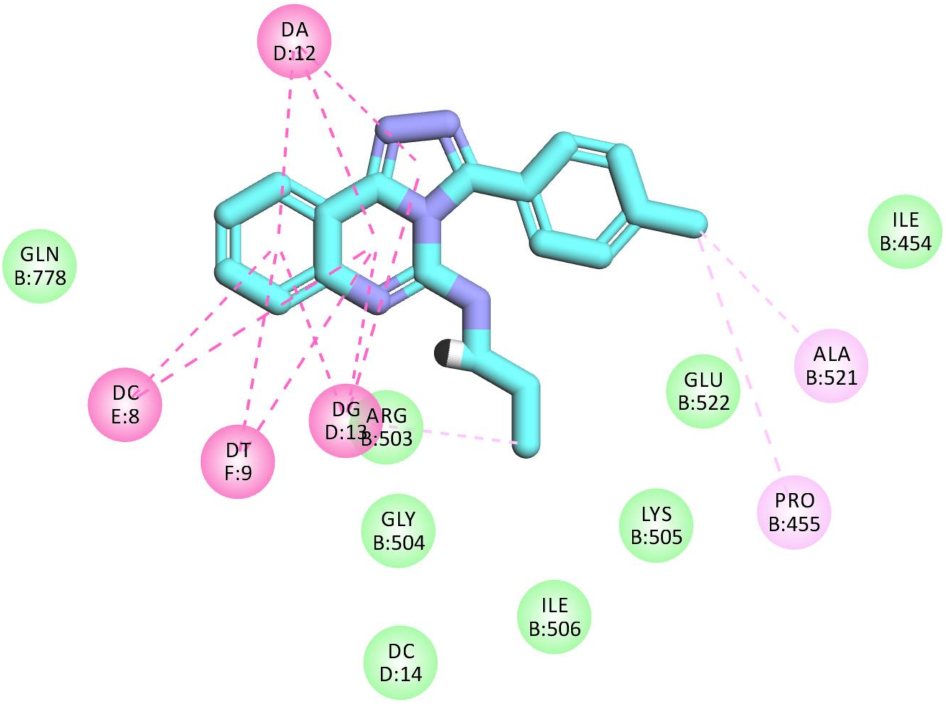

S3.2. Binding of compound **12b** with DNA-Topo II complex.

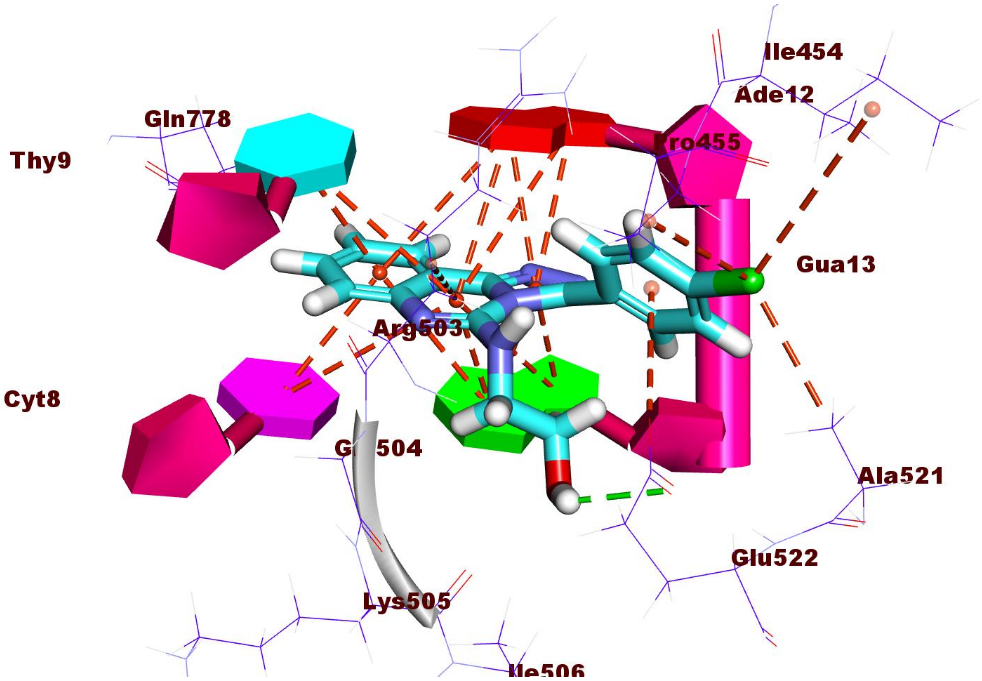

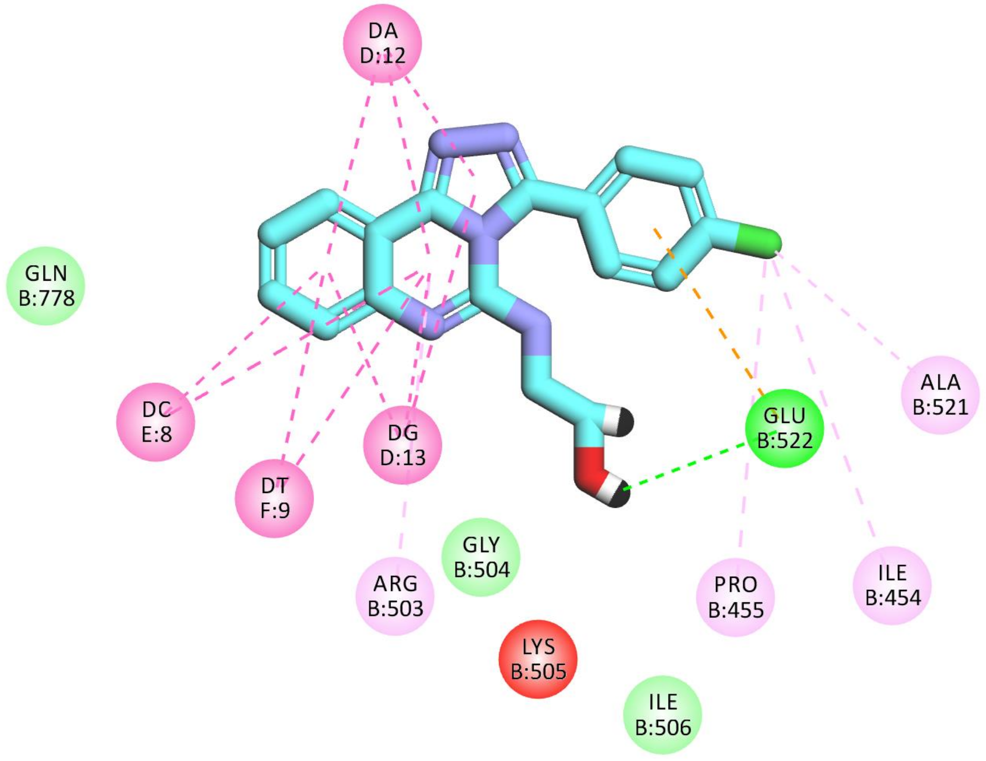

S3.3. Binding of compound **17** with DNA-Topo II complex.

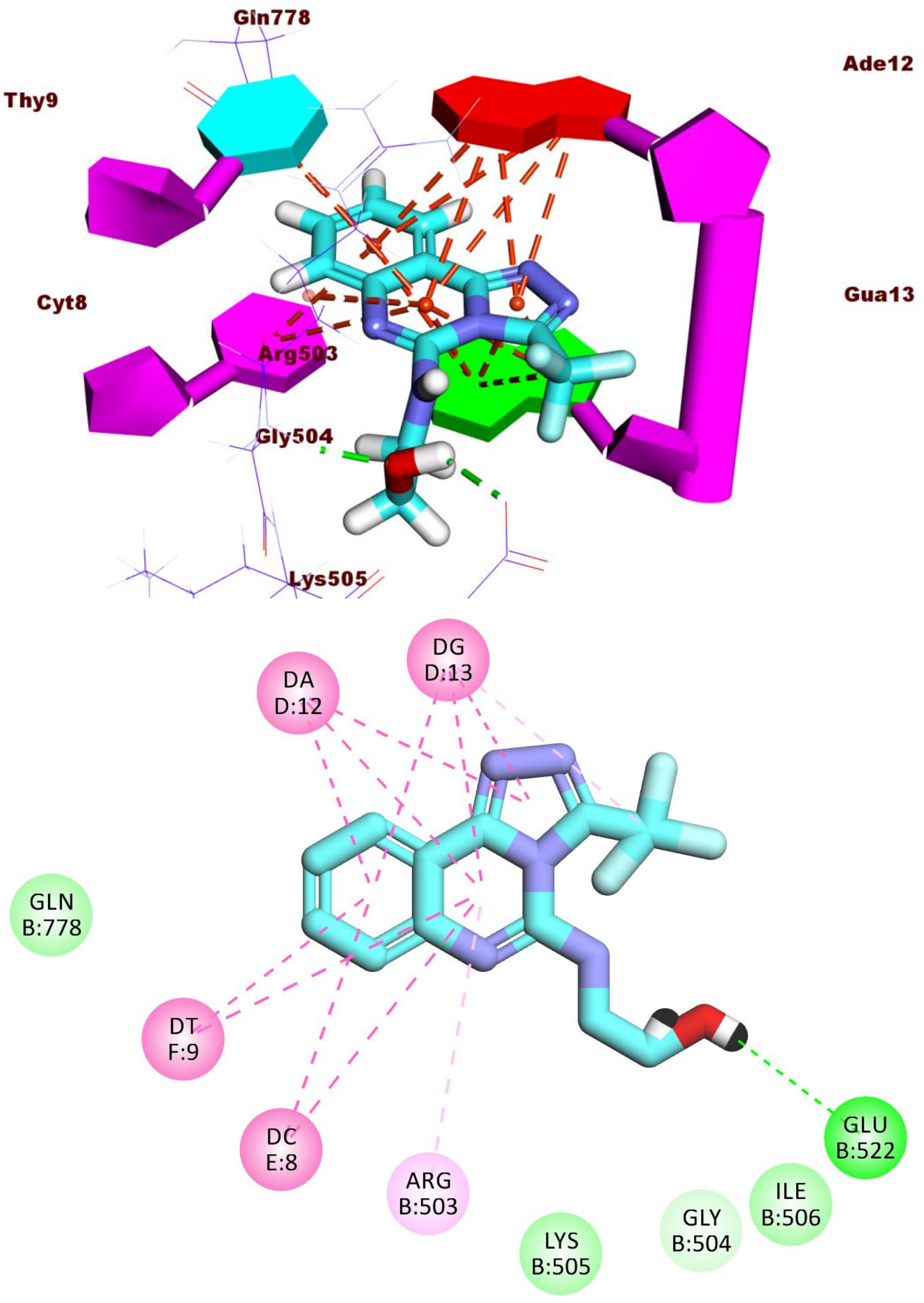

S3.4. Binding of compound **19** with DNA-Topo II complex.

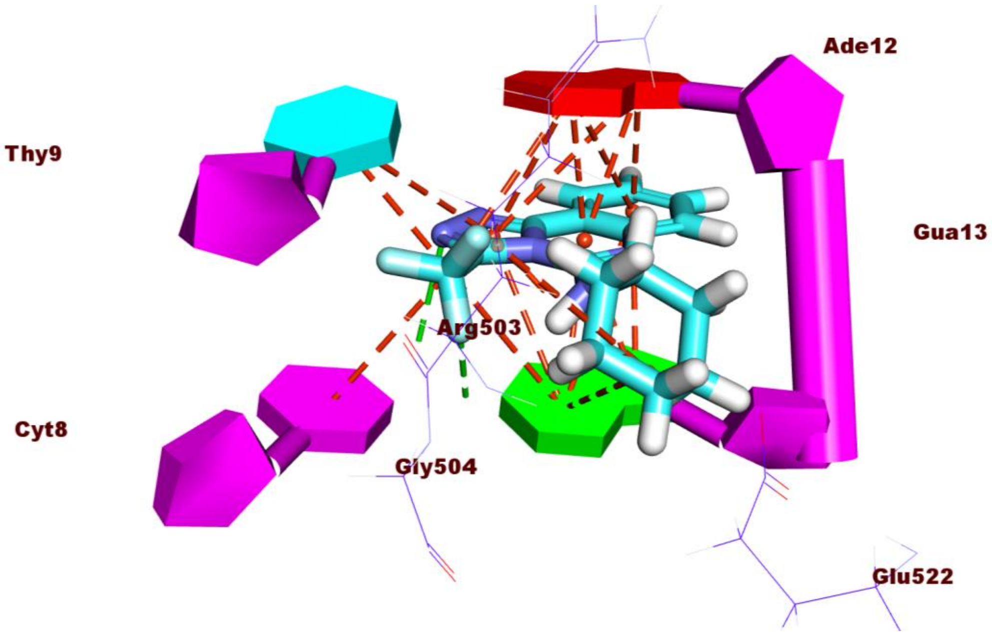

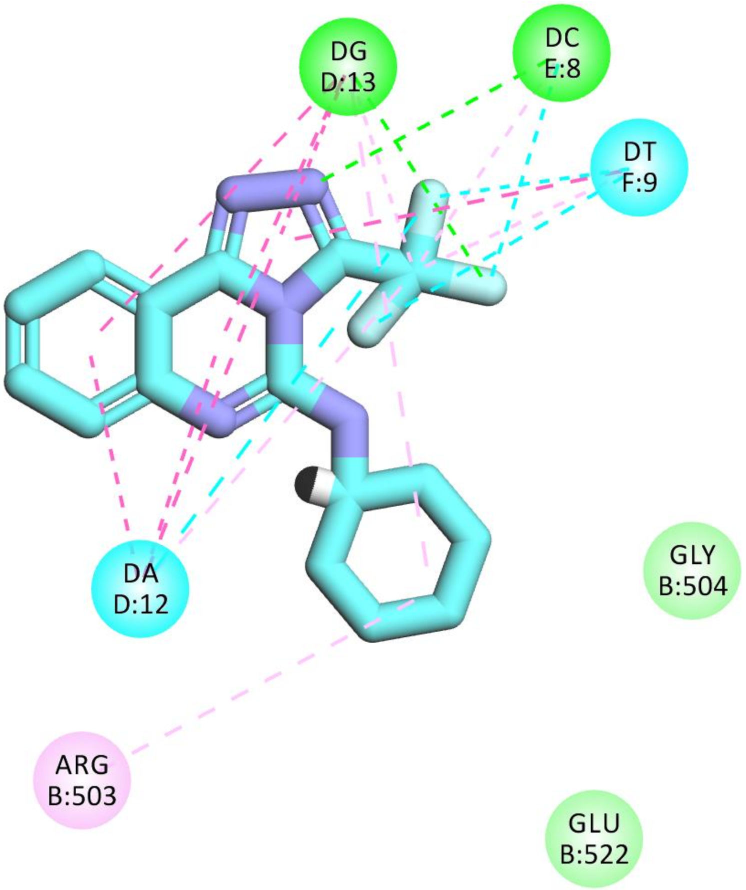

S4. Toxicity studies

The toxicity parameters of the synthesized compounds were calculated using Discovery studio 4.0. Sorafenib was used as a reference drug. At first, the CHARMM force field was applied then the compounds were prepared and minimized according to the preparation of small molecule protocol. Then different parameters were calculated from toxicity prediction (extensible) protocol.

S4.1. Toxicity properties of compounds

**Comp.**	FDA Rodent Carcinogenicity (Mouse- female)	Carcinogenic Potency TD_{50} (Rat) [a]	Rat Maximum Tolerated Dose (Feed) [b]	Rat Oral LD_{50} [b]	DPT	Rat Chronic LOAEL[b]	Skin Irritancy
**11a**	Non-Carcinogen	5.004	0.177	0.286	Non-Toxic	0.033	Non-Irritant
**11b**	Non-Carcinogen	4.274	0.221	0.160	Non-Toxic	0.015	Non-Irritant
**12a**	Non-Carcinogen	7.793	0.193	0.623	Non-Toxic	0.029	Non-Irritant
**12b**	Non-Carcinogen	6.653	0.241	0.225	Non-Toxic	0.013	Non-Irritant
**13a**	Non-Carcinogen	0.787	0.057	0.136	Non-Toxic	0.041	Non-Irritant
**13b**	Non-Carcinogen	0.674	0.071	0.078	Non-Toxic	0.021	Non-Irritant
**14a**	Non-Carcinogen	0.150	0.111	0.208	Non-Toxic	0.041	Non-Irritant
**14b**	Non-Carcinogen	0.127	0.138	0.075	Non-Toxic	0.018	Non-Irritant
**15a**	Non-Carcinogen	1.735	0.057	0.441	Non-Toxic	0.044	Non-Irritant
**15b**	Non-Carcinogen	1.474	0.070	0.159	Non-Toxic	0.020	Non-Irritant
**16**	Non-Carcinogen	28.052	0.127	0.072	Non-Toxic	0.010	Non-Irritant

**17**	Non-Carcinogen	43.706	0.139	0.101	Non-Toxic	0.009	Non-Irritant
**18**	Non-Carcinogen	4.394	0.041	0.035	Non-Toxic	0.014	Irritant
**19**	Non-Carcinogen	0.846	0.081	0.034	Non-Toxic	0.013	Non-Irritant
**20**	Non-Carcinogen	9.782	0.041	0.062	Non-Toxic	0.014	Irritant
**Amsacrine**	Non-Carcinogen	3.569	0.068	3.005	Toxic	0.025	Non-Irritant

[a] Unit: mg/kg body weight/day

[b] Unit: g/kg body weight

S5: Molecular Dynamic Simulation:

To prepare the protein-small molecule-DNA complex, we utilized CHARMM-GUI webserver to prepare the necessary files for molecular dynamic (MD) simulation [6-9]. To solvate the system, we used TIP3P water model with a padding of 10 Å in a cubic box and then neutralized the system by adding Na^+ and Cl^- ions to the physiological concentration of 0.154 M. The parameters of the protein and DNA were taken from CHAMM36 force field while CHARMM general force field (CGenFF) tool implemented in CHARMM-GUI was utilized to parameterize the ligand. GROMACS 2019 [10] was utilized to perform all the steps of the MD simulation with periodic boundary conditions (PBC) applied. First, a minimization step was needed to remove any clashes between atoms using the steepest descent algorithm. The minimization converged when the maximum force between any atom reached less than 100 $KJ.mol^{-1}.nm^{-1}$. After that, two equilibration steps were performed starting with a constant number of atoms, constant volume, and constant temperature (NVT) ensemble followed by a constant number of atoms, constant pressure, and constant temperature (NPT) ensemble. The temperature and pressure were set to 310 K, and 1 atmospheric pressure and they were maintained by V-rescale algorithm and Berendsen barostat, respectively [11]. Finally, a production run of 100 ns in the NVT ensemble was performed. In each step, the bond lengths of hydrogen-bonded atoms were constrained using LINear Constraint Solver (LINCS) algorithm[12]. The calculation of electrostatics was performed using Particle Mesh Ewald (PME) [13] algorithm with a cutoff of 1.2 nm. To integrate the Newtonian equations of motions, the Leap-frog algorithm was used with a time step of 1 femtosecond for the two equilibration steps and 2 femtoseconds for the production step. The production run was saved for every 100 picoseconds with a total of 1000 frames. Before analyzing the trajectory, the PBC was removed. The analysis of the production trajectory was performed using VMD TK scripts. Root mean square deviation (RMSD), solvent accessible surface area (SASA), radius of gyration (RoG), root mean square fluctuation (RMSF) and the number of hydrogen bonds were calculated [14]. Afterwards, the trajectory was clustered using TTClust [15] to get a representative frame for each cluster. First, backbone alignment was performed before determining the optimum number of clusters using the elbow method. For each representative frame, protein-ligand interaction profiler (PLIP) was used to detect the number and types of interactions [16].

Binding free energy calculation using MM-GBSA:

gmx_MMPBSA package was used to calculate the binding free energy using the Molecular Mechanics Generalized Born Surface Area (MM-GBSA) end-state method with decomposition analysis to get the binding energies of amino acids within 10 Å around the ligand [17-19] The salt concentration and the method of solvation (igb) were set to 0.154 M and 5, respectively. The internal and external dielectric constants were set to 1.0 and 80.0, respectively, and other options were set as default. MM-GBSA approach is depicted in Equation 1

$\Delta G = < Gcomplex - Greceptor - Gligand >$ ……………………………………………… Equation 1

Where < > represents the average of the enclosed free energies of complex, receptor, and ligand over the frames used in the calculation. In our approach, we used the whole trajectory (a total of 1000 frames). Different energy terms can be calculated according to Equations 2 to 6 as follows:

$\Delta Gbinding = \Delta H - T\Delta S$ ……………………………………………………….. **Equation 2**

$\Delta H = \Delta Egas + \Delta Esol$ ……………………………………………………….. **Equation 3**

$\Delta Egas = \Delta Eele + \Delta EvdW$ ……………………………………………………… **Equation 4**

$\Delta Esolv = EGB + ESA$ ……………………………………………………….. **Equation 5**

$ESA = \gamma.SASA$ ……………………………………………………….. **Equation 6**

Where:

ΔH is the enthalpy which can be calculated from gas-phase energy (E_{gas}) and solvation-free energy (E_{sol}). $T\Delta S$ is the entropy contribution to the free binding energy. E_{gas} is composed of electrostatic and van der Waals terms; E_{ele}, E_{vdW}, respectively. E_{sol} can be calculated from the polar solvation energy (E_{GB}) and nonpolar solvation energy (E_{SA}) which is estimated from the solvent accessible surface area [20, 21].

References

[1] T. Mosmann, Rapid colorimetric assay for cellular growth and survival: application to proliferation and cytotoxicity assays, Journal of immunological methods 65(1-2) (1983) 55-63.

[2] F. Denizot, R. Lang, Rapid colorimetric assay for cell growth and survival: modifications to the tetrazolium dye procedure giving improved sensitivity and reliability, Journal of immunological methods 89(2) (1986) 271-277.

[3] M.I. Thabrew, R.D. HUGHES, I.G. MCFARLANE, Screening of hepatoprotective plant components using a HepG2 cell cytotoxicity assay, Journal of pharmacy and pharmacology 49(11) (1997) 1132-1135.

[4] M.-K. Ibrahim, A.A. Abd-Elrahman, R.R. Ayyad, K. El-Adl, A.M. Mansour, I.H. Eissa, Design and synthesis of some novel 2-(3-methyl-2-oxoquinoxalin-1 (2H)-yl)-N-(4-(substituted) phenyl) acetamide derivatives for biological evaluation as anticonvulsant agents, Bulletin of Faculty of Pharmacy, Cairo University 51(1) (2013) 101-111.

[5] N.S. Burres, A. Frigo, R.R. Rasmussen, J.B. McAlpine, A colorimetric microassay for the detection of agents that interact with DNA, Journal of natural products 55(11) (1992) 1582-1587.

[6] B.R. Brooks, C.L. Brooks III, A.D. Mackerell Jr, L. Nilsson, R.J. Petrella, B. Roux, Y. Won, G. Archontis, C. Bartels, S.J.J.o.c.c. Boresch, CHARMM: the biomolecular simulation program, 30(10) (2009) 1545-1614.

[7] S. Jo, X. Cheng, S.M. Islam, L. Huang, H. Rui, A. Zhu, H.S. Lee, Y. Qi, W. Han, K.J.A.i.p.c. Vanommeslaeghe, s. biology, CHARMM-GUI PDB manipulator for advanced modeling and simulations of proteins containing nonstandard residues, 96 (2014) 235-265.

[8] S. Jo, T. Kim, V.G. Iyer, W.J.J.o.c.c. Im, CHARMM-GUI: a web-based graphical user interface for CHARMM, 29(11) (2008) 1859-1865.

[9] J. Lee, X. Cheng, J.M. Swails, M.S. Yeom, P.K. Eastman, J.A. Lemkul, S. Wei, J. Buckner, J.C. Jeong, Y.J.J.o.c.t. Qi, computation, CHARMM-GUI input generator for NAMD, GROMACS, AMBER, OpenMM, and CHARMM/OpenMM simulations using the CHARMM36 additive force field, 12(1) (2016) 405-413.

[10] M.J. Abraham, T. Murtola, R. Schulz, S. Páll, J.C. Smith, B. Hess, E.J.S. Lindahl, GROMACS: High performance molecular simulations through multi-level parallelism from laptops to supercomputers, 1 (2015) 19-25.

[11] G. Bussi, D. Donadio, M.J.T.J.o.c.p. Parrinello, Canonical sampling through velocity rescaling, 126(1) (2007) 014101.

[12] B. Hess, H. Bekker, H.J. Berendsen, J.G.J.J.o.c.c. Fraaije, LINCS: a linear constraint solver for molecular simulations, 18(12) (1997) 1463-1472.

[13] U. Essmann, L. Perera, M.L. Berkowitz, T. Darden, H. Lee, L.G.J.T.J.o.c.p. Pedersen, A smooth particle mesh Ewald method, 103(19) (1995) 8577-8593.

[14] W. Humphrey, A. Dalke, K.J.J.o.m.g. Schulten, VMD: visual molecular dynamics, 14(1) (1996) 33-38.

[15] T. Tubiana, J.-C. Carvaillo, Y. Boulard, S.J.J.o.c.i. Bressanelli, modeling, TTClust: a versatile molecular simulation trajectory clustering program with graphical summaries, 58(11) (2018) 2178-2182.

[16] S. Salentin, S. Schreiber, V.J. Haupt, M.F. Adasme, M.J.N.a.r. Schroeder, PLIP: fully automated protein–ligand interaction profiler, 43(W1) (2015) W443-W447.

[17] V. Ekberg, U.J.J.o.c.t. Ryde, computation, On the use of interaction entropy and related methods to estimate binding entropies, 17(8) (2021) 5379-5391.

[18] M.S. Valdés-Tresanco, M.E. Valdés-Tresanco, P.A. Valiente, E.J.J.o.C.T. Moreno, Computation, gmx_MMPBSA: a new tool to perform end-state free energy calculations with GROMACS, 17(10) (2021) 6281-6291.

[19] B.R. Miller III, T.D. McGee Jr, J.M. Swails, N. Homeyer, H. Gohlke, A.E.J.J.o.c.t. Roitberg, computation, MMPBSA. py: an efficient program for end-state free energy calculations, 8(9) (2012) 3314-3321.

[20] W. Xue, F. Yang, P. Wang, G. Zheng, Y. Chen, X. Yao, F.J.A.C.N. Zhu, What contributes to serotonin–norepinephrine reuptake inhibitors' dual-targeting mechanism? The key role of transmembrane domain 6 in human serotonin and norepinephrine transporters revealed by molecular dynamics simulation, 9(5) (2018) 1128-1140.

[21] T.J.E.o.o.d.d. Tuccinardi, What is the current value of MM/PBSA and MM/GBSA methods in drug discovery?, Taylor & Francis, 2021, pp. 1233-1237.

Methyl green

Best-fit values	
LogIC50	1.557
HillSlope	0.4353
IC50	36.07

13a

IC_{50} = 36.07
R^2 = 0.7640

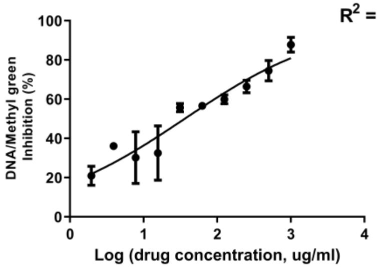

Best-fit values	
LogIC50	1.482
HillSlope	0.5366
IC50	30.37

14a

IC_{50} = 30.37
R^2 = 0.9207

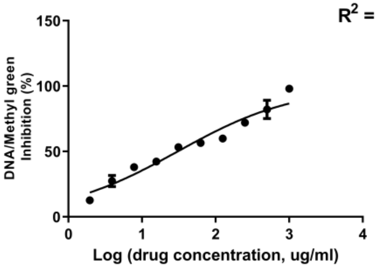

Best-fit values	
LogIC50	1.181
HillSlope	0.5304
IC50	15.16

16

IC_{50} = 15.16
R^2 = 0.8861

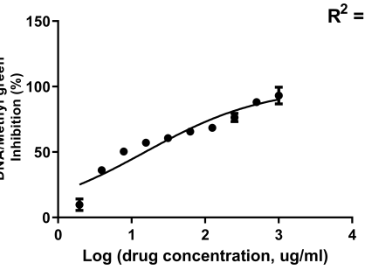

Best-fit values	
LogIC50	1.247
HillSlope	0.5610
IC50	17.66

17

IC_{50} = 17.66
R^2 = 0.9231

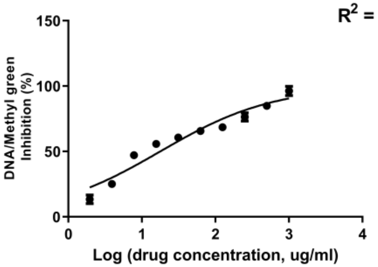

Best-fit values	
LogIC50	1.262
HillSlope	0.3412
IC50	18.28

18

IC_{50} = 18.28
R^2 = 0.8717

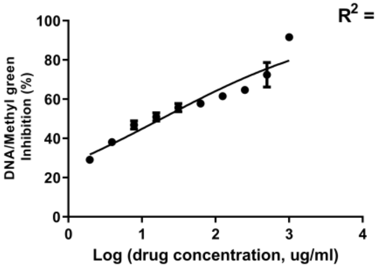

Best-fit values	
LogIC50	1.271
HillSlope	0.3896
IC50	18.66

19

IC_{50} = 18.66
R^2 = 0.8643

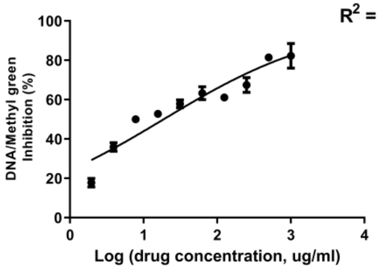

Best-fit values	
LogIC50	1.315
HillSlope	0.4183
IC50	20.65

20

IC_{50} = 20.65
R^2 = 0.8884

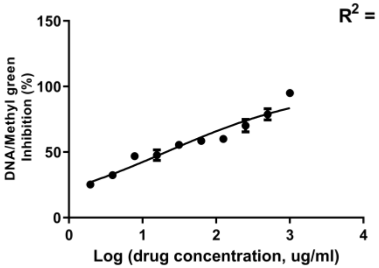

Best-fit values	
LogIC50	0.9158
HillSlope	0.4481
IC50	8.238

Doxorubicin

IC_{50} = 8.238
R^2 = 0.8430

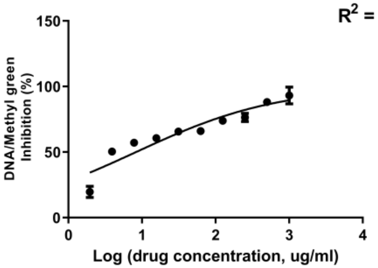

Topoisomerase II

Best-fit values	
LogIC50	1.723
HillSlope	0.4587
IC50	52.87

13a

IC_{50} = 52.87

R^2 = 0.9875

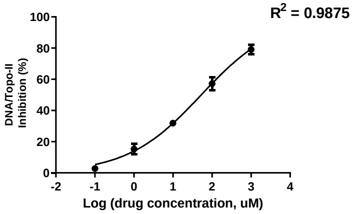

Best-fit values	
LogIC50	1.353
HillSlope	0.4583
IC50	22.54

14a

IC_{50} = 22.54

R^2 = 0.9663

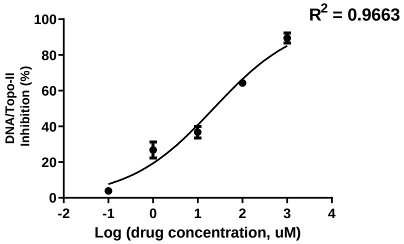

Best-fit values	
LogIC50	1.045
HillSlope	0.4423
IC50	11.09

17

IC_{50} = 11.09

R^2 = 0.9685

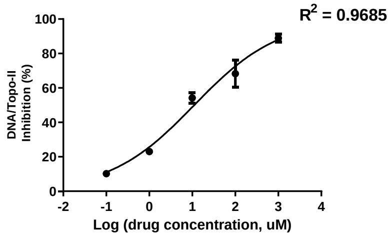

Best-fit values	
LogIC50	1.011
HillSlope	0.3743
IC50	10.25

16

IC_{50} = 10.25

R^2 = 0.9341

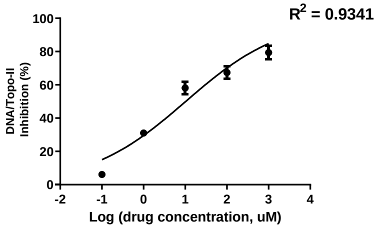

Best-fit values	
LogIC50	1.098
HillSlope	0.3961
IC50	12.54

18

IC_{50} = 12.54

R^2 = 0.9579

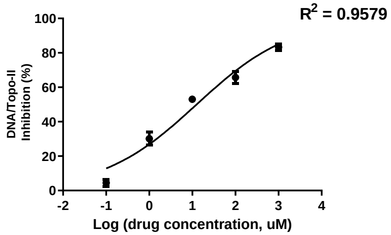

Best-fit values	
LogIC50	1.373
HillSlope	0.3612
IC50	23.58

19

IC_{50} = 23.58

R^2 = 0.9595

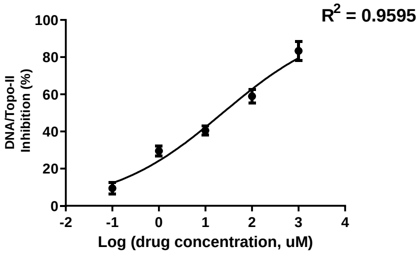

Best-fit values	
LogIC50	1.538
HillSlope	0.3425
IC50	34.51

20

IC_{50} = 34.51

R^2 = 0.9444

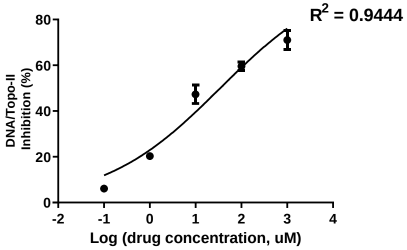

Best-fit values	
LogIC50	0.7515
HillSlope	0.3659
IC50	5.643

Doxorubicin

IC_{50} = 5.643

R^2 = 0.9499

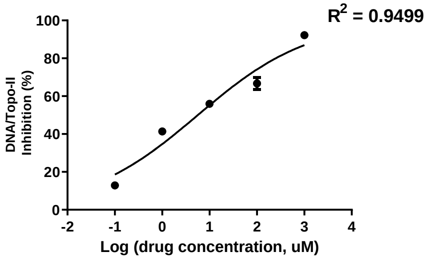

IR of compound **11a**

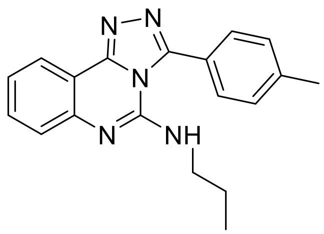

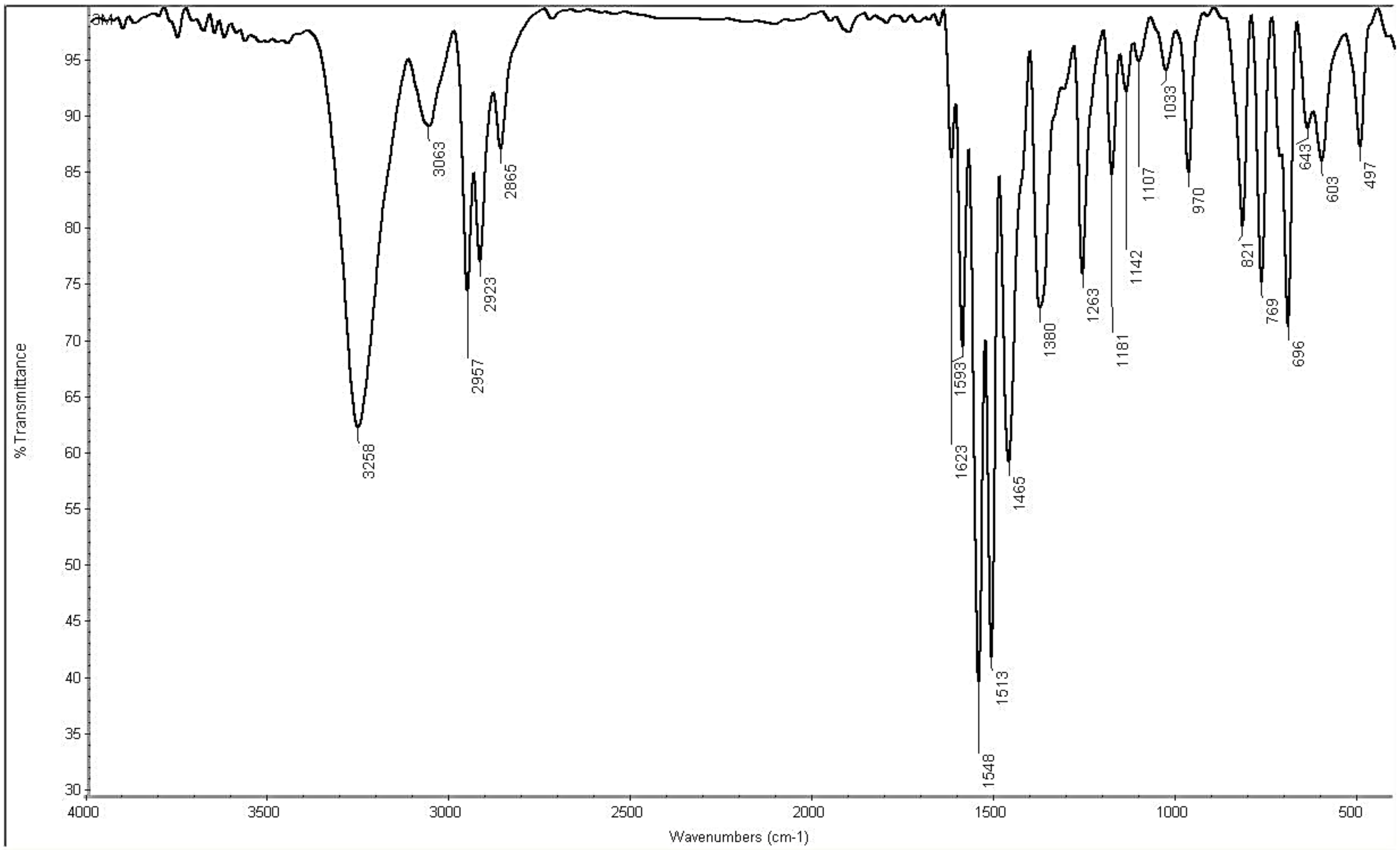

IR of compound **11b**

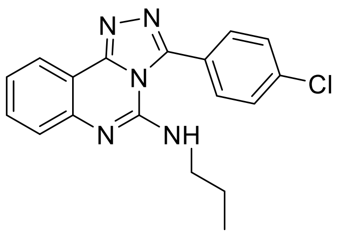

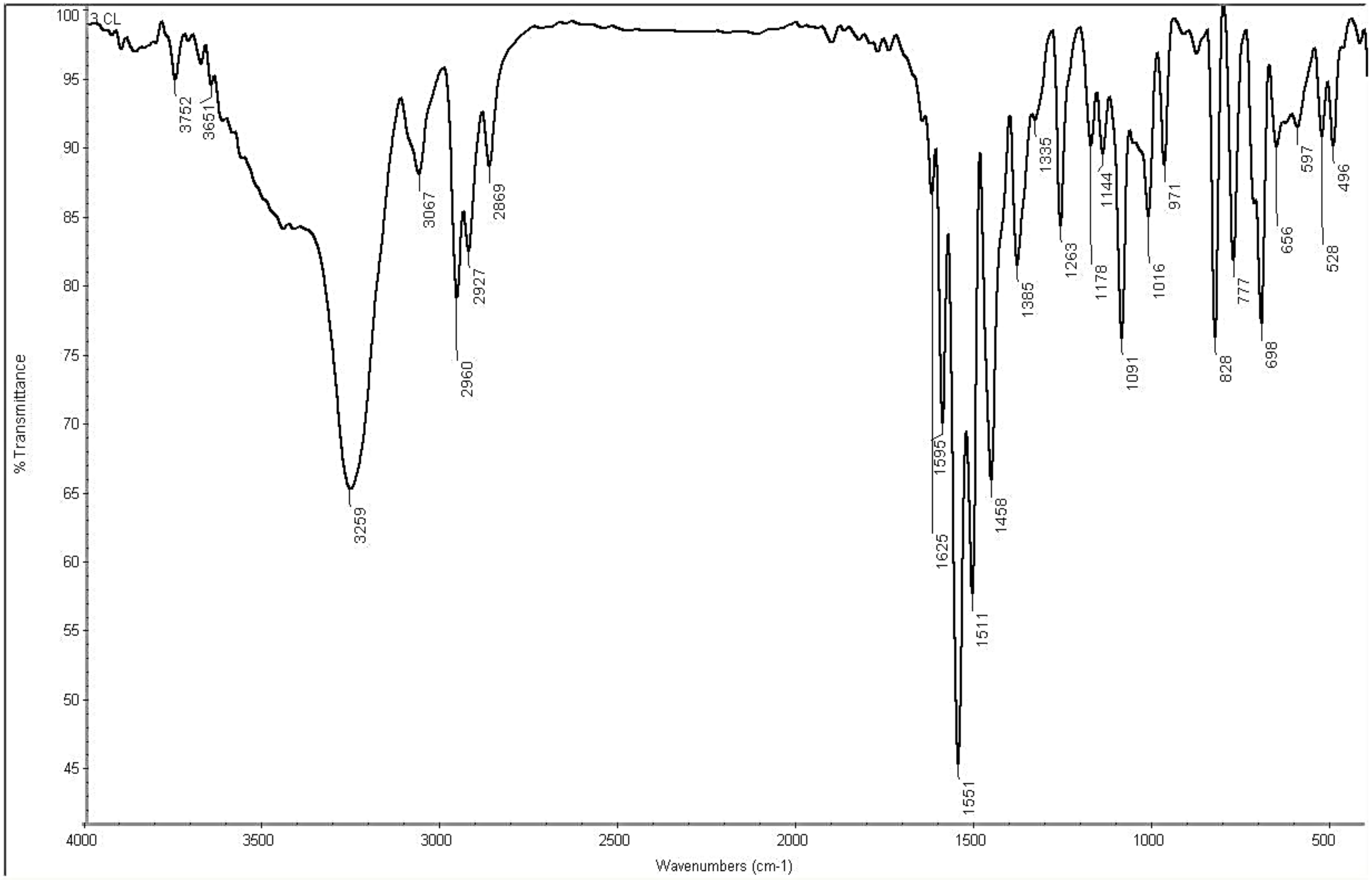

IR of compound **12a**

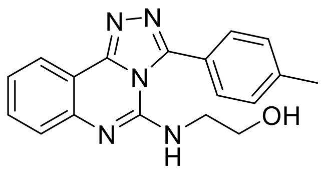

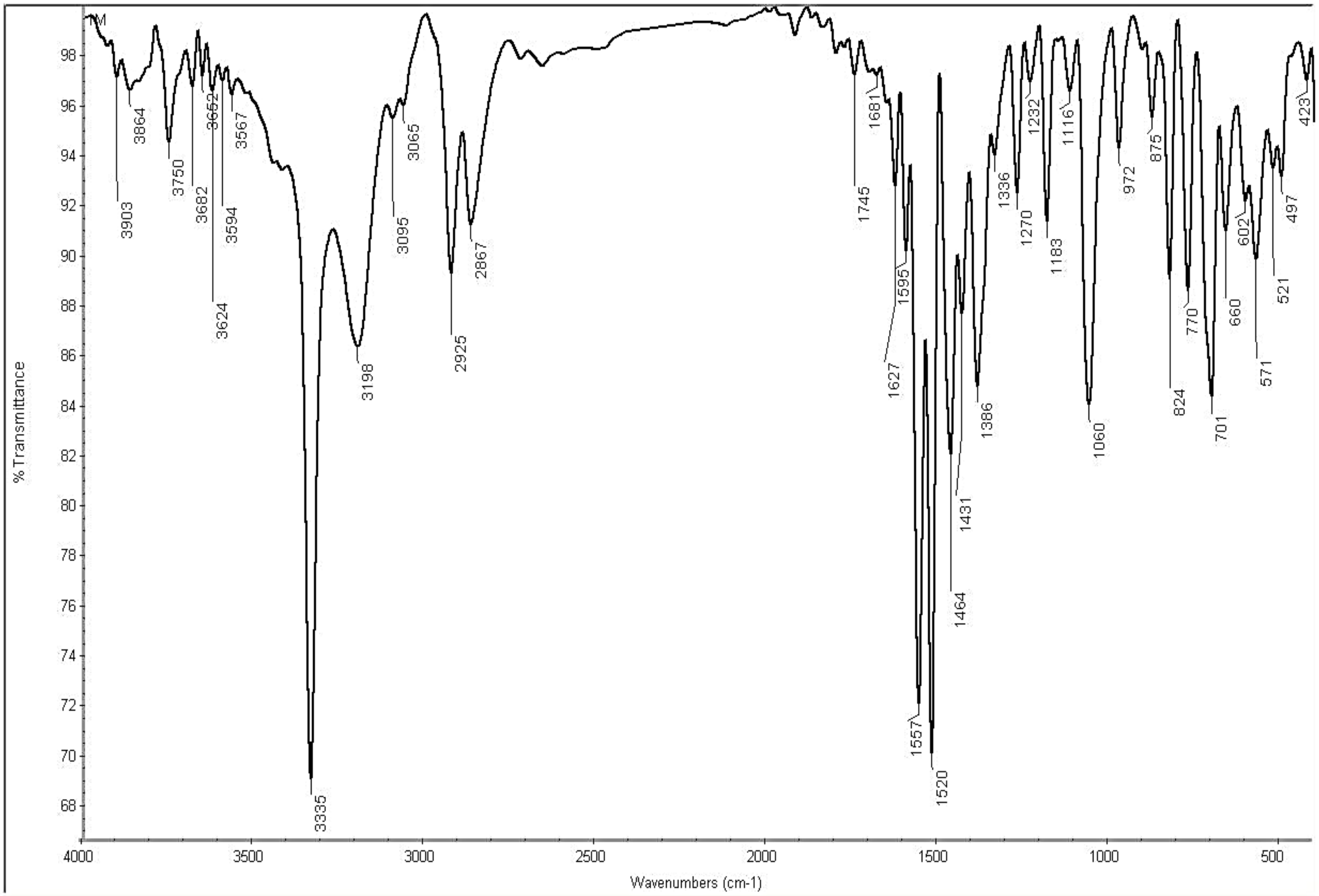

IR of compound **12b**

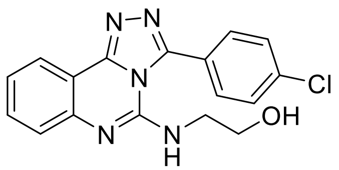

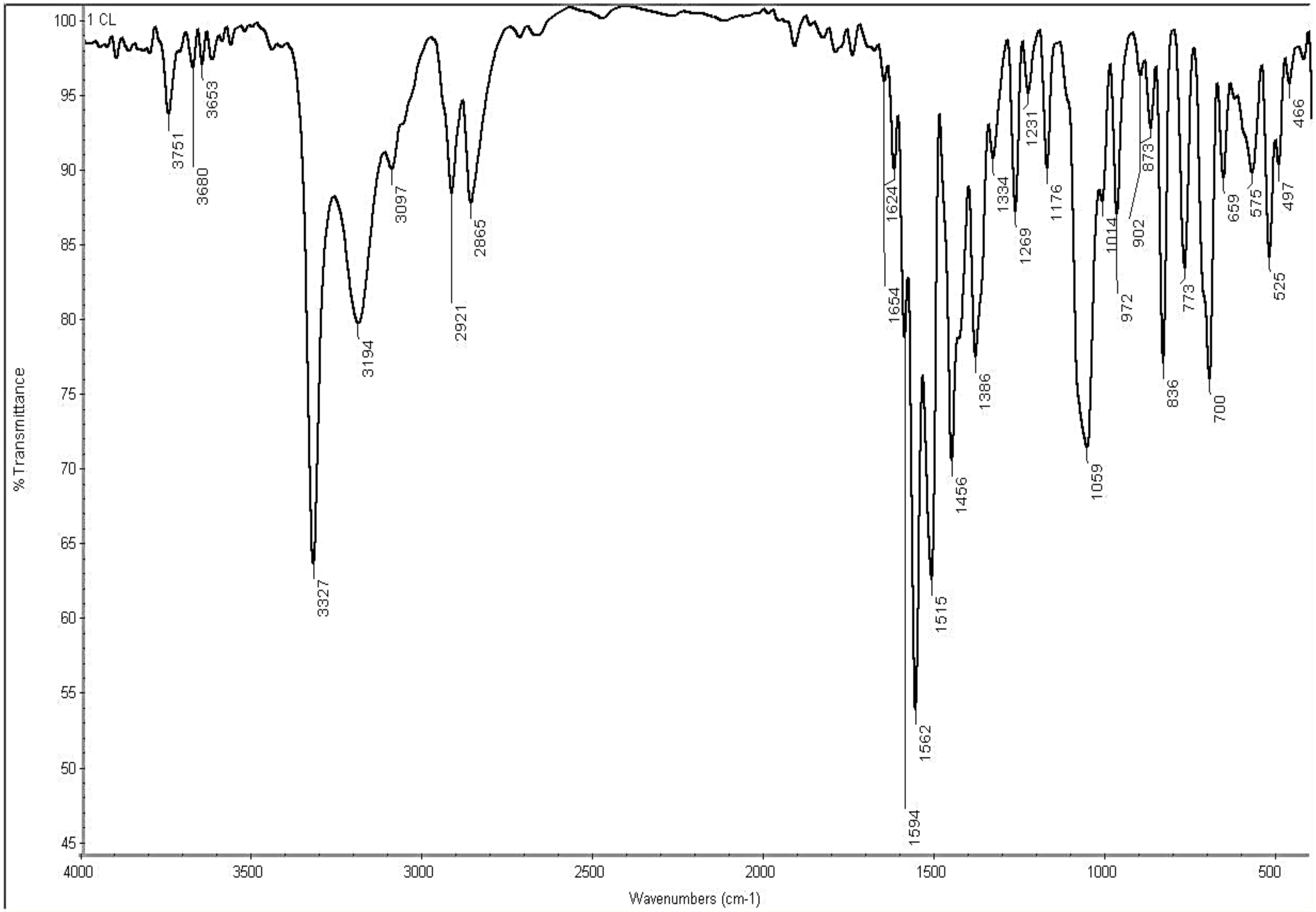

IR of compound **13a**

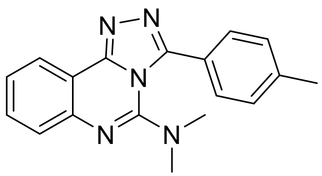

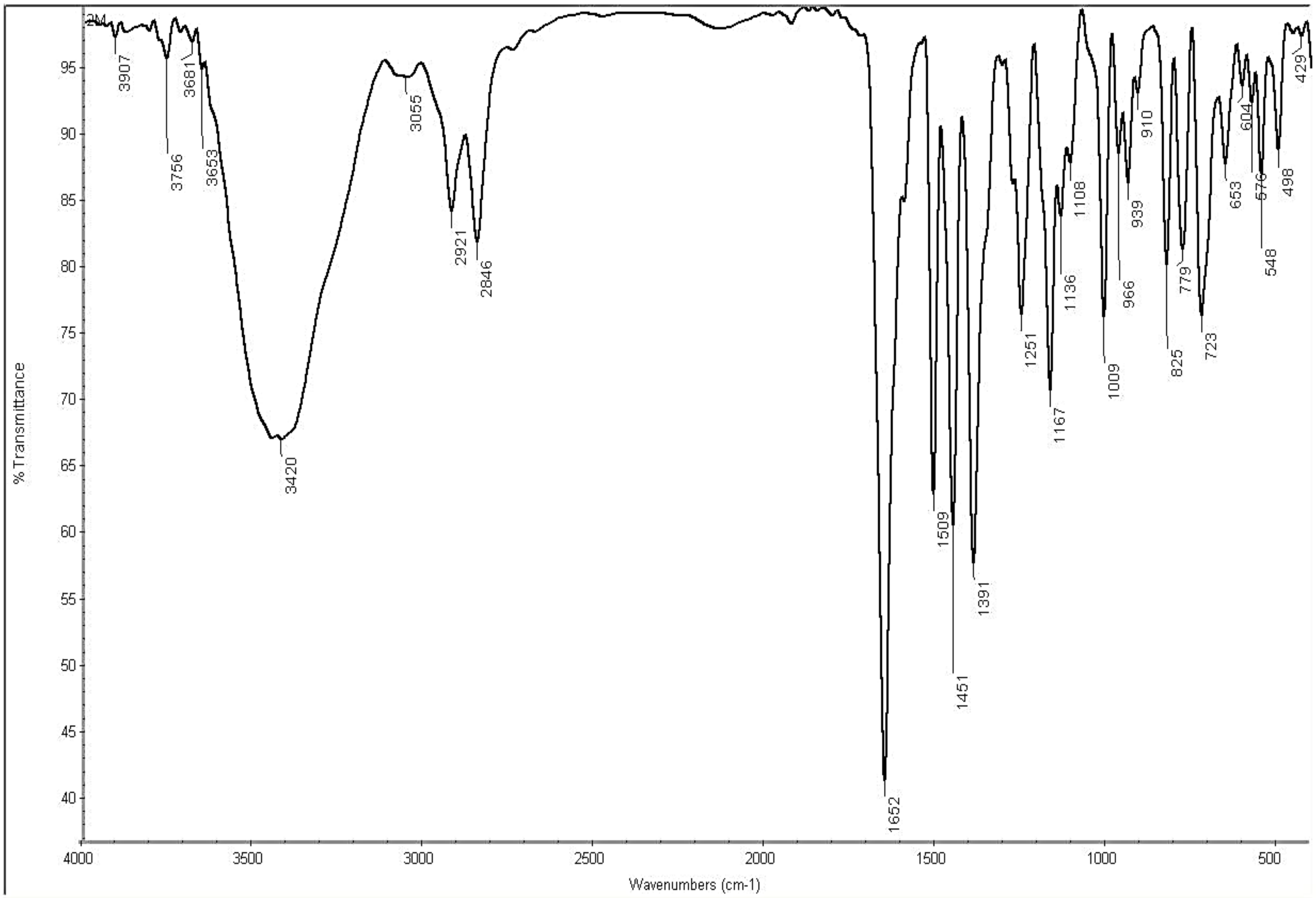

IR of compound **13b**

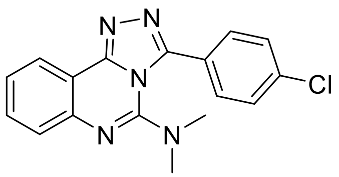

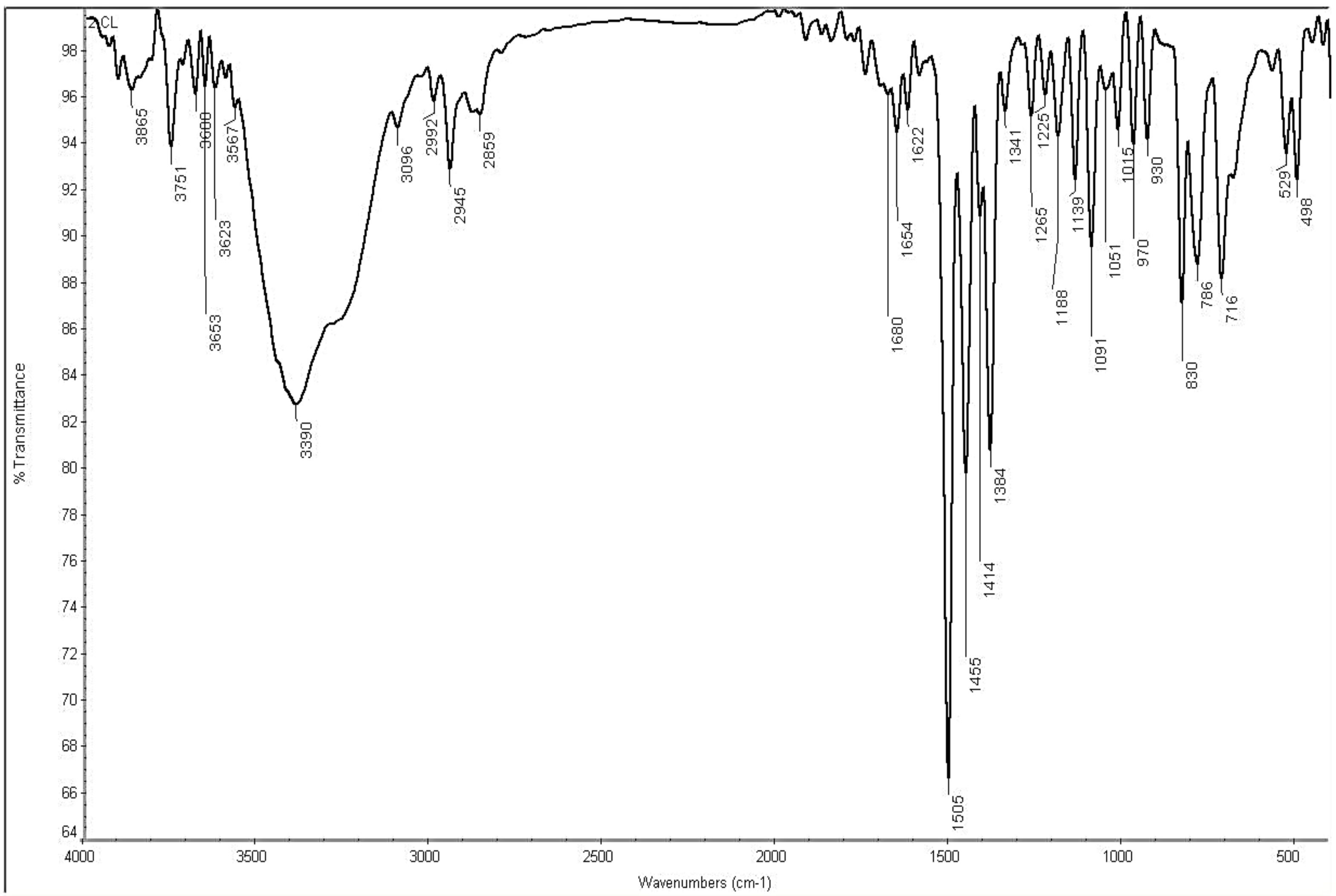

IR of compound **14a**

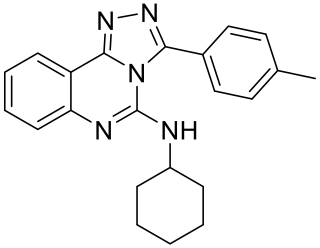

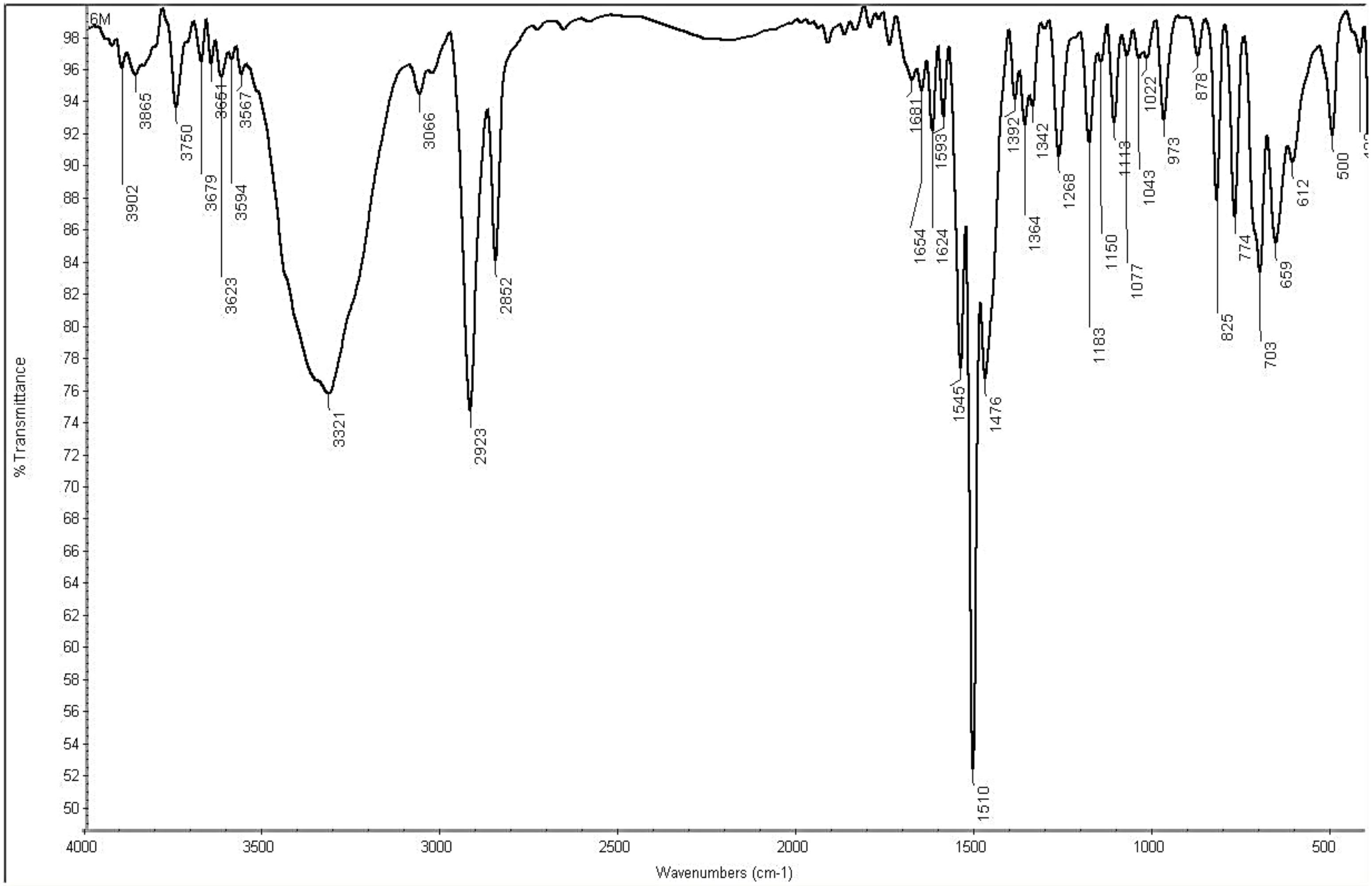

IR of compound **14b**

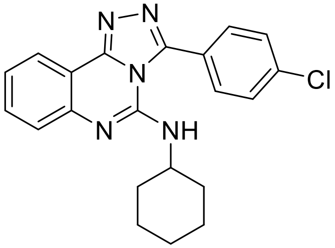

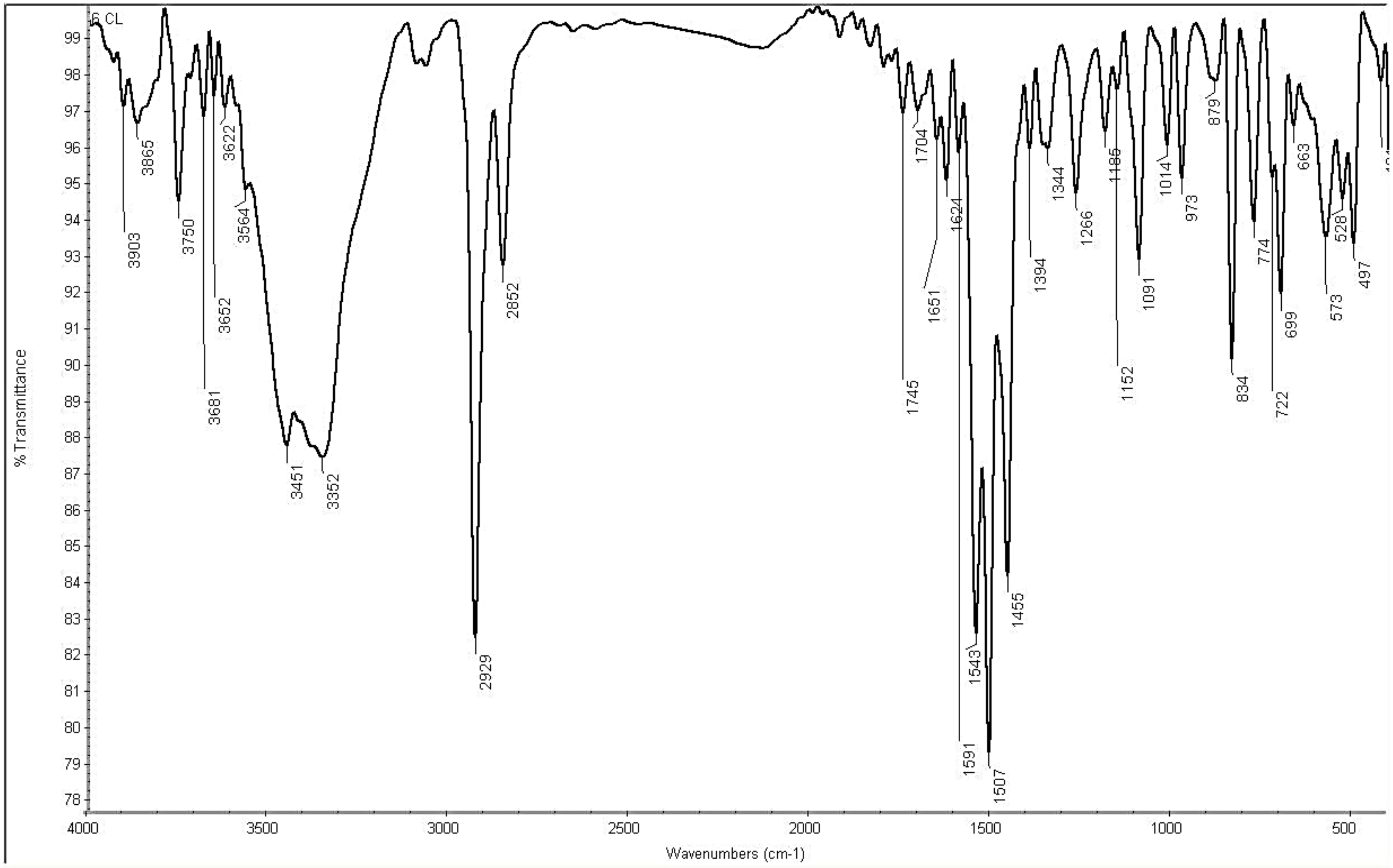

IR of compound **15a**

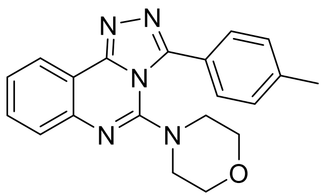

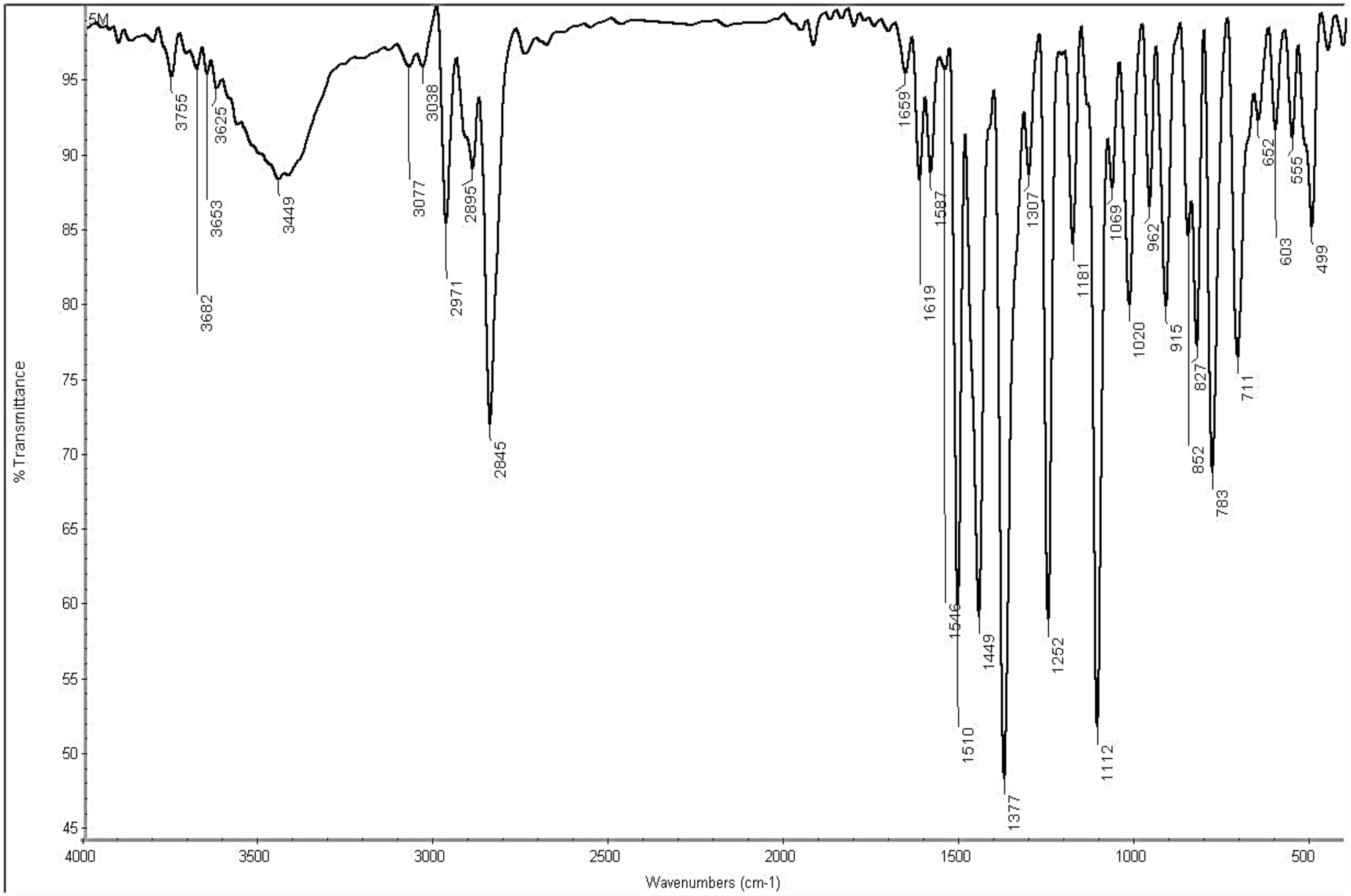

IR of compound **15b**

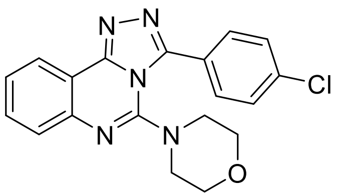

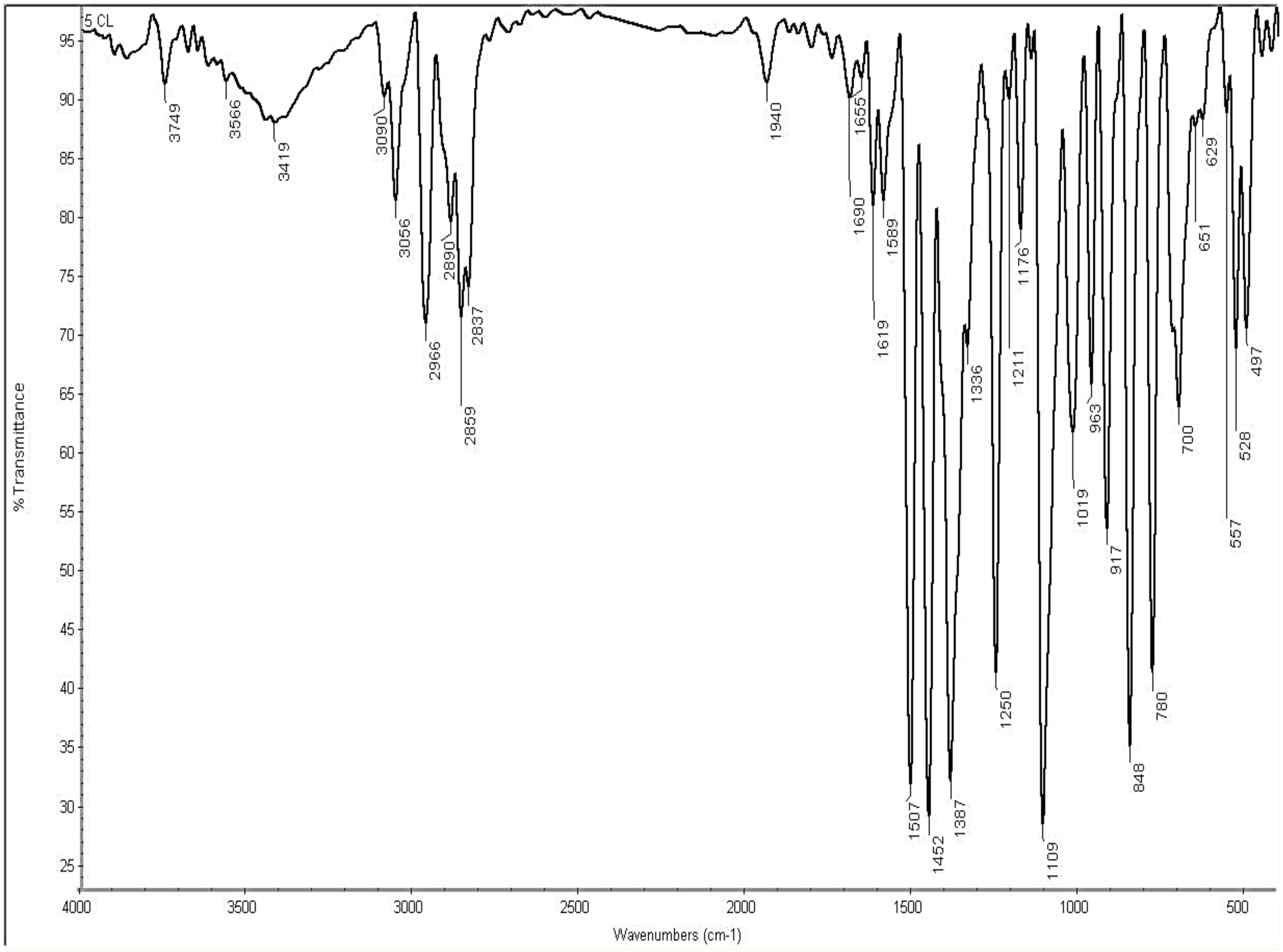

IR of compound **16**

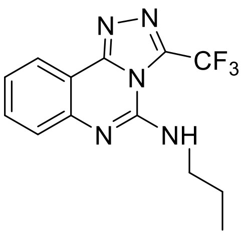

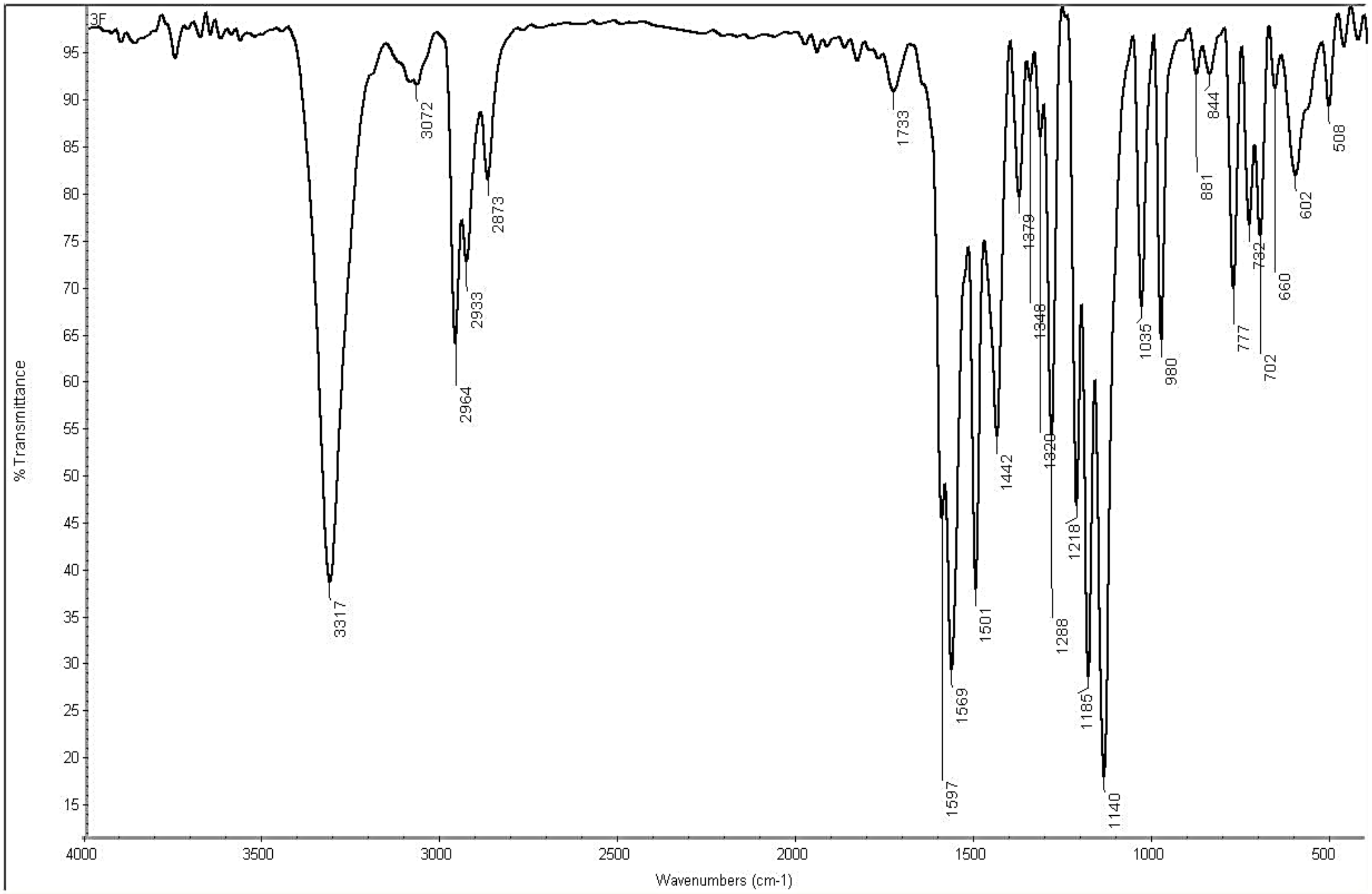

IR of compound **17**

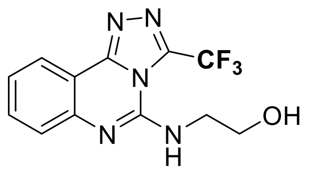

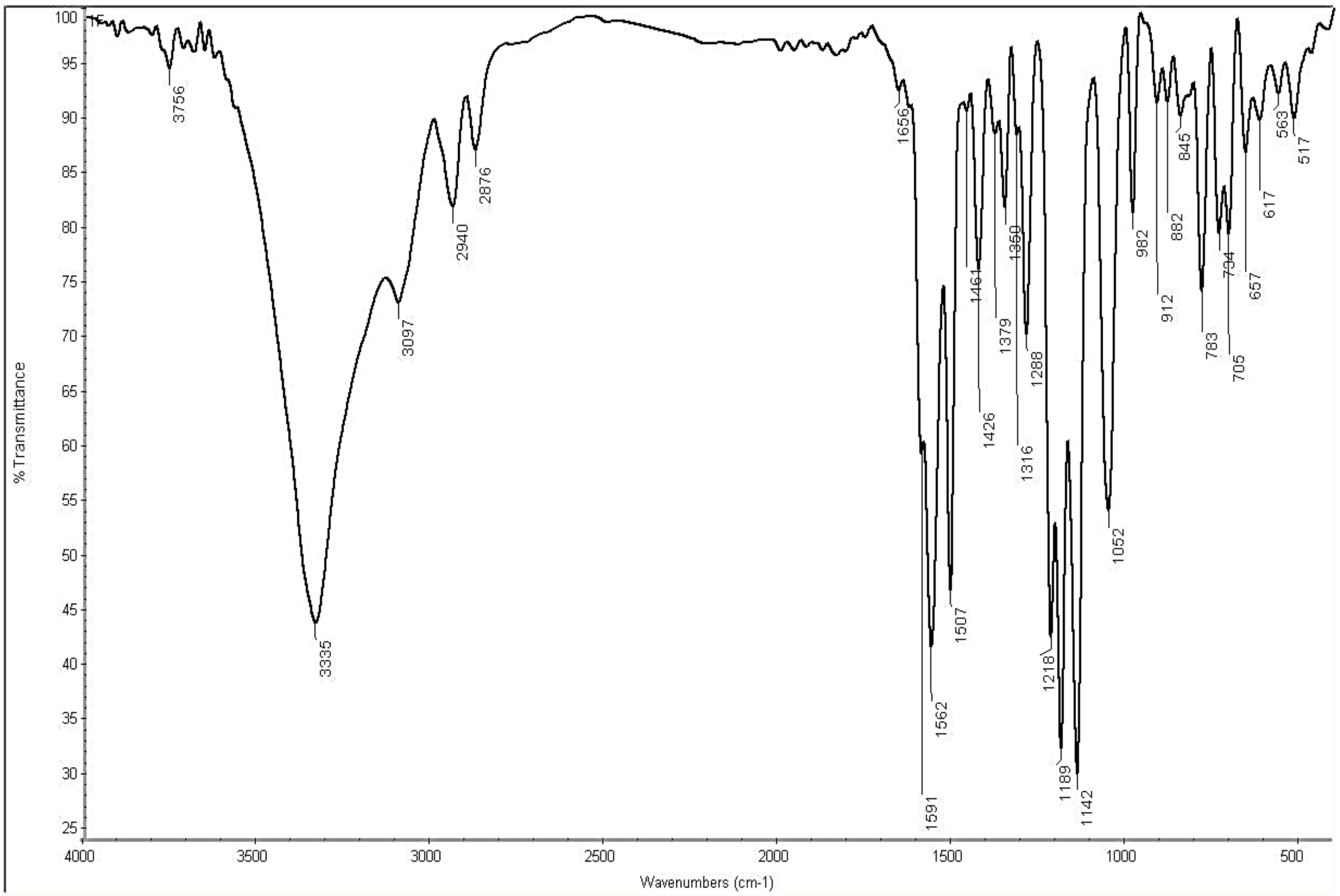

IR of compound **18**

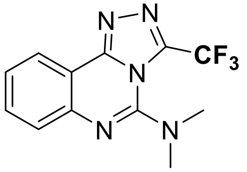

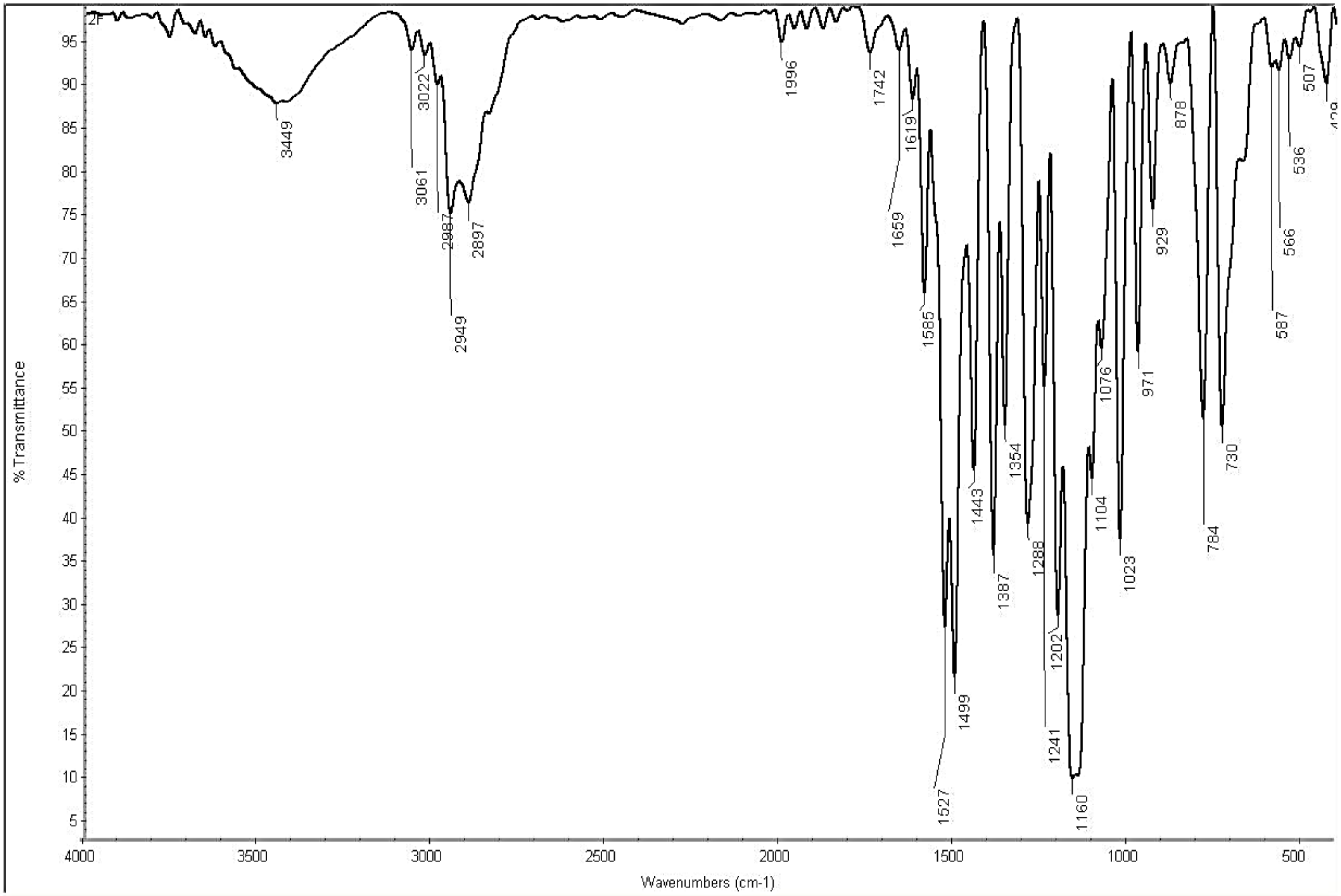

IR of compound **19**

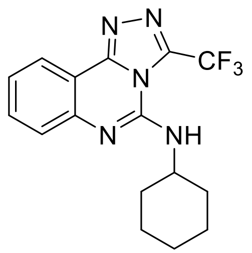

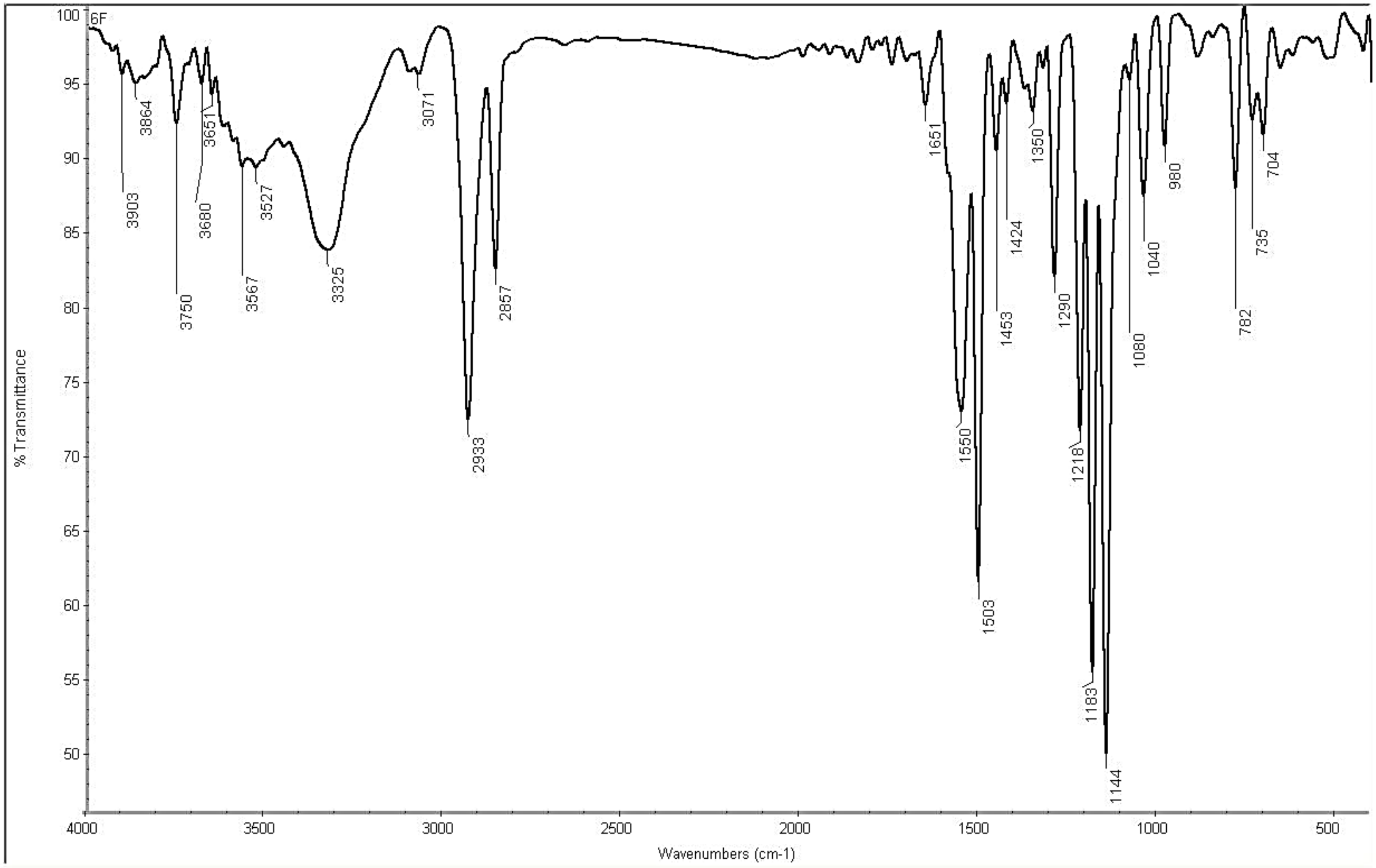

IR of compound **20**

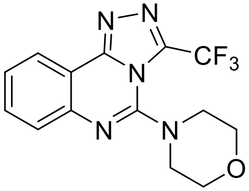

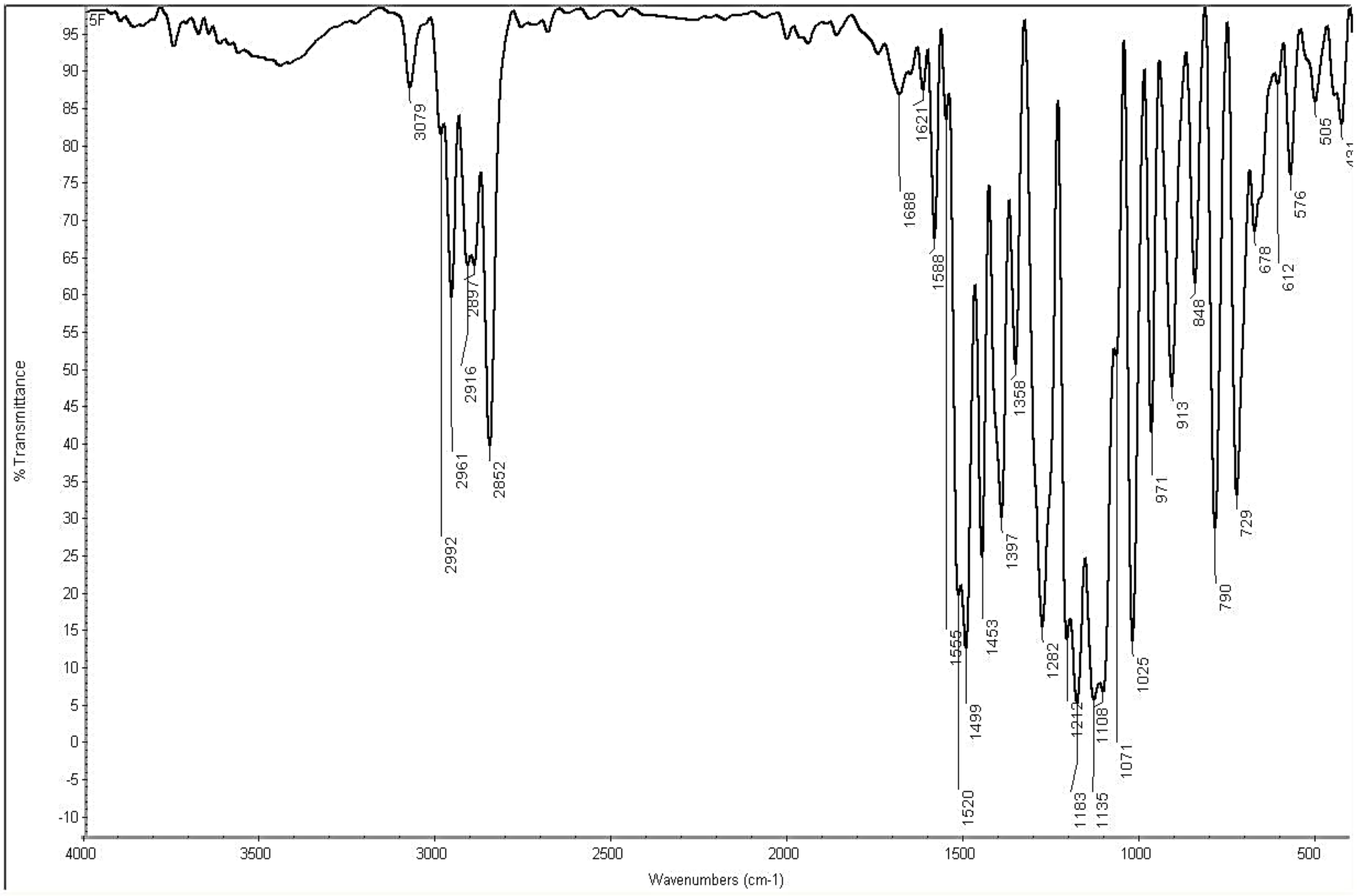

1H NMR of 11a

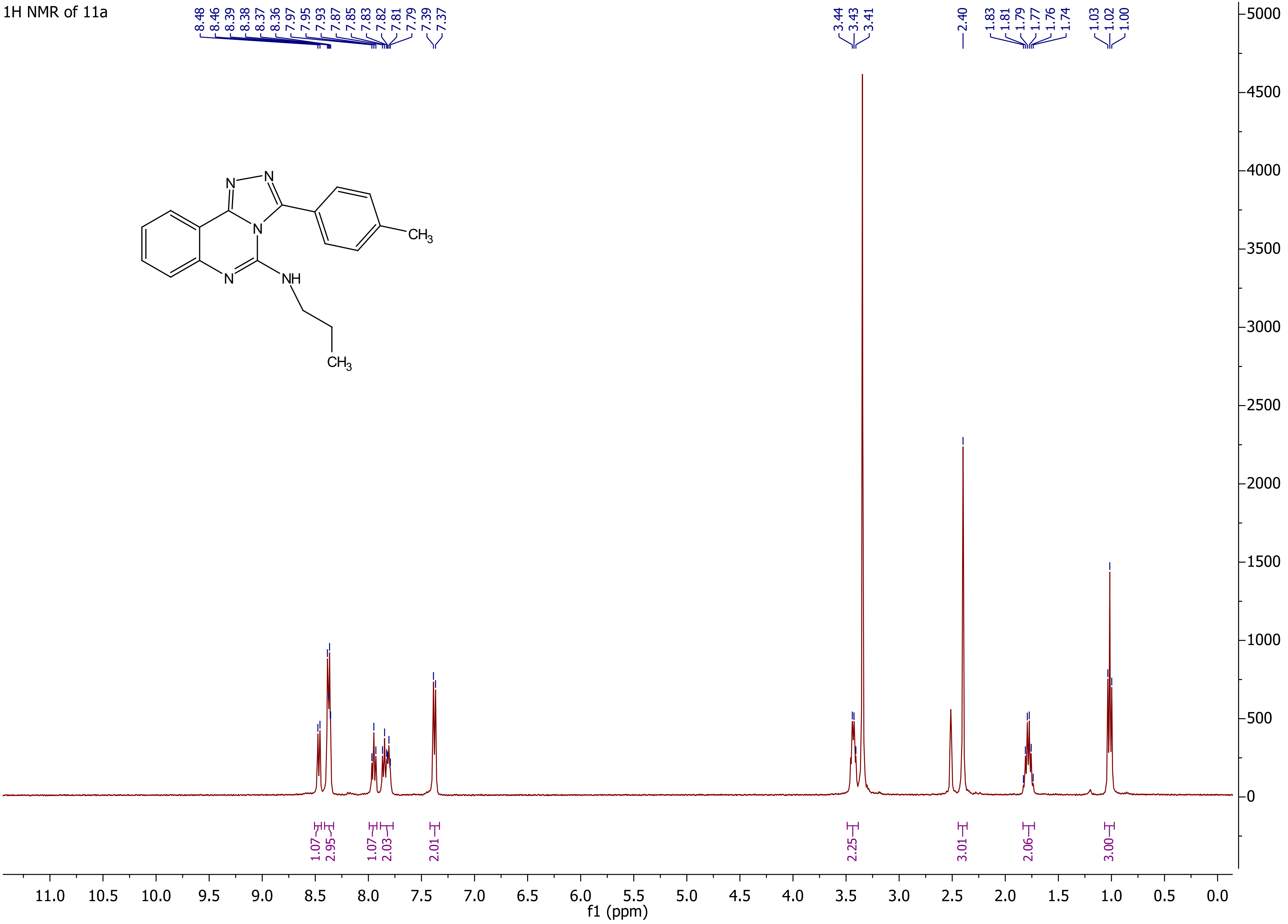

8.48 8.46 8.39 8.38 8.37 8.36 7.97 7.95 7.93 7.87 7.85 7.83 7.82 7.81 7.79 7.39 7.37
3.44 3.43 3.41
2.40
1.83 1.81 1.79 1.77 1.76 1.74
1.03 1.02 1.00
N
N
N
N
NH
CH3
CH3
1.07 2.95 1.07 2.03 2.01 2.25 3.01 2.06 3.00
f1 (ppm)

1H NMR of 11a

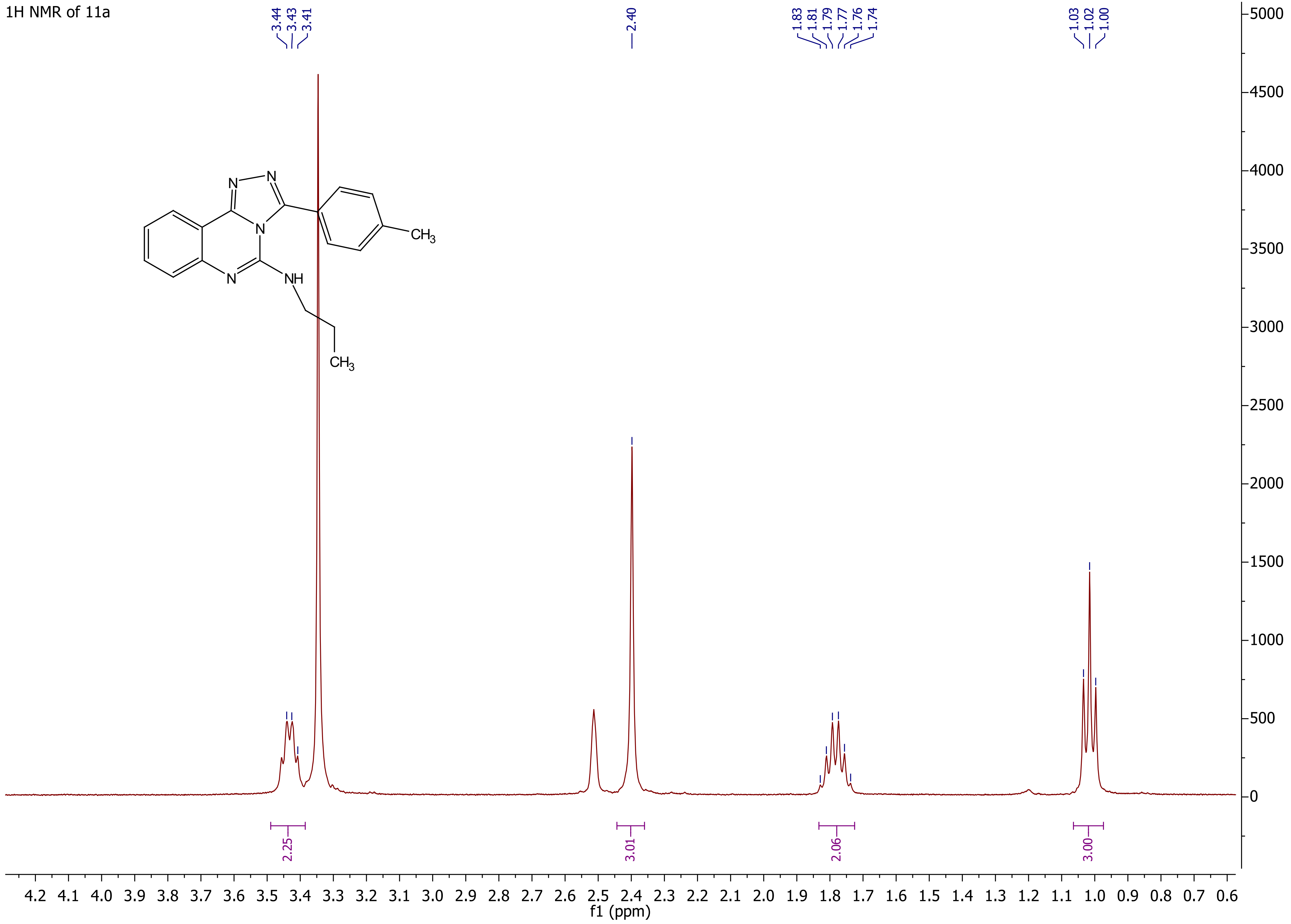

3.44
3.43
3.41
2.40
1.83
1.81
1.79
1.77
1.76
1.74
1.03
1.02
1.00
N
N
N
N
NH
CH3
CH3
2.25
3.01
2.06
3.00
f1 (ppm)

1H NMR of 11a

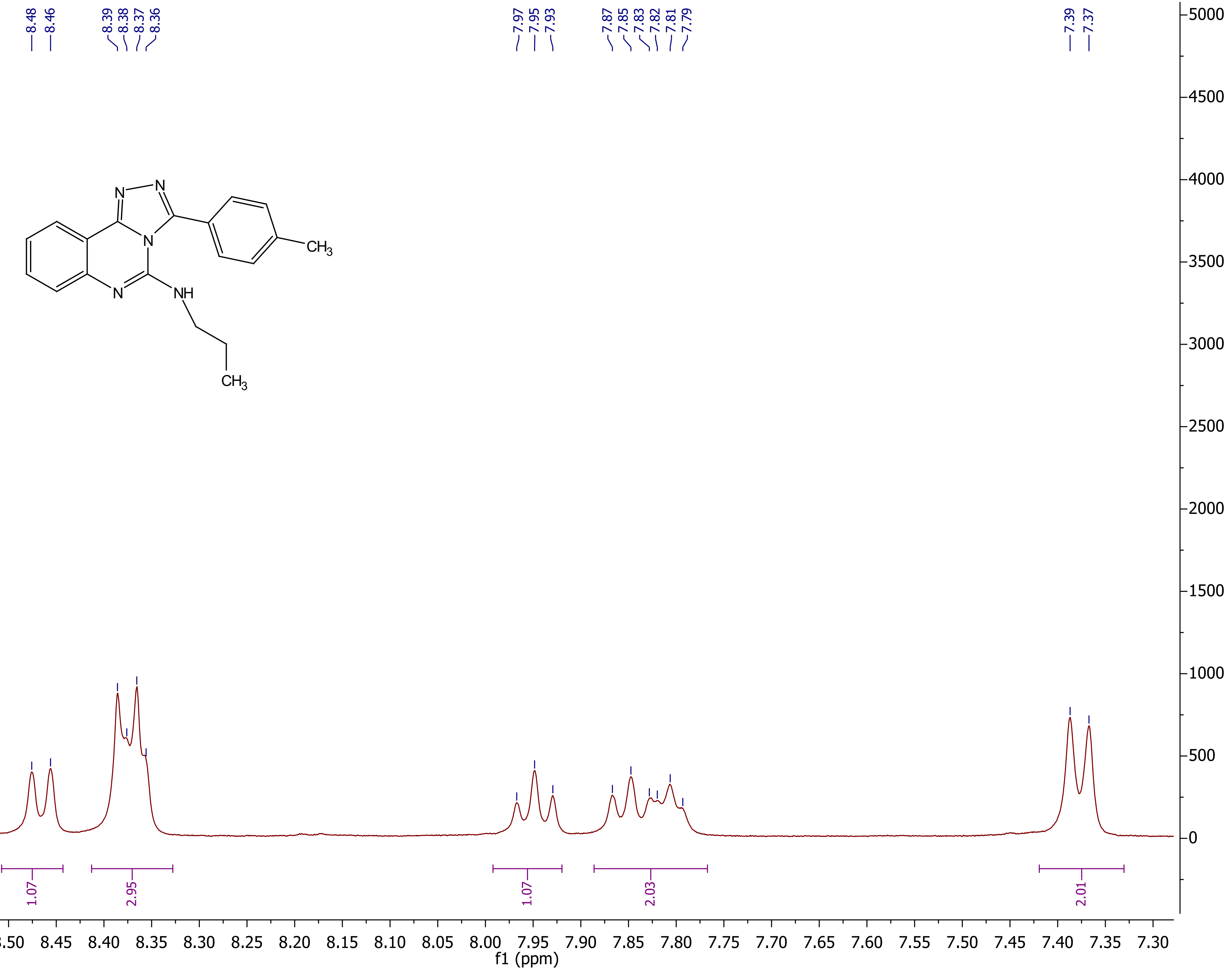

8.48
8.46
8.39
8.38
8.37
8.36
7.97
7.95
7.93
7.87
7.85
7.83
7.82
7.81
7.79
7.39
7.37
N
N
N
N
NH
CH3
CH3
1.07
2.95
1.07
2.03
2.01
5000
4500
4000
3500
3000
2500
2000
1500
1000
500
0
8.60
8.55
8.50
8.45
8.40
8.35
8.30
8.25
8.20
8.15
8.10
8.05
8.00
7.95
7.90
7.85
7.80
7.75
7.70
7.65
7.60
7.55
7.50
7.45
7.40
7.35
7.30
f1 (ppm)

1H NMR of 12a

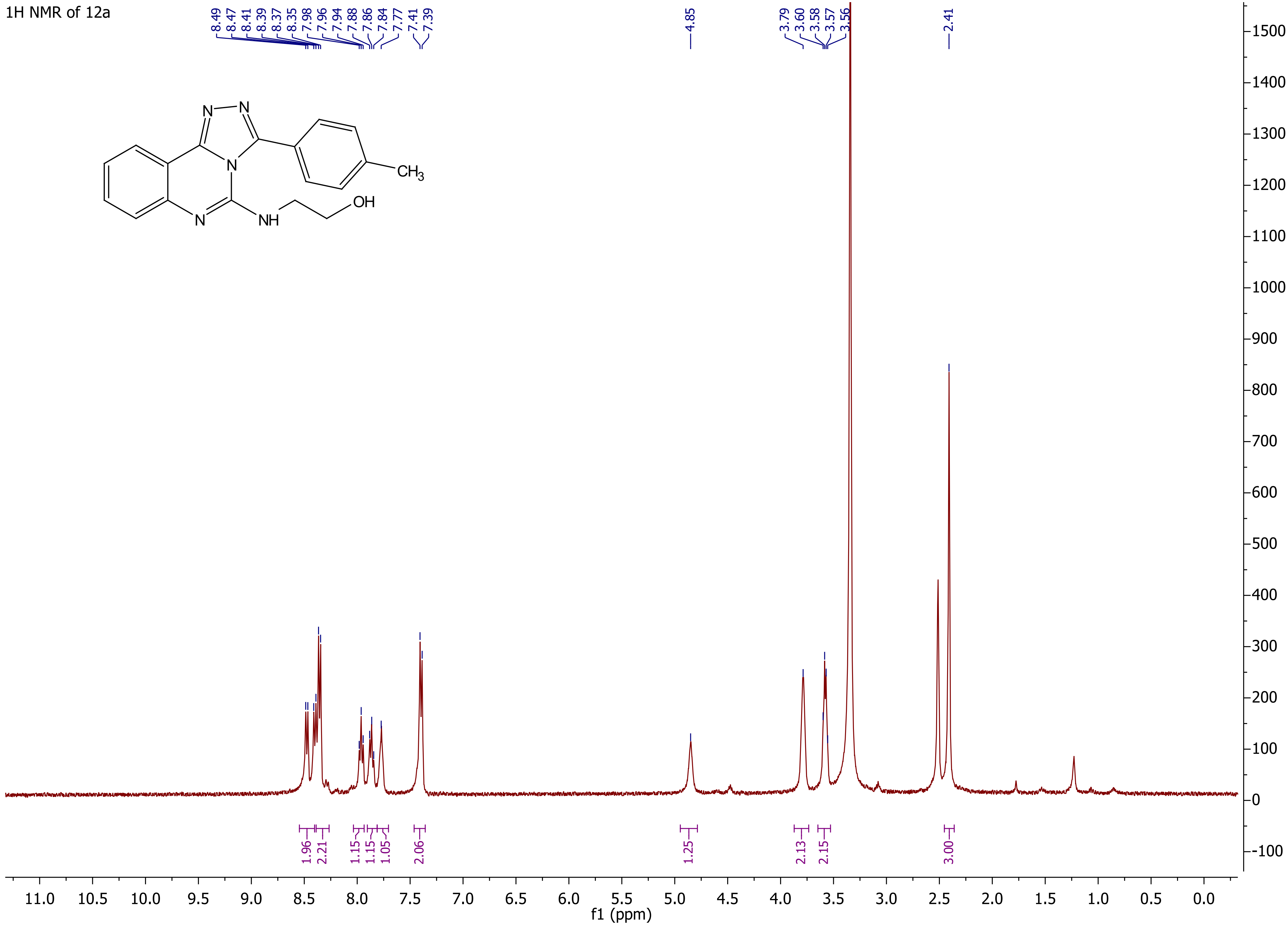
8.49
8.47
8.41
8.39
8.37
8.35
7.98
7.96
7.94
7.88
7.86
7.84
7.77
7.41
7.39
4.85
3.79
3.60
3.58
3.57
3.56
2.41
N
N
N
N
CH3
NH
OH
1.96
2.21
1.15
1.15
1.05
2.06
1.25
2.13
2.15
3.00
f1 (ppm)

1H NMR of 12a

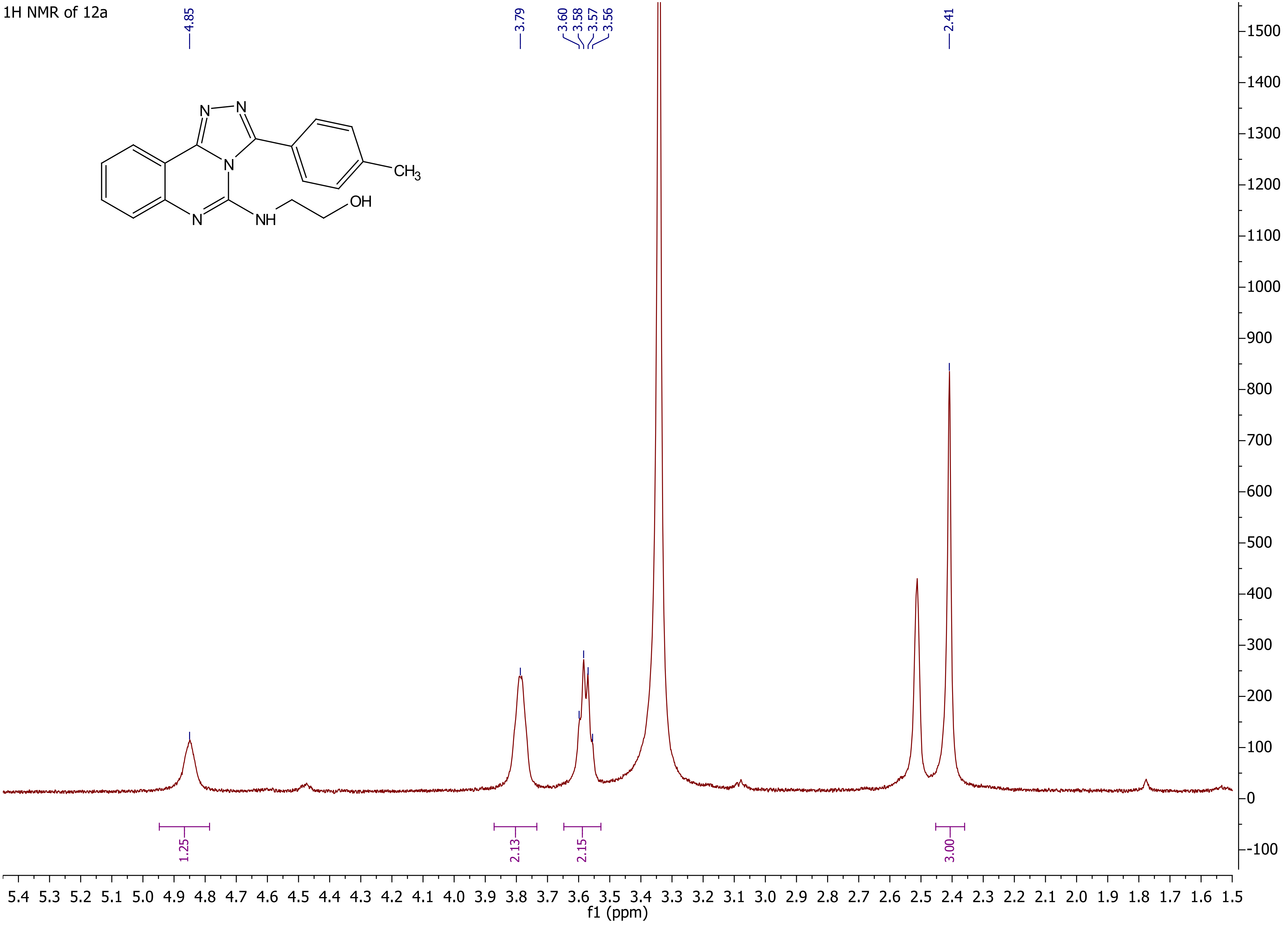
4.85
3.79
3.60
3.58
3.57
3.56
2.41
N
N
N
N
NH
OH
CH3
1.25
2.13
2.15
3.00
5.4 5.3 5.2 5.1 5.0 4.9 4.8 4.7 4.6 4.5 4.4 4.3 4.2 4.1 4.0 3.9 3.8 3.7 3.6 3.5 3.4 3.3 3.2 3.1 3.0 2.9 2.8 2.7 2.6 2.5 2.4 2.3 2.2 2.1 2.0 1.9 1.8 1.7 1.6 1.5
f1 (ppm)
1500
1400
1300
1200
1100
1000
900
800
700
600
500
400
300
200
100
0
-100

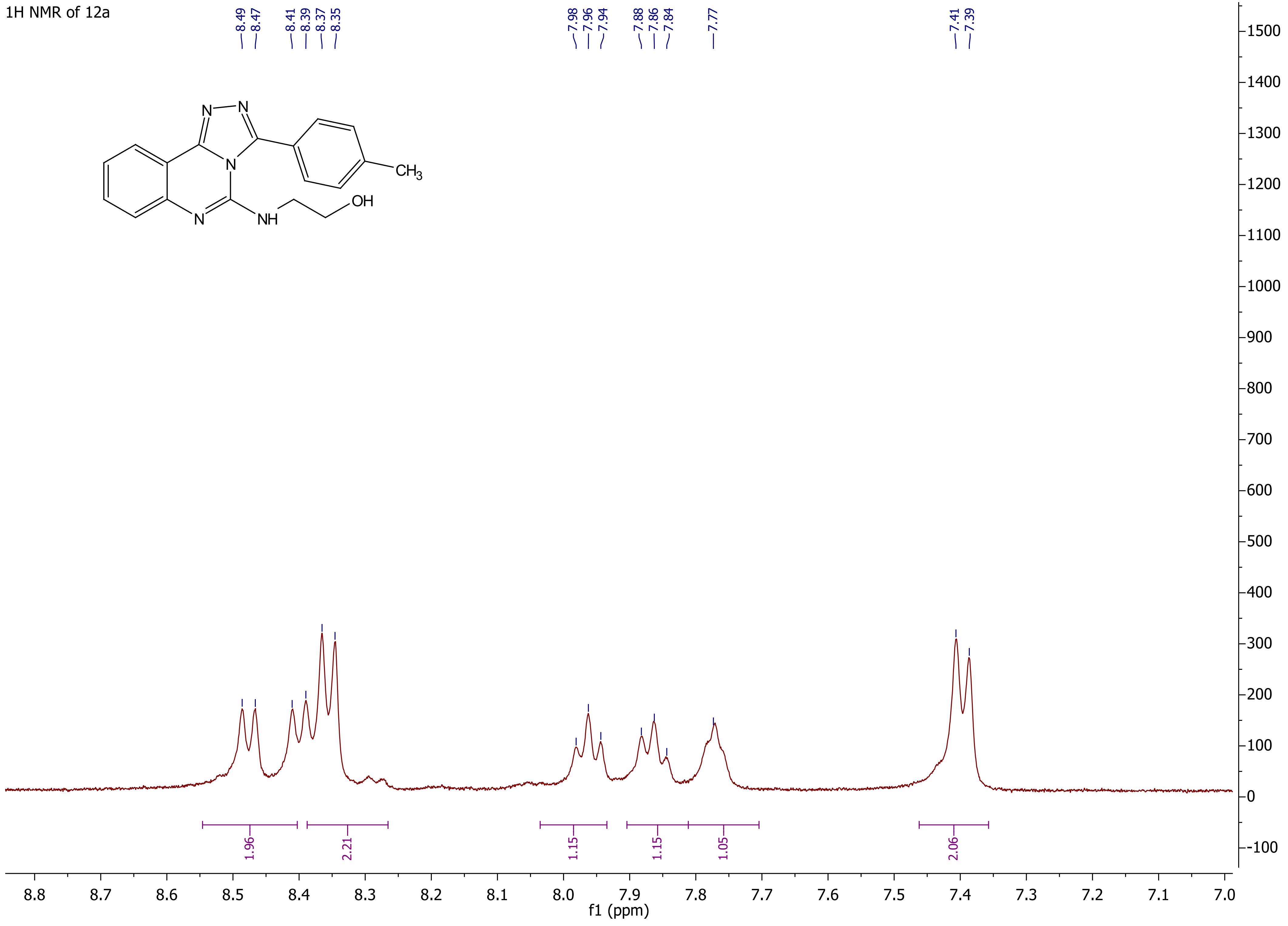
1H NMR of 12a
8.49
8.47
8.41
8.39
8.37
8.35
7.98
7.96
7.94
7.88
7.86
7.84
7.77
7.41
7.39
N
N
N
N
NH
OH
CH3
1.96
2.21
1.15
1.15
1.05
2.06
f1 (ppm)

1H NMR of 12b

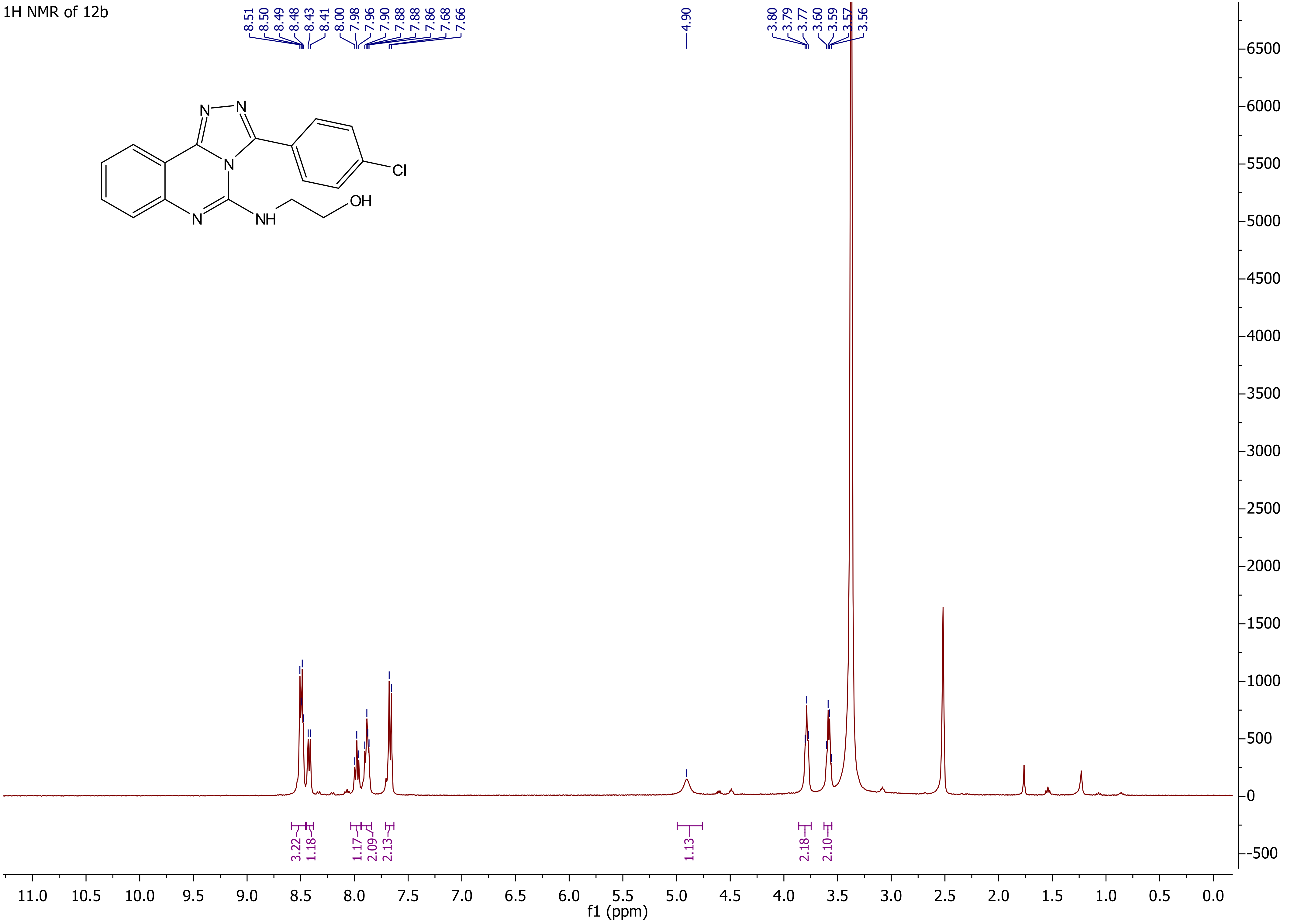
8.51 8.50 8.49 8.48 8.43 8.41 8.00 7.98 7.96 7.90 7.88 7.88 7.86 7.68 7.66
4.90
3.80 3.79 3.77 3.60 3.59 3.57 3.56
N
N
N
N
NH
OH
Cl
3.22
1.18
1.17
2.09
2.13
1.13
2.18
2.10
f1 (ppm)

1H NMR of 12b

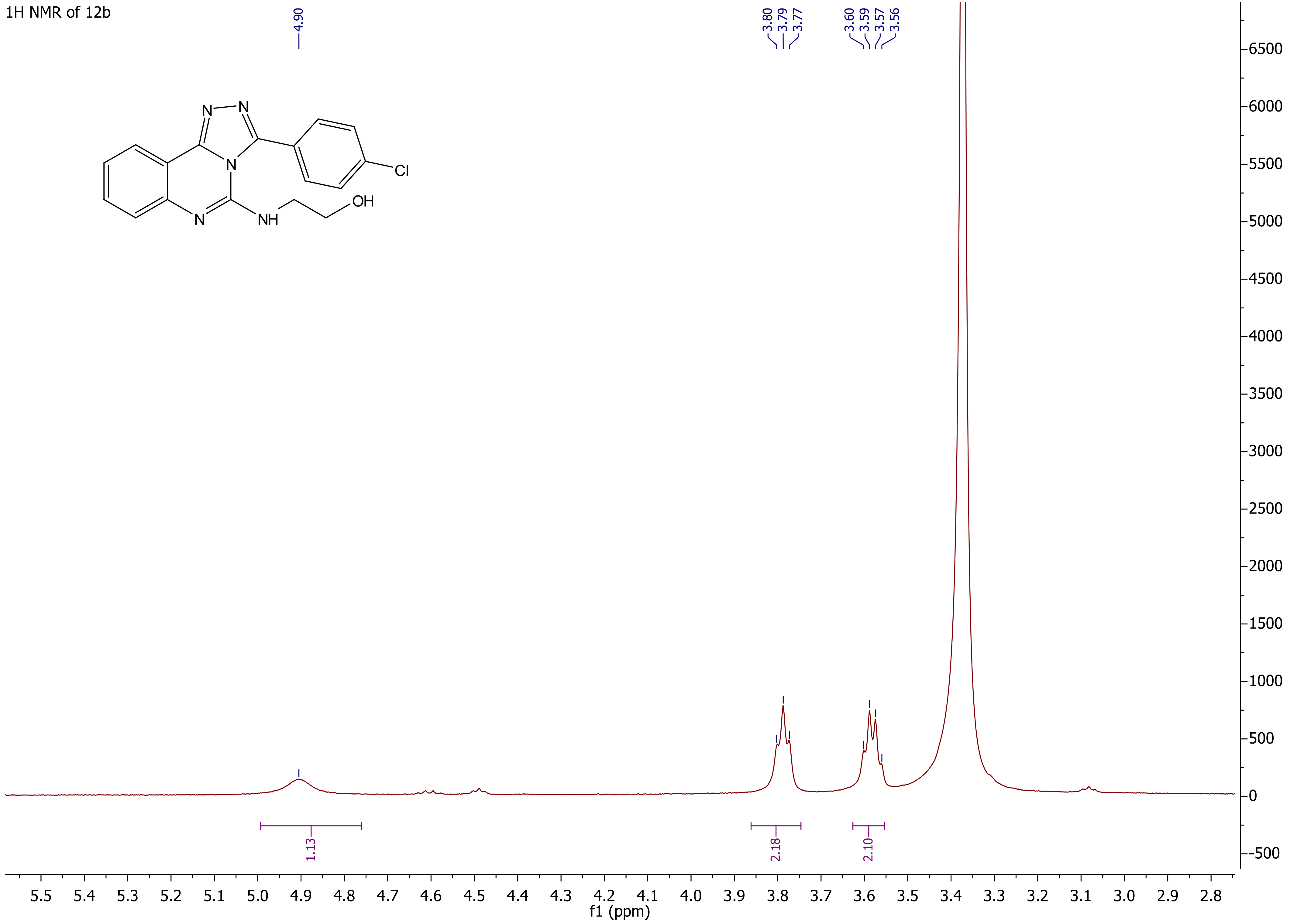

4.90
3.80
3.79
3.77
3.60
3.59
3.57
3.56
N
N
N
N
NH
OH
Cl
1.13
2.18
2.10
6500
6000
5500
5000
4500
4000
3500
3000
2500
2000
1500
1000
500
0
-500
5.5
5.4
5.3
5.2
5.1
5.0
4.9
4.8
4.7
4.6
4.5
4.4
4.3
4.2
4.1
4.0
3.9
3.8
3.7
3.6
3.5
3.4
3.3
3.2
3.1
3.0
2.9
2.8
f1 (ppm)

1H NMR of 12b

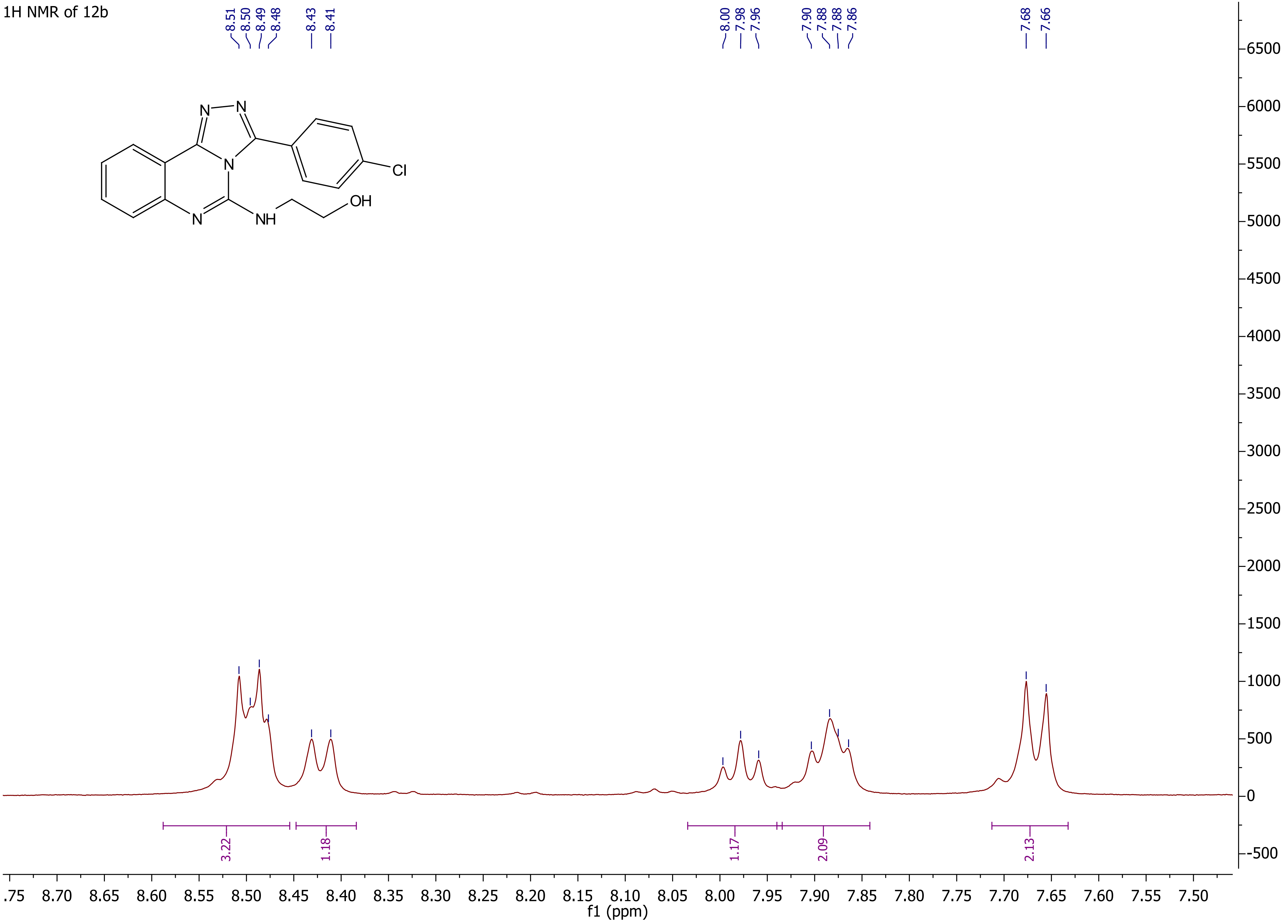

1H NMR of 13a

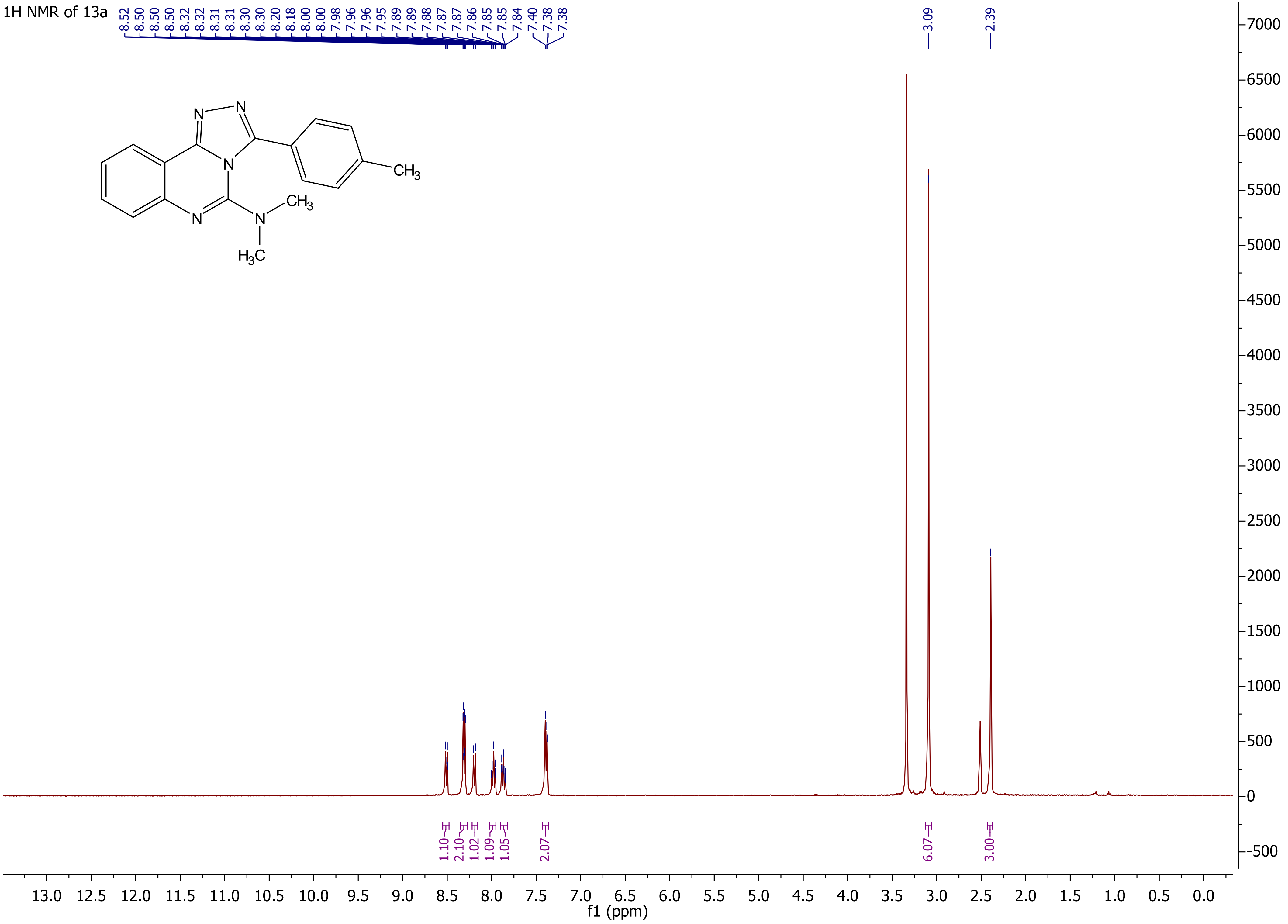

8.52 8.50 8.50 8.50 8.32 8.32 8.31 8.31 8.30 8.30 8.20 8.18 8.00 8.00 7.98 7.96 7.96 7.95 7.89 7.89 7.88 7.87 7.87 7.86 7.85 7.85 7.84 7.40 7.38 7.38
3.09
2.39
N
N
N
N
CH3
CH3
N
H3C
1.10
2.10
1.02
1.09
1.05
2.07
6.07
3.00
7000 6500 6000 5500 5000 4500 4000 3500 3000 2500 2000 1500 1000 500 0 -500
13.0 12.5 12.0 11.5 11.0 10.5 10.0 9.5 9.0 8.5 8.0 7.5 7.0 6.5 6.0 5.5 5.0 4.5 4.0 3.5 3.0 2.5 2.0 1.5 1.0 0.5 0.0
f1 (ppm)

1H NMR of 13a

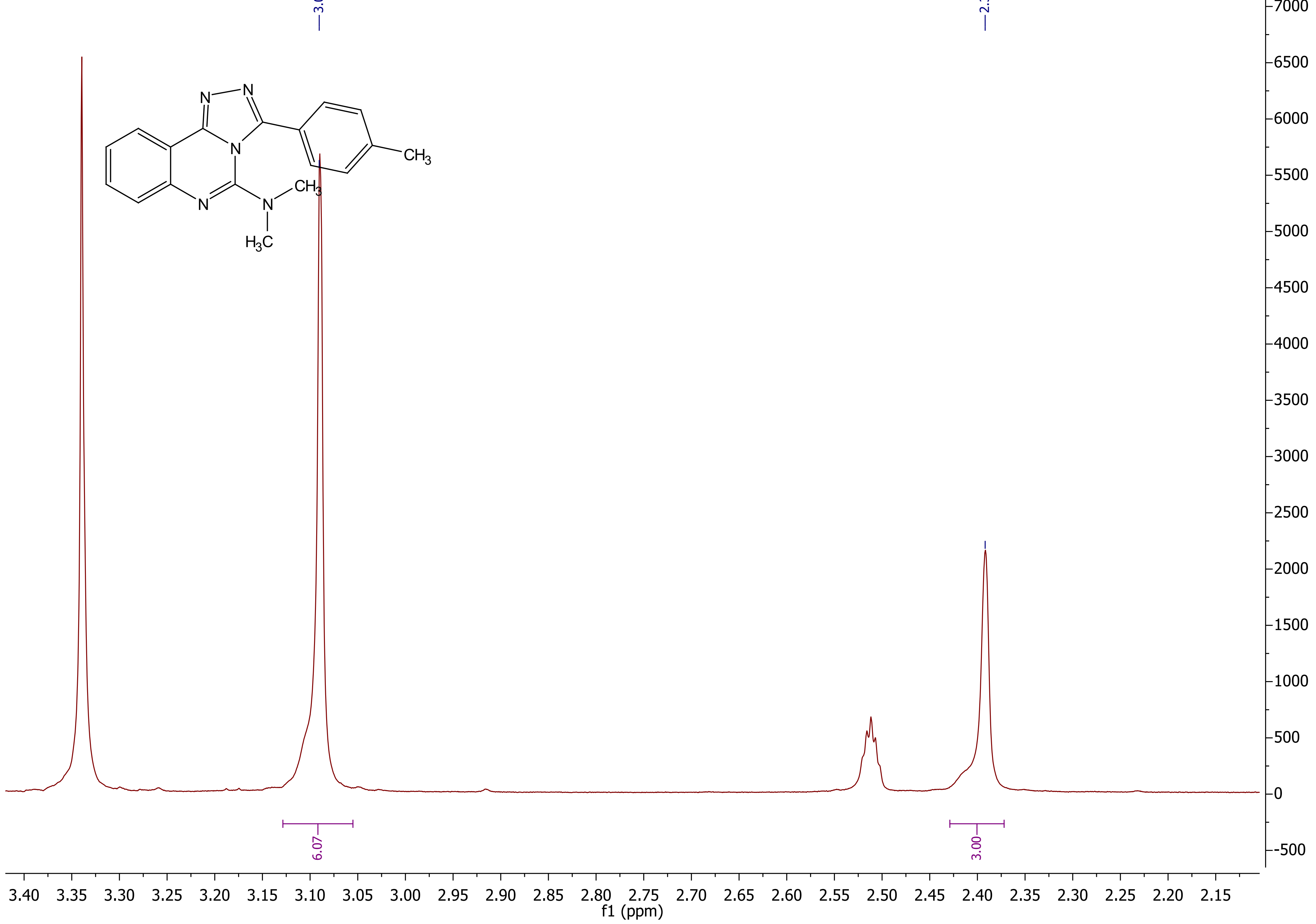

1H NMR of 13a

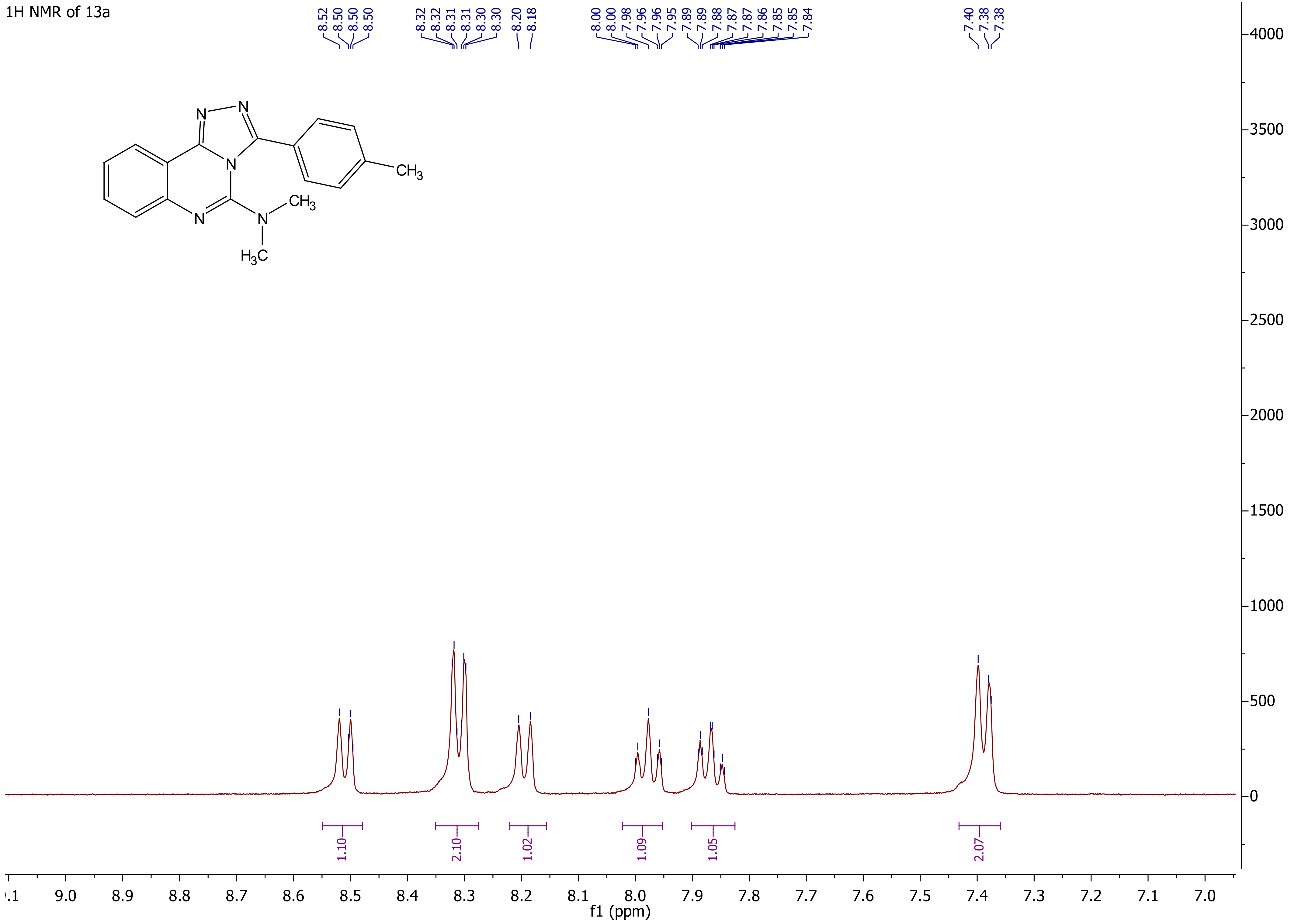
N
N
N
N
N
CH3
CH3
H3C
8.52
8.50
8.50
8.50
8.32
8.32
8.31
8.31
8.30
8.30
8.20
8.18
8.00
8.00
7.98
7.96
7.96
7.95
7.89
7.89
7.88
7.87
7.87
7.86
7.85
7.85
7.84
7.40
7.38
7.38
1.10
2.10
1.02
1.09
1.05
2.07
9.0 8.9 8.8 8.7 8.6 8.5 8.4 8.3 8.2 8.1 8.0 7.9 7.8 7.7 7.6 7.5 7.4 7.3 7.2 7.1 7.0
f1 (ppm)
4000
3500
3000
2500
2000
1500
1000
500
0

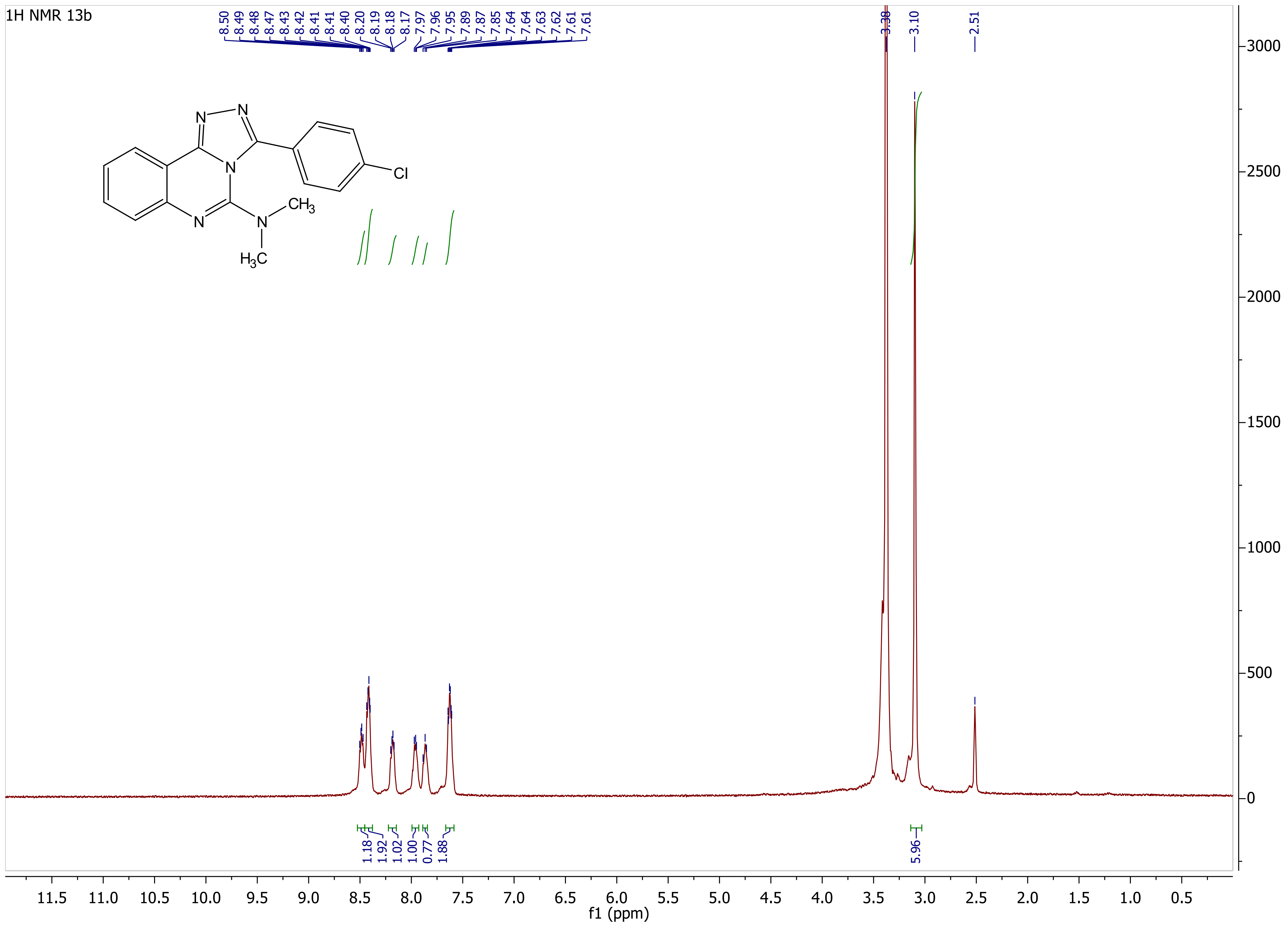
1H NMR 13b
8.50
8.49
8.48
8.47
8.43
8.42
8.41
8.41
8.40
8.20
8.19
8.18
8.17
7.97
7.96
7.95
7.89
7.87
7.85
7.64
7.64
7.63
7.62
7.61
7.61
3.38
3.10
2.51
N
N
N
N
N
Cl
CH3
H3C
1.18
1.92
1.02
1.00
0.77
1.88
5.96
f1 (ppm)

1H NMR 13b

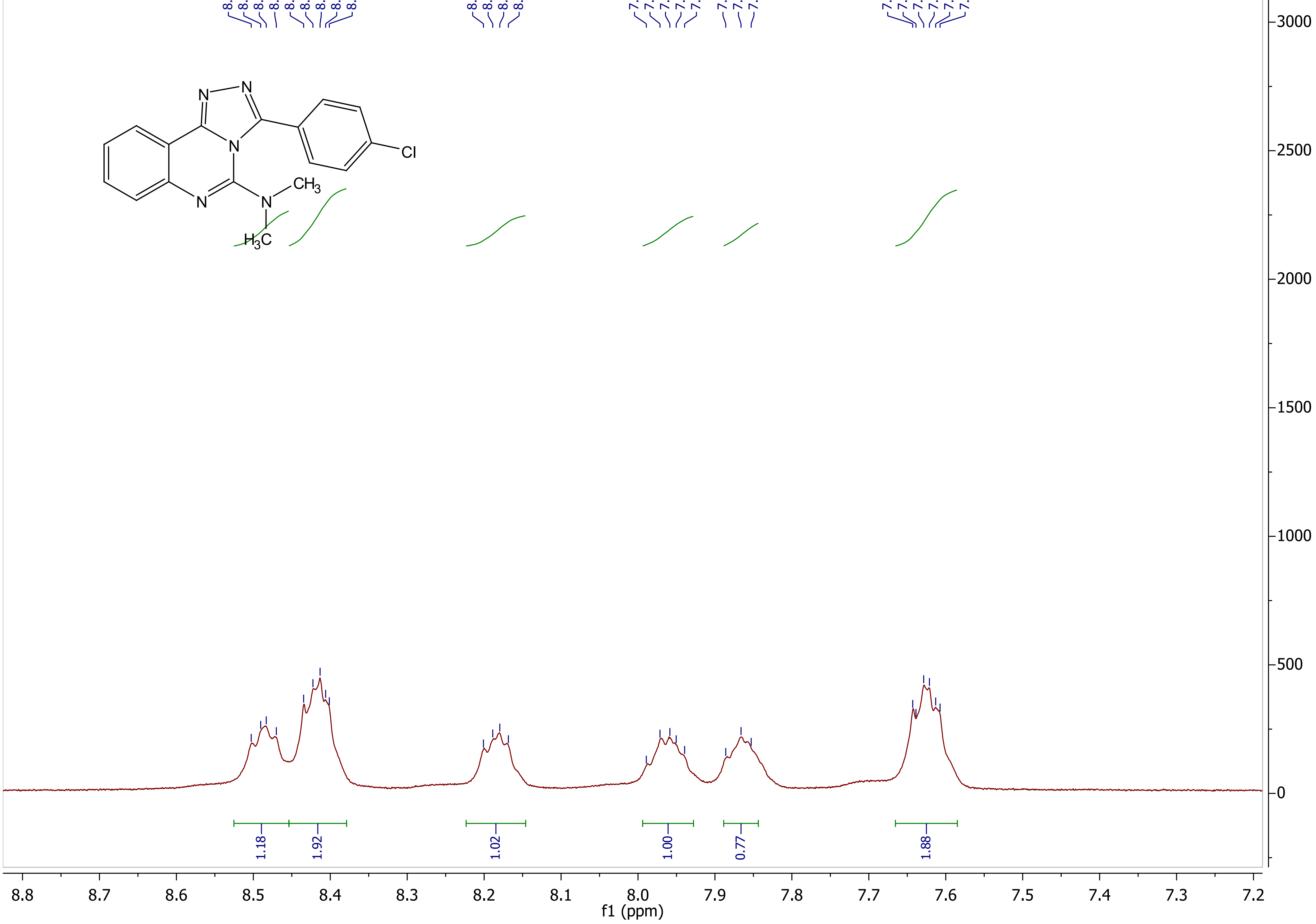
8.50
8.49
8.48
8.47
8.43
8.42
8.41
8.41
8.40
8.20
8.19
8.18
8.17
7.99
7.97
7.96
7.95
7.94
7.89
7.87
7.85
7.64
7.64
7.63
7.62
7.61
7.61
N
N
N
N
N
Cl
CH3
H3C
1.18
1.92
1.02
1.00
0.77
1.88
3000
2500
2000
1500
1000
500
0
8.8
8.7
8.6
8.5
8.4
8.3
8.2
8.1
8.0
7.9
7.8
7.7
7.6
7.5
7.4
7.3
7.2
f1 (ppm)

1H NMR of 14a

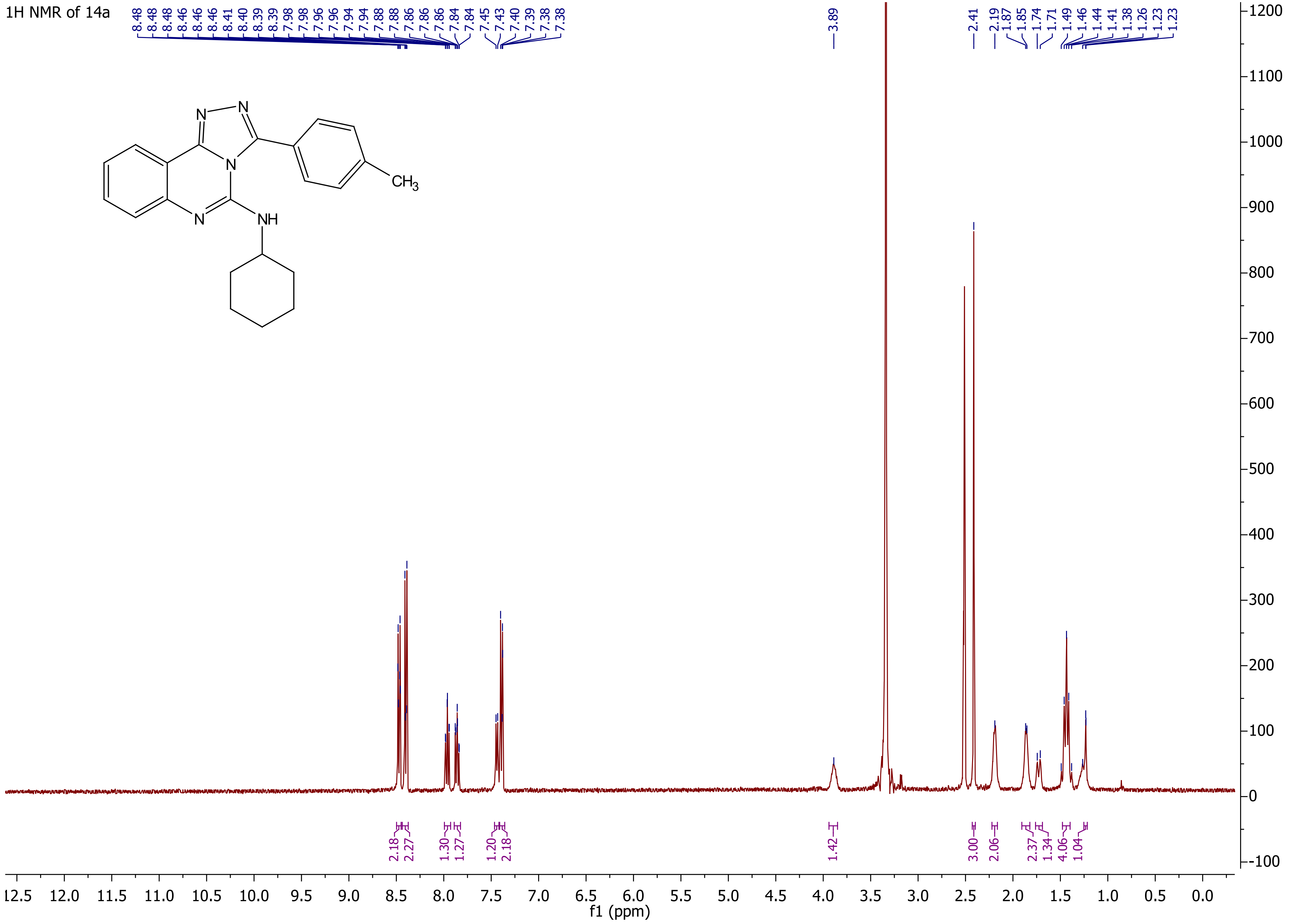

8.48 8.48 8.48 8.46 8.46 8.46 8.41 8.40 8.39 8.39 7.98 7.98 7.96 7.96 7.94 7.94 7.88 7.88 7.86 7.86 7.86 7.84 7.84 7.45 7.43 7.40 7.39 7.38 7.38
3.89
2.41 2.19 1.87 1.85 1.74 1.71 1.49 1.46 1.44 1.41 1.38 1.26 1.23 1.23
N
N
N
N
NH
CH3
2.18 2.27 1.30 1.27 1.20 2.18 1.42 3.00 2.06 2.37 1.34 4.06 1.04
f1 (ppm)

1H NMR of 14a

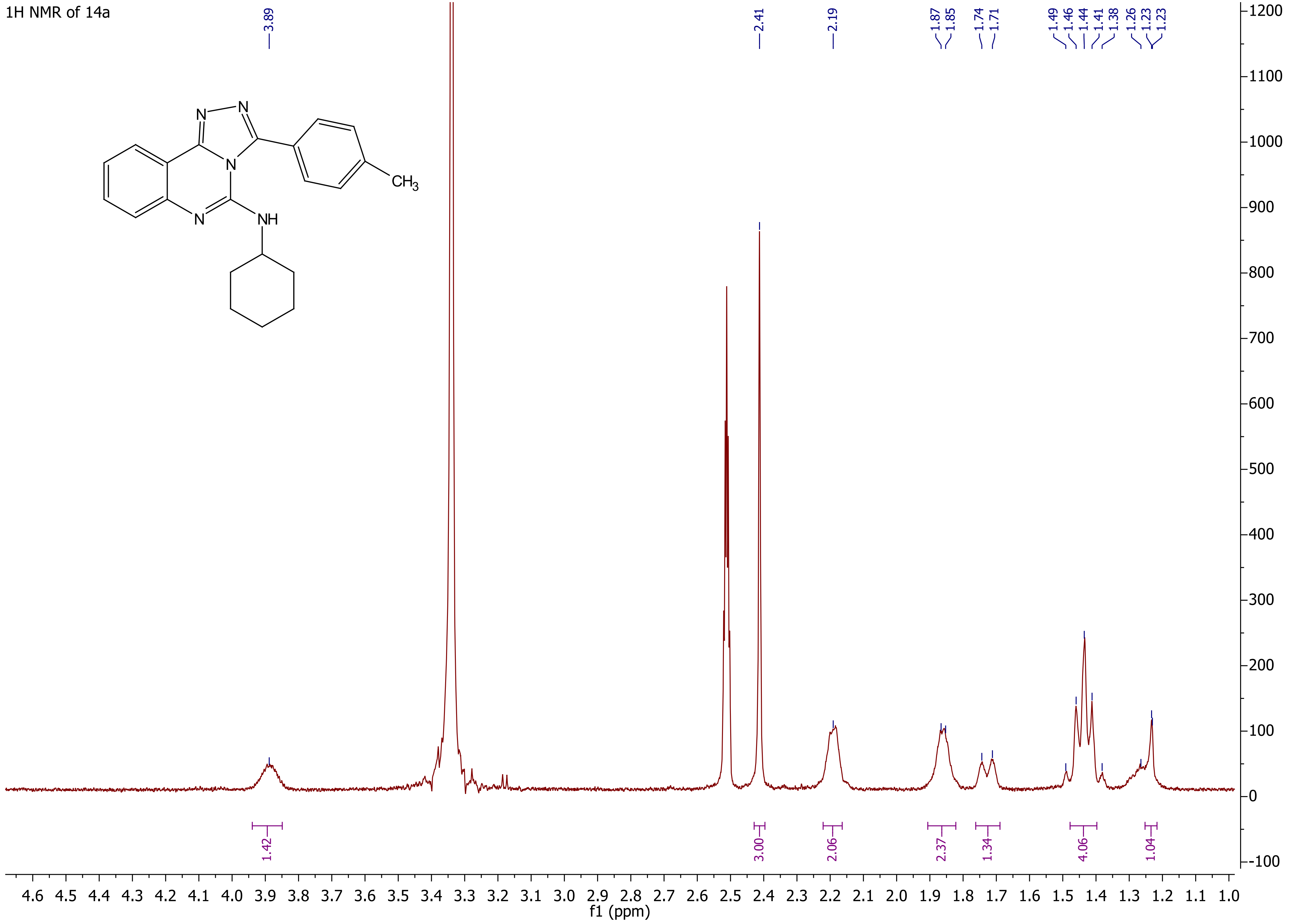

1H NMR of 14a

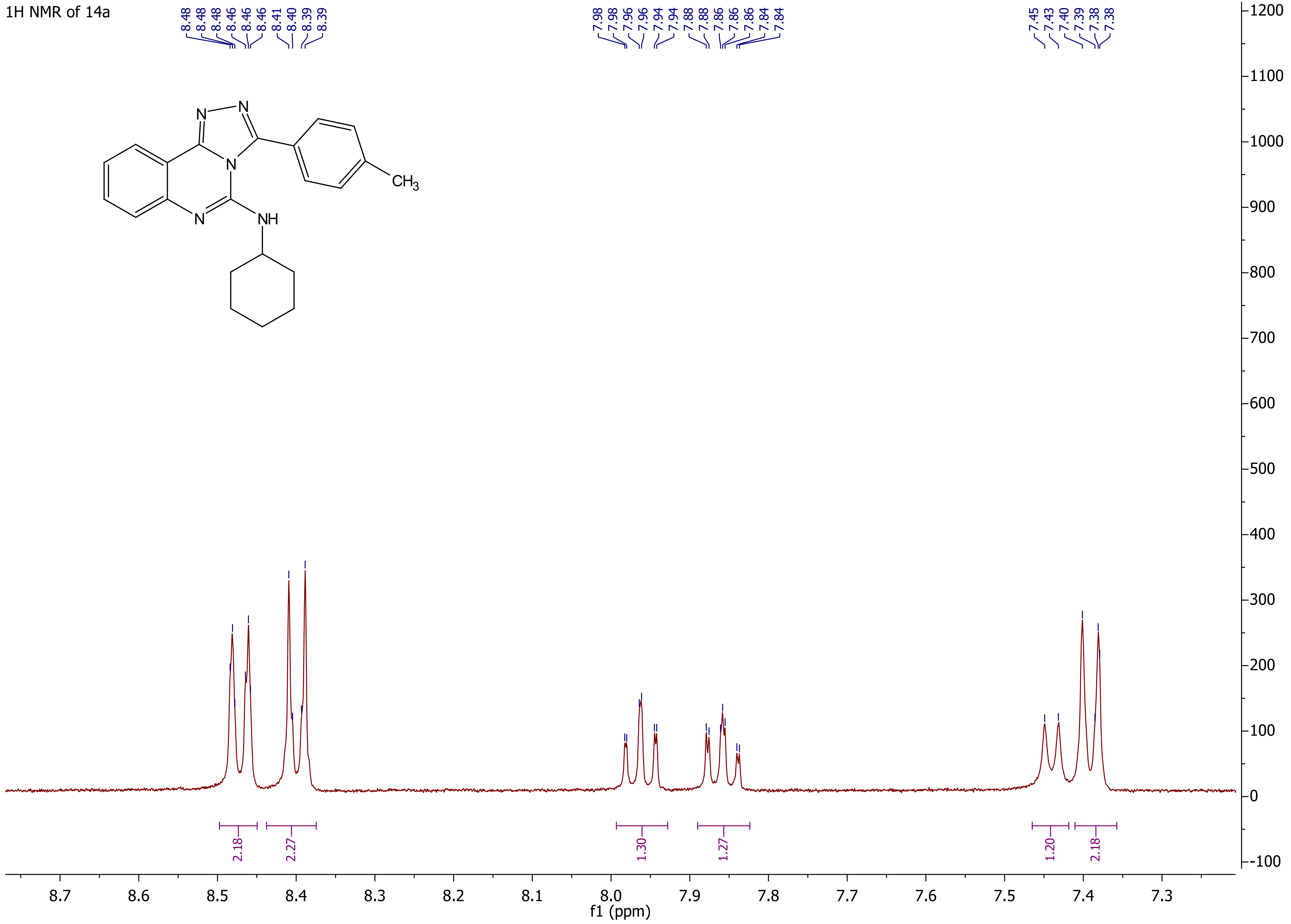
8.48
8.48
8.48
8.46
8.46
8.46
8.41
8.40
8.39
8.39
7.98
7.98
7.96
7.96
7.94
7.94
7.88
7.88
7.86
7.86
7.86
7.84
7.84
7.45
7.43
7.40
7.39
7.38
7.38
N
N
N
N
NH
CH3
2.18
2.27
1.30
1.27
1.20
2.18
8.7
8.6
8.5
8.4
8.3
8.2
8.1
8.0
7.9
7.8
7.7
7.6
7.5
7.4
7.3
f1 (ppm)
1200
1100
1000
900
800
700
600
500
400
300
200
100
0
-100

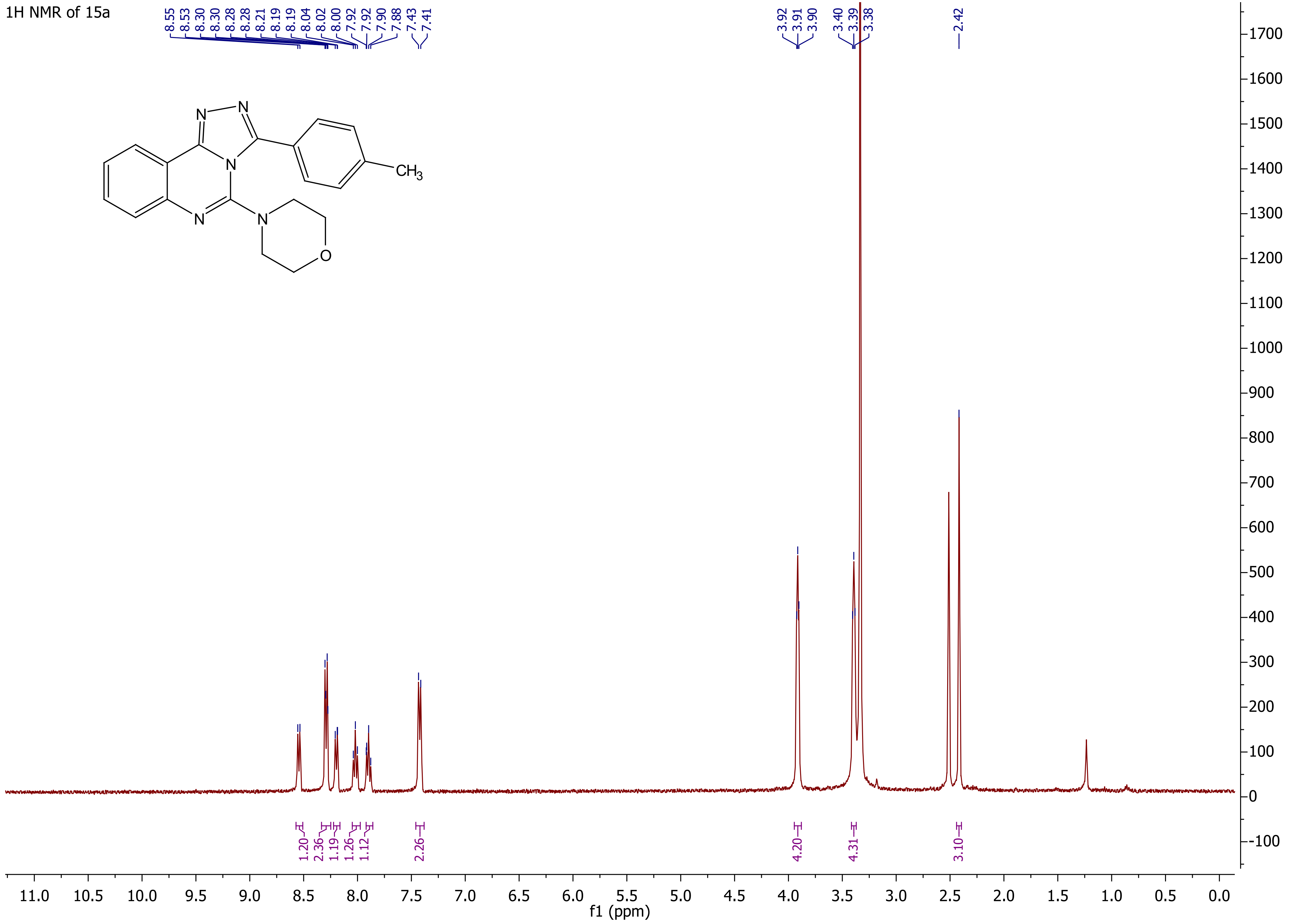

1H NMR of 15a
8.55
8.53
8.30
8.30
8.28
8.28
8.21
8.19
8.19
8.04
8.02
8.00
7.92
7.92
7.90
7.88
7.43
7.41
3.92
3.91
3.90
3.40
3.39
3.38
2.42
1.20
2.36
1.19
1.26
1.12
2.26
4.20
4.31
3.10
f1 (ppm)

1H NMR of 15a

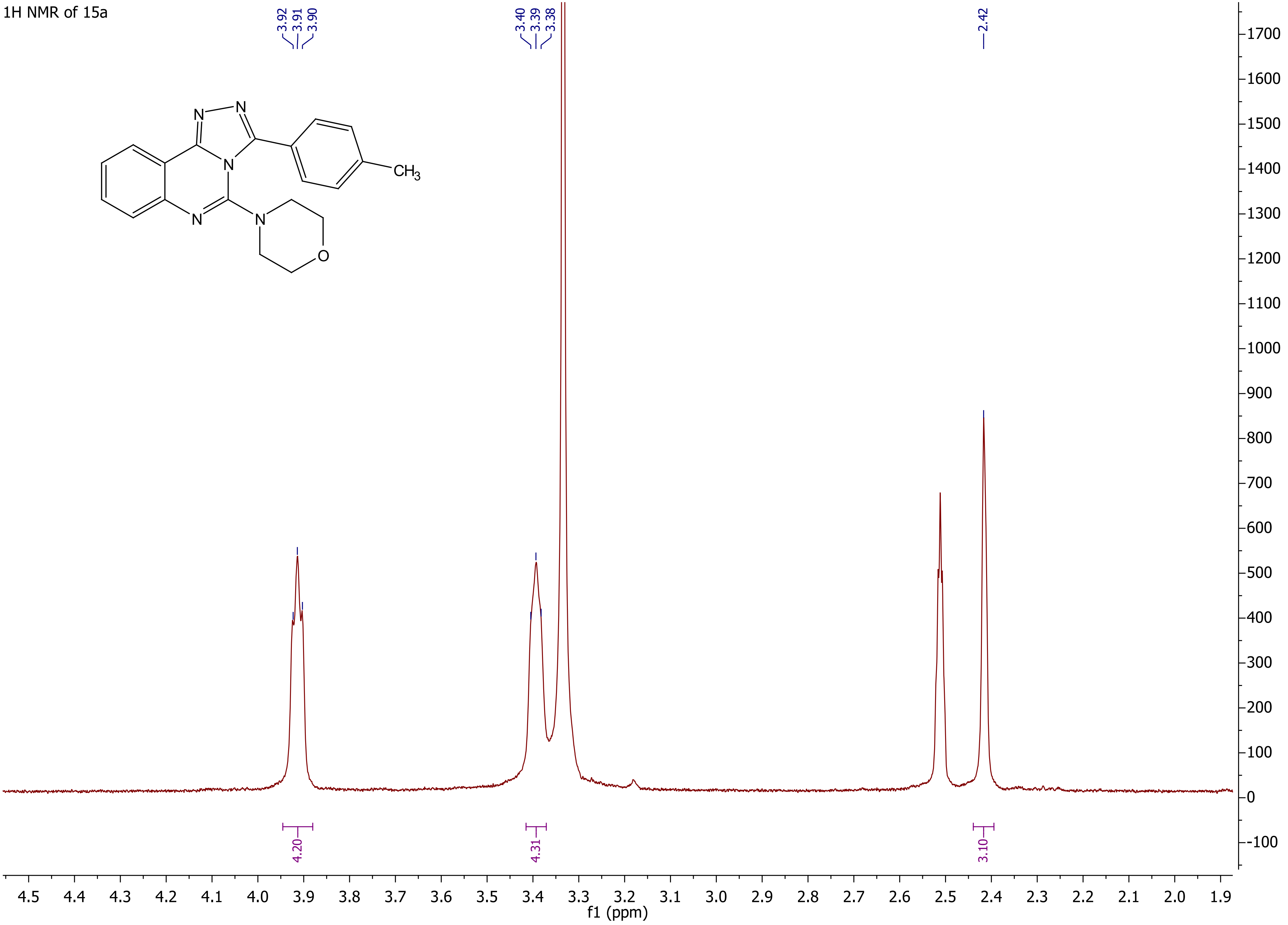

3.92
3.91
3.90
3.40
3.39
3.38
2.42
CH3
N
N
N
N
N
O
4.20
4.31
3.10
4.5 4.4 4.3 4.2 4.1 4.0 3.9 3.8 3.7 3.6 3.5 3.4 3.3 3.2 3.1 3.0 2.9 2.8 2.7 2.6 2.5 2.4 2.3 2.2 2.1 2.0 1.9
f1 (ppm)
1700 1600 1500 1400 1300 1200 1100 1000 900 800 700 600 500 400 300 200 100 0 -100

1H NMR of 15a

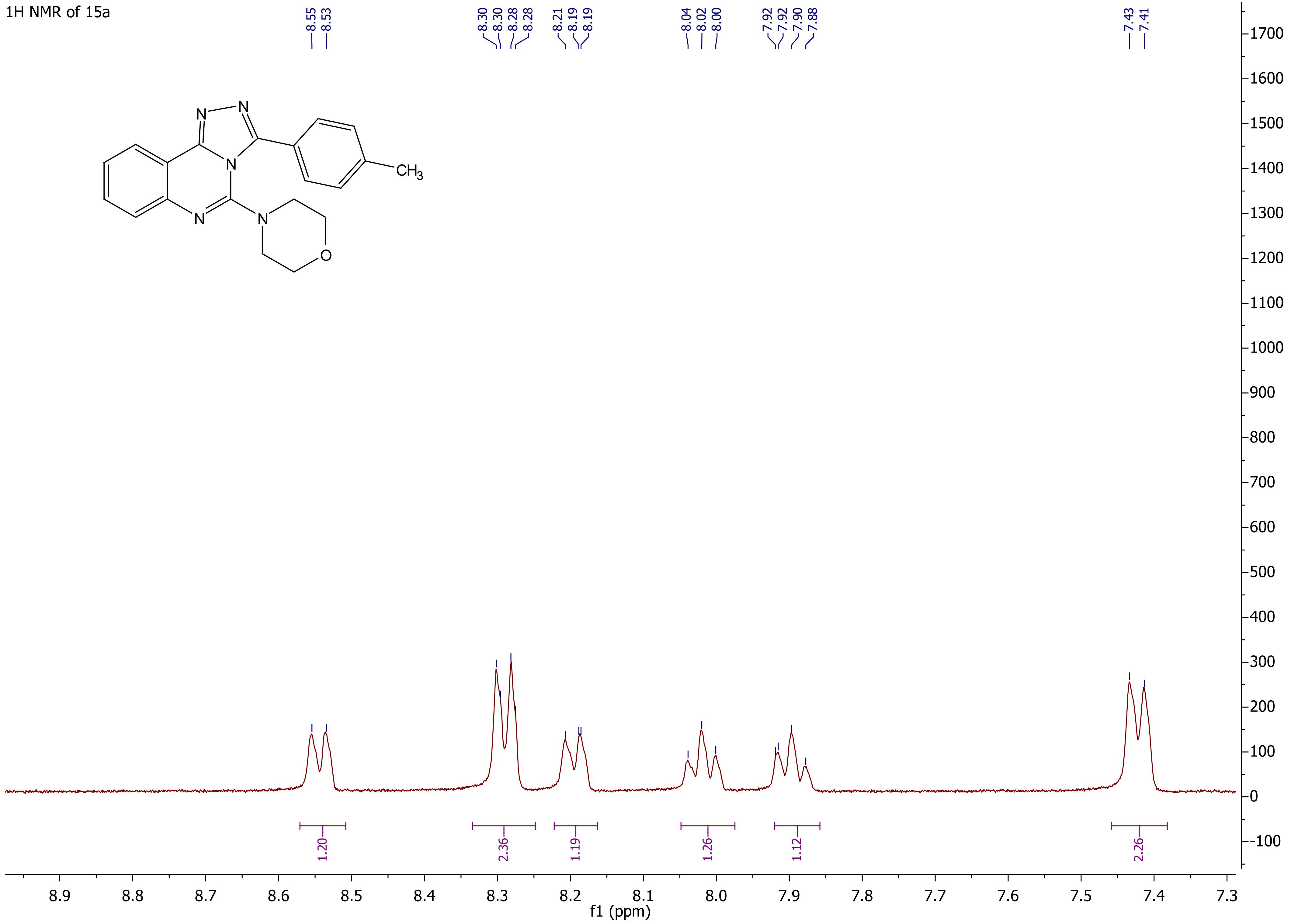

N
N
N
N
N
O
CH3
8.55
8.53
8.30
8.30
8.28
8.28
8.21
8.19
8.19
8.04
8.02
8.00
7.92
7.92
7.90
7.88
7.43
7.41
1.20
2.36
1.19
1.26
1.12
2.26
f1 (ppm)

1H NMR of 16

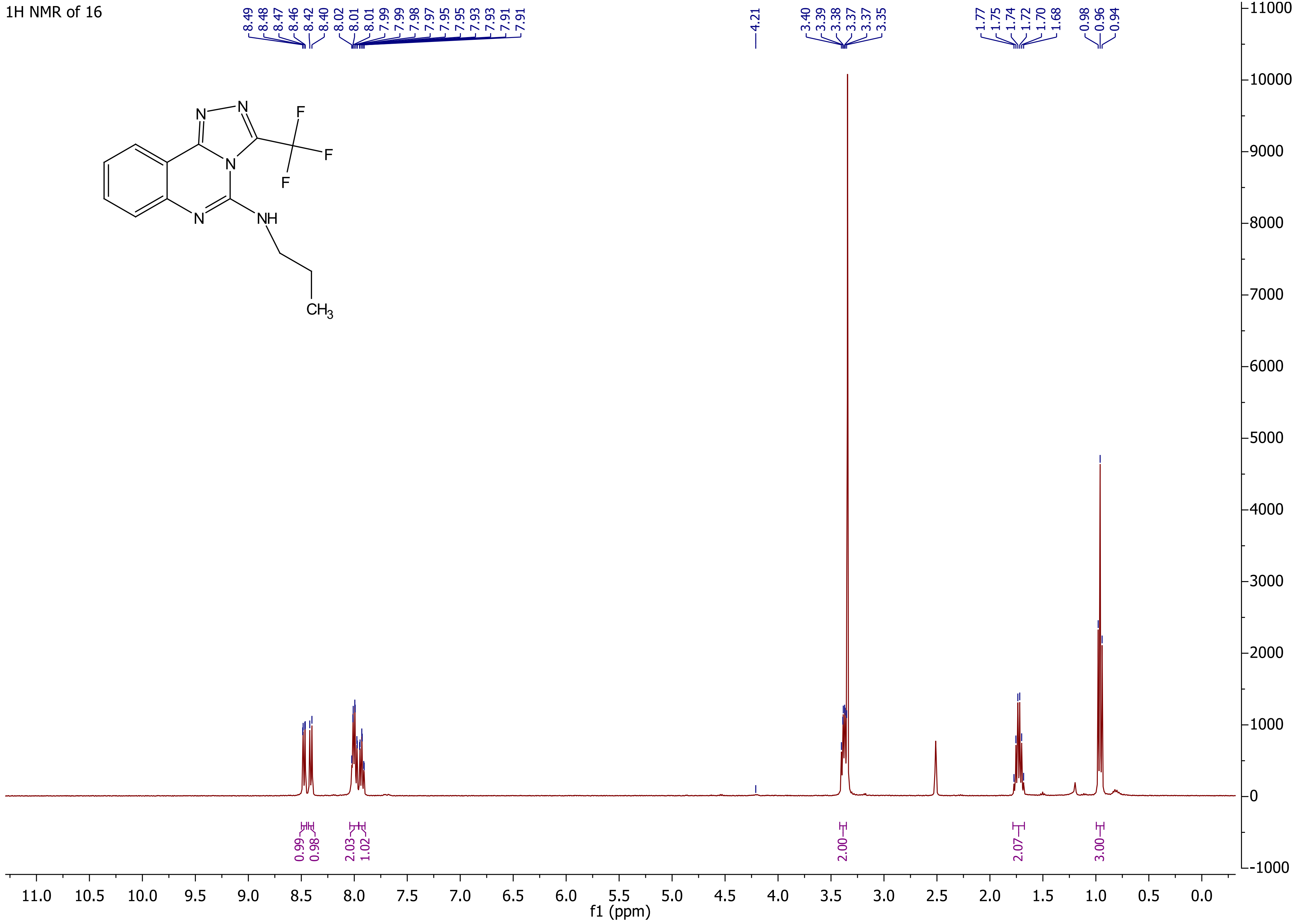

8.49
8.48
8.47
8.46
8.42
8.40
8.02
8.01
8.01
7.99
7.99
7.98
7.97
7.95
7.95
7.93
7.93
7.91
7.91
4.21
3.40
3.39
3.38
3.37
3.37
3.35
1.77
1.75
1.74
1.72
1.70
1.68
0.98
0.96
0.94
N
N
N
N
NH
F
F
F
CH3
0.99
0.98
2.03
1.02
2.00
2.07
3.00
11.0 10.5 10.0 9.5 9.0 8.5 8.0 7.5 7.0 6.5 6.0 5.5 5.0 4.5 4.0 3.5 3.0 2.5 2.0 1.5 1.0 0.5 0.0
f1 (ppm)
11000 10000 9000 8000 7000 6000 5000 4000 3000 2000 1000 0 -1000

1H NMR of 16

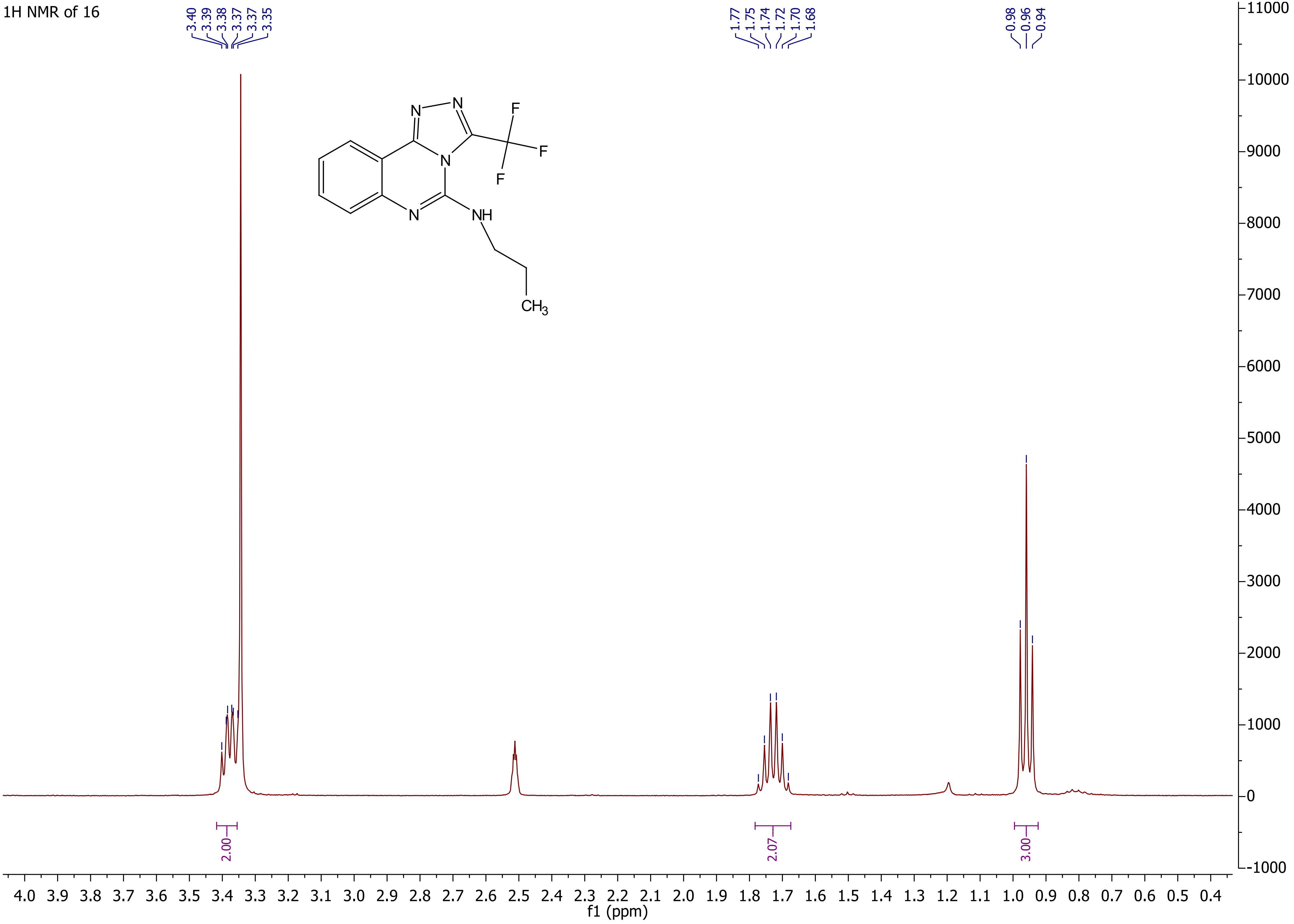

3.40
3.39
3.38
3.37
3.37
3.35
1.77
1.75
1.74
1.72
1.70
1.68
0.98
0.96
0.94
N
N
N
N
F
F
F
NH
CH3
2.00
2.07
3.00
f1 (ppm)

1H NMR of 16

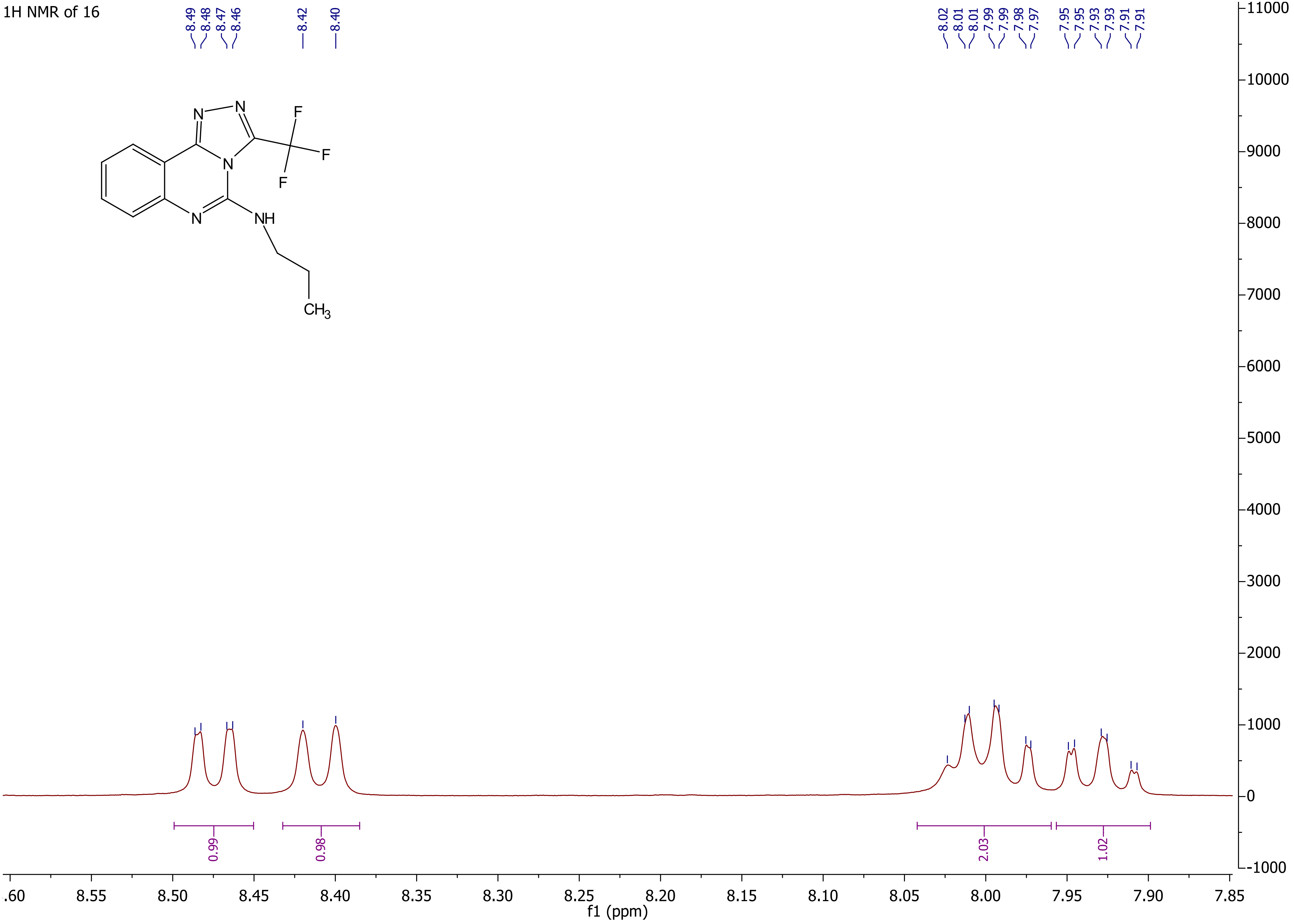
8.49
8.48
8.47
8.46
8.42
8.40
8.02
8.01
8.01
7.99
7.99
7.98
7.97
7.95
7.95
7.93
7.93
7.91
7.91
N
N
N
N
F
F
F
NH
CH3
0.99
0.98
2.03
1.02
f1 (ppm)

1H NMR of 17

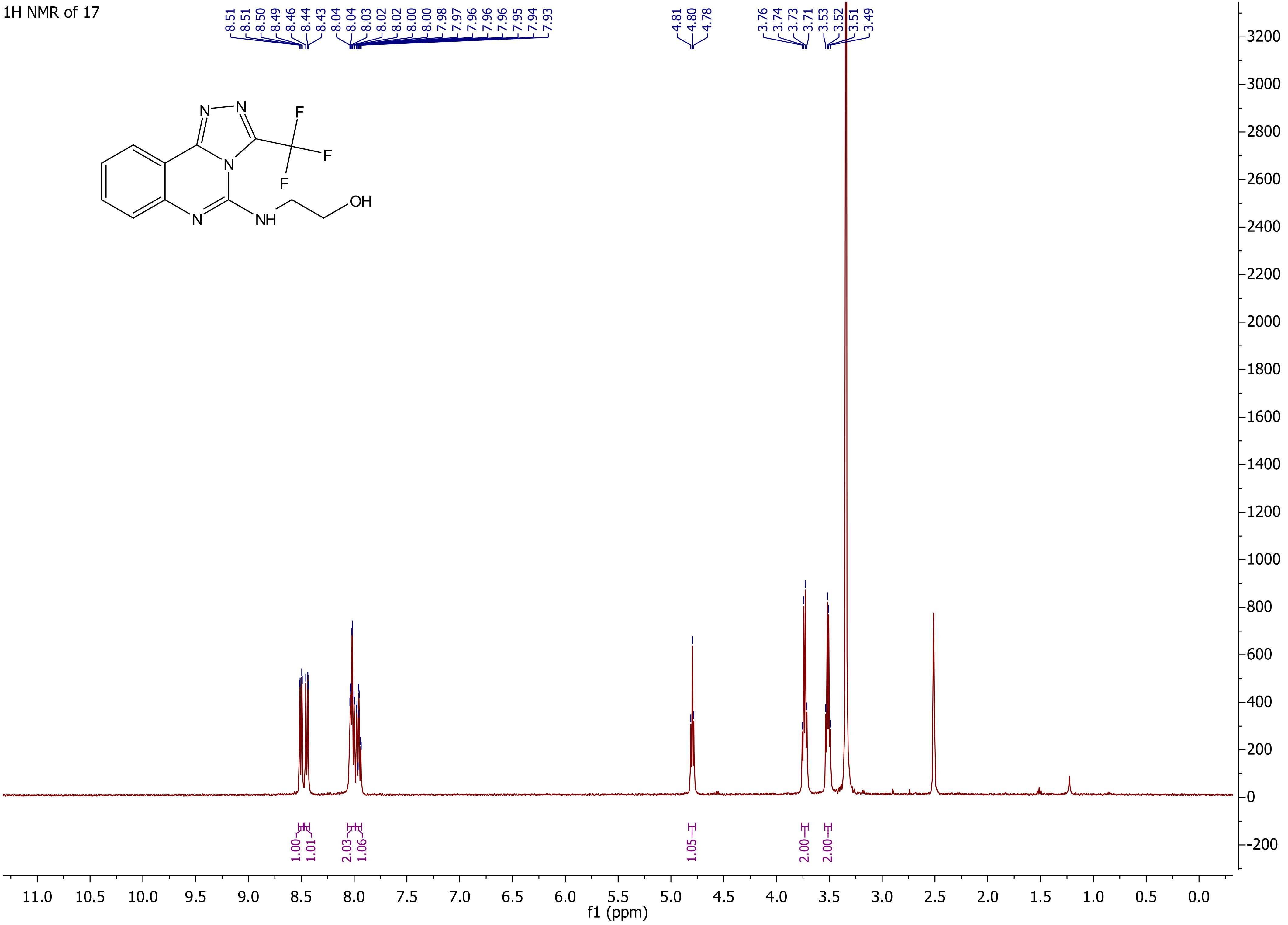
8.51
8.51
8.50
8.49
8.46
8.44
8.43
8.04
8.04
8.03
8.02
8.02
8.00
8.00
7.98
7.97
7.96
7.96
7.96
7.95
7.94
7.93
4.81
4.80
4.78
3.76
3.74
3.73
3.71
3.53
3.52
3.51
3.49
N
N
F
F
F
N
N
NH
OH
1.00
1.01
2.03
1.06
1.05
2.00
2.00
f1 (ppm)

1H NMR of 17

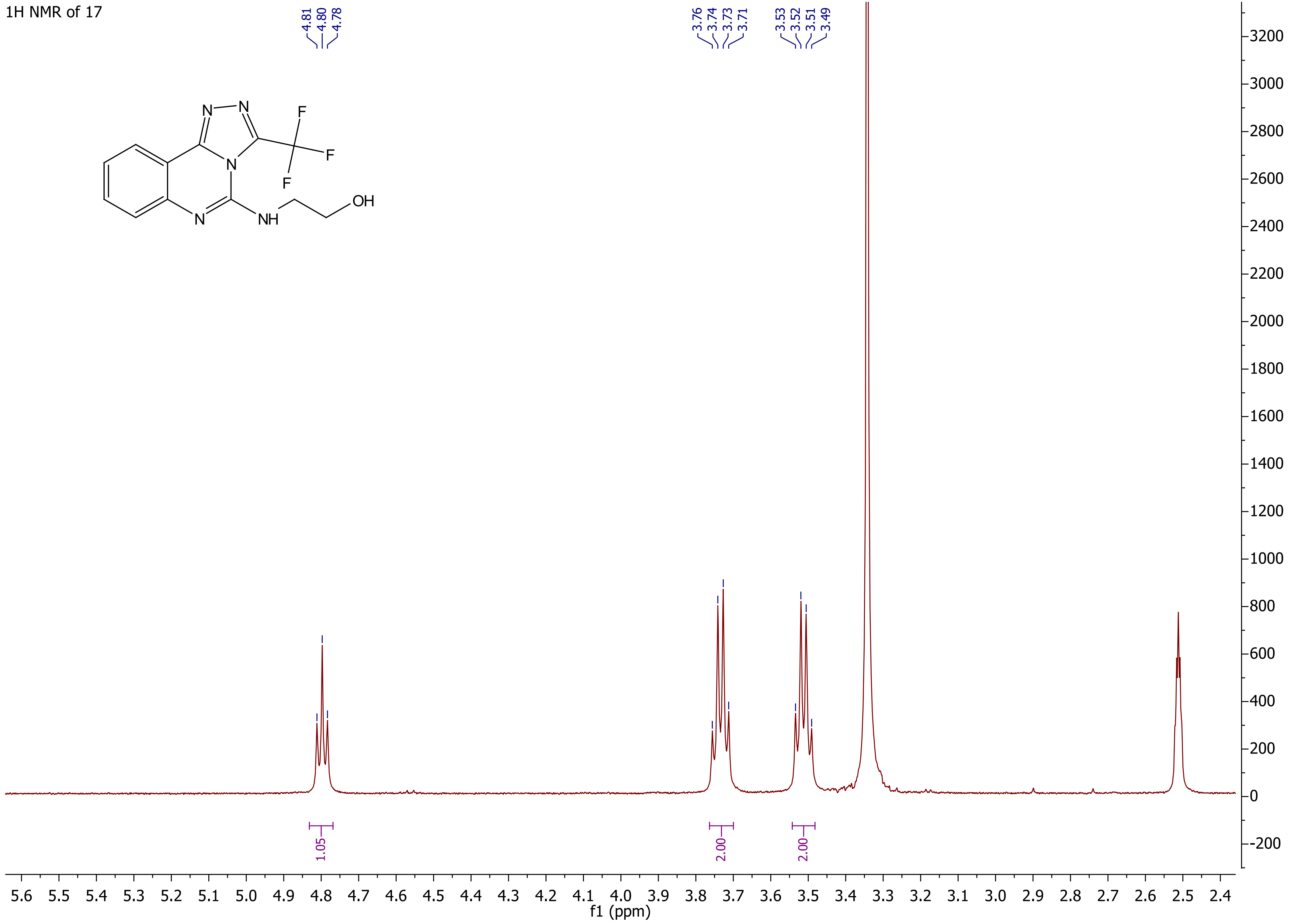
4.81
4.80
4.78
3.76
3.74
3.73
3.71
3.53
3.52
3.51
3.49
N
N
F
F
F
N
N
NH
OH
1.05
2.00
2.00
5.6 5.5 5.4 5.3 5.2 5.1 5.0 4.9 4.8 4.7 4.6 4.5 4.4 4.3 4.2 4.1 4.0 3.9 3.8 3.7 3.6 3.5 3.4 3.3 3.2 3.1 3.0 2.9 2.8 2.7 2.6 2.5 2.4
f1 (ppm)
3200
3000
2800
2600
2400
2200
2000
1800
1600
1400
1200
1000
800
600
400
200
0
-200

1H NMR of 17

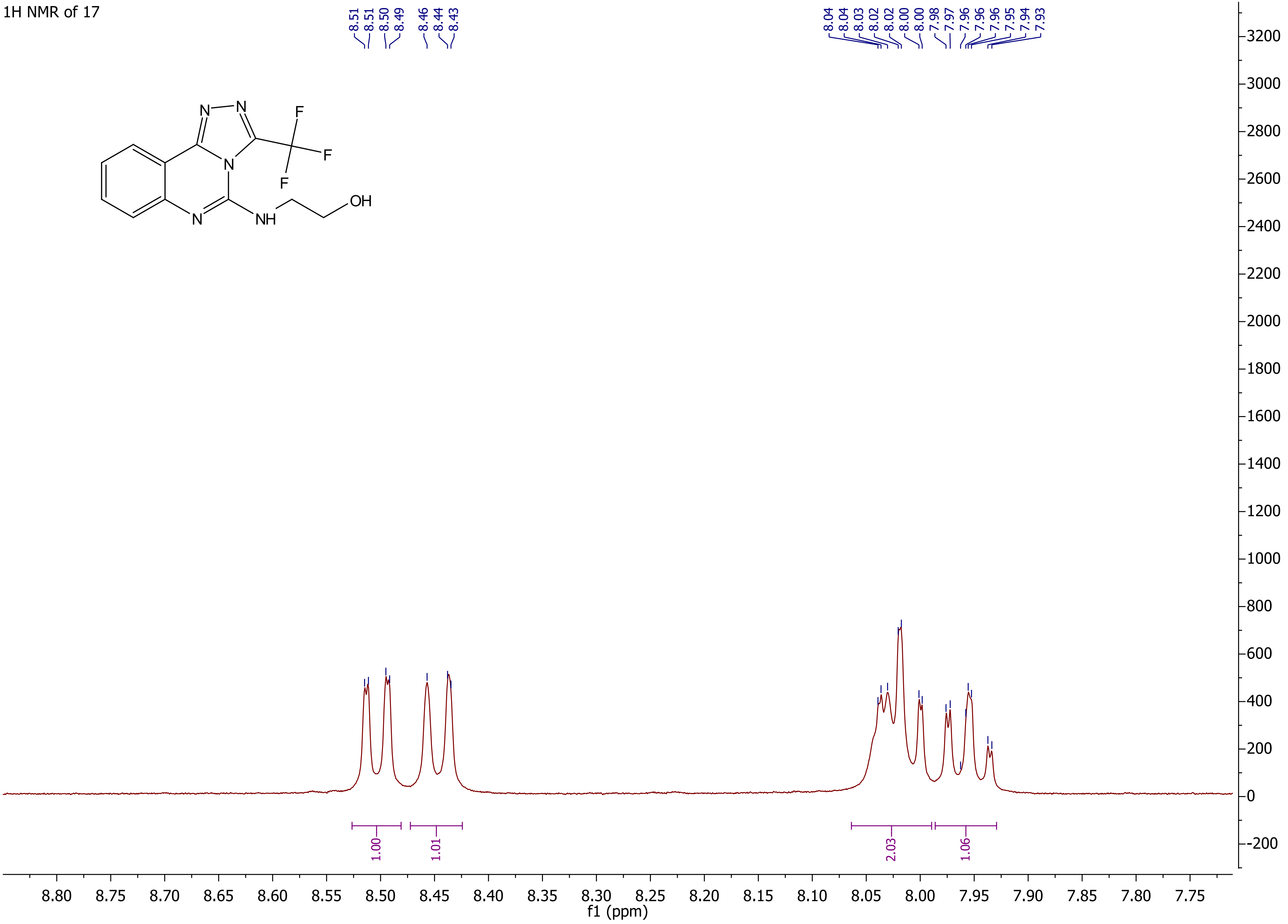

8.51
8.51
8.50
8.49
8.46
8.44
8.43
8.04
8.04
8.03
8.02
8.02
8.00
8.00
7.98
7.97
7.96
7.96
7.96
7.95
7.94
7.93
N
N
N
N
F
F
F
NH
OH
1.00
1.01
2.03
1.06
f1 (ppm)

1H NMR of 18

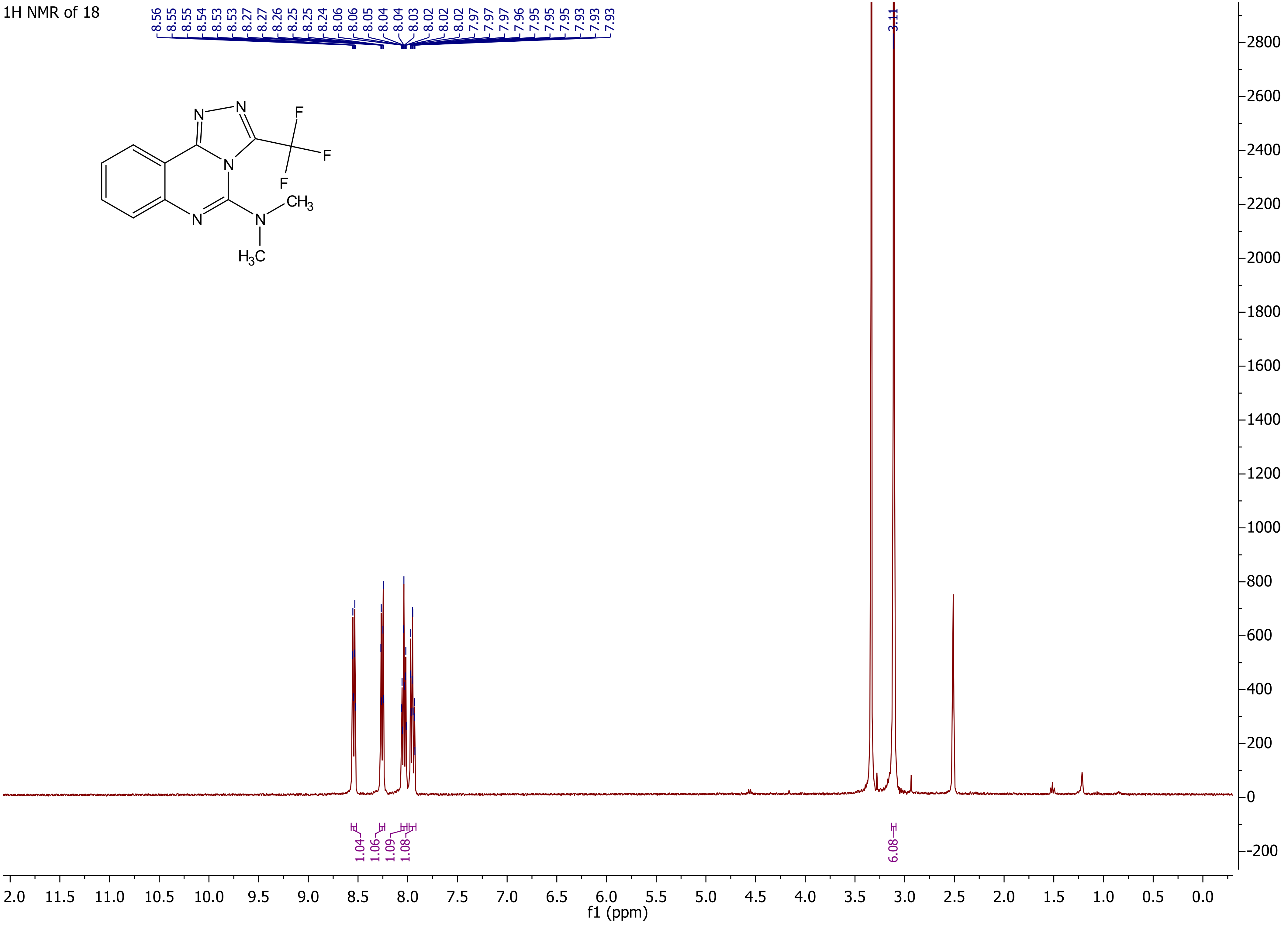

8.56 8.55 8.55 8.54 8.53 8.53 8.27 8.27 8.26 8.25 8.25 8.24 8.06 8.06 8.05 8.04 8.04 8.03 8.02 8.02 8.02 7.97 7.97 7.97 7.96 7.95 7.95 7.95 7.93 7.93 7.93
3.11
N N F F F N CH3 N N H3C
1.04 1.06 1.09 1.08
6.08
f1 (ppm)

1H NMR of 18

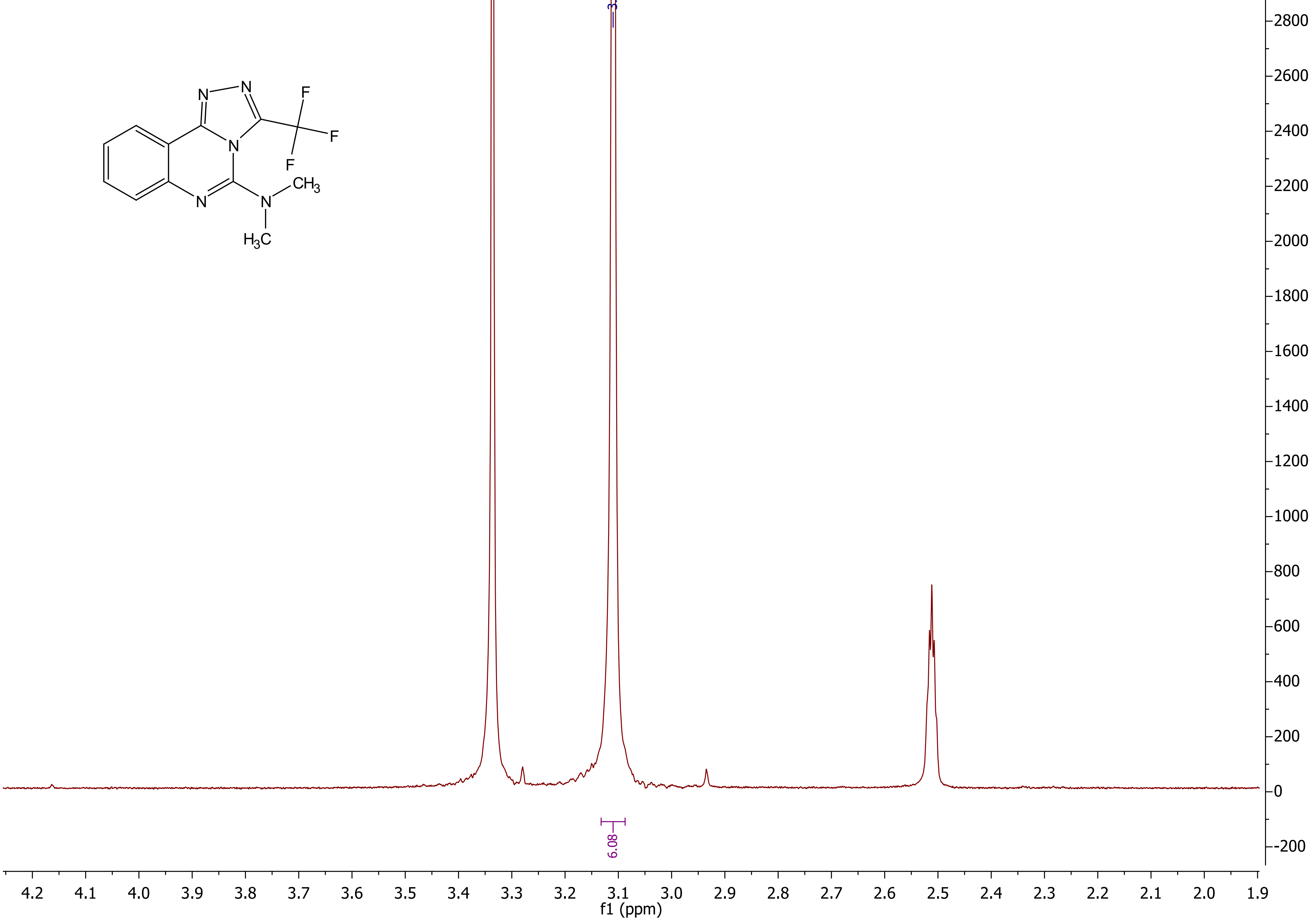

N
N
N
N
N
F
F
F
CH3
H3C
3.11
6.08
4.2 4.1 4.0 3.9 3.8 3.7 3.6 3.5 3.4 3.3 3.2 3.1 3.0 2.9 2.8 2.7 2.6 2.5 2.4 2.3 2.2 2.1 2.0 1.9
f1 (ppm)
2800 2600 2400 2200 2000 1800 1600 1400 1200 1000 800 600 400 200 0 -200

1H NMR of 18

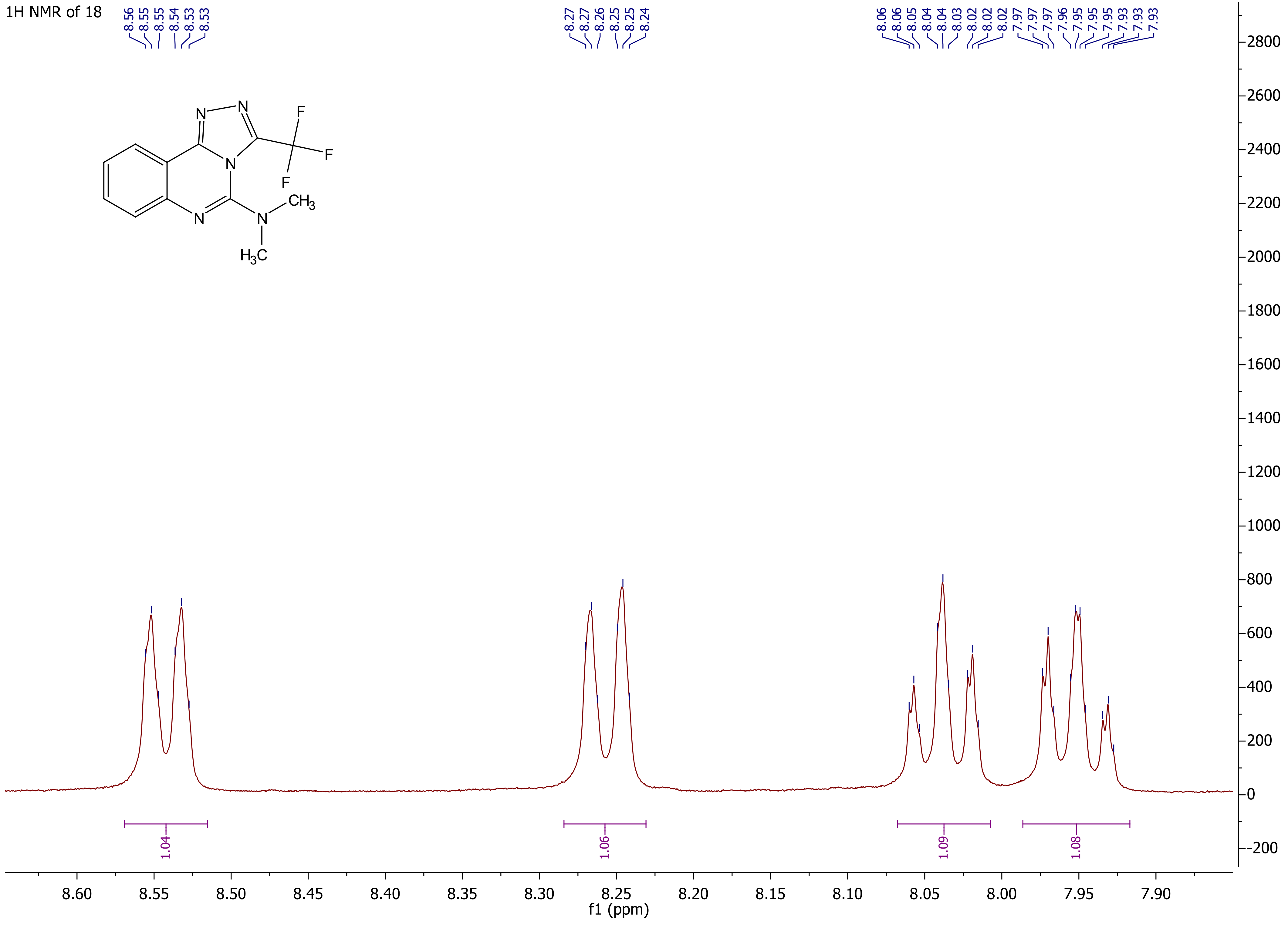
8.56
8.55
8.55
8.54
8.53
8.53
8.27
8.27
8.26
8.25
8.25
8.24
8.06
8.06
8.05
8.04
8.04
8.03
8.02
8.02
8.02
7.97
7.97
7.97
7.96
7.95
7.95
7.95
7.93
7.93
7.93
1.04
1.06
1.09
1.08
f1 (ppm)

1H NMR of 19

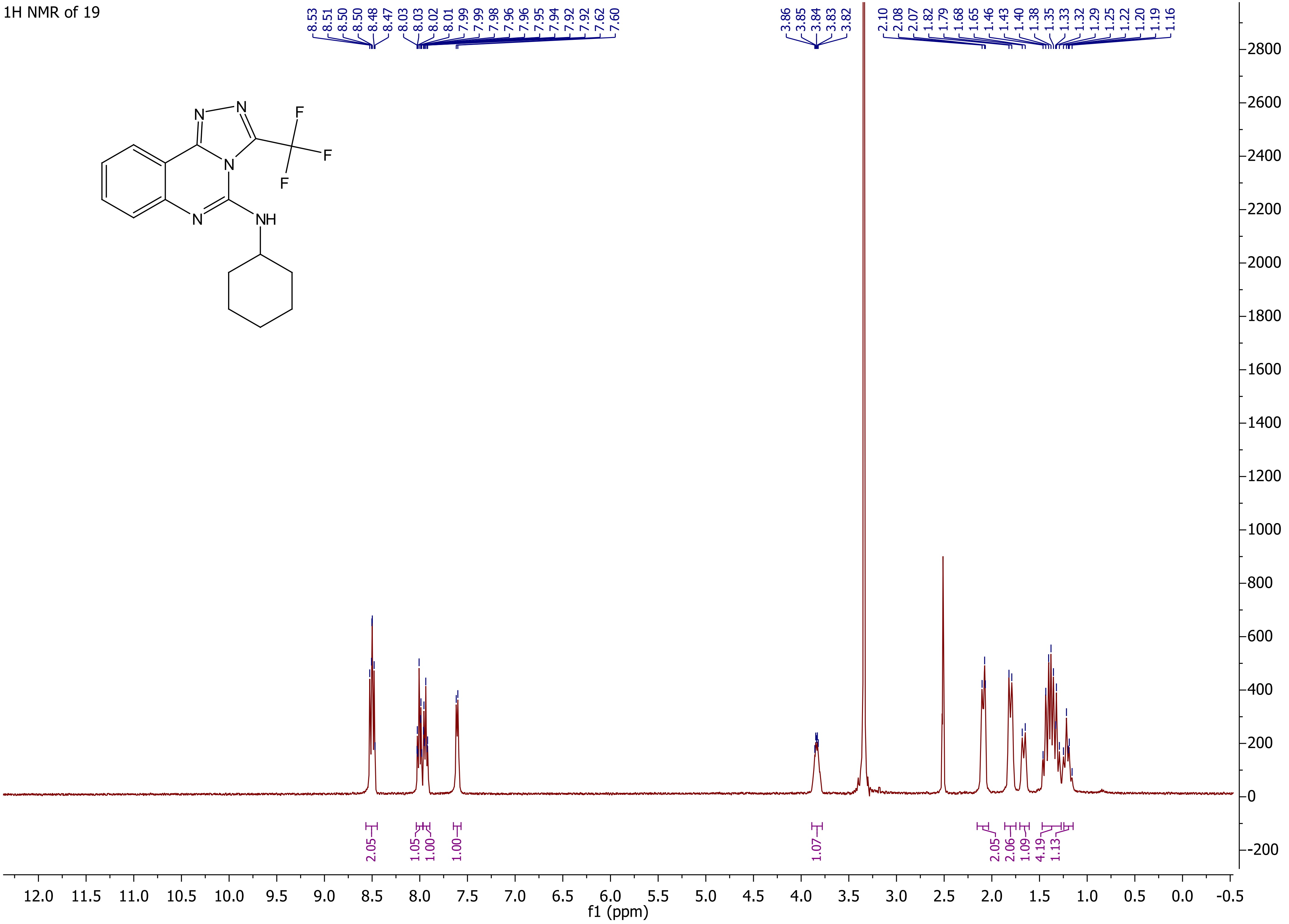
8.53
8.51
8.50
8.50
8.48
8.47
8.03
8.03
8.02
8.01
7.99
7.99
7.98
7.96
7.96
7.95
7.94
7.92
7.92
7.62
7.60
3.86
3.85
3.84
3.83
3.82
2.10
2.08
2.07
1.82
1.79
1.68
1.65
1.46
1.43
1.40
1.38
1.35
1.33
1.32
1.29
1.25
1.22
1.20
1.19
1.16
N
N
N
N
F
F
F
NH
2.05
1.05
1.00
1.00
1.07
2.05
2.06
1.09
4.19
1.13
f1 (ppm)

1H NMR of 19

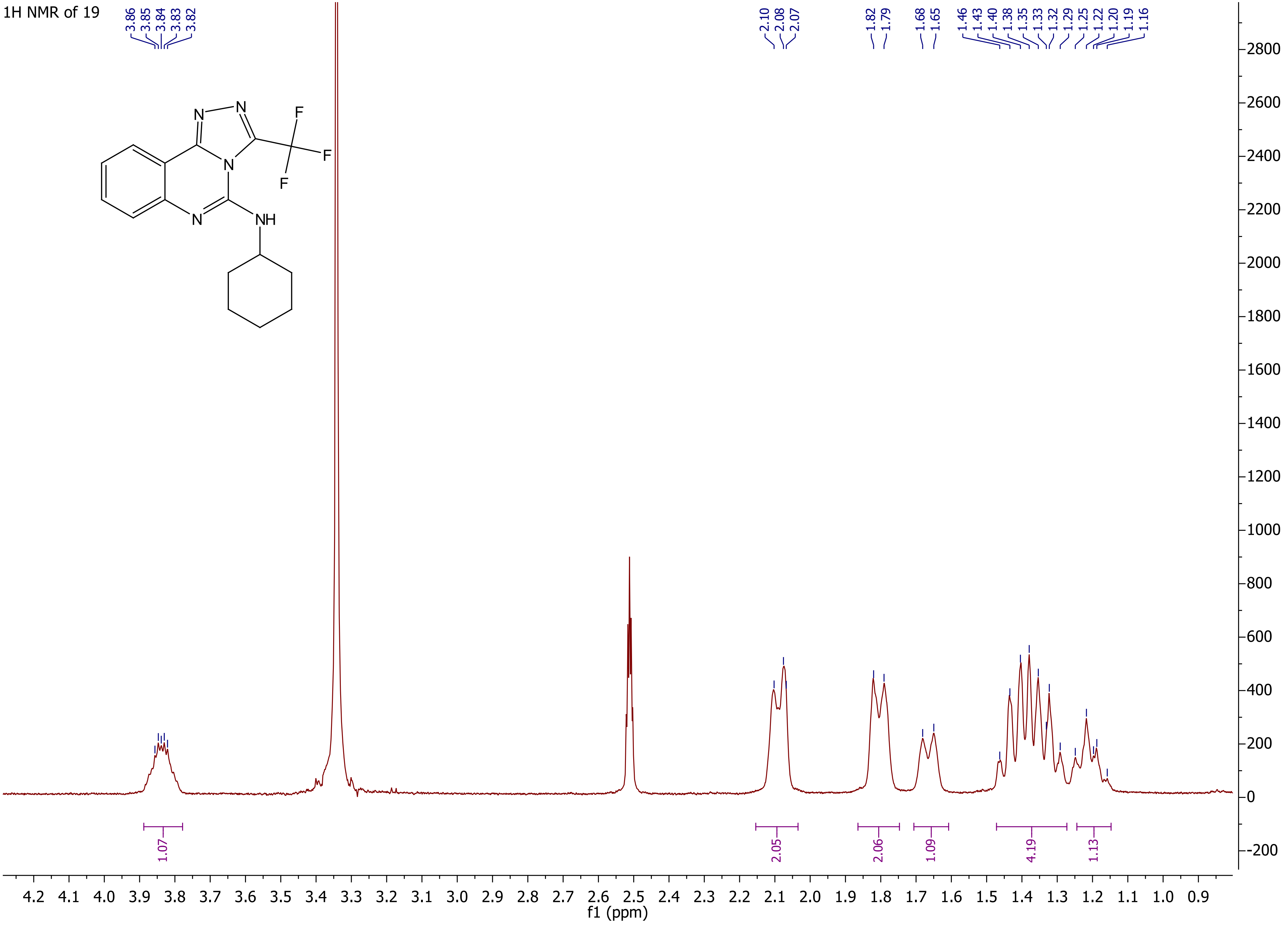

3.86
3.85
3.84
3.83
3.82
2.10
2.08
2.07
1.82
1.79
1.68
1.65
1.46
1.43
1.40
1.38
1.35
1.33
1.32
1.29
1.25
1.22
1.20
1.19
1.16
N
N
F
F
F
N
N
NH
1.07
2.05
2.06
1.09
4.19
1.13
f1 (ppm)

1H NMR of 19

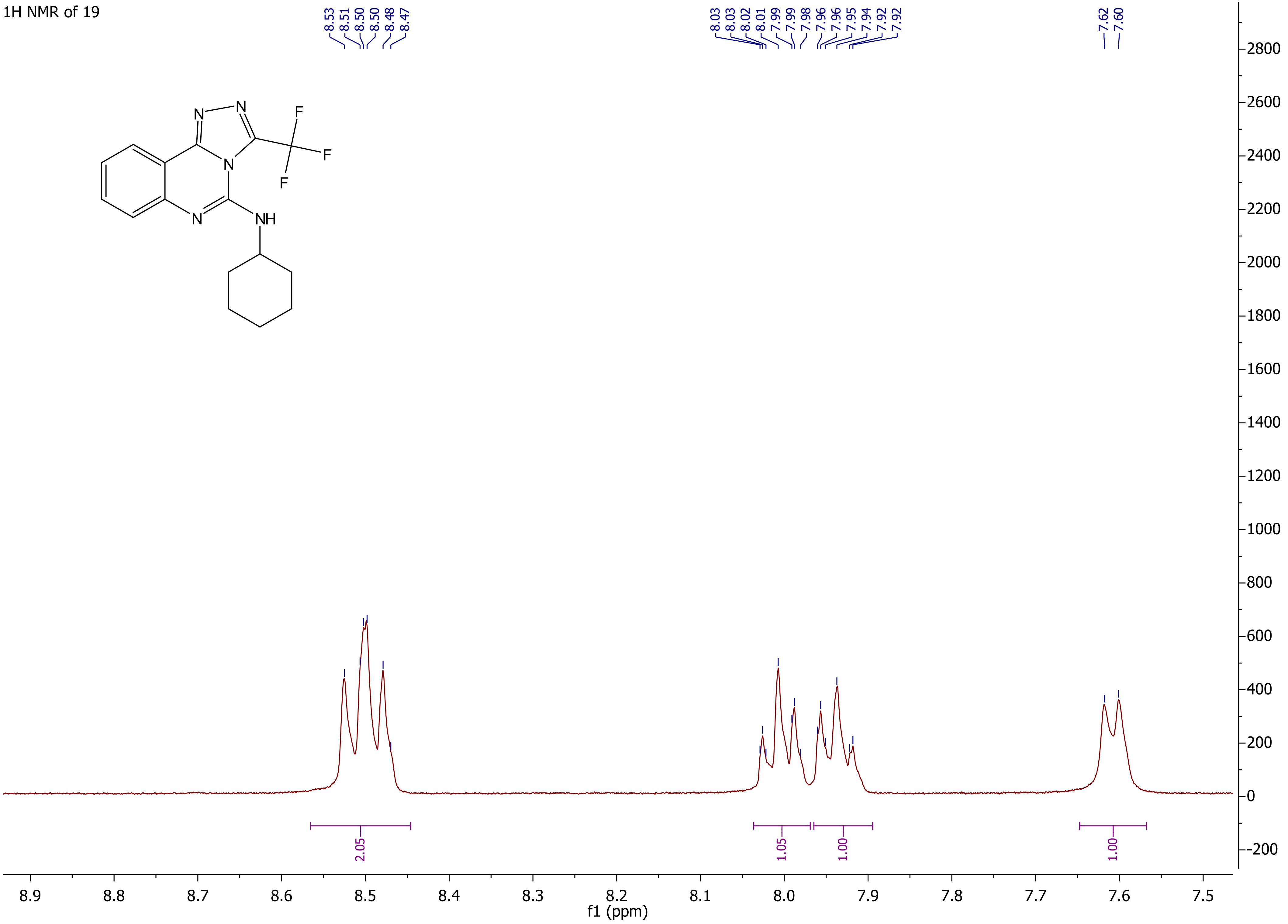
8.53
8.51
8.50
8.50
8.48
8.47
8.03
8.03
8.02
8.01
7.99
7.99
7.98
7.96
7.96
7.95
7.94
7.92
7.92
7.62
7.60
N
N
N
N
NH
F
F
F
2.05
1.05
1.00
1.00
f1 (ppm)

1H NMR of 20

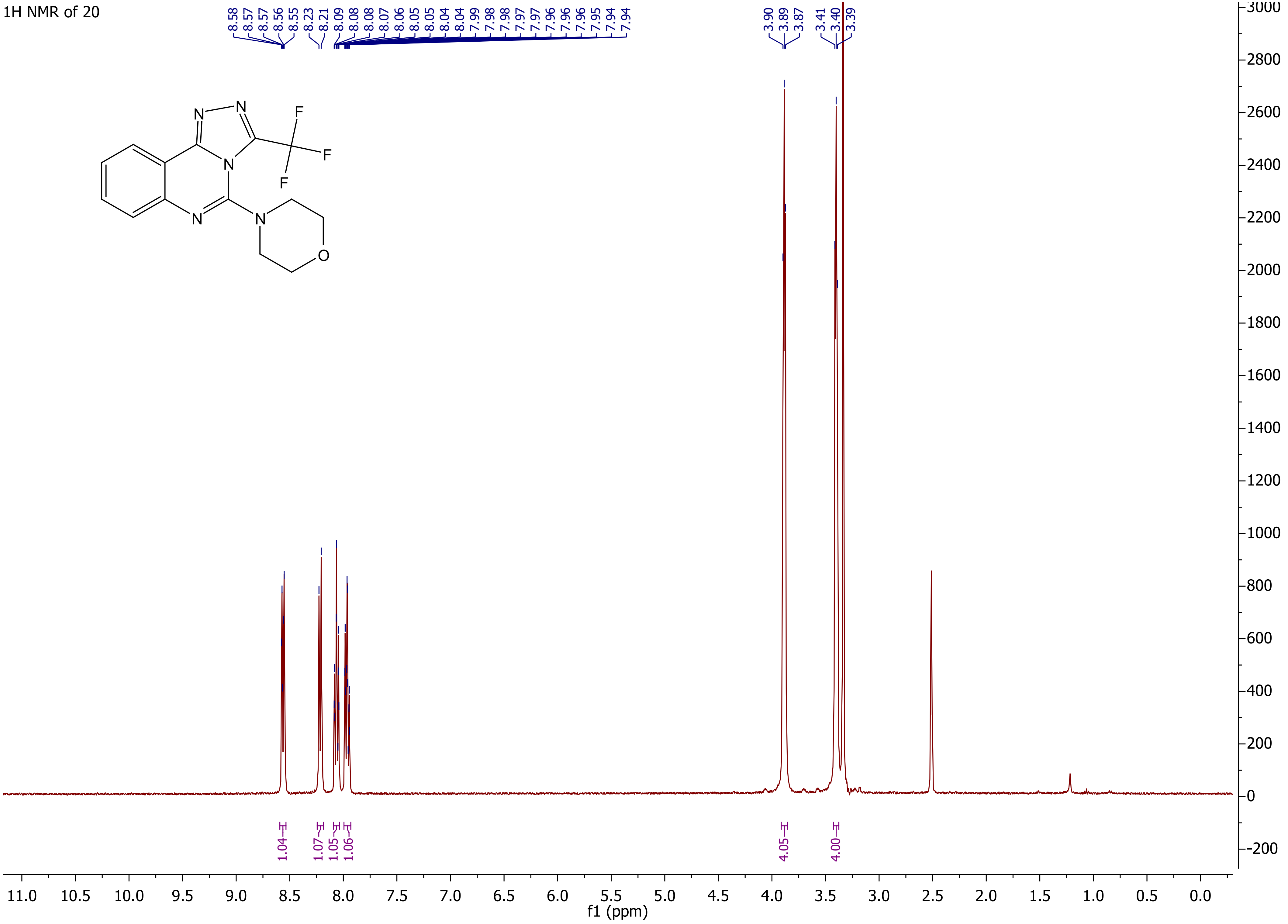

8.58
8.57
8.57
8.56
8.55
8.23
8.21
8.09
8.08
8.08
8.07
8.06
8.05
8.05
8.04
8.04
7.99
7.98
7.98
7.97
7.97
7.96
7.96
7.96
7.95
7.94
7.94
3.90
3.89
3.87
3.41
3.40
3.39
1.04
1.07
1.05
1.06
4.05
4.00
11.0 10.5 10.0 9.5 9.0 8.5 8.0 7.5 7.0 6.5 6.0 5.5 5.0 4.5 4.0 3.5 3.0 2.5 2.0 1.5 1.0 0.5 0.0
f1 (ppm)
3000 2800 2600 2400 2200 2000 1800 1600 1400 1200 1000 800 600 400 200 0 -200

1H NMR of 20

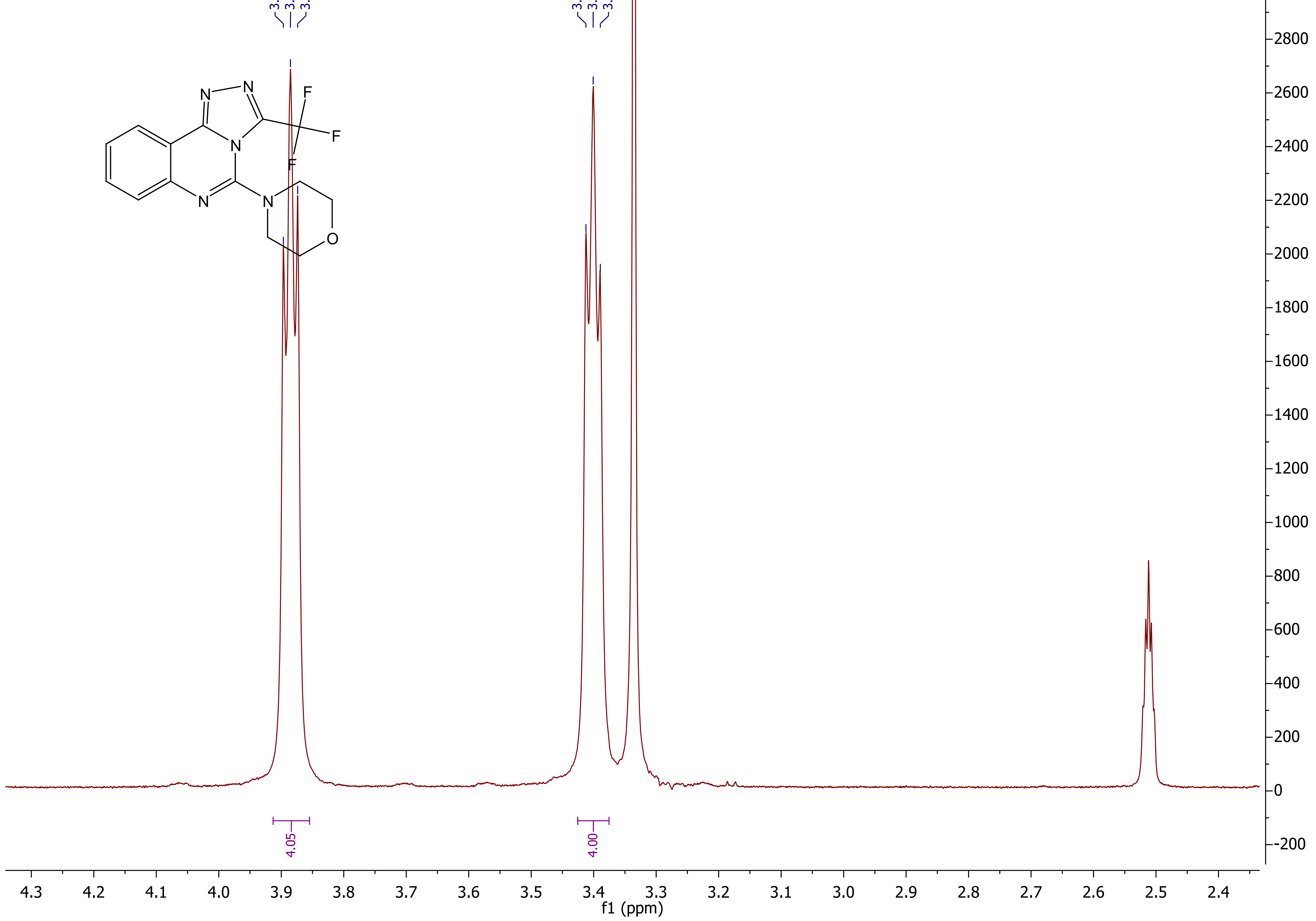

1H NMR of 20

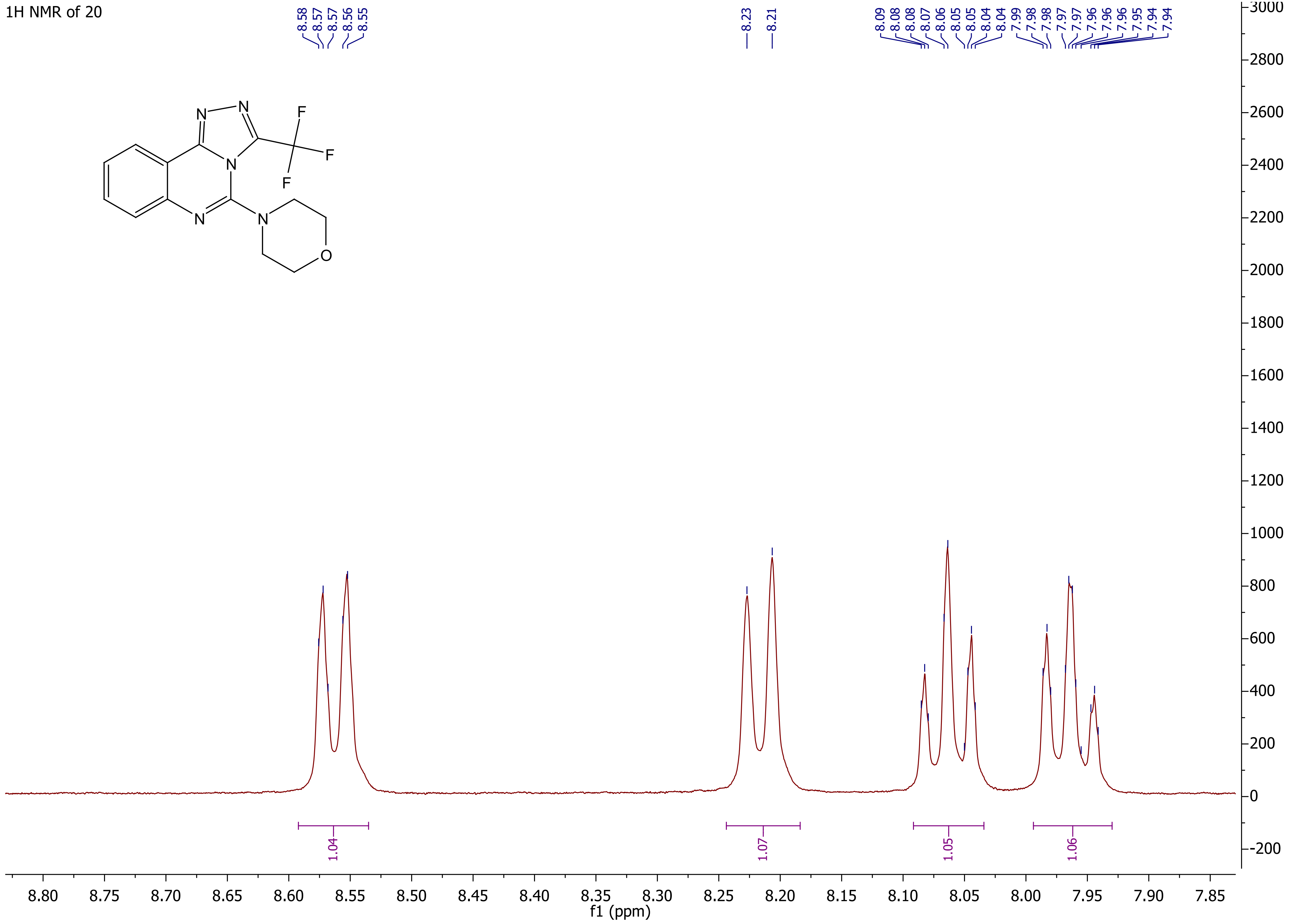
8.58
8.57
8.57
8.56
8.55
8.23
8.21
8.09
8.08
8.08
8.07
8.06
8.05
8.05
8.04
8.04
7.99
7.98
7.98
7.97
7.97
7.96
7.96
7.96
7.95
7.94
7.94
N
N
N
N
F
F
F
N
O
1.04
1.07
1.05
1.06
8.80 8.75 8.70 8.65 8.60 8.55 8.50 8.45 8.40 8.35 8.30 8.25 8.20 8.15 8.10 8.05 8.00 7.95 7.90 7.85
f1 (ppm)
3000 2800 2600 2400 2200 2000 1800 1600 1400 1200 1000 800 600 400 200 0 -200

13C NMR 13b

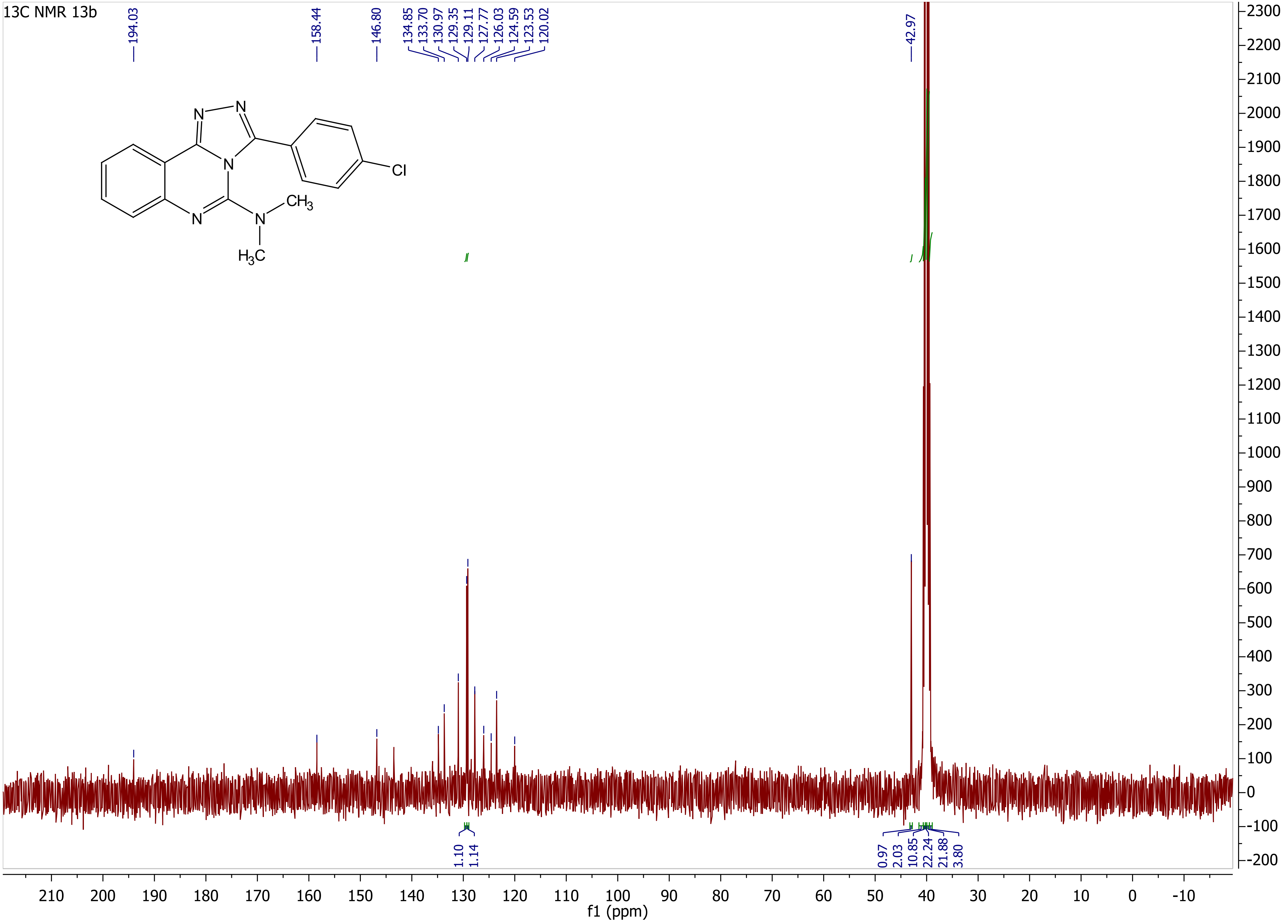

194.03
158.44
146.80
134.85
133.70
130.97
129.35
129.11
127.77
126.03
124.59
123.53
120.02
42.97
N
N
N
N
Cl
CH3
H3C
N
1.10
1.14
0.97
2.03
10.85
22.24
21.88
3.80
f1 (ppm)

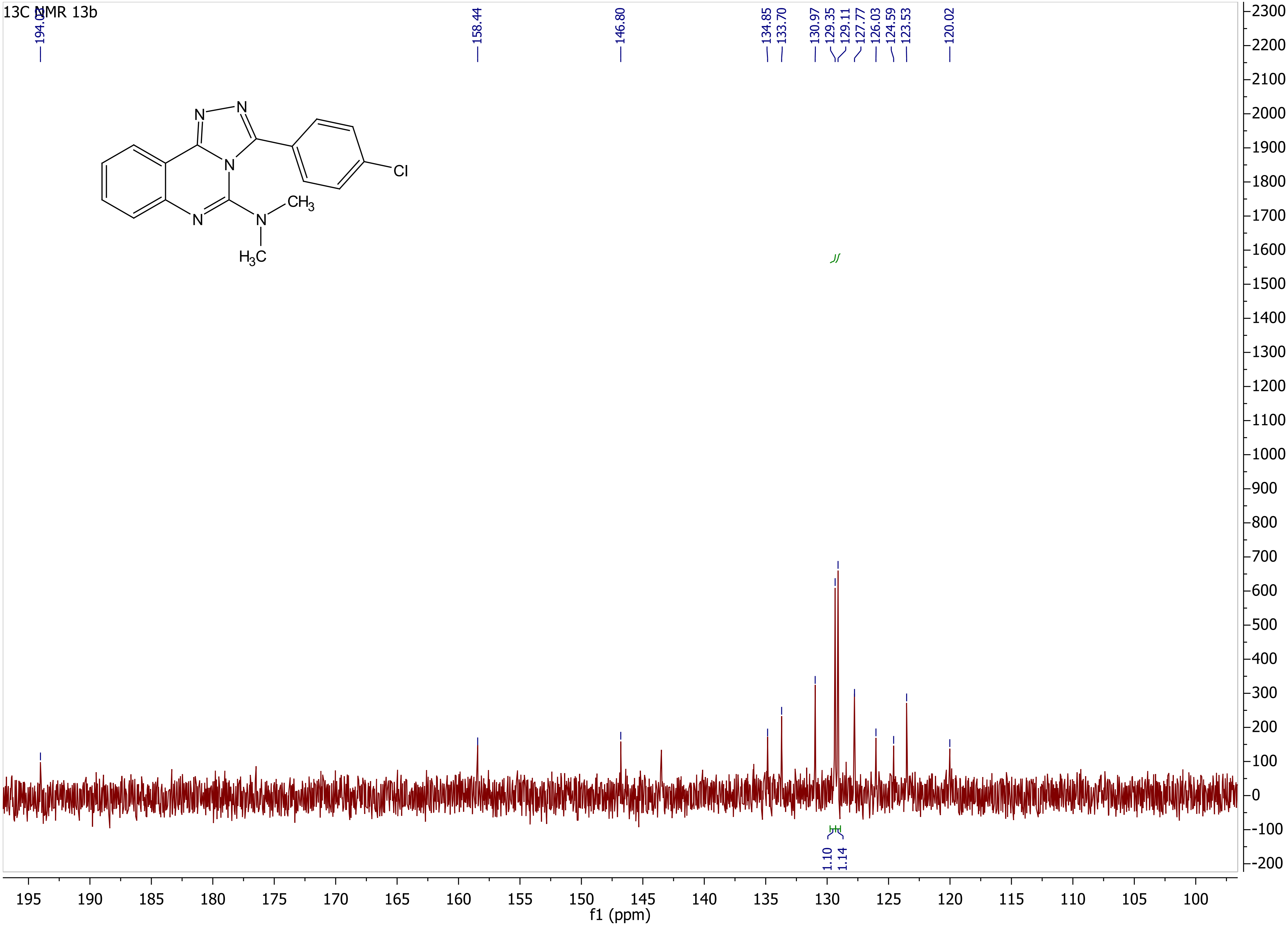
13C NMR 13b
194.0
158.44
146.80
134.85
133.70
130.97
129.35
129.11
127.77
126.03
124.59
123.53
120.02
1.10
1.14
N
N
N
N
N
CH3
H3C
Cl
f1 (ppm)

13C NMR 15b

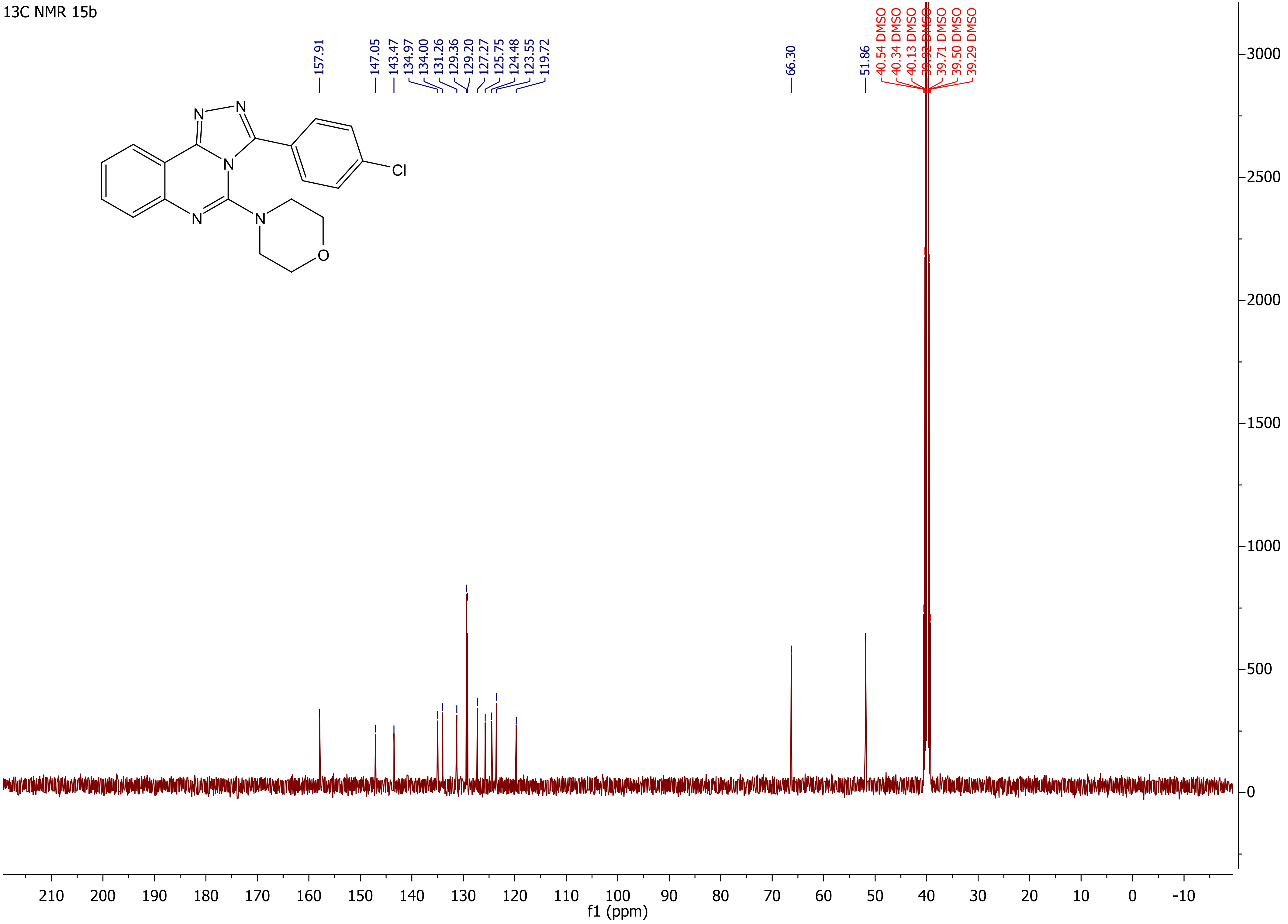

157.91
147.05
143.47
134.97
134.00
131.26
129.36
129.20
127.27
125.75
124.48
123.55
119.72
66.30
51.86
40.54 DMSO
40.34 DMSO
40.13 DMSO
39.92 DMSO
39.71 DMSO
39.50 DMSO
39.29 DMSO
N
N
N
N
N
O
Cl
3000
2500
2000
1500
1000
500
0
210 200 190 180 170 160 150 140 130 120 110 100 90 80 70 60 50 40 30 20 10 0 -10
f1 (ppm)

13C NMR 15b

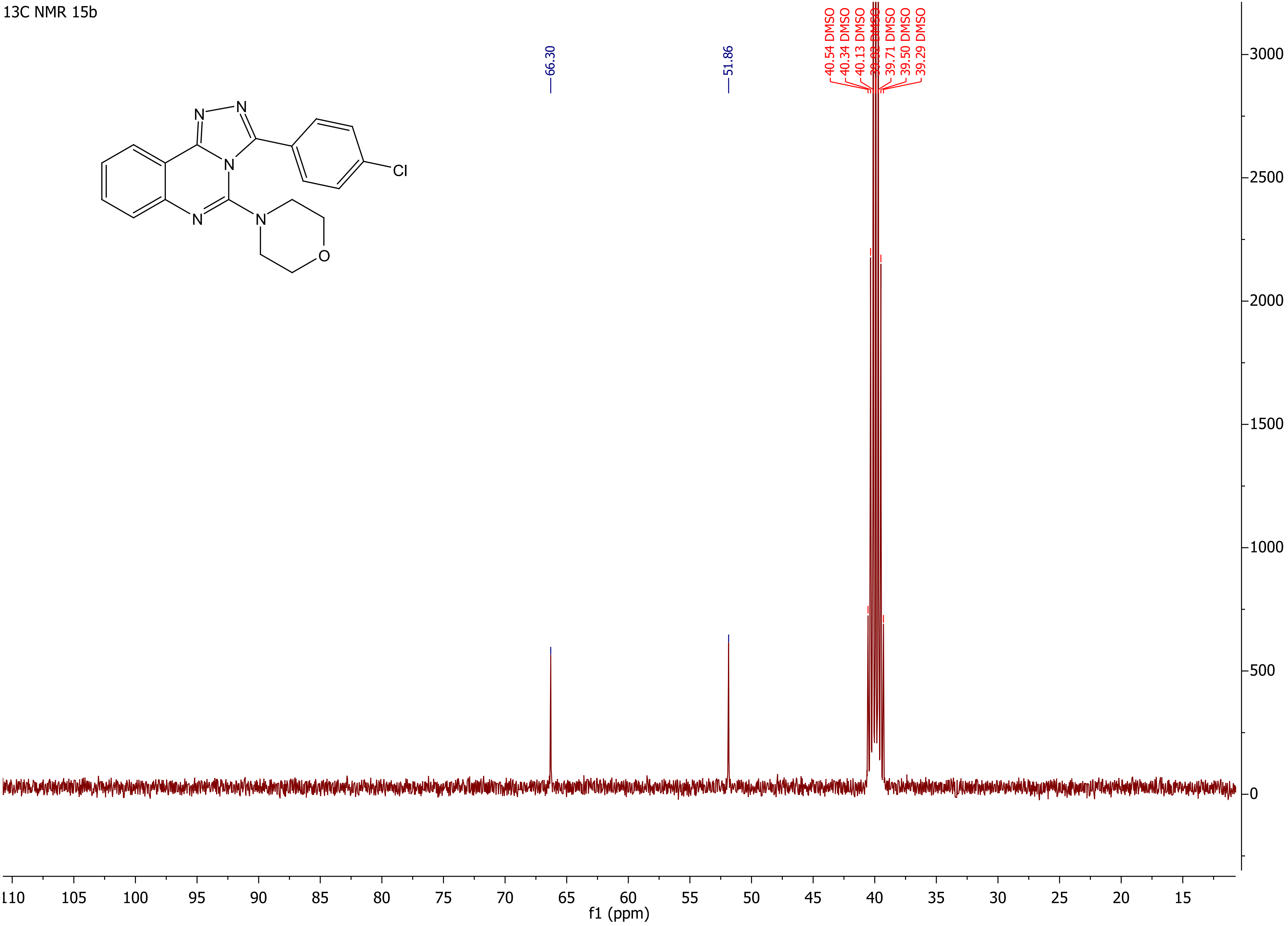

66.30
51.86
40.54 DMSO
40.34 DMSO
40.13 DMSO
39.92 DMSO
39.71 DMSO
39.50 DMSO
39.29 DMSO
N
N
N
N
N
O
Cl
3000
2500
2000
1500
1000
500
0
110
105
100
95
90
85
80
75
70
65
60
55
50
45
40
35
30
25
20
15
f1 (ppm)

13C NMR 15b

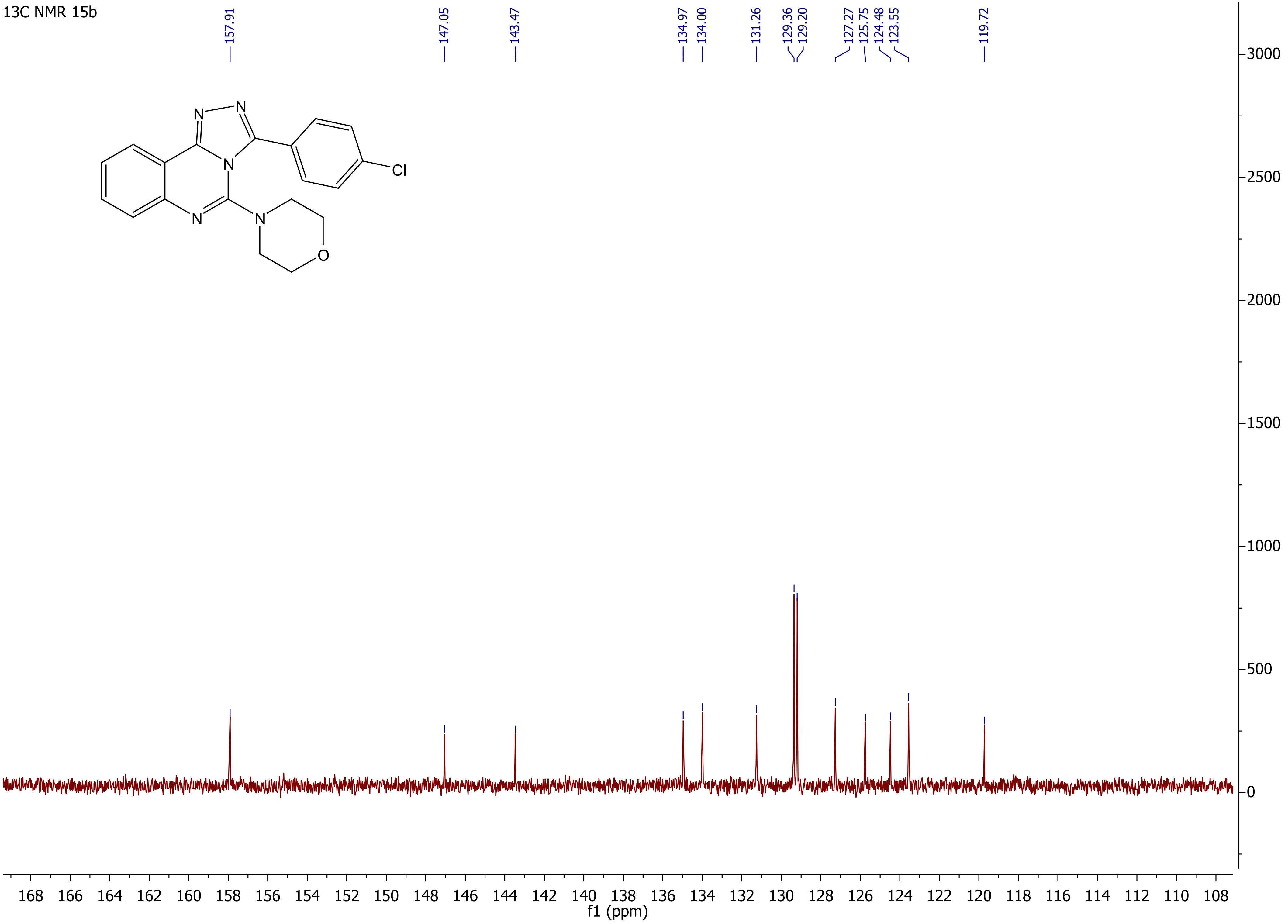

157.91
147.05
143.47
134.97
134.00
131.26
129.36
129.20
127.27
125.75
124.48
123.55
119.72
N
N
N
N
N
O
Cl
3000
2500
2000
1500
1000
500
0
168 166 164 162 160 158 156 154 152 150 148 146 144 142 140 138 136 134 132 130 128 126 124 122 120 118 116 114 112 110 108
f1 (ppm)

13C NMR of 16

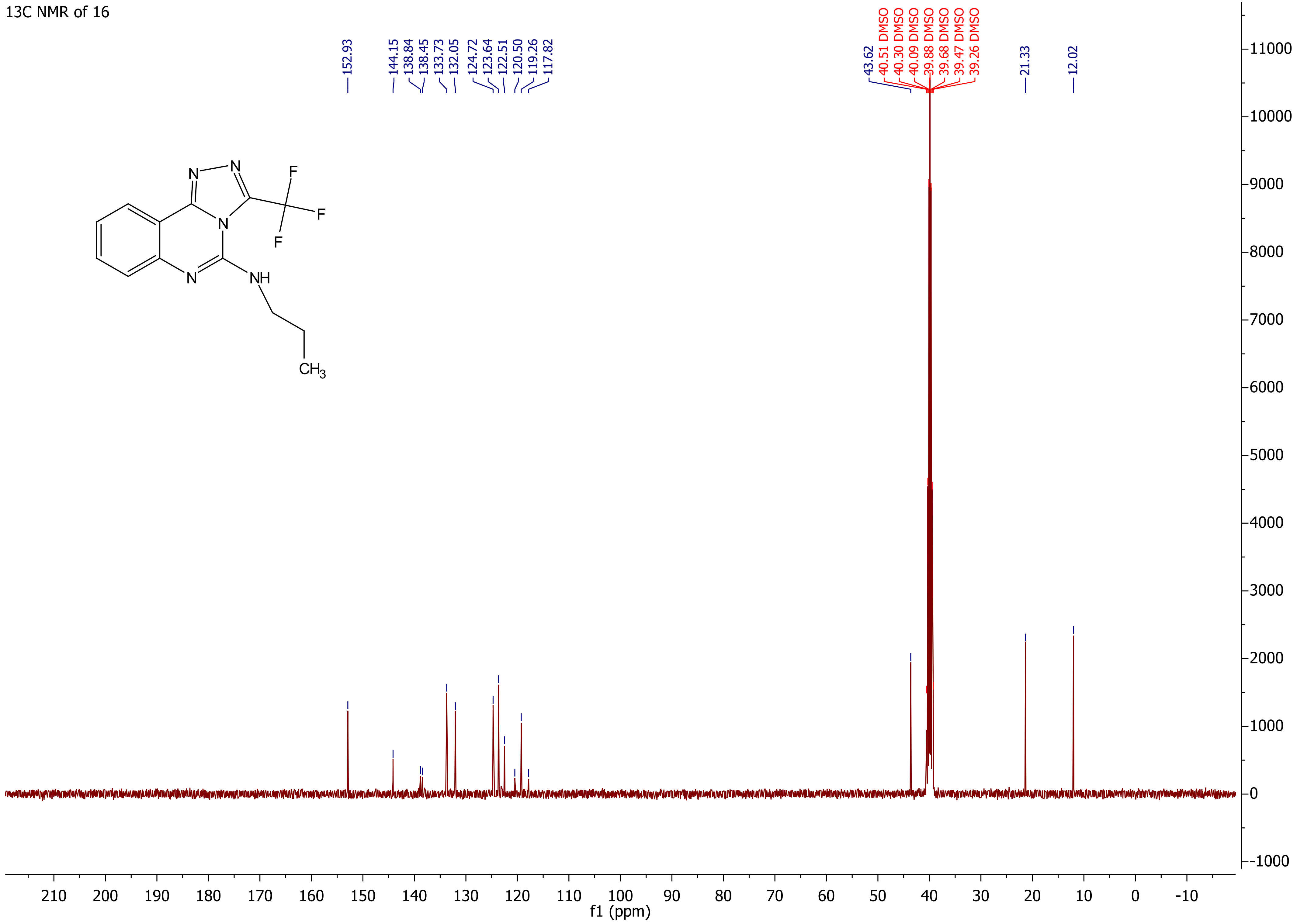

152.93
144.15
138.84
138.45
133.73
132.05
124.72
123.64
122.51
120.50
119.26
117.82
43.62
40.51 DMSO
40.30 DMSO
40.09 DMSO
39.88 DMSO
39.68 DMSO
39.47 DMSO
39.26 DMSO
21.33
12.02
N
N
N
N
F
F
F
NH
CH3
f1 (ppm)

13C NMR of 16

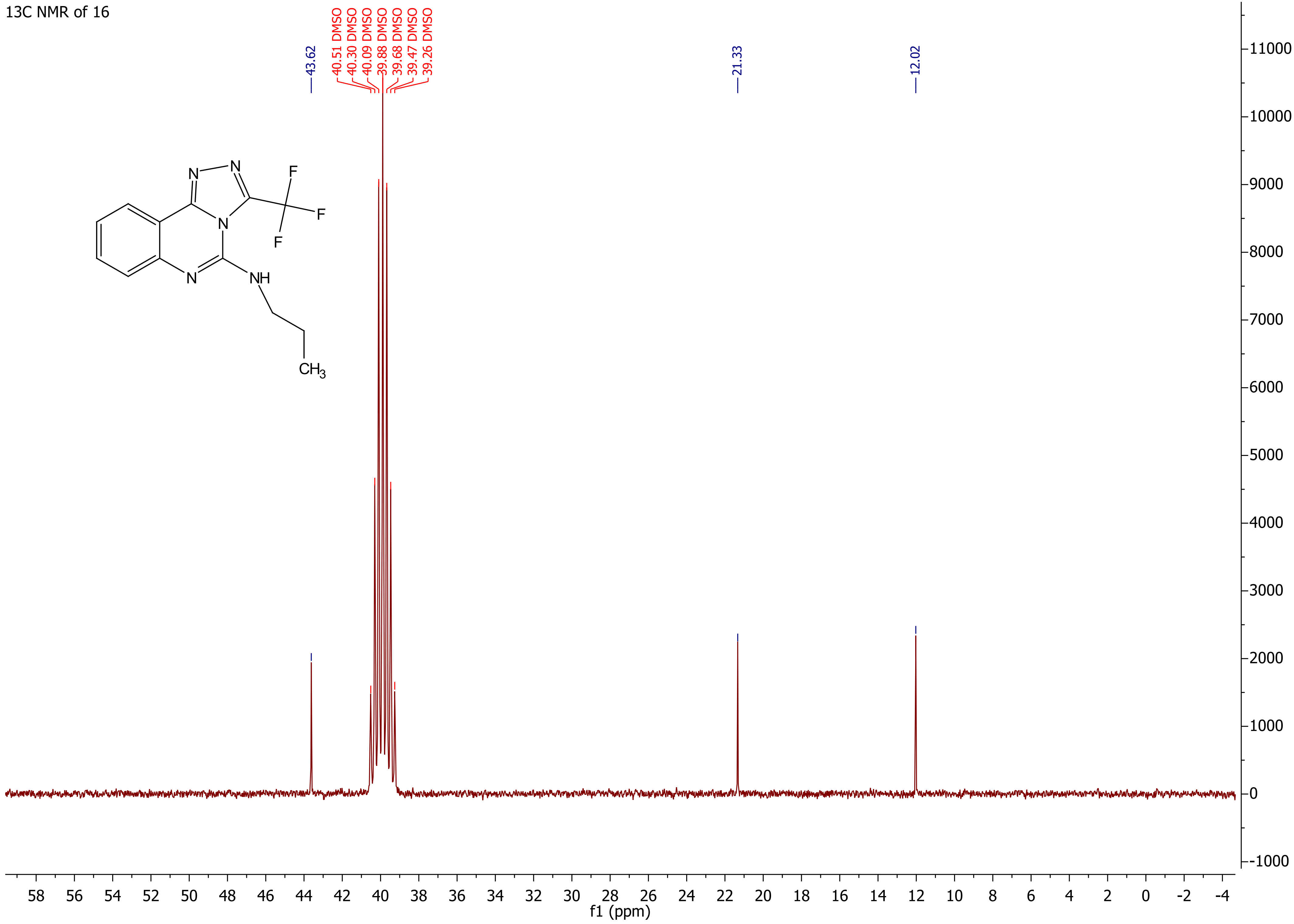

43.62
40.51 DMSO
40.30 DMSO
40.09 DMSO
39.88 DMSO
39.68 DMSO
39.47 DMSO
39.26 DMSO
21.33
12.02
N
N
N
N
NH
F
F
F
CH3
11000
10000
9000
8000
7000
6000
5000
4000
3000
2000
1000
0
-1000
58 56 54 52 50 48 46 44 42 40 38 36 34 32 30 28 26 24 22 20 18 16 14 12 10 8 6 4 2 0 -2 -4
f1 (ppm)

13C NMR of 16

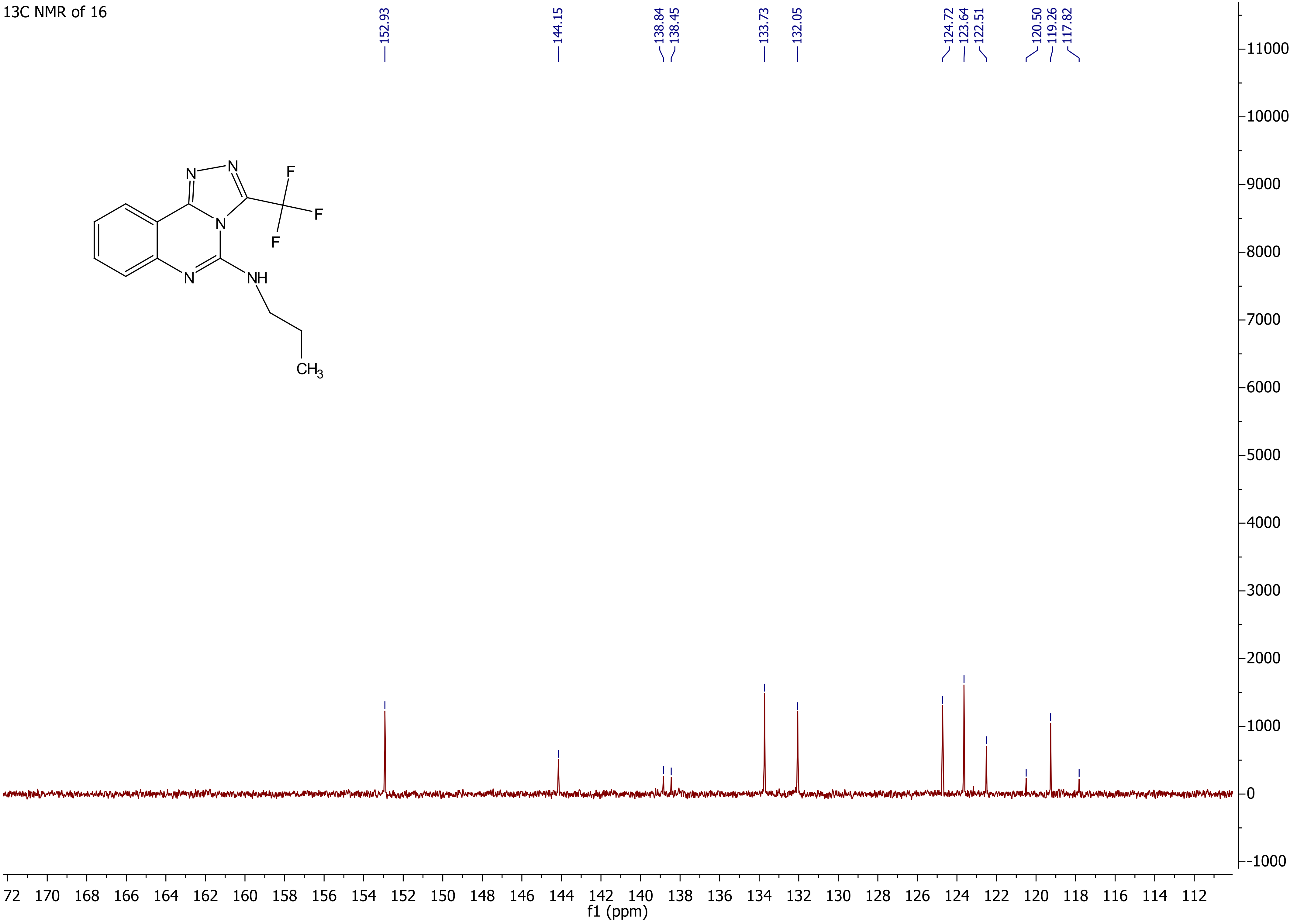

152.93
144.15
138.84
138.45
133.73
132.05
124.72
123.64
122.51
120.50
119.26
117.82
N
N
N
N
F
F
F
NH
CH3
11000
10000
9000
8000
7000
6000
5000
4000
3000
2000
1000
0
-1000
72 170 168 166 164 162 160 158 156 154 152 150 148 146 144 142 140 138 136 134 132 130 128 126 124 122 120 118 116 114 112
f1 (ppm)

13C NMR of 17

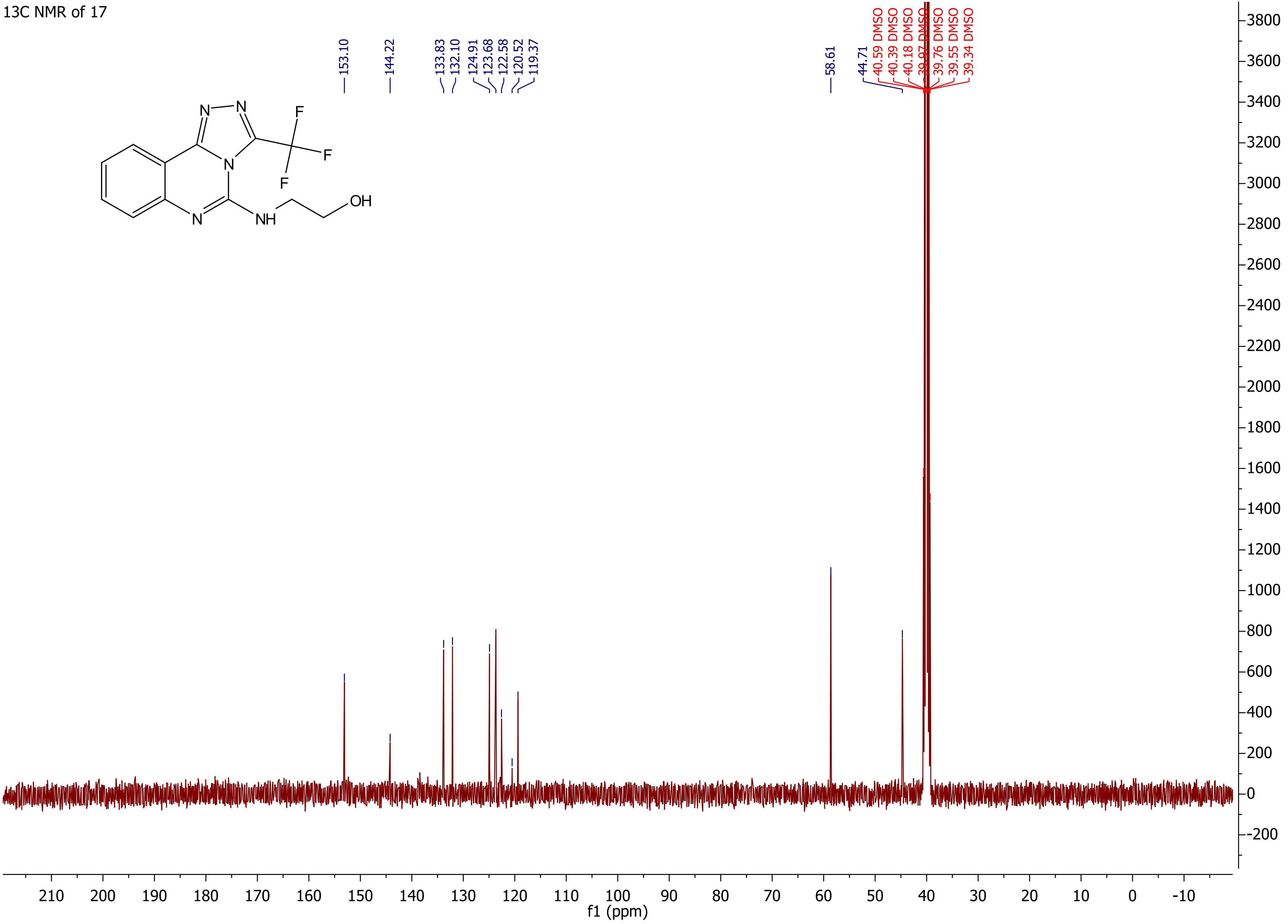

153.10
144.22
133.83
132.10
124.91
123.68
122.58
120.52
119.37
58.61
44.71
40.59 DMSO
40.39 DMSO
40.18 DMSO
39.97 DMSO
39.76 DMSO
39.55 DMSO
39.34 DMSO
N
N
F
F
F
N
N
NH
OH
f1 (ppm)
210 200 190 180 170 160 150 140 130 120 110 100 90 80 70 60 50 40 30 20 10 0 -10
3800 3600 3400 3200 3000 2800 2600 2400 2200 2000 1800 1600 1400 1200 1000 800 600 400 200 0 -200

13C NMR of 17

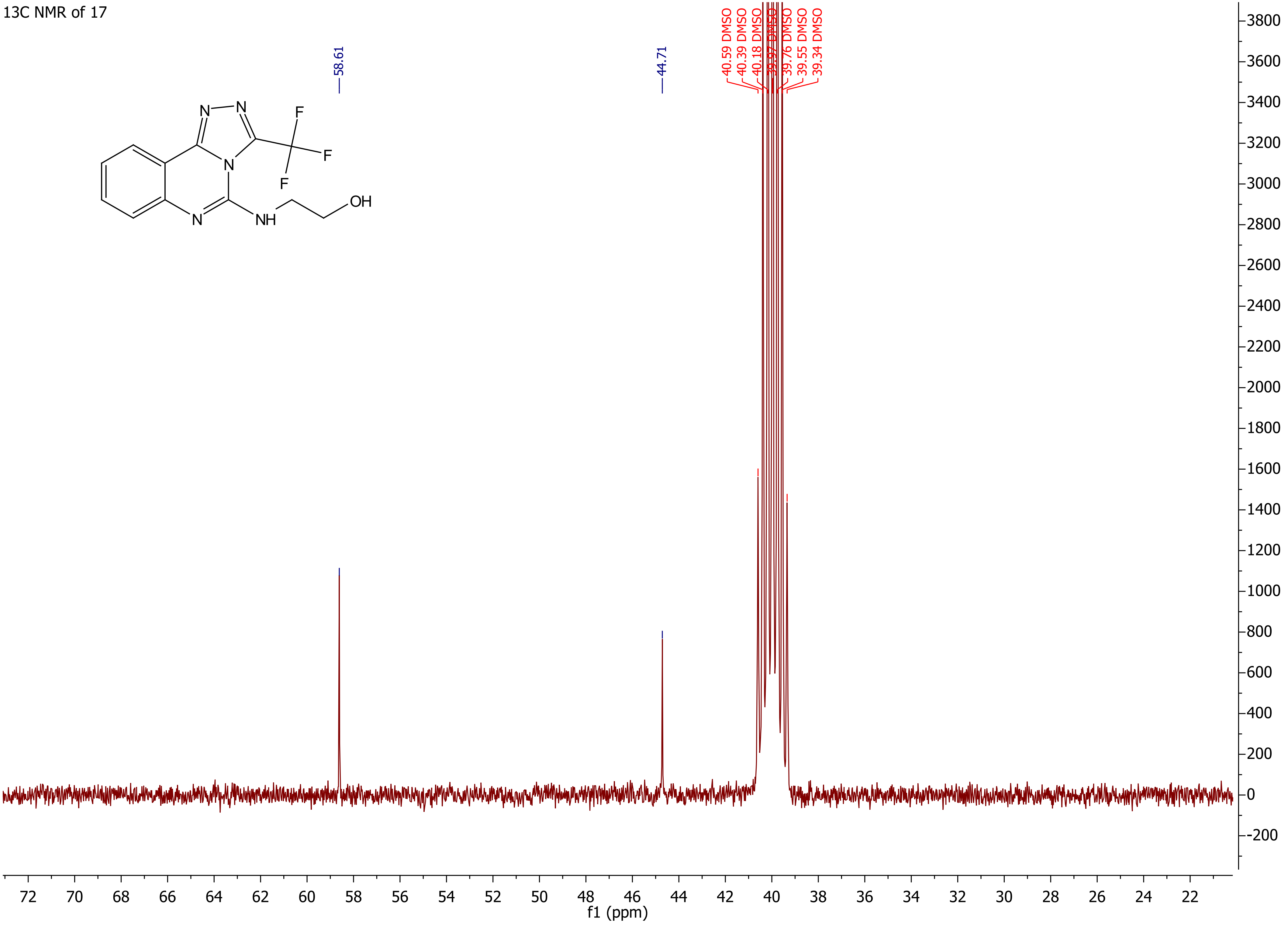
58.61
44.71
40.59 DMSO
40.39 DMSO
40.18 DMSO
39.97 DMSO
39.76 DMSO
39.55 DMSO
39.34 DMSO
f1 (ppm)

13C NMR of 17

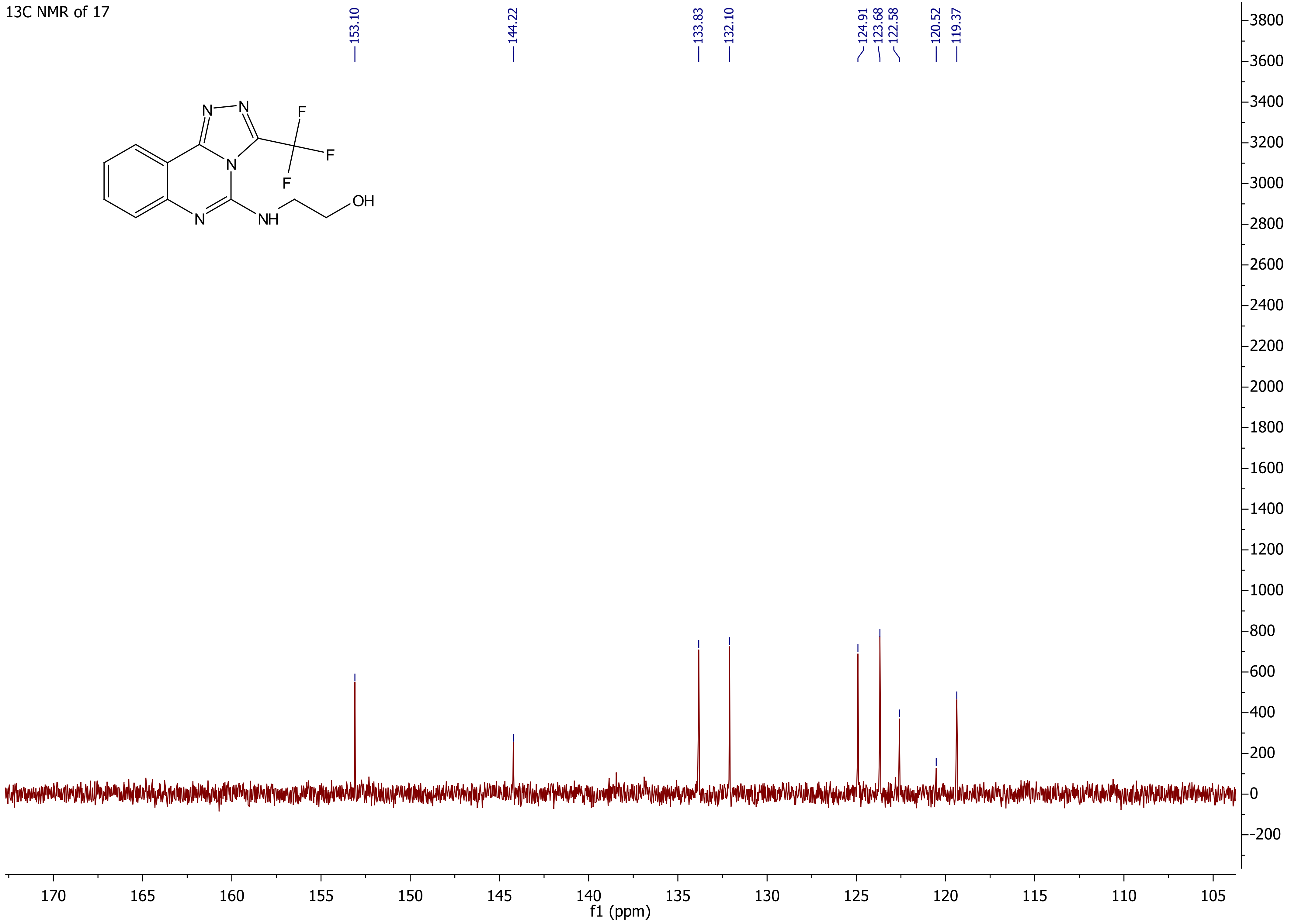

153.10
144.22
133.83
132.10
124.91
123.68
122.58
120.52
119.37
f1 (ppm)

13C NMR of 18

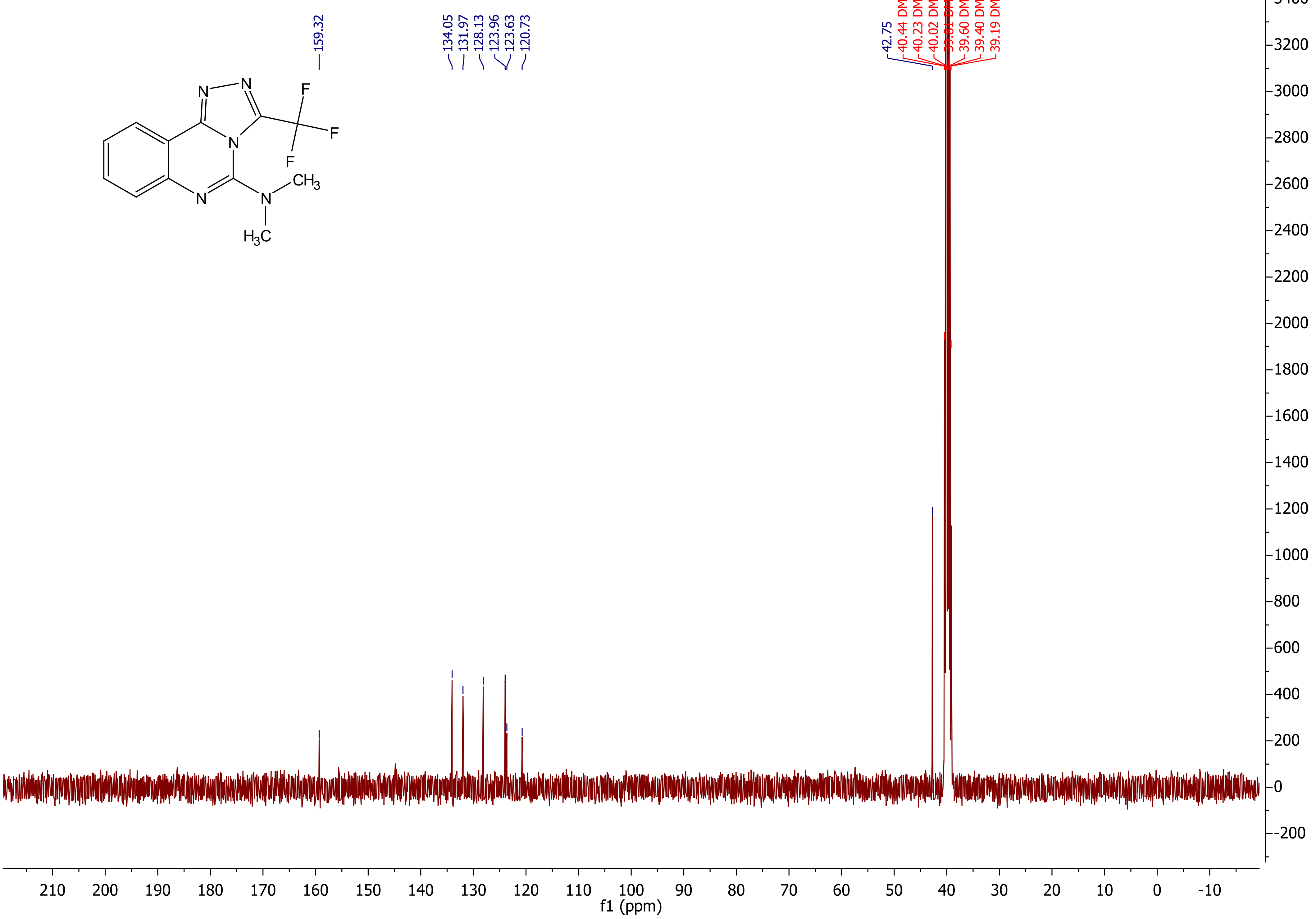

159.32
134.05
131.97
128.13
123.96
123.63
120.73
42.75
40.44 DMSO
40.23 DMSO
40.02 DMSO
39.81 DMSO
39.60 DMSO
39.40 DMSO
39.19 DMSO
f1 (ppm)

13C NMR of 18

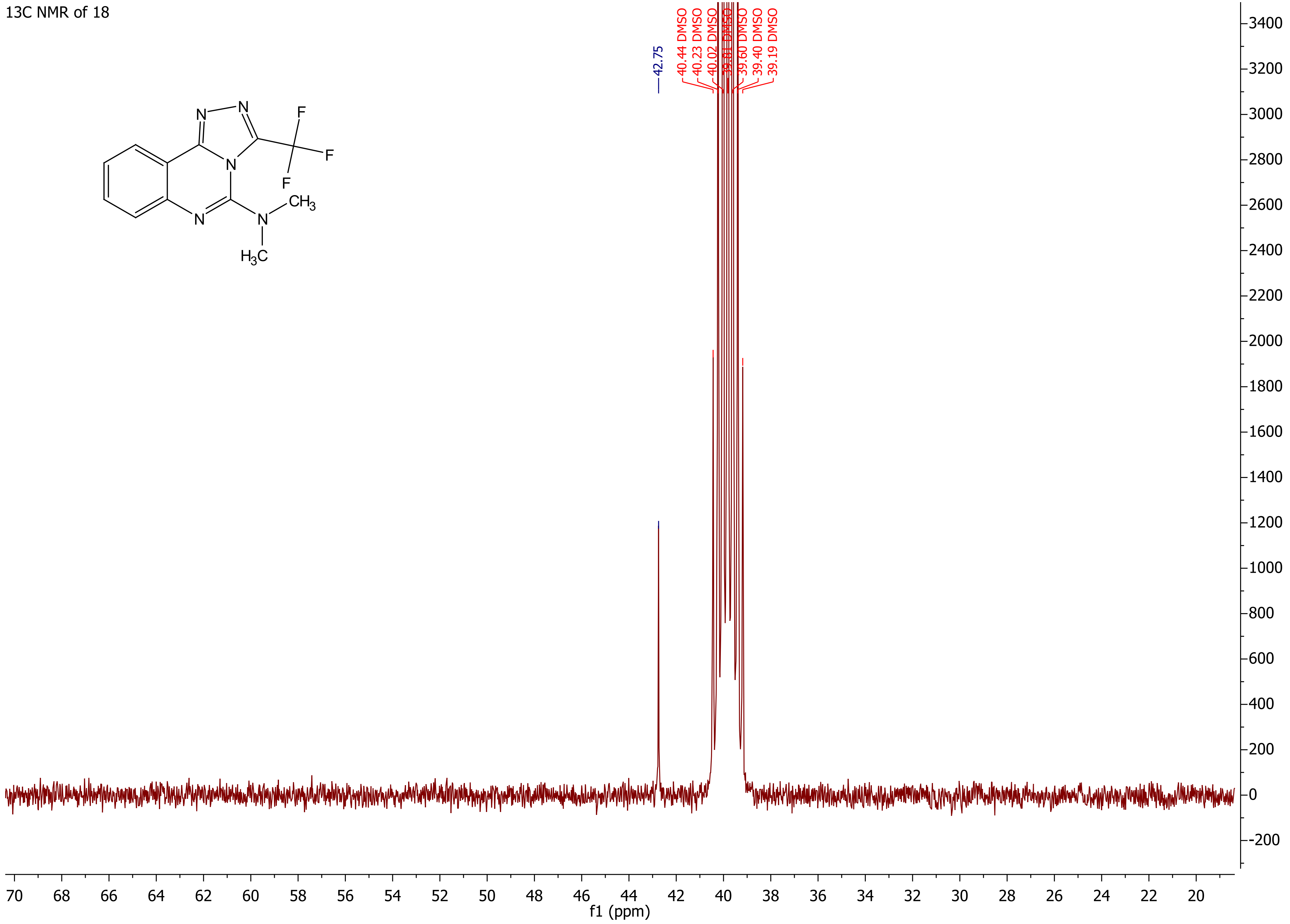

N
N
F
F
F
N
N
N
CH3
H3C
42.75
40.44 DMSO
40.23 DMSO
40.02 DMSO
39.81 DMSO
39.60 DMSO
39.40 DMSO
39.19 DMSO
f1 (ppm)

13C NMR of 18

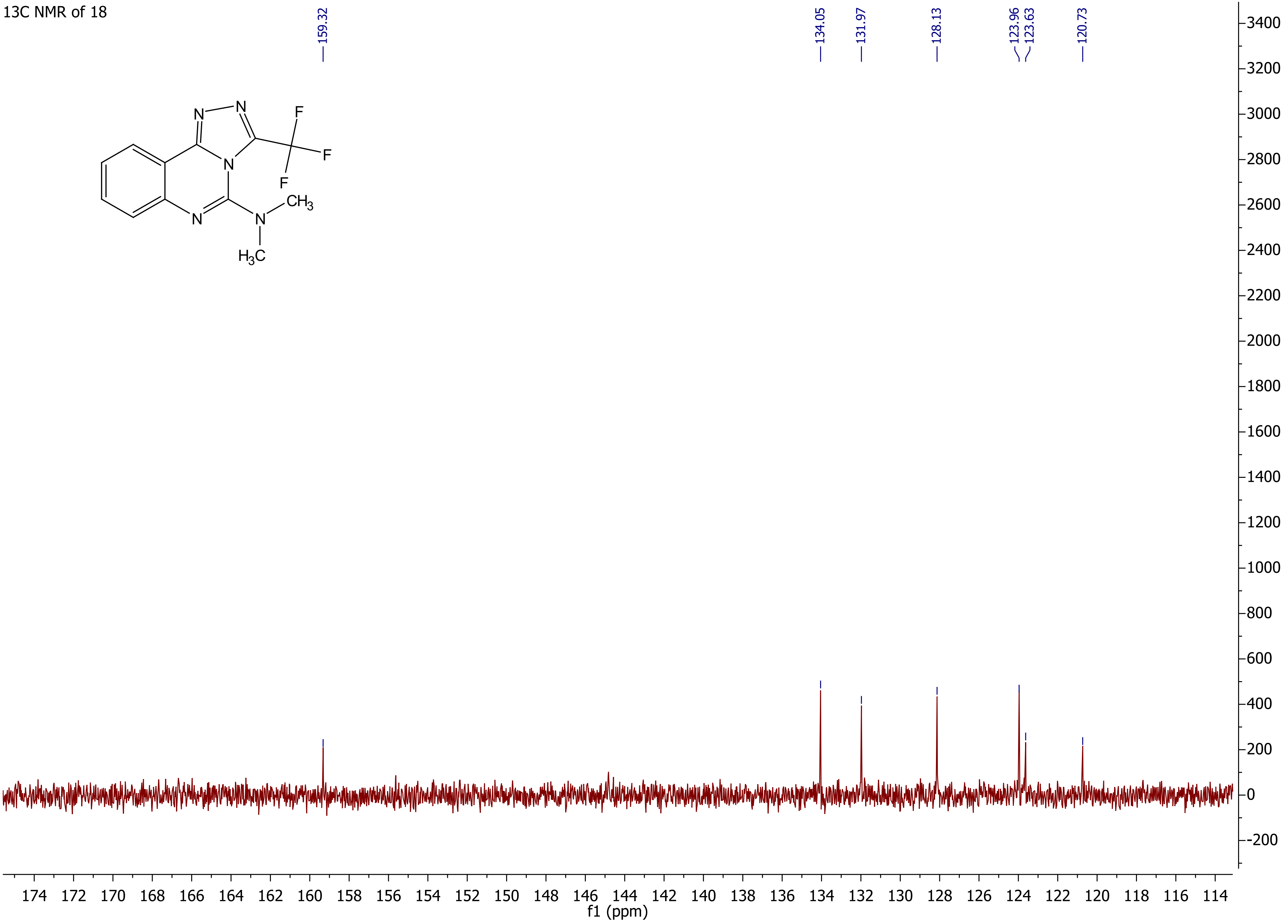

159.32
134.05
131.97
128.13
123.96
123.63
120.73
3400
3200
3000
2800
2600
2400
2200
2000
1800
1600
1400
1200
1000
800
600
400
200
0
-200
174 172 170 168 166 164 162 160 158 156 154 152 150 148 146 144 142 140 138 136 134 132 130 128 126 124 122 120 118 116 114
f1 (ppm)

13C NMR of 19

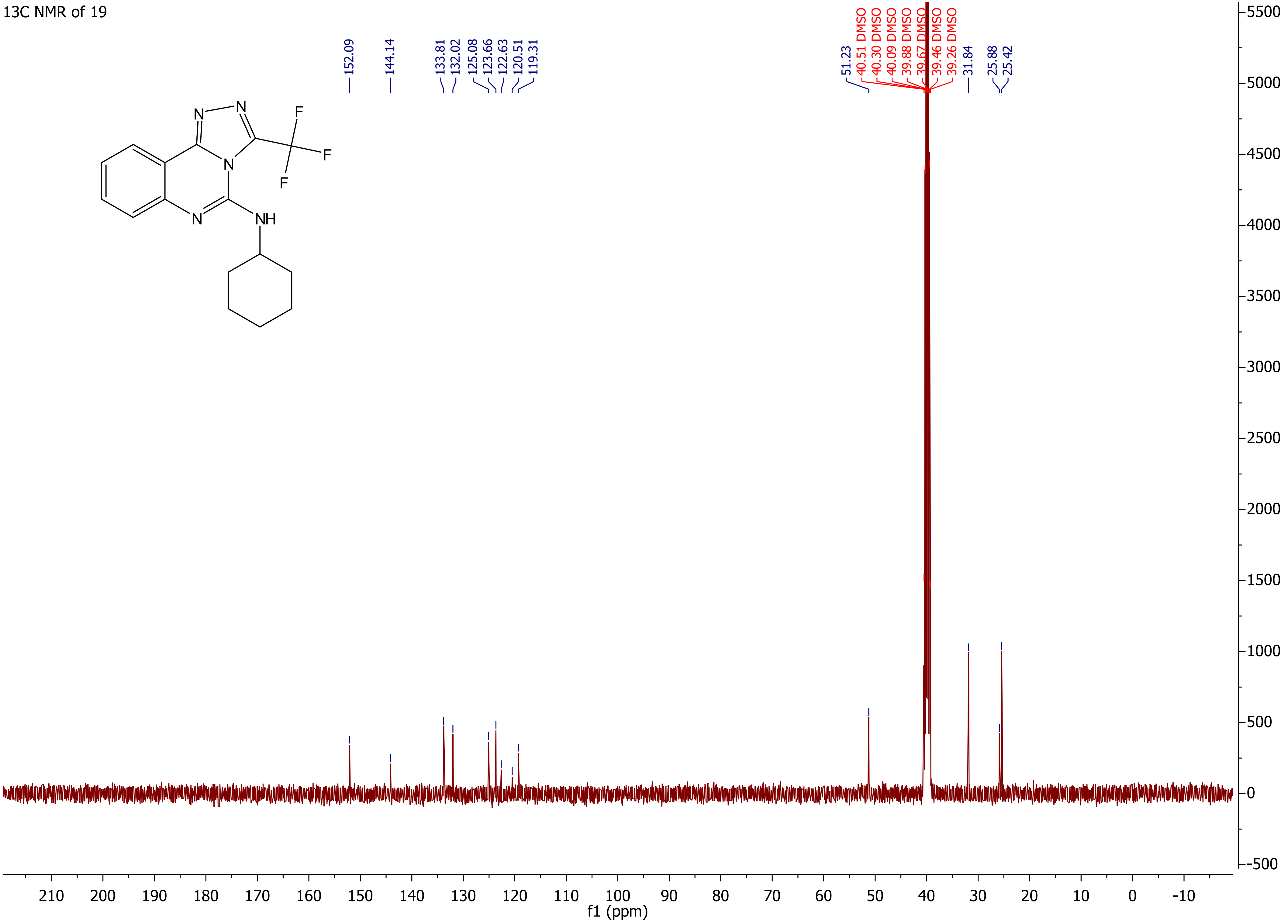

152.09
144.14
133.81
132.02
125.08
123.66
122.63
120.51
119.31
51.23
40.51 DMSO
40.30 DMSO
40.09 DMSO
39.88 DMSO
39.67 DMSO
39.46 DMSO
39.26 DMSO
31.84
25.88
25.42
N
N
F
F
F
N
N
NH
f1 (ppm)

13C NMR of 19

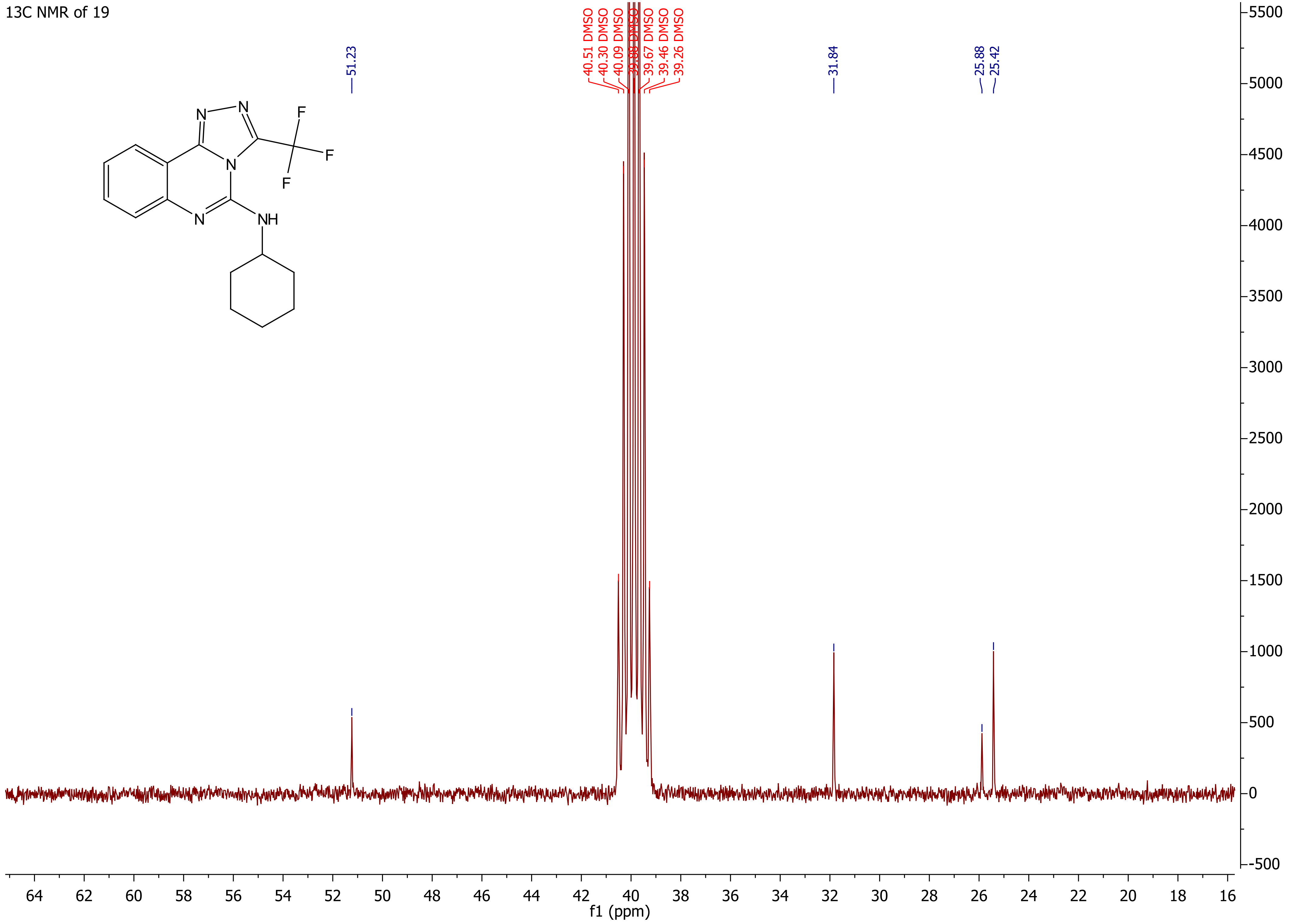

51.23
40.51 DMSO
40.30 DMSO
40.09 DMSO
39.88 DMSO
39.67 DMSO
39.46 DMSO
39.26 DMSO
31.84
25.88
25.42
5500
5000
4500
4000
3500
3000
2500
2000
1500
1000
500
0
-500
64
62
60
58
56
54
52
50
48
46
44
42
40
38
36
34
32
30
28
26
24
22
20
18
16
f1 (ppm)

13C NMR of 19

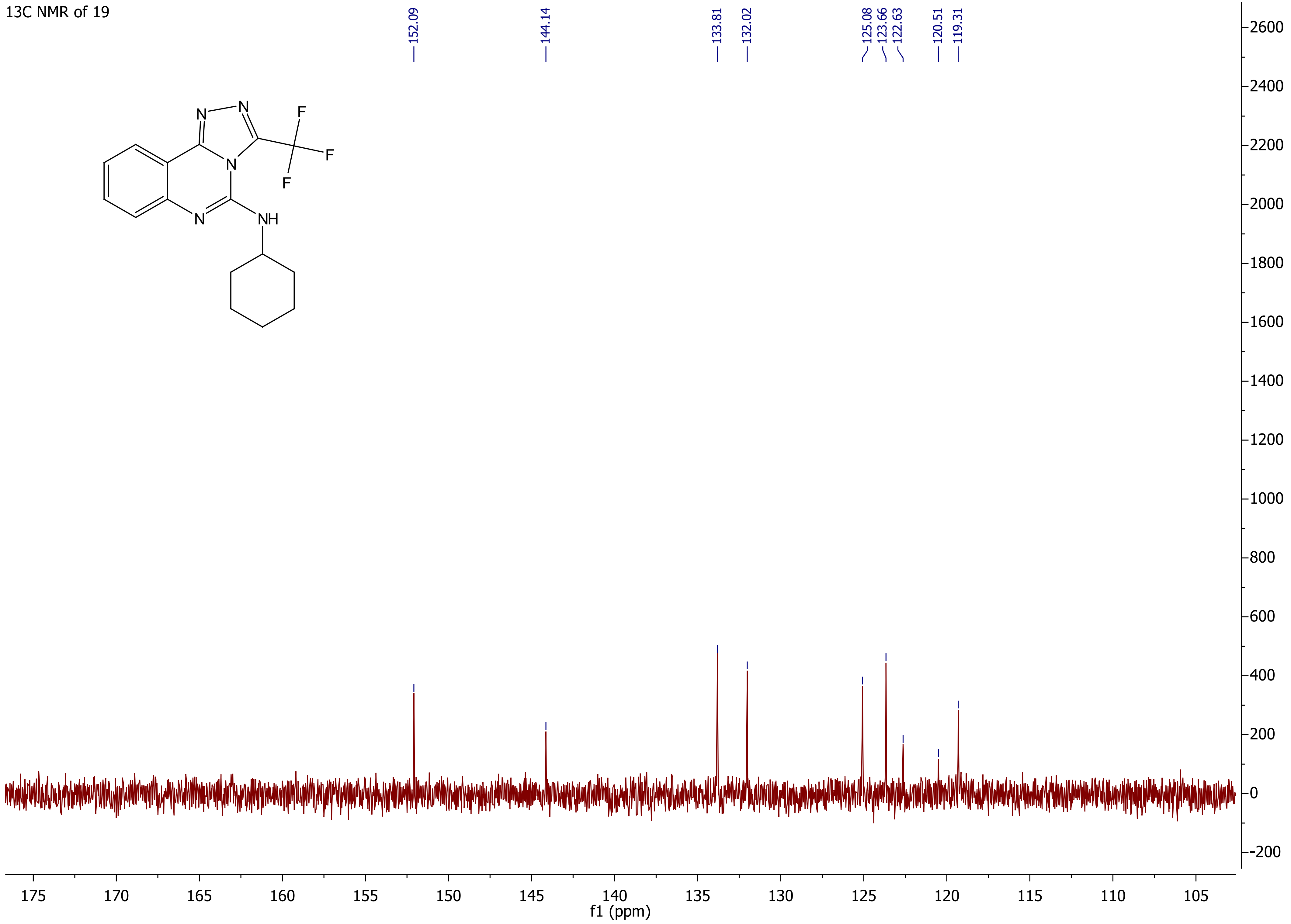

152.09
144.14
133.81
132.02
125.08
123.66
122.63
120.51
119.31
N
N
F
F
N
F
N
NH
f1 (ppm)
175
170
165
160
155
150
145
140
135
130
125
120
115
110
105
2600
2400
2200
2000
1800
1600
1400
1200
1000
800
600
400
200
0
-200

13C NMR of 20

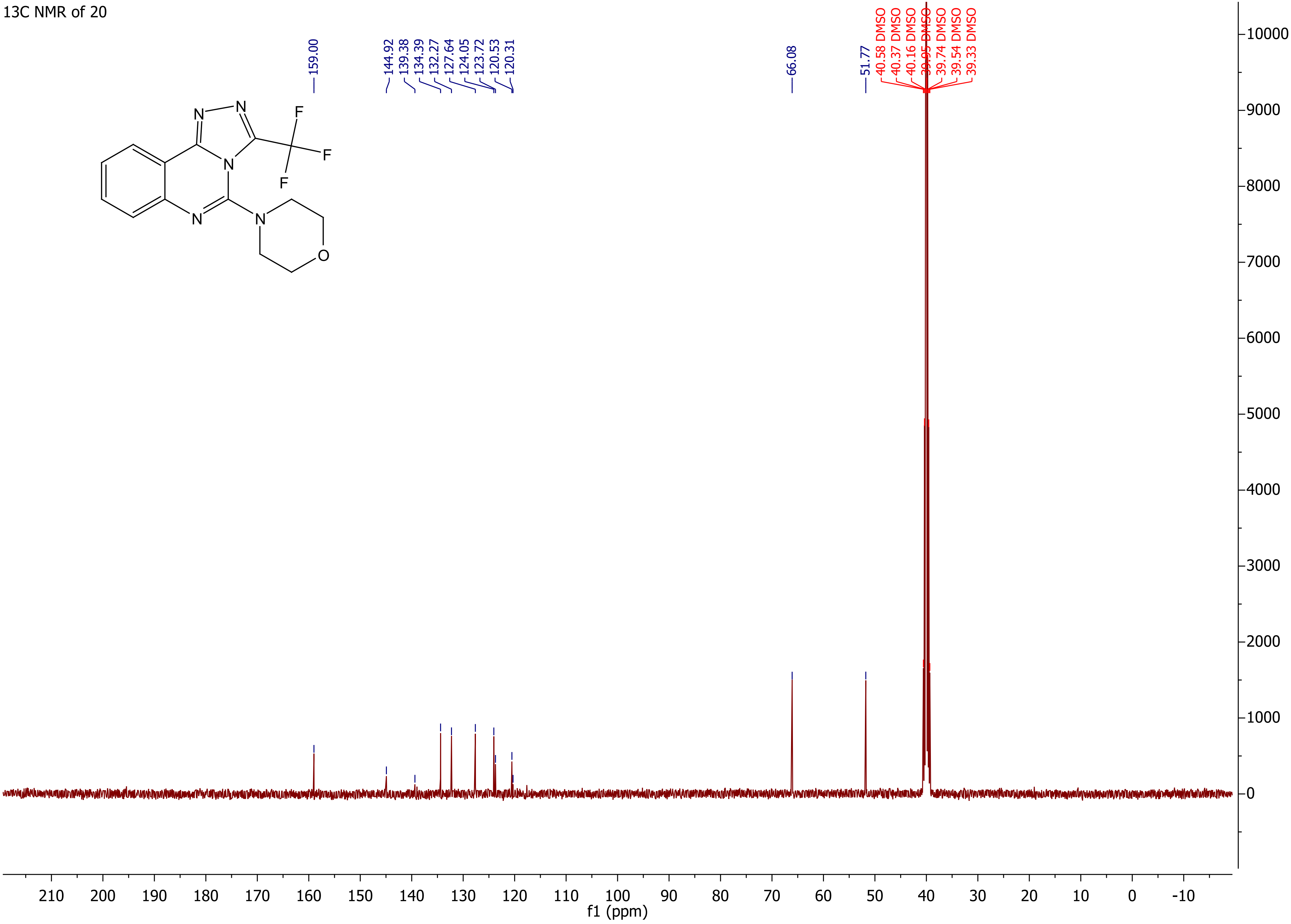

159.00
144.92
139.38
134.39
132.27
127.64
124.05
123.72
120.53
120.31
66.08
51.77
40.58 DMSO
40.37 DMSO
40.16 DMSO
39.95 DMSO
39.74 DMSO
39.54 DMSO
39.33 DMSO
f1 (ppm)

13C NMR of 20

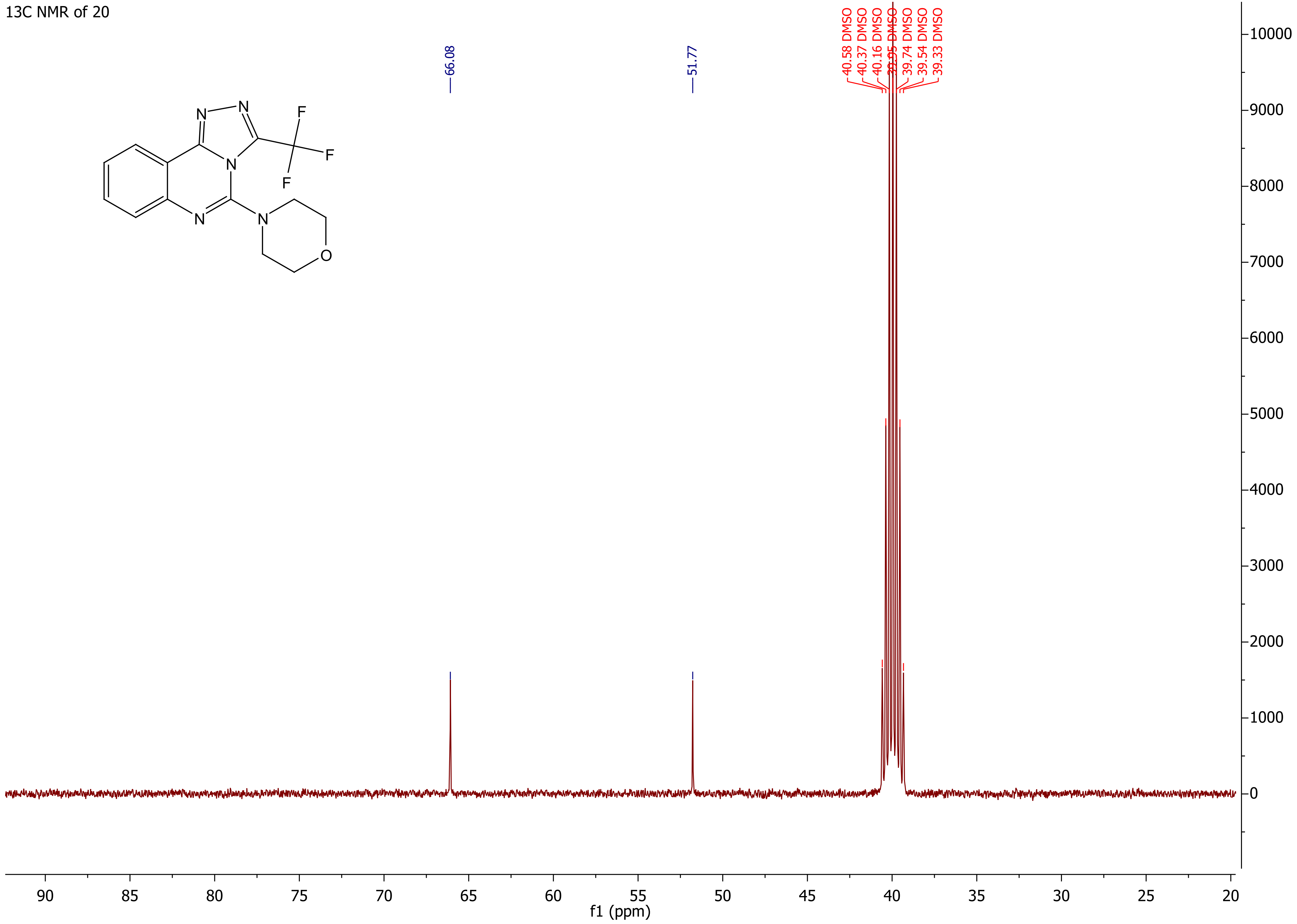
66.08
51.77
40.58 DMSO
40.37 DMSO
40.16 DMSO
39.95 DMSO
39.74 DMSO
39.54 DMSO
39.33 DMSO
N
N
N
N
N
F
F
F
O
10000
9000
8000
7000
6000
5000
4000
3000
2000
1000
0
90
85
80
75
70
65
60
55
50
45
40
35
30
25
20
f1 (ppm)

13C NMR of 20

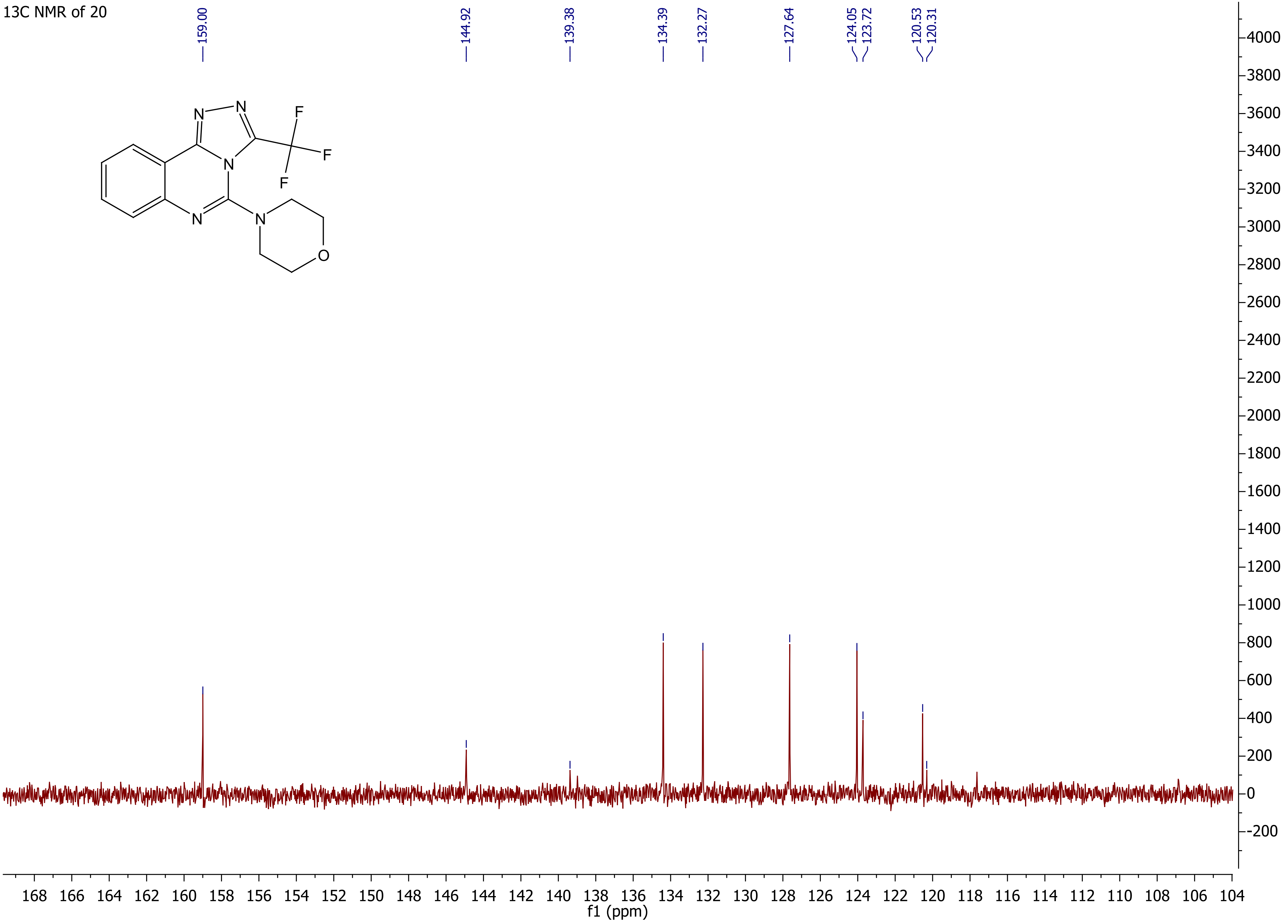

159.00
144.92
139.38
134.39
132.27
127.64
124.05
123.72
120.53
120.31
f1 (ppm)
